# Study on Design, Synthesis and Herbicidal Activity of Novel 4-Amino-6-(5-Aryl-Substituted-1-Pyrazolyl)-3-Chloro-5-Fluoro-2-Picolinic Acids

**DOI:** 10.3390/molecules30051022

**Published:** 2025-02-23

**Authors:** Huiting Li, Wei Wei, Rongchuan Shi, Yunhan Gou, Xiaofei Li, Chengyang Li, Yiqun Li, Yiming Cao, Shangzhong Liu

**Affiliations:** 1 Innovation Center of Pesticide Research, Department of Applied Chemistry, College of Science, China Agricultural University, Beijing 100193, China; bs20223100772@cau.edu.cn (H.L.); bs20233100863@cau.edu.cn (W.W.); shirongchuan@cau.edu.cn (R.S.); 2019310060123@cau.edu.cn (Y.G.); sy20233102324@cau.edu.cn (X.L.); sy20243102429@cau.edu.cn (C.L.); qingtong@cau.edu.cn (Y.L.); 2 Key Laboratory of National Forestry and Grassland Administration on Pest Chemical Control, China Agricultural University, Beijing 100193, China

**Keywords:** discovery, auxinic molecule, 6-pyrazolyl-2-picolinic acid, synthesis, herbicidal activity

## Abstract

6-Aryl-2-picolinic acid herbicides are an important subclass of auxin herbicides, characterized by their good absorption and conductivity, broad weed control spectrum, and excellent herbicidal activity against some resistant weeds. Based on previous studies from our group and the distinct characteristics of physico-chemical properties and biological activities of active skeleton structure containing fluorine atoms, this paper introduces the design and synthesis of 41 novel 4-amino-6-(5-aryl-substituted-1-pyrazolyl)-3-chloro-5-fluoro-2-picolinic acid compounds. The test of inhibiting *A. thaliana* roots growth showed that most of the **S**-series compounds exhibited superior inhibitory effects compared to picloram, with six compounds demonstrated even better inhibitory capability than the new herbicidal molecule florpyrauxifen. For example, compound **S202**, at a concentration of 0.5 µmol/L, exhibited a 78.4% inhibition of *A. thaliana* root growth, whereas florpyrauxifen showed only a 33.8% inhibition. Root growth inhibition tests on weeds showed that 28 compounds, at a concentration of 250 µM, demonstrated a greater than 80% inhibition of *Brassica napus* (BN) root growth. Post-emergence herbicidal activity tests showed that most compounds exhibited good inhibitory effects on broadleaf weeds, with 10 compounds achieving a 100% inhibition of the growth of *Amaranthus retroflexus L* (AL). These results demonstrate that some of the 4-amino-6-(5-aryl-substituted-1-pyrazolyl)-3-chloro-5-fluoro-2-picolinic acid compounds could be used as potential lead structures in the discovery of novel synthetic auxin herbicides.

## 1. Introduction

To address global population growth and climate change, increasing food production is crucial for safeguarding the basic livelihood of all humanity [1,2]. As one of the three major biological threats to global food production, weed control is a significant focus in agricultural productivity. Weeds impact crop growth by competing for essential resources such as water, light, and nutrients, resulting in up to 34% crop yield losses [3,4,5]. Since the beginning of agricultural practices, humans have been attempting to ensure food production through various measures to control weed growth, among which chemical control has evolved into the most economical and effective approach [3,6]. Synthetic herbicides, as vital tools for enhancing crop yields and improving farmland management efficiency, hold an important position in modern agricultural practices [7]. However, the widespread use of most herbicides has led to increased weed resistance, and predictably, herbicides launched recently and those marketed in the near future will also face the issue of weed resistance. Therefore, it is necessary to discover and develop novel, efficient, and environmentally friendly herbicides for future food production [8,9].

Synthetic auxin herbicides, due to their complex modes of action, exhibit lower levels of resistance compared to herbicides with other modes of action [10,11]. Taking dicamba as an example, it has been used for more than 50 years in both crop and non-crop fields, and only six weed species have been found to develop resistance [12]. Therefore, plant auxin proteins have always been one of the main types of action targets of herbicides, and the discovery of new synthetic hormonal herbicides with high selectivity and low environmental risks plays a crucial role in mitigating weed resistance, as new herbicidal molecules acting on this target have been constantly emerging over the last 70 years.

**Figure 1:**
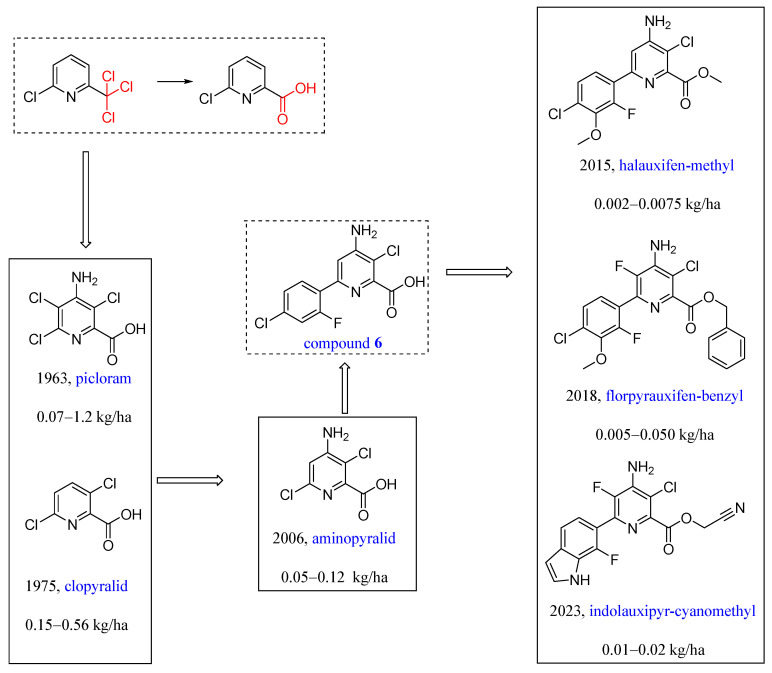
The development journey of 2-picolinic acid herbicides.

2-Picolinic acid herbicides are an important subclass of synthetic hormonal herbicides, characterized by their good absorption and conductivity, broad weed control spectrum, and herbicidal activity against resistant weeds [13,14]. Figure 1 shows that picloram (Tordon, 1963) is the first synthetic 2-picolinic acid herbicide, and it exhibits good control efficacy against perennial weeds with deep root systems. However, it can easily cause phyto-toxicity in subsequent crops, limiting its application in cereal crop fields due to its stable presence in soil and slow degradation [15]. Clopyralid (Lontrel), launched in 1975, demonstrates excellent herbicidal activity against a limited number of key dicotyledonous weeds (such as Canada thistle) and is applied in some crop fields. Aminopyralid (Milestone), commercialized in 2006, exhibits 4-fold herbicidal activity against Canada thistle compared to clopyralid, and thus serves as an alternative to clopyralid in some application scenarios [16,17]. Subsequently, Corteva Agriscience (former Dow AgroSciences) introduced a substituted phenyl group at the 6-position of aminopyralid to discover compound **6**, and after optimizing the results halauxifen-methyl (Arylex 2015) and florpyrauxifen-benzyl (Rinskor 2018) were introduced [18], which opened up a new class of 6-Arylpicolinates herbicides. In 2023, Corteva Agriscience introduced its latest herbicidal molecule indolauxipyr-cyanomethyl with the trade name Bexoveld, and expects its launch in North America in 2028 and in Europe in 2030 (Figure 1).

**Figure 2:**
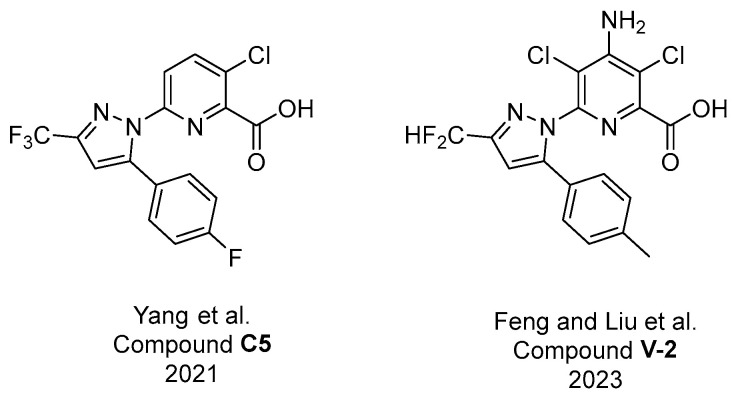
Structures of herbicidal compounds 6-phenylpyrazolyl-2-picolinic acids [19,20,21].

Our group has been focusing on design and synthesis of 6-heteroaryl-2-picolinic acids for discovering new herbicidal molecules. In 2021, Yang et al. designed and synthesized a series of novel 3-chloro-6-pyrazolyl-2-picolinic acid derivatives using clopyralid as the parent compound by replacing the chlorine atom at the 6-position of clopyralid with a substituted pyrazolyl ring, and the representative compound **C5** exhibits better herbicidal activity and a broader weed control spectrum compared to the commercial clopyralid [19]. Subsequently, Feng and Liu et al. designed and synthesized a series of novel 6-(5-aryl-substituted-1-pyrazolyl)-2-picolinic acids with picloram as a leading compound, and the IC_50_ value of the representative compound **V-2** against *A. thaliana* root growth was 62 times lower than that of picloram, and 26 times lower than that of halauxifen-methyl (Figure 2) [20,21]. These results demonstrated that 6-(5-aryl-substituted-1-pyrazolyl)-2-picolinic acid compounds could be used as potential lead structures in the discovery of novel synthetic auxin herbicidal molecules.

Based on the above studies, 41 novel 4-amino-2-chloro-5-fluoro-6-pyrazolyl-2-picolinic acid compounds were designed and synthesized using florpyrauxifen as the parent compound by introducing a phenyl-substituted pyrazolyl group at the 6-position. Their activities were evaluated by inhibiting *A. thaliana* and representative weeds’ root growth and testing post-emergence herbicidal activity in the greenhouse.

## 2. Results and Discussion

### 2.1. Chemistry

**Scheme 1:**
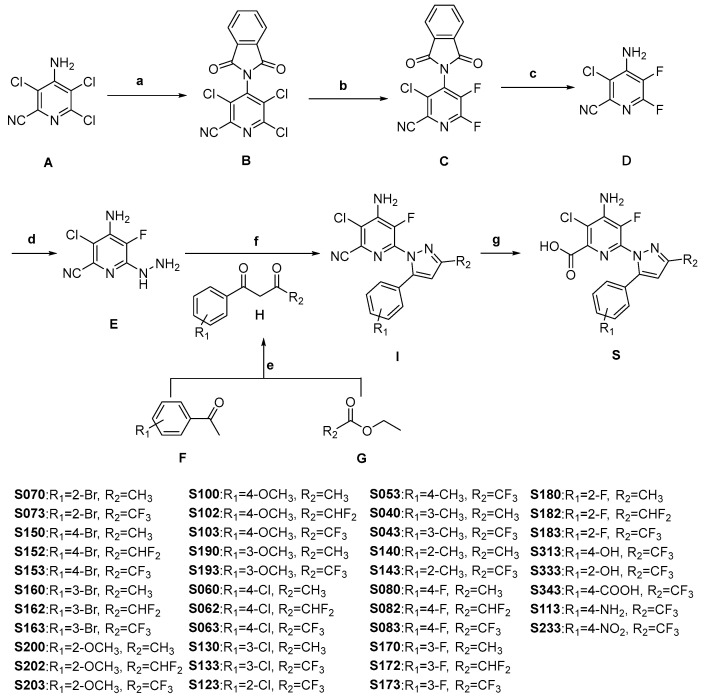
Synthesis of S-series compounds. Reagents and conditions: (**a**) DMAP, TEA, Phthaloyl dichloride, acetonitrile, −10 °C, 6 h; (**b**) CsF, DMSO, r.t., 2 h; (**c**) NH_3_·H_2_O, r.t., 7 h; (**d**) N_2_H_4_·H_2_O, THF and DMSO, 65 °C, 0.5 h; (**e**) NaH, r.t., 6 h; (**f**) sulfuric acid, ethanol, 75 °C, 2 h; (**g**) 1 M aq. KOH, 100 °C, 2–9 h.

The synthetic route is shown in Scheme 1 and is illustrated as follows. Starting from 4-amino-3,5,6-trichloro-2-picolinonitrile (**A**), the amino group protection in **A** is conducted with phthaloyl chloride in the presence of DMAP (4-dimethylaminopyridine) and TEA (triethylamine) to yield intermediate **B**, followed by fluorination with dried CsF in DMSO to obtain intermediate **C** [22]. Concentrated ammonium hydroxide is then employed to remove the protecting group, yielding intermediate **D** [23]. Intermediate **D** undergoes nucleophilic substitution with hydrazine hydrate to generate intermediate **E** [24], followed by a Knorr cyclization reaction to produce intermediate **I** [25,26] with compound **H,** which was prepared by a Claisen condensation reaction of R_1_-substituted acetophenone **F** and R_2_-substituted ethyl formate **G**. Finally, the cyano group of intermediate **I** was hydrolyzed to generate the target product **S** [27].

During the preparation of target products from the starting material **A**, all reactions were conducted according to the procedure used in the references to obtain the desired products, except for the deprotection of intermediate **C**. Hydrazine hydrate was first attempted to release the amino group from the phthalimide fragment in intermediate **C** at room temperature. TLC monitoring of the reaction progress found that the fluorine atom at the 6-position on pyridine in intermediate **D** or **C** could be hydrazinolyzed, probably forming intermediate **E** before the complete hydrazinolysis of the intermediate **C**. Upon this observation, we attempted to use hydrazine hydrate at a higher temperature to remove the protection group in the intermediate **C** while simultaneously performing nucleophilic substitution of fluorine atom at the 6-position to obtain intermediate **E** in a single step. Unfortunately, TLC monitoring revealed that dihydrazide and phthalhydrazide were also formed and the reaction resulted in a complex mixture of products with trace amounts of **E**. Then, different reagents were tested, and ammonium hydroxide was finally identified as a gentle deprotecting reagent for intermediate **C** to obtain **D**, followed by nucleophilic substitution of fluorine atom in the pyridine ring with hydrazine hydrate to yield intermediate **E**.

All the target compounds were characterized through HRMS and NMR, and their NMR spectra and data are shown in the Supplementary Materials.

### 2.2. Root Growth Inhibition Assays of A. thaliana and SAR Analysis

**Figure 3:**
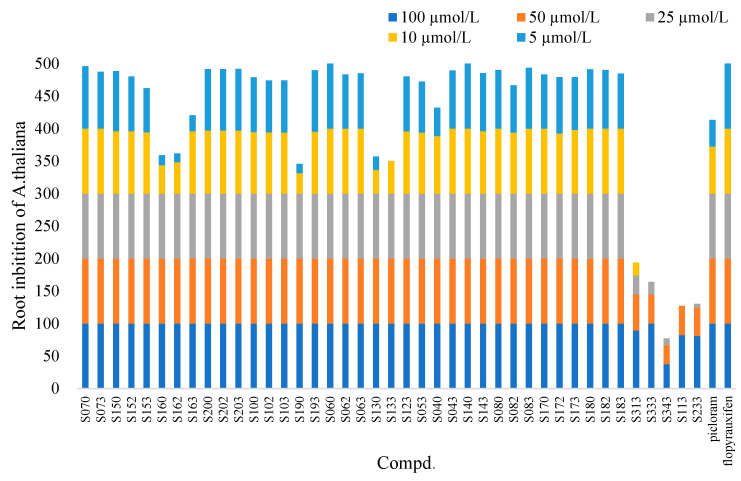
The inhibitory activity of compounds **S** against *A. thaliana* root growth.

*A. thaliana* was used as a model plant to screen the preliminary herbicidal activity of new compounds **S** by inhibiting its root growth [28]. Tests began at concentrations of 100 µmol/L, and for compounds showing potential inhibitory effects, the test concentration was further reduced. The inhibition effects of new compounds and those of commercial picloran and florpyrauxifen were taken as reference compounds; florpyrauxifen and DMSO (solvent) at different concentrations against *A. thaliana* root growth are shown in Figure 3.

**Figure 4:**
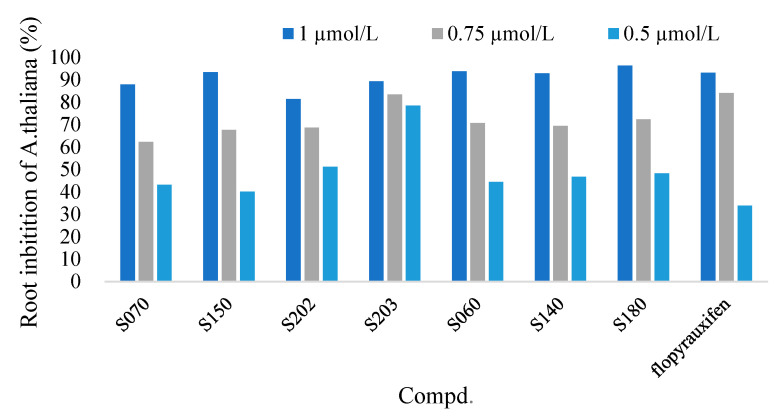
The inhibitory activity of some compounds **S** against *A. thaliana* root growth at lower concentrations.

Figure 3 shows that most of the compounds **S** exhibited excellent inhibitory effects against the root growth of *A. thaliana* at the concentrations of 100 µmol/L, 50 µmol/L, and 25 µmol/L. As the applied concentration decreased, the inhibitory activity of some compounds became more prominent. At a concentration of 0.5 µmol/L, more than 75% of the new compounds exhibited better inhibitory activity than picloram, and compounds **S070**, **S150**, **S202**, **S203**, **S060**, **S140**, and **S180** exhibited better inhibitory effects than florpyrauxifen (Figure 4).

The study of the structure–activity relationship revealed that when the R_1_ group (F, Br, Cl, methyl, or methoxy) is located at the 2 and 4 positions of the phenyl ring in the pyrazole fragment, the corresponding compounds possess superior inhibitory activity compared to those with substitutions at the 3 position of the phenyl ring. Meanwhile, both strong electron-withdrawing and electron-donating groups, such as carboxyl, nitro, and hydroxyl, amino, decrease inhibitory activity of the compounds. And the substituents R_2_ (methyl, difluoromethyl, trifluoromethyl) have less influence on inhibitory activity.

### 2.3. Root Growth Inhibition of Weeds and SAR Analysis

The herbicidal activity of new compounds **S** against the root growth of three grass seeds including *Echinochloa crusgalli* (EC), *Abutilon theophrastiMedicus* (AM), and *Brassica napus* (BN) at concentrations of 500 µM and 250 µM was evaluated according to a reported procedure [20]. Picloram and florpyrauxifen were used as the control, and each experiment had three replicates; the inhibitory effects are listed in Table 1.

Table 1 shows that most compounds had certain inhibitory effects on the root growth of weeds, with generally weak inhibitory activity observed on EC root growth. The study of the structure-activity relationship showed that the positions of substituents on phenyl fragments are related to the root growth inhibitory activity of new compounds **S**. Even in these cases, compounds **S** with R_1_ substituents on the 2 and 4 positions of the phenyl fragments exhibited a much better inhibitory effect, while the R_1_ substituents on 3 position of the phenyl fragments decreased the inhibitory activities of the corresponding compounds **S**. Similar to inhibiting *A. thaliana* root growth, strong electron-withdrawing groups and strong electron-donating groups both negatively influenced the inhibitory activity of the corresponding compounds **S.** It is noted that the compounds S exhibited excellent inhibitory activity against BN, potentially due to the fact that both BN and *A. thaliana* belong to the *Brassicaceae Burnett* family.

### 2.4. Post-Emergence Herbicidal Activity and SAR Analysis

**Figure 5:**
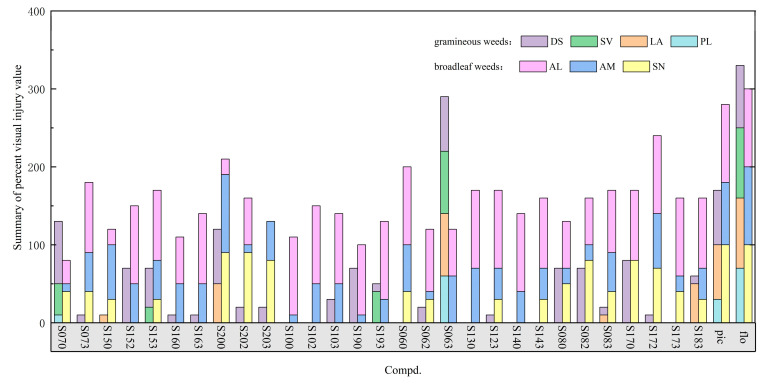
Summary of the visual injury percentages of seven weeds treated by compounds **S** over 14 days at the concentration of 500 g/ha, the first column is the accumulated herbicidal activity of each compound on gramineous weeds and the second column is that on broadleaf weeds.

In addition, the post-emergence herbicidal activities of the 41 new compounds **S** against seven common weeds were tested, including four gramineous weeds: *Poa annua* L. (PL), *Leptochloa chinensis* (LA), *Setaria viridis* (SV), and *Digitaria sanguinalis* (DS); and three broadleaf weeds: *Solanum nigrum* (SN), *Abutilon theophrastiMedicus* (AM), and *Amaranthus retroflexus* L. (AL). Two commercial herbicides, picloram and florpyrauxifen, were selected as the controls, and the results of some compounds S are shown in Figure 5.

As shown in Figure 5, the majority of compounds exhibited certain post-emergence herbicidal activity against broadleaf weeds at an application concentration of 500 g/ha, and most possessed very weak post-emergence herbicidal activity against gramineous weed species. However, it is worth noting that compound **S063** demonstrated relatively superior overall control efficacy against four gramineous weed species, outperforming picloram, and the post-emergence herbicidal activity of compounds **S** against these weed species was inferior to that of florpyrauxifen.

## 3. Materials and Methods

### 3.1. Chemicals and Instruments

All the reagents and solvents were purchased from commercial supplier Beijing InnoChem Science & Technology Co., Ltd., Beijing, China. All reactions were monitored using thin-layer chromatography (TLC) on silica gel glass plates (Qingdao Broadchem Industrial, Qingdao, China). ^1^H NMR, ^13^C NMR, and ^19^F NMR spectroscopy were recorded using a Bruker AM-500 spectrometer (Bruker, Billerica, Massachusetts, America), using DMSO-d_6_ as the solvent. HRMS were determined with an Agilent 6540 QTOF instrument (Santa Clara, California, America). The commercial herbicide picloram was provided by Nutrichem Company Ltd. (Beijing, China), and florpyrauxifen was synthesized based on methods reported by Jeffrey B. Epp [18]. The melting points of all the compounds were determined on a B-III microscope (Beijing Technical Instrument Co., Beijing, China) and were uncorrected. The *A. thaliana* and weeds root growth data were obtained using IMAGEJ 1.54i software.

### 3.2. Synthesis

#### 3.2.1. General Synthetic Procedure of Intermediate **B**

In a 1 L three-necked, round-bottom flask, compound **A** (224.77 mmol), DMAP (67.43 mmol) and TEA (674.30 mmol) were added to acetonitrile (500 mL). Then, phthaloyl dichloride (337.15 mmol) was added dropwise to the reaction mixture under stirring at −10 °C. After the addition completion, the reaction mixture was warmed to room temperature and maintained for 6 h. Once the reaction was complete, the reaction mixture was poured into water (1 L), and stirred for 0.5 h. The resulting precipitate was collected by filtration and purified by a silica gel column to obtain intermediate **B** as a white solid with 82% yield.

#### 3.2.2. General Synthetic Procedure of Intermediate **C**

In a 500 mL three-necked, round-bottom flask, CsF (297.83 mmol) were added to DMSO (300 mL), and DMSO (50 mL) was removed by vacuum distillation. Intermediate **B** was added to the reaction solution and stirred at room temperature for about 2 h. When the reaction conversion reached 85% through HPLC monitoring, the reaction mixture was poured into water (500 mL) and stirred for 0.5 h. The resulting solid was collected by filtration to obtain intermediate **C** (yield 80%).

#### 3.2.3. General Synthetic Procedure of Intermediate **D**

Intermediate **C** (82.70 mmol) was added to a solution of ammonia water (200 mL) and stirred at room temperature for 7 h. At the completion of the reaction, ethyl acetate (300 mL) was added to the reaction mixture, after phase separation, the organic layer was washed three times with a 5% aqueous NaOH and then dried with anhydrous sodium sulfate. The ethyl acetate is removed using a rotary evaporator to obtain a purple-red solid **D** with 63% yield.

#### 3.2.4. General Synthetic Procedure of Intermediate **E**

To a solution of hydrazine hydrate (158.27 mmol) in THF (15 mL), the solution of compound D (52.76 mmol) in DMSO (15 mL) was added dropwise at 65 °C and the reaction was stirred for another 0.5 h. At the completion of the reaction, the mixture was cooled to room temperature and then poured into water (200 mL). The solution was filtered to obtain a yellow solid **E** with a yield of 46%.

#### 3.2.5. General Synthetic Procedure of Intermediate **H**

Two different procedures were used to synthesize intermediate **H** in this study. When R_1_ was a methyl group, 60% sodium hydride (15.93 mmol) was slowly added into the mixture of compound **F** (14.48 mmol) in ethyl acetate (20 mL) under stirring at −5 °C. After addition, the reaction mixture was warmed to room temperature and stirred for 6 h. After the reaction was complete, the reaction was quenched with aqueous hydrochloric acid (1 N, 20 mL), and the reaction solution was acidified to a pH range of 1–2. Subsequently, the mixture was extracted three times using ethyl acetate (15 mL). The combined organic phase was dried over anhydrous sodium sulfate and concentrated under a vacuum. The residue was purified via flash column chromatography (n-hexane) to obtain intermediate **H** with 62–76% yields.

When R_1_ was a difluoromethyl or trifluoromethyl, compound **F** (14.48 mmol) and compound **G** (17.37 mmol) were added to carbon tetrachloride (20 mL) under stirring at −5 °C, and then 60% sodium hydride (15.93 mmol) was slowly added into the mixture. After addition, the reaction solution was warmed to room temperature and stirred for 6 h. The subsequent workup was identical to the method used when R_1_ was a methyl group, resulting in the final intermediate **H** with yields of 84–91%.

#### 3.2.6. General Synthetic Procedure of Intermediate **I**

Intermediate **E** (4.96 mmol) was added to a solution of intermediate **H** (4.96 mmol) in ethanol (20 mL) at room temperature and was stirred for about 15 min. Concentrated sulfuric acid (98%, 2 drops) was added, and the reaction mixture was heated to 75 °C and maintained for 2 h. When the reaction was completed, the reaction mixture was cooled to room temperature and treated with a saturated sodium carbonate solution. Then, the mixture was extracted three times using ethyl acetate (15 mL), the combined organic phases were dried over anhydrous sodium sulfate and concentrated under vacuum. The residue was purified via flash column chromatography (n-hexane/ethyl acetate = 6:1) to afford intermediate **I** with 76–94% yields.

#### 3.2.7. General Synthetic Procedure of Compounds **S**

Intermediate **I** (3.97 mmol) was dissolved in KOH aqueous (1M, 10 mL) in a 25 mL round-bottom flask, and was heated to 100 °C and maintained for 2–9 h. Then, the reaction mixture was cooled to room temperature and acidified to a pH range of 1–2. The white solid was collected through filtration and dried to obtain the target compound **S** with yields of 88–95%. The data of ^1^H NMR, ^19^F NMR, ^13^C NMR, and HRMS for compounds **S** are listed below.

Compound **S070** 4-Amino-6-(5-(2-bromophenyl)-3-methyl-1*H*-pyrazol-1-yl)-3-chloro-5-fluoro-2-picolinic acid, a white solid with melting point of 186–188 °C; ^1^H NMR (500.13 MHz, DMSO-d_6_) δ 13.46 (s, 1H), 7.63 (dd, *J* = 7.9, 1.2 Hz, 1H), 7.36 (td, *J* = 7.5, 1.2 Hz, 1H), 7.28 (td, *J* = 7.7, 1.8 Hz, 1H), 7.24 (dd, *J* = 7.6, 1.7 Hz, 1H), 7.04 (s, 2H), 2.31 (s, 3H); ^13^C NMR (125.77 MHz, DMSO- d_6_) δ 165.66, 149.67, 144.17 (d, ^3^*J_F-C_* = 4.6 Hz), 143.48, 143.19 (d, ^2^*J_F-C_* = 12.5 Hz), 140.41 (d, ^1^*J_F-C_* = 260.5 Hz), 136.97 (d, ^2^*J_F-C_* = 8.0 Hz), 133.21, 132.24, 131.56, 131.04, 127.93, 122.88, 112.45, 109.47, 13.80; ^19^F NMR (470.54 MHz, DMSO- d_6_) δ −143.30; HRMS for C_16_H_11_BrClFN_4_O_2_ ([M + H]^+^) calcd. 424.9816 and found 424.9821.

Compound **S073** 4-Amino-6-(5-(2-bromophenyl)-3-(trifluoromethyl)-1*H*-pyrazol-1-yl)-3-chloro-5-fluoro-2-picolinic acid, a white solid with melting point of 182–185 °C; ^1^H NMR (500.13 MHz, DMSO-d_6_) δ 13.69 (s, 1H), 7.70 (dd, *J* = 7.9, 1.3 Hz, 1H), 7.42 (td, *J* = 7.4, 1.3 Hz, 1H), 7.40–7.34 (m, 2H), 7.26 (s, 2H), 7.25 (s, 1H); ^13^C NMR (125.77 MHz, DMSO-d_6_) δ 165.40, 145.15, 144.61 (d, ^3^*J_F-C_* = 4.5 Hz), 143.56 (d, ^2^*J_F-C_* = 12.5 Hz), 142.75 (q, ^2^*J_F-C_* = 38.4 Hz), 140.62 (d, ^1^*J_F-C_* = 261.2 Hz), 135.67 (d, ^2^*J_F-C_* = 8.2 Hz), 133.31, 132.64, 132.09, 129.63, 128.16, 123.10, 121.54 (q, ^1^*J_F-C_* = 269.0 Hz), 113.64, 107.66; ^19^F NMR 470.54 MHz, DMSO-d_6_) δ −60.89, −143.36; HRMS for C_16_H_8_BrClF_4_N_4_O_2_ ([M + Na]^+^) calcd. 500.9353 and found 500.9358.

Compound **S150** 4-Amino-6-(5-(4-bromophenyl)-3-methyl-1*H*-pyrazol-1-yl)-3-chloro-5-fluoro-2-picolinic acid, a white solid with melting point of 196 °C; ^1^H NMR (500.13 MHz, DMSO-d_6_) δ 13.70 (s, 1H), 7.60–7.55 (m, 2H), 7.21–7.16 (m, 2H), 7.16 (s, 2H), 6.59 (s, 1H); ^13^C NMR (125.77 MHz, DMSO-d_6_) δ 165.75, 150.31, 144.83 (d, ^3^*J_F-C_* = 4.6 Hz), 144.14, 143.39 (d, ^2^*J_F-C_* = 12.5 Hz), 141.17 (d, ^1^*J_F-C_* = 258.8 Hz), 137.24 (d, ^2^*J_F-C_* = 9.7 Hz), 132.21, 129.64, 129.06, 122.33, 113.28, 107.68, 13.71; ^19^F NMR (470.54 MHz, DMSO-d_6_) δ −143.81; HRMS for C_16_H_11_BrClFN_4_O_2_ ([M + H]^+^) calcd. 424.9816 and found 424.9819.

Compound **S152** 4-Amino-6-(5-(4-bromophenyl)-3-(difluoromethyl)-1*H*-pyrazol-1-yl)-3-chloro-5-fluoro-2-picolinic acid, a white solid with melting point of 198–201 °C; ^1^H NMR (500.13 MHz, DMSO-d_6_) δ 13.83 (s, 1H), 7.62 (d, *J* = 8.5 Hz, 2H), 7.30 (s, 2H), 7.26 (d, *J* = 8.5 Hz, 2H), 7.15 (t, ^1^*J_F-H_* = 41.4 Hz, 1H), 7.12 (s, 1H); ^13^C NMR (125.77 MHz, DMSO-d_6_) δ 165.56, 148.32 (t, ^2^*J_F-C_* = 28.9 Hz), 145.21, 145.06 (d, ^3^*J_F-C_* = 4.5 Hz), 143.66 (d, ^2^*J_F-C_* = 12.4 Hz), 141.19 (d, ^1^*J_F-C_* = 260.0 Hz), 136.34 (d, ^2^*J_F-C_* = 9.2 Hz), 132.40, 130.06, 127.91, 123.22, 114.08, 111.57 (t, ^1^*J_F-C_* = 233.0 Hz), 105.04; ^19^F NMR (470.54 MHz, DMSO-d_6_) δ −112.32, −143.98; HRMS for C_16_H_9_BrClF_3_N_4_O_2_([M + Na]^+^) calcd.482.9447 and found 482.9448.

Compound **S153** 4-Amino-6-(5-(4-bromophenyl)-3-(trifluoromethyl)-1*H*-pyrazol-1-yl)-3-chloro-5-fluoro-2-picolinic acid, a white solid with melting point of 205 °C; ^1^H NMR (500.13 MHz, DMSO-d_6_) δ 13.89 (s, 1H), 7.64 (d, *J* = 8.5 Hz, 2H), 7.38 (s, 1H), 7.34 (s, 2H), 7.29 (d, *J* = 8.5 Hz, 1H); ^13^C NMR (125.77 MHz, DMSO-d_6_) δ 165.50, 145.74, 145.21 (d, ^3^*J_F-C_* = 4.3 Hz), 143.77 (d, ^2^*J_F-C_* = 12.3 Hz), 143.36 (q, ^2^*J_F-C_* = 37.6 Hz), 141.18 (d, ^1^*J_F-C_* = 260.1 Hz), 135.90 (d, ^2^*J_F-C_* = 9.3 Hz), 132.48, 130.21, 127.29, 123.64, 121.51 (q, ^1^*J_F-C_* = 269.0 Hz), 114.37, 105.86; ^19^F NMR (470.54 MHz, DMSO-d_6_) δ −61.12, −144.03; HRMS for C_16_H_8_BrClF_4_N_4_O_2_([M + Na]^+^) calcd. 500.9353 and found 500.9352.

Compound **S160** 4-Amino-6-(5-(3-bromophenyl)-3-methyl-1*H*-pyrazol-1-yl)-3-chloro-5-fluoro-2-picolinic acid, a white solid with melting point of 176–182 °C; ^1^H NMR (500.13 MHz, DMSO-d_6_) δ 13.73 (s, 1H), 7.58–7.49 (m, 2H), 7.31 (t, *J* = 7.9 Hz, 1H), 7.23–7.10 (m, 3H), 6.65 (s, 1H), 2.28 (s, 3H); ^13^C NMR (125.77 MHz, DMSO-d_6_) δ 165.74, 150.29, 144.85 (d, ^3^*J_F-C_* = 4.7 Hz), 143.64, 143.38 (d, ^2^*J_F-C_* = 12.5 Hz), 141.22 (d, ^1^*J_F-C_* = 258.8 Hz), 137.17 (d, ^2^*J_F-C_* = 9.2 Hz), 132.01, 131.70, 131.28, 130.39, 126.56, 122.36, 113.26, 108.02, 13.73; ^19^F NMR (470.54 MHz, DMSO-d_6_) δ −143.81; HRMS for C_16_H_11_BrClFN_4_O_2_([M + H]^+^) calcd. 424.9816 and found 424.9819.

Compound **S162** 4-Amino-6-(5-(3-bromophenyl)-3-(difluoromethyl)-1*H*-pyrazol-1-yl)-3-chloro-5-fluoro-2-picolinic acid, a white solid with melting point of 139–171 °C; ^1^H NMR (500.13 MHz, DMSO-d_6_) δ 13.83 (s, 1H), 7.64 (t, *J* = 1.9 Hz, 1H), 7.63–7.59 (m, 1H), 7.35 (t, *J* = 7.9 Hz, 1H), 7.31 (s, 2H), 7.21 (dt, *J* = 7.8, 1.3 Hz, 1H), 7.19 (s, 1H), 7.15 (t, ^1^*J_F-H_* = 54.3 Hz, 1H); ^13^C NMR (125.77 MHz, DMSO-d_6_) δ 165.54, 148.29 (t, ^2^*J_F-C_* = 28.8 Hz), 145.05 (d, ^3^*J_F-C_* = 4.5 Hz), 144.70, 143.65 (d, ^2^*J_F-C_* = 12.4 Hz), 141.24 (d, ^1^*J_F-C_* = 259.9 Hz), 136.29 (d, ^2^*J_F-C_* = 10.0 Hz), 132.48, 131.41, 130.88, 130.83, 126.94, 122.50, 114.03, 111.55 (t, ^1^*J_F-C_* = 233.1 Hz), 105.42; ^19^F NMR (470.54 MHz, DMSO-d_6_) δ −112.36, −143.94; HRMS for C_16_H_9_BrClF_3_N_4_O_2_([M + Na]^+^) calcd. 482.9447 and found 482.9448.

Compound **S163** 4-Amino-6-(5-(3-bromophenyl)-3-(trifluoromethyl)-1*H*-pyrazol-1-yl)-3-chloro-5-fluoro-2-picolinic acid, a white solid with melting point of 113–152 °C; ^1^H NMR (500.13 MHz, DMSO-d_6_) δ 13.73 (d, *J* = 136.4 Hz, 1H), 7.68 (t, *J* = 1.8 Hz, 1H), 7.63 (dd, *J* = 7.9, 1.9 Hz, 1H), 7.45 (s, 1H), 7.40–7.31 (m, 3H), 7.23 (dt, *J* = 7.8, 1.3 Hz, 1H); ^13^C NMR (125.77 MHz, DMSO-d_6_) δ 165.46, 145.21, 145.12 (d, ^3^*J_F-C_* = 4.5 Hz), 143.77 (d, ^2^*J_F-C_* = 11.6 Hz), 143.33 (q, ^2^*J_F-C_* = 37.5 Hz), 141.23 (d, ^1^*J_F-C_* = 260.8 Hz), 135.85 (d, ^2^*J_F-C_* = 9.1 Hz), 132.84, 131.48, 131.09, 130.19, 127.04, 122.56, 121.66 (q, ^1^*J_F-C_* = 269.1 Hz), 114.37 (d, ^4^*J_F-C_* = 2.3 Hz), 106.26; ^19^F NMR (470.54 MHz, DMSO-d_6_) δ 61.12, −143.93; HRMS for C_16_H_8_BrClF_4_N_4_O_2_([M + Na]^+^) calcd. 500.9353 and found 500.9357.

Compound **S200** 4-Amino-3-chloro-5-fluoro-6-(5-(2-methoxyphenyl)-3-methyl-1*H*-pyrazol-1-yl)-2-picolinic acid, a white solid with melting point of 189 °C; ^1^H NMR (500.13 MHz, DMSO-d_6_) δ 13.52 (s, 1H), 7.31 (td, *J* = 8.2, 1.7 Hz, 1H), 7.20 (dd, *J* = 7.7, 1.7 Hz, 1H), 6.97 (s, 2H), 6.96–6.91 (m, 2H), 6.38 (s, 1H), 3.43 (s, 3H), 2.27 (s, 3H); ^13^C NMR (125.77 MHz, DMSO-d_6_) δ 165.92, 156.11, 149.47, 144.28 (d, ^3^*J_F-C_* = 4.6 Hz), 142.96 (d, ^2^*J_F-C_* = 12.6 Hz), 142.32, 140.51 (d, ^1^*J_F-C_* = 258.5 Hz), 138.32 (d, ^2^*J_F-C_* = 9.1 Hz), 130.73, 130.61, 120.92, 119.21, 112.14 (d, ^4^*J_F-C_* = 1.4 Hz), 111.95, 108.60, 55.27, 13.76; ^19^F NMR (470.54 MHz, DMSO-d_6_) δ −144.21; HRMS For C_17_H_14_ClFN_4_O_3_([M + H]^+^) calcd. 377.0817 and found 377.0822.

Compound **S202** 4-Amino-3-chloro-6-(3-(difluoromethyl)-5-(2-methoxyphenyl)-1*H*-pyrazol-1-yl)-5-fluoro-2-picolinic acid, a white solid with melting point of 167–173 °C; ^1^H NMR (500.13 MHz, DMSO-d_6_) δ 13.66 (s, 1H), 7.38 (td, *J* = 7.9, 1.7 Hz, 1H), 7.29 (dd, *J* = 7.8, 1.7 Hz, 1H), 7.15 (s, 2H), 7.13 (t, ^1^*J_F-H_* = 54.5 Hz, 1H), 7.01–6.97 (m, 2H), 6.87 (s, 1H); ^13^C NMR (125.77 MHz, DMSO-d_6_) δ 165.75, 156.18, 147.67 (t, ^2^*J_F-C_* = 28.8 Hz), 144.57 (d, ^3^*J_F-C_* = 5.3 Hz), 143.53, 143.23 (d, ^2^*J_F-C_* = 12.5 Hz), 140.57 (d, ^1^*J_F-C_* = 259.4 Hz), 137.43 (d, ^2^*J_F-C_* = 9.2 Hz), 131.56, 130.98, 121.10, 117.89, 112.94, 112.01, 111.77 (t, ^1^*J_F-C_* = 232.5 Hz), 105.86, 55.36; ^19^F NMR (470.54 MHz, DMSO-d_6_) δ −111.85, −144.38; HRMS for C_17_H_12_ClF_3_N_4_O_3_([M + Na]^+^) calcd. 435.0448 and found 435.0452.

Compound **S203** 4-Amino-3-chloro-5-fluoro-6-(5-(2-methoxyphenyl)-3-(trifluoromethyl)-1*H*-pyrazol-1-yl)-2-picolinic acid, a white solid with melting point of 177–178 °C; ^1^H NMR (500.13 MHz, DMSO-d_6_) δ 13.74 (s, 1H), 7.40 (dd, *J* = 7.9, 1.7 Hz, 1H), 7.32 (dd, *J* = 7.7, 1.7 Hz, 1H), 7.20 (s, 2H), 7.12 (s, 1H), 7.03–6.97 (m, 2H), 3.46 (s, 3H); ^13^C NMR (125.77 MHz, DMSO-d_6_) δ 165.67, 156.21, 144.69 (d, ^3^*J_F-C_* = 4.5 Hz), 144.10, 143.34 (d, ^2^*J_F-C_* = 12.4 Hz), 142.74 (q, ^2^*J_F-C_* = 37.4 Hz), 140.57 (d, ^1^*J_F-C_* = 260.7 Hz), 137.02 (d, ^2^*J_F-C_* = 9.2 Hz), 131.93, 131.12, 121.70 (q, ^1^*J_F-C_* = 268.9 Hz), 121.15, 117.21, 113.27, 112.05, 106.66, 55.41; ^19^F NMR (470.54 MHz, DMSO-d_6_) δ −60.90, −144.41; HRMS for C_17_H_11_ClF_4_N_4_O_3_([M + Na]^+^) calcd. 453.0354 and found 453.0357.

Compound **S100** 4-Amino-3-chloro-5-fluoro-6-(5-(4-methoxyphenyl)-3-methyl-1*H*-pyrazol-1-yl)-2-picolinic acid, a white solid with a melting point 173–182 °C; ^1^H NMR (500.13 MHz, DMSO-d_6_) δ 7.21–7.10 (m, 4H), 6.95–6.86 (m, 2H), 6.46 (s, 1H), 3.74 (s, 3H), 2.26 (s, 3H); ^13^C NMR (125.77 MHz, DMSO-d_6_) δ 165.84, 159.78, 150.00, 145.23, 144.90 (d, ^3^*J_F-C_* = 5.3 Hz), 143.24 (d, ^2^*J_F-C_* = 12.5 Hz), 141.45 (d, ^1^*J_F-C_* = 258.1 Hz), 137.66 (d, ^2^*J_F-C_* = 10.0 Hz), 128.99, 122.18, 114.67, 113.23, 106.47, 55.61, 13.74; ^19^F NMR (470.54 MHz, DMSO-d_6_) δ −143.60; HRMS for C_17_H_14_ClFN_4_O_3_([M + H]^+^) calcd. 377.0817 and found 377.0822.

Compound **S102** 4-Amino-3-chloro-6-(3-(difluoromethyl)-5-(4-methoxyphenyl)-1*H*-pyrazol-1-yl)-5-fluoro-2-picolinic acid, a white solid with melting point of 183–186 °C; ^1^H NMR (500.13 MHz, DMSO-d_6_) δ 13.87 (s, 1H), 7.27 (s, 2H), 7.24 (d, *J* = 8.8 Hz, 2H), 7.06 (t, ^1^*J_F-H_* = 54.3 Hz, 1H), 6.99–6.94 (m, 3H), 3.97–3.48 (m, 6H); ^13^C NMR (125.77 MHz, DMSO-d_6_) δ 165.66, 160.31, 148.13 (t, ^2^*J_F-C_* = 28.9 Hz), 146.29, 145.13 (d, ^3^*J_F-C_* = 4.5 Hz), 143.52 (d, ^2^*J_F-C_* = 12.5 Hz), 141.43 (d, ^1^*J_F-C_* = 259.2 Hz), 136.75 (d, ^2^*J_F-C_* = 9.4 Hz), 129.42, 120.95, 114.86, 113.99, 111.72 (t, ^1^*J_F-C_* = 232.7 Hz), 103.77, 55.70; ^19^F NMR (470.54 MHz, DMSO-d_6_) δ −112.11, −143.84; HRMS for C_17_H_12_ClF_3_N_4_O_3_ ([M + Na]^+^) calcd. 435.0448 and found 435.0448.

Compound **S103** 4-Aamino-3-chloro-5-fluoro-6-(5-(4-methoxyphenyl)-3-(trifluoromethyl)-1*H*-pyrazol-1-yl)-2-picolinic acid, a white solid with melting point of 196–197 °C; ^1^H NMR (500.13 MHz, DMSO-d_6_) δ 13.94 (s, 1H), 7.33 (s, 2H), 7.28–7.25 (m, 2H), 7.23 (s, 1H), 7.01–6.95 (m, 2H), 3.76 (s, 3H); ^13^C NMR (125.77 MHz, DMSO-d_6_) δ 165.57, 160.56, 146.82, 145.23 (d, ^3^*J_F-C_* = 4.5 Hz), 143.63 (d, ^2^*J_F-C_* = 12.4 Hz), 143.19 (q, ^2^*J_F-C_* = 37.8 Hz), 141.41 (d, ^1^*J_F-C_* = 260.0 Hz), 136.31 (d, ^2^*J_F-C_* = 10.1 Hz), 129.59, 121.64 (q, ^1^*J_F-C_* = 268.9 Hz), 120.28, 114.94, 114.32, 104.61, 55.74; ^19^F NMR (470.54 MHz, DMSO-d_6_) δ −61.08, −143.90; HRMS for C_17_H_11_ClF_4_N_4_O_3_ ([M + Na]^+^) calcd. 453.0354 and found 453.0356.

Compound **S190** 4-Amino-3-chloro-5-fluoro-6-(5-(3-methoxyphenyl)-3-methyl-1*H*-pyrazol-1-yl)-2-picolinic acid, a white solid with melting point of 186–188 °C; ^1^H NMR (500.13 MHz, DMSO-d_6_) δ 13.79 (s, 1H), 7.27 (t, *J* = 8.2 Hz, 1H), 7.15 (s, 2H), 6.92–6.87 (m, 1H), 6.78 (d, *J* = 1.4 Hz, 2H), 6.58 (s, 1H), 3.67 (s, 3H), 2.27 (s, 3H); ^13^C NMR (125.77 MHz, DMSO-d_6_) δ 165.86, 159.62, 150.06, 145.17, 145.01 (d, ^3^*J_F-C_* = 4.6 Hz), 143.23 (d, ^2^*J_F-C_* = 13.4 Hz), 141.56 (d, ^1^*J_F-C_* = 258.3 Hz), 137.59 (d, ^2^*J_F-C_* = 10.1 Hz), 130.96, 130.41, 119.91, 114.80, 113.20 (d, ^4^*J_F-C_* = 1.9 Hz), 112.90, 107.24, 55.42, 13.73; ^19^F NMR (470.54 MHz, DMSO-d_6_) δ −143.72; HRMS for C_17_H_14_ClFN_4_O_3_ ([M + H]^+^) calcd. 377.0817 and found 377.0820.

Compound **S193** 4-Amino-3-chloro-5-fluoro-6-(5-(3-methoxyphenyl)-3-(trifluoromethyl)-1*H*-pyrazol-1-yl)-2-picolinic acid, a white solid with melting point of 172 °C; ^1^H NMR (500.13 MHz, DMSO-d_6_) δ 13.97 (s, 1H), 7.37 (s, 1H), 7.36 (s, 2H), 7.33 (t, *J* = 8.0 Hz, 1H), 6.99 (dd, *J* = 8.2, 2.6 Hz, 1H), 6.92 (t, *J* = 2.1 Hz, 1H), 6.86 (dt, *J* = 7.8, 1.1 Hz, 1H); ^13^C NMR (125.77 MHz, DMSO-d_6_) δ 165.60, 159.76, 146.75, 145.32 (d, ^3^*J_F-C_* = 4.5 Hz), 143.62 (d, ^2^*J_F-C_* = 12.5 Hz), 143.24 (q, ^2^*J_F-C_* = 37.5 Hz), 141.50 (d, ^1^*J_F-C_* = 260.0 Hz), 136.23 (d, ^2^*J_F-C_* = 10.1 Hz), 130.68, 129.16, 121.58 (q, ^1^*J_F-C_* = 268.9 Hz) 120.28, 116.10, 114.28 (d, ^4^*J_F-C_* = 2.3 Hz), 113.34, 105.49, 55.56; ^19^F NMR (470.54 MHz, DMSO-d_6_) δ −61.08, −143.97; HRMS for C_17_H_11_ClF_4_N_4_O_3_([M + Na]^+^) calcd. 453.0354 and found 453.0357.

Compound **S060** 4-Amino-3-chloro-6-(5-(4-chlorophenyl)-3-methyl-1*H*-pyrazol-1-yl)-5-fluoro-2-picolinic acid, a white solid with melting point of 198–204 °C; ^1^H NMR (500.13 MHz, DMSO-d_6_) δ 13.51 (s, 1H), 7.44 (d, *J* = 8.6 Hz, 2H), 7.24 (d, *J* = 8.6 Hz, 2H), 7.15 (s, 2H), 6.59 (s, 1H), 2.28 (s, 3H); ^13^C NMR (125.77 MHz, DMSO-d_6_) δ 165.74, 150.28, 144.84 (d, ^3^*J_F-C_* = 5.3 Hz), 144.09, 143.39 (d, ^2^*J_F-C_* = 13.1 Hz), 141.18 (d, ^1^*J_F-C_* = 258.5 Hz), 137.25 (d, ^2^*J_F-C_* = 9.7 Hz), 133.67, 129.29, 128.71, 107.69, 13.71; ^19^F NMR (470.54 MHz, DMSO-d_6_) δ −143.81; HRMS for C_16_H_11_Cl_2_FN_4_O_2_([M + H]^+^) calcd. 381.0321 and found 381.0326.

Compound **S062** 4-Amino-3-chloro-6-(5-(4-chlorophenyl)-3-(difluoromethyl)-1*H*-pyrazol-1-yl)-5-fluoro-2-picolinic acid, a white solid with melting point of 198–204 °C; ^1^H NMR (500.13 MHz, DMSO-d_6_) δ 13.82 (s, 1H), 7.49 (d, *J* = 8.6 Hz, 2H), 7.33 (d, *J* = 8.6 Hz, 2H), 7.29 (s, 2H), 7.15 (t, ^1^*J_F-H_* = 54.3 Hz, 1H), 7.12 (s, 1H); ^13^C NMR (125.77 MHz, DMSO-d_6_) δ 165.57, 148.30 (t, ^2^*J_F-C_* = 28.8 Hz), 145.15, 145.06 (d, ^3^*J_F-C_* = 4.5 Hz), 143.66 (d, ^2^*J_F-C_* = 12.4 Hz), 141.20 (d, ^1^*J_F-C_* = 259.9 Hz), 136.34 (d, ^2^*J_F-C_* = 10.0 Hz), 134.51, 129.84, 129.48, 127.56, 114.05, 111.58 (t, ^1^*J_F-C_* = 233.2 Hz), 105.05; ^19^F NMR (470.54 MHz, DMSO-d_6_) δ −112.31, −143.98; HRMS for C_16_H_9_Cl_2_F_3_N_4_O_2_([M + H]^+^) calcd. 417.0133 and found 417.0132.

Compound **S063** 4-Amino-3-chloro-6-(5-(4-chlorophenyl)-3-(trifluoromethyl)-1*H*-pyrazol-1-yl)-5-fluoro-2-picolinic acid, a white solid with melting point of 202–204 °C; ^1^H NMR (500.13 MHz, DMSO-d_6_) δ 13.88 (s, 1H), 7.51 (d, *J* = 8.6 Hz, 2H), 7.37 (d, *J* = 5.1 Hz, 2H), 7.35 (m, 3H); ^13^C NMR (125.77 MHz, DMSO-d_6_) δ 165.48, 145.68, 145.12 (d, ^3^*J_F-C_* = 4.5 Hz), 143.78 (d, ^2^*J_F-C_* = 12.3 Hz), 143.35 (q, ^2^*J_F-C_* = 37.7 Hz), 141.21 (d, ^1^*J_F-C_* = 260.3 Hz), 135.91 (d, ^2^*J_F-C_* = 9.8 Hz), 134.90, 130.01, 129.55, 126.95, 121.52 (q, ^1^*J_F-C_* = 268.9 Hz), 114.40, 105.88; ^19^F NMR (470.54 MHz, DMSO-d_6_) δ −61.13, −143.98; HRMS for C_16_H_8_Cl_2_F_4_N_4_O_2_([M + Na]^+^) calcd. 456.9858 and found 456.9868.

Compound **S130** 4-Amino-3-chloro-6-(5-(3-chlorophenyl)-3-methyl-1*H*-pyrazol-1-yl)-5-fluoro-2-picolinic acid, a white solid with melting point of 178–181 °C; ^1^H NMR (500.13 MHz, DMSO-d_6_) δ 7.44–7.33 (m, 3H), 7.20 (s, 2H), 7.10 (dt, *J* = 7.3, 1.6 Hz, 1H), 6.66 (s, 1H), 2.28 (s, 3H); ^13^C NMR (125.77 MHz, DMSO-d_6_) δ 165.73, 150.30, 144.87 (d, ^3^*J_F-C_* = 4.6 Hz), 143.76, 143.38 (d, ^2^*J_F-C_* = 12.5 Hz), 141.22 (d, ^1^*J_F-C_* = 258.8 Hz), 137.18 (d, ^2^*J_F-C_* = 9.1 Hz), 133.83, 131.79, 131.05, 128.81, 127.55, 126.22, 113.26, 108.03, 13.72; ^19^F NMR (470.54 MHz, DMSO-d_6_) δ −143.78; HRMS for C_16_H_11_Cl_2_FN_4_O_2_([M + H]^+^) calcd. 381.0321 and found 381.0325.

Compound **S133** 4-Amino-3-chloro-6-(5-(3-chlorophenyl)-3-(trifluoromethyl)-1*H*-pyrazol-1-yl)-5-fluoro-2-picolinic acid, a white solid with melting point of 144–168 °C; ^1^H NMR (500.13 MHz, DMSO-d_6_) δ 13.92 (s, 1H), 7.56 (d, *J* = 1.9 Hz, 1H), 7.51 (dd, *J* = 8.1, 1.9 Hz, 1H), 7.47–7.41 (m, 2H), 7.38 (s, 2H), 7.21–7.15 (m, 1H); ^13^C NMR (125.77 MHz, DMSO-d_6_) δ 165.49, 145.31, 145.14 (d, ^3^*J_F-C_* = 4.4 Hz), 143.77 (d, ^2^*J_F-C_* = 12.3 Hz), 143.33 (q, ^2^*J_F-C_* = 37.5 Hz), 141.21 (d, ^1^*J_F-C_* = 260.7 Hz), 135.85 (d, ^2^*J_F-C_* = 9.9 Hz), 134.08, 131.28, 129.99, 129.96, 128.29, 126.69, 121.49 (q, ^1^*J_F-C_* = 269.1 Hz), 114.33 (d, ^4^*J_F-C_* = 2.3 Hz), 106.28; ^19^F NMR (470.54 MHz, DMSO-d_6_) δ −61.12, −143.95; HRMS For C_16_H_8_Cl_2_F_4_N_4_O_2_ ([M + Na]^+^) calcd. 456.9858 and found 456.9854.

Compound **S123** 4-Amino-3-chloro-6-(5-(2-chlorophenyl)-3-(trifluoromethyl)-1*H*-pyrazol-1-yl)-5-fluoro-2-picolinic acid, a white solid with melting point of 162–168 °C; ^1^H NMR (500.13 MHz, DMSO-d_6_) δ 13.70 (s, 1H), 7.54 (d, *J* = 7.8 Hz, 1H), 7.49–7.43 (m, 1H), 7.42–7.35 (m, 2H), 7.27 (s, 1H), 7.26 (s, 2H); ^13^C NMR (125.77 MHz, DMSO-d_6_) δ 165.42, 144.70 (d, ^3^*J_F-C_* = 4.5 Hz), 143.62, 143.51, 142.91 (q, ^2^*J_F-C_* = 38.2 Hz), 140.60 (d, ^1^*J_F-C_* = 261.1 Hz), 135.77 (d, ^2^*J_F-C_* = 8.9 Hz), 132.76, 132.44, 131.99, 130.20, 127.78, 127.58, 121.53 (q, ^1^*J_F-C_* = 269.0 Hz), 113.69, 107.75; ^19^F NMR (470.54 MHz, DMSO-d_6_) δ −60.94, −143.82; HRMS for C_16_H_8_Cl_2_F_4_N_4_O_2_ ([M + Na]^+^) calcd. 456.9858 and found 456.9862.

Compound **S053** 4-Amino-3-chloro-5-fluoro-6-(5-(p-tolyl)-3-(trifluoromethyl)-1*H*-pyrazol-1-yl)-2-picolinic acid, a white solid with melting point of 189–193 °C; ^1^H NMR (500.13 MHz, DMSO-d_6_) δ 13.93 (s, 1H), 7.33 (s, 2H), 7.28 (s, 1H), 7.25–7.19 (m, 4H), 2.30 (s, 3H); ^13^C NMR (125.77 MHz, DMSO-d_6_) δ 164.52, 145.96, 144.17 (d, ^3^*J_F-C_* = 4.5 Hz), 142.57 (d, ^2^*J_F-C_* = 12.4 Hz), 142.21 (q, ^2^*J_F-C_* = 38.2 Hz), 140.36 (d, ^1^*J_F-C_* = 260.1 Hz), 138.79, 135.22 (d, ^2^*J_F-C_* = 9.2 Hz), 124.16, 120.57 (q, ^1^*J_F-C_* = 269.0 Hz), 113.28 (d, ^4^*J_F-C_* = 2.4 Hz), 103.97, 20.19; ^19^F NMR (470.54 MHz, DMSO-d_6_) δ −61.09, −143.95; HRMS for C_17_H_11_ClF_4_N_4_O_2_([M + Na]^+^) calcd. 437.0404 and found 437.0408.

Compound **S040** 4-Amino-3-chloro-5-fluoro-6-(3-methyl-5-(m-tolyl)-1*H*-pyrazol-1-yl)-2-picolinic acid, a white solid with melting point of 107 °C; ^1^H NMR (500.13 MHz, DMSO-d_6_) δ 7.24–7.07 (m, 5H), 6.91 (d, *J* = 7.7 Hz, 1H), 6.52 (s, 1H), 2.27 (s, 3H), 2.26 (s, 3H); ^13^C NMR (125.77 MHz, DMSO-d_6_) δ 165.84, 150.03, 145.48, 144.87 (d, ^3^*J_F-C_* = 5.3 Hz), 143.21 (d, ^2^*J_F-C_* = 12.7 Hz), 141.45 (d, ^1^*J_F-C_* = 258.7 Hz), 138.41, 137.60 (d, ^2^*J_F-C_* = 10.0 Hz), 129.71, 129.60, 129.00, 128.57, 124.58, 113.14, 107.10, 21.39, 13.74; ^19^F NMR (470.54 MHz, DMSO-d_6_) δ −143.67; HRMS for C_17_H_14_ClFN_4_O_2_([M + H]^+^) calcd. 361.0868 and found 361.0872.

Compound **S043** 4-Amino-3-chloro-5-fluoro-6-(5-(m-tolyl)-3-(trifluoromethyl)-1*H*-pyrazol-1-yl)-2-picolinic acid, a white solid with melting point of 129–159 °C; ^1^H NMR (500.13 MHz, DMSO-d_6_) δ 13.93 (s, 1H), 7.32 (s, 2H), 7.30 (s, 1H), 7.25 (dd, *J* = 19.9, 7.6 Hz, 3H), 7.01 (d, *J* = 7.6 Hz, 1H), 2.28 (s, 3H); ^13^C NMR (125.77 MHz, DMSO-d_6_) δ 165.58, 147.07, 145.20 (d, ^3^*J_F-C_* = 4.5 Hz), 143.61 (d, ^2^*J_F-C_* = 12.0 Hz), 143.23 (q, ^2^*J_F-C_* = 38.3 Hz), 141.43 (d, ^1^*J_F-C_* = 260.0 Hz), 138.80, 130.69, 129.25, 129.15, 127.93, 125.01, 121.61 (q, ^1^*J_F-C_* = 269.0 Hz), 114.24, 105.29, 21.31; ^19^F NMR (470.54 MHz, DMSO-d_6_) δ −61.09, −143.97; HRMS for C_17_H_11_ClF_4_N_4_O_2_([M + Na]^+^) calcd. 437.0404 and found 437.0407.

Compound **S140** 4-Amino-3-chloro-5-fluoro-6-(3-methyl-5-(o-tolyl)-1*H*-pyrazol-1-yl)-2-picolinic acid, a white solid with melting point of 180–184 °C; ^1^H NMR (500.13 MHz, DMSO-d_6_) δ 13.64 (s, 1H), 7.24 (d, *J* = 3.9 Hz, 2H), 7.11 (dd, *J* = 8.3, 4.1 Hz, 1H), 7.02 (s, 2H), 6.99 (d, *J* = 7.5 Hz, 1H), 6.39 (s, 1H), 2.29 (s, 4H), 2.17 (s, 3H); ^13^C NMR (125.77 MHz, DMSO-d_6_) δ 165.78, 149.70, 144.62 (d, ^3^*J_F-C_* = 4.6 Hz), 144.37, 142.96 (d, ^2^*J_F-C_* = 12.6 Hz), 140.96 (d, ^1^*J_F-C_* = 258.1 Hz), 137.18 (d, ^2^*J_F-C_* = 9.1 Hz), 136.96, 130.65, 130.01, 129.83, 129.23, 125.95, 112.75, 108.50, 20.26, 13.81; ^19^F NMR (470.54 MHz, DMSO-d_6_) δ −144.06; HRMS for C_17_H_14_ClFN_4_O_2_([M + H]^+^) calcd. 361.0868 and found 361.0872.

Compound **S143** 4-Amino-3-chloro-5-fluoro-6-(5-(o-tolyl)-3-(trifluoromethyl)-1*H*-pyrazol-1-yl)-2-picolinic acid, a white solid with melting point of 179–180 °C; ^1^H NMR (500.13 MHz, DMSO-d_6_) δ 13.83 (s, 1H), 7.35–7.28 (m, 2H), 7.24 (s, 2H), 7.20 (s, 1H), 7.17 (dd, *J* = 6.8, 2.1 Hz, 1H), 7.07 (dd, *J* = 7.6, 1.2 Hz, 1H), 2.21 (s, 3H); ^13^C NMR (125.77 MHz, DMSO-d_6_) δ 165.52, 146.03, 144.96 (d, ^3^*J_F-C_* = 4.5 Hz), 143.33 (d, ^2^*J_F-C_* = 12.4 Hz), 142.99 (q, ^2^*J_F-C_* = 37.3 Hz), 141.09 (d, ^1^*J_F-C_* = 260.0 Hz), 137.48, 135.84 (d, ^2^*J_F-C_* = 9.9 Hz), 130.85, 130.27, 130.23, 127.84, 126.19, 121.68 (q, ^1^*J_F-C_* = 268.9 Hz), 113.95 (d, ^4^*J_F-C_* = 2.4 Hz), 106.66, 20.08; ^19^F NMR (470.54 MHz, DMSO-d_6_) δ −60.89, −144.19; HRMS for C_17_H_11_ClF_4_N_4_O_2_([M + Na]^+^) calcd. 437.0404 and found 437.0407.

Compound **S080** 4-Amino-3-chloro-5-fluoro-6-(5-(4-fluorophenyl)-3-methyl-1*H*-pyrazol-1-yl)-2-picolinic acid, a white solid with melting point of 189 °C; ^1^H NMR (500.13 MHz, DMSO-d_6_) δ 13.66 (s, 1H), 7.27 (dd, ^3^*J_F-H_* = 8.8, 5.5 Hz, 2H), 7.25–7.19 (m, 2H), 7.14 (s, 2H), 6.55 (s, 1H), 2.27 (s, 3H); ^13^C NMR (125.77 MHz, DMSO-d_6_) δ 165.76, 162.44 (d, ^1^*J_F-C_* = 246.3 Hz), 150.15, 144.83 (d, ^3^*J_F-C_* = 4.6 Hz), 144.33, 143.34 (d, ^2^*J_F-C_* = 12.5 Hz), 141.26 (d, ^1^*J_F-C_* = 258.8 Hz), 137.32 (d, ^2^*J_F-C_* = 9.1 Hz), 129.96, 129.88, 126.41 (d, ^3^*J_F-C_* = 3.3 Hz), 116.32, 116.14, 113.22, 107.40, 13.72; ^19^F NMR (470.54 MHz, DMSO-d_6_) δ −112.89, −143.76; HRMS for C_16_H_11_ClF_2_N_4_O_2_([M + H]^+^) calcd. 365.0617 and found 365.0619.

Compound **S082** 4-Amino-3-chloro-6-(3-(difluoromethyl)-5-(4-fluorophenyl)-1*H*-pyrazol-1-yl)-5-fluoro-2-picolinic acid, a white solid with melting point of 188 °C; ^1^H NMR (500.13 MHz, DMSO-d_6_) δ 13.81 (s, 1H), 7.37 (dd, ^3^*J_F-H_* = 8.8, 5.4 Hz, 2H), 7.32–7.22 (m, 4H), 7.14 (t, ^1^*J_F-H_* = 51.3 Hz, 1H), 7.08 (s, 1H); ^13^C NMR (125.77 MHz, DMSO-d_6_) δ 165.57, 162.86 (d, ^1^*J_F-C_* = 247.5 Hz), 148.21 (t, ^2^*J_F-C_* = 28.8 Hz), 145.39, 145.05 (d, ^3^*J_F-C_* = 5.1 Hz), 143.62 (d, ^2^*J_F-C_* = 12.4 Hz), 141.27 (d, ^1^*J_F-C_* = 259.9 Hz), 136.41 (d, ^2^*J_F-C_* = 9.3 Hz), 130.48, 130.41, 125.25 (d, ^3^*J_F-C_* = 3.3 Hz), 116.55, 116.38, 114.01 (d, ^3^*J_F-C_* = 2.1 Hz), 111.62 (t, ^1^*J_F-C_* = 232.9 Hz), 104.76; ^19^F NMR (470.54 MHz, DMSO-d_6_) δ −111.71, −112.25, −143.94; HRMS for C_16_H_9_ClF_4_N_4_O_2_([M + Na]^+^) calcd. 423.0248 and found 423.0252.

Compound **S083** 4-Amino-3-chloro-5-fluoro-6-(5-(4-fluorophenyl)-3-(trifluoromethyl)-1*H*-pyrazol-1-yl)-2-picolinic acid, a white solid with melting point of 175–185 °C; ^1^H NMR (500.13 MHz, DMSO-d_6_) δ 13.87 (s, 1H), 7.40 (dd, ^3^*J_F-H_* = 8.8, 5.3 Hz, 2H), 7.34 (s, 2H), 7.33 (s, 1H), 7.29 (dd, ^4^*J_F-H_* = 8.8, 5.3 Hz, 2H); ^13^C NMR (125.77 MHz, DMSO-d_6_) δ 165.49, 163.05 (d, ^1^*J_F-C_* = 247.6 Hz), 145.91, 145.15 (d, ^3^*J_F-C_* = 4.5 Hz), 143.74 (d, ^2^*J_F-C_* = 12.5 Hz), 143.27 (q, ^2^*J_F-C_* = 37.4 Hz), 141.26 (d, ^1^*J_F-C_* = 260.1 Hz), 135.97 (d, ^2^*J_F-C_* = 10.0 Hz), 130.65 (d, ^2^*J_F-C_* = 8.3 Hz), 124.63 (d, ^3^*J_F-C_* = 3.6 Hz), 121.55 (q, ^1^*J_F-C_* = 269.0 Hz), 116.56 (d, ^2^*J_F-C_* = 21.7 Hz), 114.34 (d, ^3^*J_F-C_* = 3.2 Hz), 105.59; ^19^F NMR (470.54 MHz, DMSO-d_6_) δ −61.12, −111.16, −143.96; HRMS for C_16_H_8_ClF_5_N_4_O_2_([M + Na]^+^) calcd. 441.0154 and found 441.0155.

Compound **S170** 4-Amino-3-chloro-5-fluoro-6-(5-(3-fluorophenyl)-3-methyl-1*H*-pyrazol-1-yl)-2-picolinic acid, a white solid with melting point of 189–192 °C; ^1^H NMR (500.13 MHz, DMSO-d_6_) δ 7.40 (td, ^3^*J_F-H_* = 8.0, 6.1 Hz, 1H), 7.28–7.09 (m, 4H), 7.01 (dt, ^4^*J_F-H_* = 7.8, 1.2 Hz, 1H), 6.64 (s, 1H), 2.28 (s, 3H); ^13^C NMR (125.77 MHz, DMSO-d_6_) δ 165.76, 162.43 (d, ^1^*J_F-C_* = 244.2 Hz), 150.25, 144.87 (d, ^4^*J_F-C_* = 5.1 Hz), 143.96, 143.37 (d, ^2^*J_F-C_* = 13.2 Hz), 141.28 (d, ^1^*J_F-C_* = 258.8 Hz), 137.24 (d, ^2^*J_F-C_* = 9.5 Hz), 131.97 (d, ^3^*J_F-C_* = 8.2 Hz), 131.32 (d, ^3^*J_F-C_* = 8.3 Hz), 123.75 (d, ^4^*J_F-C_* = 2.4 Hz), 115.79 (d, ^2^*J_F-C_* = 20.6 Hz), 114.59 (d, ^2^*J_F-C_* = 22.8 Hz), 113.24, 107.97, 13.71; ^19^F NMR (470.54 MHz, DMSO-d_6_) δ −112.35, −143.79; HRMS for C_16_H_11_ClF_2_N_4_O_2_([M + H]^+^) calcd. 365.0617 and found 365.0613.

Compound **S172** 4-Amino-3-chloro-6-(3-(difluoromethyl)-5-(3-fluorophenyl)-1*H*-pyrazol-1-yl)-5-fluoro-2-picolinic acid, a white solid with melting point of 187–191 °C; ^1^H NMR (500.13 MHz, DMSO-d_6_) δ 13.85 (s, 1H), 7.49–7.42 (m, 1H), 7.31 (s, 2H), 7.26 (ddd, ^3^*J_F-H_* = 9.1, 4.6, 2.1 Hz, 2H), 7.17 (s, 1H), 7.28–7.03 (m, 1H), 7.13–7.06 (m, 1H); ^13^C NMR (125.77 MHz, DMSO-d_6_) δ 165.57, 162.45 (d, ^1^*J_F-C_* = 244.2 Hz), 148.27 (t, ^2^*J_F-C_* = 28.9 Hz), 145.08 (d, ^3^*J_F-C_* = 5.6 Hz), 145.00, 143.65 (d, ^2^*J_F-C_* = 12.4 Hz), 141.30 (d, ^1^*J_F-C_* = 260.1 Hz), 136.35 (d, ^2^*J_F-C_* = 9.5 Hz), 131.53 (d, ^3^*J_F-C_* = 8.9 Hz), 130.78 (d, ^3^*J_F-C_* = 8.8 Hz), 124.14 (d, ^4^*J_F-C_* = 2.7 Hz), 116.61 (d, ^2^*J_F-C_* = 21.2 Hz), 115.18 (d, ^2^*J_F-C_* = 23.3 Hz), 114.01 (d, ^4^*J_F-C_* = 2.1 Hz), 111.57 (t, ^1^*J_F-C_* = 232.9 Hz), 105.35; ^19^F NMR (470.54 MHz, DMSO-d_6_) δ −112.00, −112.34, −143.94; HRMS for C_16_H_9_ClF_4_N_4_O_2_([M + Na]^+^) calcd. 423.0248 and found 423.0252.

Compound **S173** 4-Amino-3-chloro-5-fluoro-6-(5-(3-fluorophenyl)-3-(trifluoromethyl)-1*H*-pyrazol-1-yl)-2-picolinic acid, a white solid with melting point of 150–178 °C; ^1^H NMR (500.13 MHz, DMSO-d_6_) δ 13.89 (s, 1H), 7.47 (td, ^3^*J_F-H_* = 8.0, 6.1 Hz, 1H), 7.42 (s, 1H), 7.36 (s, 2H), 7.33–7.27 (m, 2H), 7.14–7.08 (m, 1H); ^13^C NMR (125.77 MHz, DMSO-d_6_) δ 165.49, 162.44 (d, ^1^*J_F-C_* = 244.8 Hz), 145.51, 145.14 (d, ^3^*J_F-C_* = 4.7 Hz), 143.16 (q, ^2^*J_F-C_* = 38.2 Hz), 136.40 (d, ^1^*J_F-C_* = 217.1 Hz), 135.91 (d, ^2^*J_F-C_* = 9.1 Hz), 131.62 (d, ^3^*J_F-C_* = 8.3 Hz), 130.13 (d, ^3^*J_F-C_* = 8.3 Hz), 124.28 (d, ^4^*J_F-C_* = 3.1 Hz), 121.50 (q, ^1^*J_F-C_* = 268.9 Hz), 116.99 (d, ^2^*J_F-C_* = 20.6 Hz), 115.41 (d, ^2^*J_F-C_* = 23.6 Hz), 114.34 (d, ^4^*J_F-C_* = 2.5 Hz), 106.19; ^19^F NMR (470.54 MHz, DMSO-d_6_) δ −61.13, −111.87, −143.93; HRMS for C_16_H_8_ClF_5_N_4_O_2_([M + Na]^+^) calcd. 441.0154 and found 441.0158.

Compound **S180** 4-Amino-3-chloro-5-fluoro-6-(5-(2-fluorophenyl)-3-methyl-1*H*-pyrazol-1-yl)-2-picolinic acid, a white solid with melting point of 99–131°C; ^1^H NMR (500.13 MHz, DMSO-d_6_) δ 13.60 (s, 1H), 7.41 (dddd, ^3^*J_F-H_* = 8.6, 7.2, 5.2, 1.9 Hz, 1H), 7.28–7.19 (m, 3H), 7.09 (s, 2H), 6.54 (s, 1H), 2.30 (s, 3H); ^13^C NMR (125.77 MHz, DMSO-d_6_) δ 165.72, 158.91 (d, ^1^*J_F-C_* = 247.5 Hz), 150.18, 144.48 (d, ^3^*J_F-C_* = 5.4 Hz), 143.24 (d, ^2^*J_F-C_* = 12.7 Hz), 140.59 (d, ^1^*J_F-C_* = 259.1 Hz), 139.13, 137.41 (d, ^3^*J_F-C_* = 8.4 Hz), 131.38 (d, ^3^*J_F-C_* = 8.6 Hz), 130.82, 125.09 (d, ^4^*J_F-C_* = 3.4 Hz), 118.15 (d, ^2^*J_F-C_* = 13.7 Hz), 116.38 (d, ^2^*J_F-C_* = 22.1 Hz), 112.69, 109.36, 13.7; ^19^F NMR (470.54 MHz, DMSO-d_6_) δ −114.82, −144.34; HRMS for C_16_H_11_ClF_2_N_4_O_2_([M + H]^+^) calcd. 365.0617 and found 365.0620.

Compound **S182** 4-Amino-3-chloro-6-(3-(difluoromethyl)-5-(2-fluorophenyl)-1*H*-pyrazol-1-yl)-5-fluoro-2-picolinic acid, a white solid with melting point of 120–177°C; ^1^H NMR (500.13 MHz, DMSO-d_6_) δ 13.69 (s, 1H), 7.51–7.45 (m, 1H), 7.35 (td, ^3^*J_F-H_* = 7.6, 1.7 Hz, 1H), 7.31–7.21 (m, 4H), 7.18 (t, ^1^*J_F-H_* = 54.3 Hz, 1H), 7.05 (s, 1H); ^13^C NMR (125.77 MHz, DMSO-d_6_) δ 165.55, 159.03 (d, ^1^*J_F-C_* = 247.7 Hz), 148.19 (t, ^2^*J_F-C_* = 28.9 Hz), 144.73 (d, ^3^*J_F-C_* = 4.9 Hz), 143.51 (d, ^2^*J_F-C_* = 12.4 Hz), 140.68 (d, ^1^*J_F-C_* = 260.2 Hz), 140.32, 136.52 (d, ^3^*J_F-C_* = 9.1 Hz), 132.27 (d, ^3^*J_F-C_* = 8.5 Hz), 131.19, 125.32, 116.98 (d, ^2^*J_F-C_* = 14.4 Hz), 116.49 (d, ^2^*J_F-C_* = 21.5 Hz), 113.53 (d, ^4^*J_F-C_* = 1.9 Hz), 111.56 (t, ^1^*J_F-C_* = 233.0 Hz), 106.73; ^19^F NMR (470.54 MHz, DMSO-d_6_) δ −112.29, −114.79, −144.53; HRMS for C_16_H_9_ClF_4_N_4_O_2_([M + Na]^+^) calcd. 423.0248 and found 423.0248.

Compound **S183** 4-Amino-3-chloro-5-fluoro-6-(5-(2-fluorophenyl)-3-(trifluoromethyl)-1*H*-pyrazol-1-yl)-2-picolinic acid, a white solid with melting point of 161–173 °C; ^1^H NMR (500.13 MHz, DMSO-d_6_) δ 13.79 (s, 1H), 7.51 (tdd, ^4^*J_F-H_* = 7.6, 5.2, 1.8 Hz, 1H), 7.39 (td, ^3^*J_F-H_* = 7.6, 1.8 Hz, 1H), 7.36–7.21 (m, 5H); ^13^C NMR (125.77 MHz, DMSO-d_6_) δ 165.46, 159.06 (d, ^1^*J_F-C_* = 248.0 Hz), 144.88 (d, ^3^*J_F-C_* = 4.5 Hz), 143.61 (d, ^2^*J_F-C_* = 12.5 Hz), 143.24 (q, ^2^*J_F-C_* = 37.8 Hz), 140.91, 140.69 (d, ^1^*J_F-C_* = 260.8 Hz), 136.08 (d, ^2^*J_F-C_* = 9.0 Hz), 132.67 (d, ^3^*J_F-C_* = 8.5 Hz), 131.34, 125.39 (d, ^3^*J_F-C_* = 3.3 Hz), 121.49 (q, ^1^*J_F-C_* = 268.8 Hz), 116.53 (d, ^2^*J_F-C_* = 21.5 Hz), 116.35 (d, ^2^*J_F-C_* = 14.7 Hz), 113.86, 107.50; ^19^F NMR (470.54 MHz, DMSO-d_6_) δ −61.05, −114.71, −144.57; HRMS for C_16_H_8_ClF_5_N_4_O_2_([M + Na]^+^) calcd. 441.0154 and found 441.0159.

Compound **S313** 4-Amino-3-chloro-5-fluoro-6-(5-(4-hydroxyphenyl)-3-(trifluoromethyl)-1*H*-pyrazol-1-yl)-2-picolinic acid, a white solid with melting point 211–218 °C; ^1^H NMR (500.13 MHz, DMSO-d_6_) δ 13.77 (s, 1H), 9.94 (s, 1H), 7.30 (s, 2H), 7.16–7.12 (m, 3H), 6.77 (d, *J* = 8.6 Hz, 2H); ^13^C NMR (125.77 MHz, DMSO-d_6_) δ 165.60, 159.04, 147.26, 145.16 (d, *J* = 4.8 Hz), 143.61 (d, ^2^*J_F-C_* = 12.4 Hz), 143.14 (q, ^2^*J_F-C_* = 37.7 Hz), 141.41 (d, ^1^*J_F-C_* = 260.0 Hz), 136.39 (d, ^2^*J_F-C_* = 10.0 Hz), 129.67, 121.67 (q, ^1^*J_F-C_* = 269.0 Hz), 118.69, 116.21, 114.22 (d, ^3^*J_F-C_* = 2.2 Hz), 104.21; ^19^F NMR (470.54 MHz, DMSO-d_6_) δ −61.08, −143.79; HRMS for C_16_H_9_ClF_4_N_4_O_3_([M + Na]^+^) calcd. 439.0197 and found 439.0201.

Compound **S333** 4-Amino-3-chloro-5-fluoro-6-(5-(2-hydroxyphenyl)-3-(trifluoromethyl)-1*H*-pyrazol-1-yl)-2-picolinic acid, a white solid with melting point of 190–196 °C; ^1^H NMR (500.13 MHz, DMSO-d_6_) δ 10.17 (s, 1H), 7.85 (dd, *J* = 7.8, 1.6 Hz, 1H), 7.64 (s, 1H), 7.44 (s, 2H), 7.30–7.23 (m, 1H), 7.03 (d, *J* = 7.7 Hz, 1H), 6.96–6.89 (m, 1H); ^13^C NMR (125.77 MHz, DMSO-d_6_) δ 165.66, 155.70, 150.46, 144.85 (d, ^3^*J_F-C_* = 4.6 Hz), 143.76 (d, ^2^*J_F-C_* = 12.4 Hz), 141.29 (d, ^1^*J_F-C_* = 260.8 Hz), 135.57 (d, ^2^*J_F-C_* = 9.0 Hz), 133.02 (q, ^2^*J_F-C_* = 39.3 Hz), 130.75, 128.25, 119.92, 119.88 (q, ^1^*J_F-C_* = 269.2 Hz), 117.36, 117.02, 114.25 (d, ^4^*J_F-C_* = 2.0 Hz), 110.17; ^19^F NMR (470.54 MHz, DMSO-d_6_) δ −57.82, −144.28; HRMS for C_16_H_9_ClF_4_N_4_O_3_([M + Na]^+^) calcd.439.0197 and found 439.0196.

Compound **S343** 4-Amino-6-(5-(4-carboxyphenyl)-3-(trifluoromethyl)-1*H*-pyrazol-1-yl)-3-chloro-5-fluoro-2-picolinic acid, a white solid with melting point of 226 °C; ^1^H NMR (500.13 MHz, DMSO-d_6_) δ 13.29 (s, 2H), 7.96 (d, *J* = 8.4 Hz, 2H), 7.48 (d, *J* = 8.4 Hz, 2H), 7.45 (s, 1H), 7.35 (s, 2H); ^13^C NMR (125.77 MHz, DMSO-d_6_) δ 167.06, 165.49, 145.91, 145.16 (d, ^3^*J_F-C_* = 4.3 Hz), 143.79 (d, ^2^*J_F-C_* = 12.4 Hz), 143.42 (q, ^2^*J_F-C_* = 37.9 Hz), 141.20 (d, ^1^*J_F-C_* = 260.2 Hz), 135.93 (d, ^2^*J_F-C_* = 9.5 Hz), 132.01, 131.92, 130.25, 128.45, 121.49 (q, ^1^*J_F-C_* = 269.0 Hz), 114.39 (d, ^4^*J_F-C_* = 2.7 Hz), 106.33; ^19^F NMR (470.54 MHz, DMSO-d_6_) δ −61.12, −144.08; HRMS for C_17_H_9_ClF_4_N_4_O_4_([M + Na]^+^) calcd. 467.0146 and found 467.0150.

Compound **S113** 4-Amino-6-(5-(4-aminophenyl)-3-(trifluoromethyl)-1*H*-pyrazol-1-yl)-3-chloro-5-fluoro-2-picolinic acid, a white solid with melting point of 213 °C; ^1^H NMR (500.13 MHz, DMSO-d_6_) δ 7.27 (s, 2H), 7.04 (s, 1H), 6.96 (d, *J* = 8.7 Hz, 2H), 6.51 (d, *J* = 8.6 Hz, 2H); ^13^C NMR (125.77 MHz, DMSO-d_6_) δ 165.69, 150.48, 148.04, 145.31 (d, ^3^*J_F-C_* = 4.4 Hz), 143.53 (d, ^2^*J_F-C_* = 12.4 Hz), 143.06 (q, ^2^*J_F-C_* = 37.6 Hz), 141.48 (d, ^1^*J_F-C_* = 259.8 Hz), 136.69 (d, ^2^*J_F-C_* = 10.0 Hz), 129.03, 121.76 (q, ^1^*J_F-C_* = 268.9 Hz), 114.74, 114.07, 103.12; ^19^F NMR (470.54 MHz, DMSO-d_6_) δ −61.08, −143.69; HRMS for C_16_H_10_ClF_4_N_5_O_2_([M + H]^+^) calcd. 416.0537 and found 416.0542.

Compound **S233** 4-Amino-3-chloro-5-fluoro-6-(5-(4-nitrophenyl)-3-(trifluoromethyl)-1*H*-pyrazol-1-yl)-2-picolinic acid, a white solid with melting point of 211–212 °C; ^1^H NMR (500.13 MHz, DMSO-d_6_) δ 13.88 (s, 1H), 8.29–8.24 (m, 2H), 7.70–7.62 (m, 2H), 7.57 (s, 1H), 7.39 (s, 2H); ^13^C NMR (125.77 MHz, DMSO-d_6_) δ 165.41, 148.12, 145.13 (d, ^3^*J_F-C_* = 4.6 Hz), 144.71, 143.94 (d, ^2^*J_F-C_* = 12.3 Hz), 143.50 (q, ^2^*J_F-C_* = 38.1 Hz), 141.10 (d, ^1^*J_F-C_* = 261.0 Hz), 135.67 (d, ^2^*J_F-C_* = 9.1 Hz), 134.21, 129.66, 124.54, 121.40 (q, ^1^*J_F-C_* = 337.9 Hz), 114.49, 107.22; ^19^F NMR (470.54 MHz, DMSO-d_6_) δ −61.16, −144.08; HRMS for C_16_H_8_ClF_4_N_5_O_4_([M + H]^+^) calcd. 446.0279 and found 446.0284.

### 3.3. Biological Assays

#### 3.3.1. *A. thaliana* Root Growth Inhibition

*A. thaliana* seeds were disinfected with a 5% sodium hypochlorite solution for 15 min, followed by rinsing with sterile water 7 to 8 times. Then, the disinfected *A. thaliana* seeds were spread evenly onto the 1/2 M/S culture medium containing a certain concentration of tested compounds in Petri dishes. Subsequently, the dishes were transferred to the dark at 4 °C for 48 h and then were placed vertically into the incubator at 22 °C for 16 h:8 h (day/night). After a 7-day culture, the root lengths of *A. thaliana* were measured using IMAGEJ, and the inhibitory activity of tested compounds were determined with the formula below:

$$I_{1}=\frac{P_{a0}-P_{a}}{P_{a0}}\times 100\%$$

where *I*_1_ is the inhibitory activity, and *P_a_* and *P_a_*_0_ are the mean root lengths of the *A. thaliana* treated by the target compounds and with no treatment controls, respectively.

#### 3.3.2. Weeds Root Growth Inhibition

The compounds **S** were dissolved in DMSO to obtain a concentrated stock solution and diluted with Tween-8 (0.1%) to obtain a specific concentration solution. A sheet of seed germination paper was placed at the bottom of a Petri dish, 10 weed seeds ready for germination were evenly distributed on the germination paper, and the specific diluted solution were added into the Petri dish. The Petri dish was covered with its lid and transferred to an incubator at 22 °C for 16 h:8 h (day/night). After 7 days, the root length of the weed seeds was measured using IMAGEJ, and the inhibitory activity of tested compounds were determined as formula below:

$$I_{2}=\frac{P_{b0}-P_{b}}{P_{b0}}\times 100\%$$

where *I*_2_ is the inhibitory activity, and *P_b_* and *P_b_*_0_ are the mean root lengths of the weed seeds treated by the target compounds and in no treatment controls, respectively.

#### 3.3.3. Post Emergence Herbicidal Activity

Compounds S were dissolved in a mixed solution of acetone and DMSO at a ratio of 97:3 (*v*/*v*) to obtain a concentrated stock solution. The concentrated stock solution was then diluted with a mixture of acetone, water, isopropanol, DMSO, Atplus 411F crop oil concentrate, and Triton^®^X-155 surfactant at a ratio of 45:39:10:1.5:1.0:0.02 (*v*/*v*) to obtain a spray solution. The weeds were treated with spray solutions at their 2–3 leaf stage. The herbicidal effect of the compounds was evaluated 14 days after treatment by visual observation based on the index of all dead: 100%; stems atrophy and dead leaves: 80%; stems atrophy and partially dead leaves: 60%; normal stems and partially dead leaves: 40%; normal stems and partially curled leaves: 20%; and normal stems and leaves = 0 [29].

## 4. Conclusions

This study designed and synthesized 41 novel 4-amino-6-(5-aryl-substituted-1-pyrazolyl)-3-chloro-5-fluoro-2-picolinic acid compounds. The results of inhibiting *A. thaliana* root growth demonstrated that most compounds **S** had a superior inhibitory effect when the R_1_ group was at the 2 or 4 position of the phenyl fragment. In particular, at a concentration of 5 µmol/L, thirty compounds **S** demonstrated better inhibitory effects than picloram; at a concentration of 0.5 µmol/L, compounds **S070**, **S150**, **S202**, **S203**, **S060**, **S140**, and **S180** exhibited excellent inhibitory effects, which were better than florpyrauxifen. Most compounds exhibited significant inhibitory effects on the growth of BN seeds, while having little inhibitory effect on the germination and growth of EC seeds. Compound **S063** demonstrated superior overall control efficacy against four gramineous weed species, outperforming picloram in the greenhouse. In summary, the newly synthesized compounds exhibited excellent herbicidal activity in the initial screening, and several could be used as promising lead molecules for herbicidal molecule discovery. Continuous optimization and modification of this series of compounds holds promise for the development of efficient and novel 6-heteroaryl-2-picolinic acid herbicidal molecules.

## Figures and Tables

**Table 1:** Herbicidal activity of compounds **S** inhibiting three weeds root growth.

Compd.	500 µM			250 µM		
	AM	BN	EC	AM	BN	EC
**S070**	93.7	100.0	100.0	94.0	94.7	91.4
**S073**	80.8	94.5	70.7	92.6	/	/
**S150**	83.3	92.9	30.6	76.5	88.7	/
**S152**	86.0	90.4	59.2	76.0	93.2	/
**S153**	57.1	92.9	42.1	/	80.9	/
**S160**	100.0	90.8	55.1	68.9	79.7	/
**S162**	51.6	84.8	7.3	/	71.7	/
**S163**	49.4	86.5	51.8	/	70.0	/
**S200**	82.4	86.8	45.0	80.8	88.5	/
**S202**	82.6	87.5	47.0	80.6	89.7	/
**S203**	71.8	87.8	34.6	/	87.5	/
**S100**	82.3	85.6	17.8	82.4	84.5	/
**S102**	100.0	96.3	0.0	71.1	76.8	/
**S103**	67.7	86.3	0.0	/	80.6	/
**S190**	40.9	86.2	0.0	/	0.0	/
**S193**	63.9	87.0	37.9	/	83.4	/
**S060**	79.1	87.9	31.6	/	89.6	/
**S062**	77.1	86.6	43.5	/	86.9	/
**S063**	77.1	87.9	100.0	/	88.8	35.3
**S130**	81.1	92.3	0.0	77.7	82.1	/
**S133**	43.4	86.9	0.0	/	82.7	/
**S123**	85.7	94.3	80.1	89.5	91.5	41.0
**S053**	74.4	89.3	51.9	/	84.4	/
**S040**	76.3	84.0	41.4	/	85.7	/
**S043**	57.7	82.6	39.6	/	76.0	/
**S140**	91.7	95.3	70.8	92.8	92.2	/
**S143**	78.7	92.7	0.0	/	83.7	/
**S080**	80.5	91.2	0.0	79.9	88.6	/
**S082**	75.4	90.0	45.1	/	87.0	/
**S083**	58.7	90.6	72.5	/	84.6	/
**S170**	50.2	88.3	0.0	/	86.2	/
**S172**	70.6	0.0	0.0	/	/	/
**S173**	100.0	91.0	31.4	75.0	82.3	/
**S180**	88.2	90.1	58.0	85.9	90.2	/
**S182**	72.1	92.2	61.2	/	89.4	/
**S183**	63.0	92.6	38.4	/	86.5	/
**S313**	37.6	0.0	0.0	/	/	/
**S333**	100.0	28.1	0.0	75.6	/	/
**S343**	100.0	5.2	0.0	/	/	/
**S113**	44.5	24.9	0.0	/	/	/
**S233**	58.7	45.4	0.0	/	/	/
**picloram**	100.0	100.0	100.0	100.0	100.0	100.0
**florpyrauxifen**	100.0	100.0	100.0	100.0	100.0	100.0

## Data Availability

Data are contained within the article and Supplementary Materials.

## Supplementary Materials

The following supporting information can be downloaded at: https://www.mdpi.com/article/10.3390/molecules30051022/s1.
