# Supplementary material for: Study on Design, Synthesis and Herbicidal Activity of Novel 4-Amino-6-(5-Aryl-Substituted-1-Pyrazolyl)-3-Chloro-5-Fluoro-2-Picolinic Acids
Source: Molecules. 2025 Feb 23;30(5):1022. doi: 10.3390/molecules30051022 (PMC11901791; doi:10.3390/molecules30051022)
Supplement: Supplementary file 1 [file molecules-30-01022-s001.zip › molecules-3476313-supplementary.pdf]

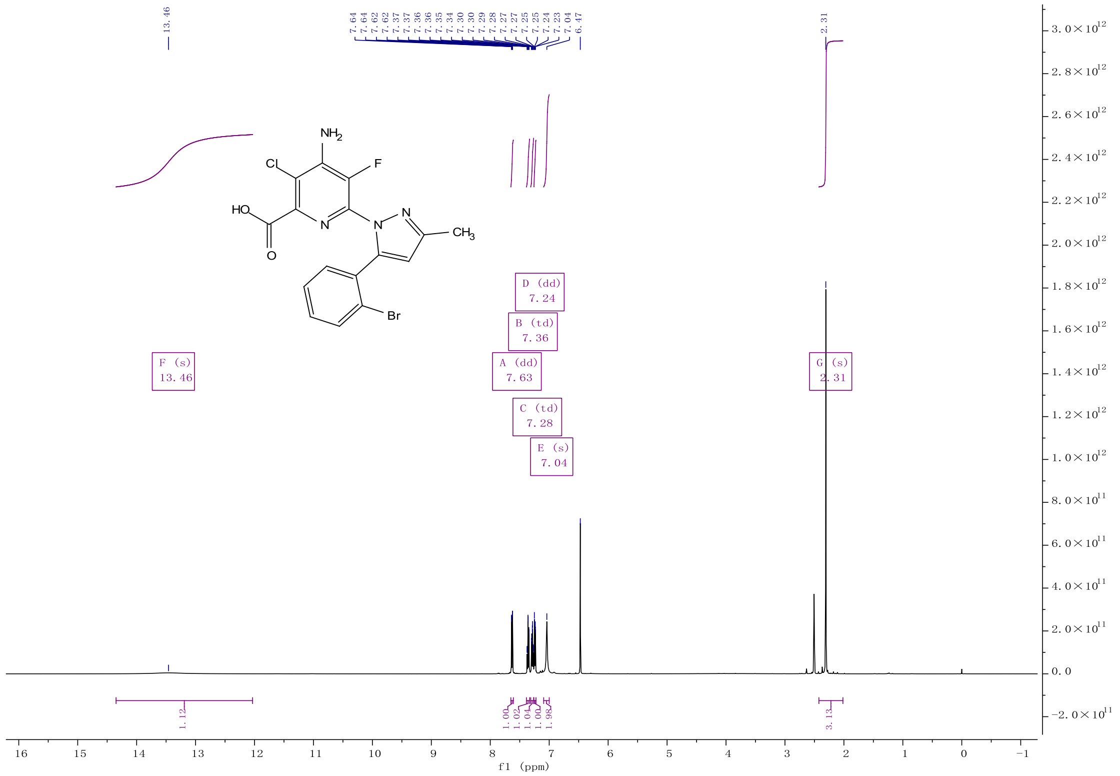

Figure S1. ^{1}H NMR (500.13 MHz, DMSO-d6) spectrum of compound S070.

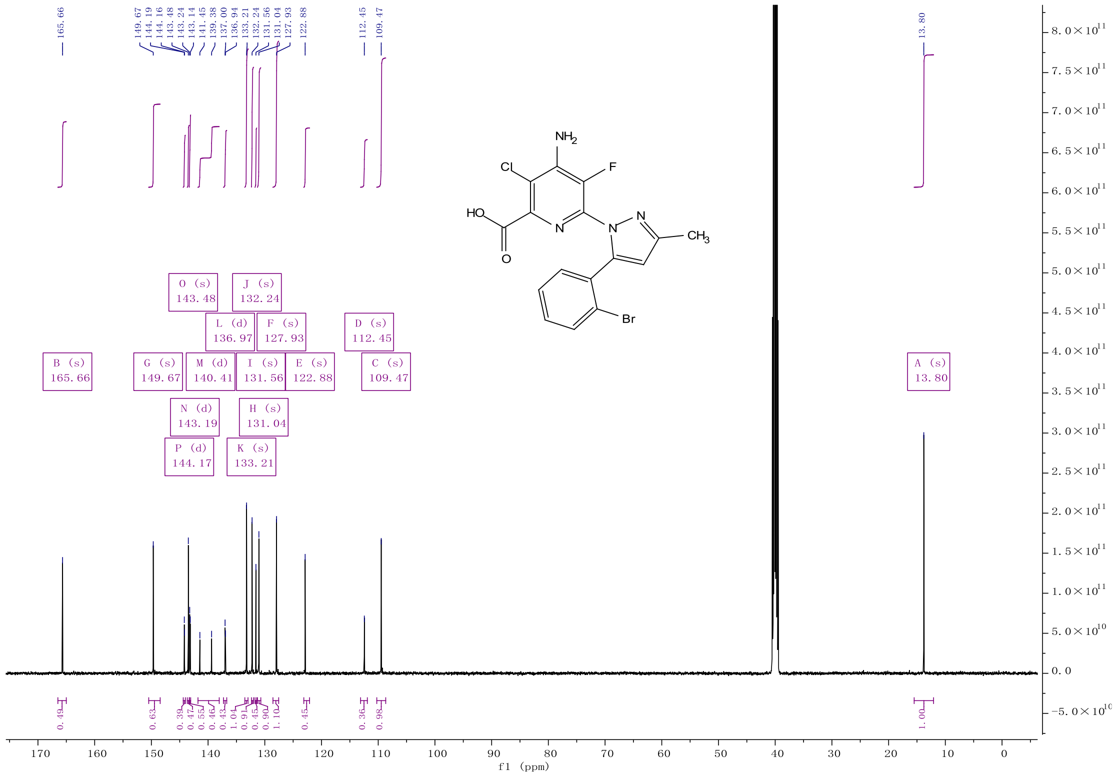

Figure S2. ^{13}C NMR (125.77 MHz, DMSO-d_6) spectrum of compound S070.

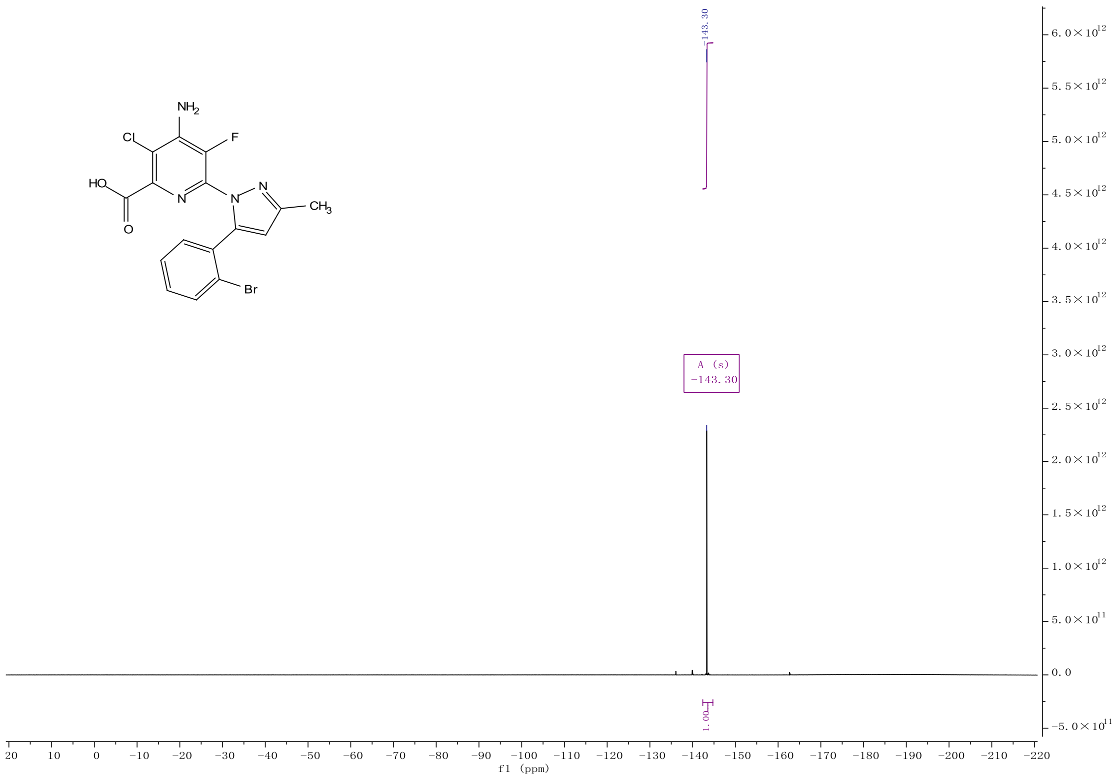

Figure S3. ^{19}F NMR (470.54 MHz, DMSO-d6) spectrum of compound S070.

Single Mass Analysis
Tolerance = 5.0 mDa / DBE: min = -1.5, max = 50.0
Element prediction: Off
Number of isotope peaks used for i-FIT = 3

Monoisotopic Mass, Even Electron Ions
6644 formula(e) evaluated with 1 results within limits (up to 50 best isotopic matches for each mass)
Elements Used:
C: 16-16 H: 12-12 N: 0-50 O: 0-50 F: 1-6 Na: 0-3 Cl: 1-2 Br: 1-2

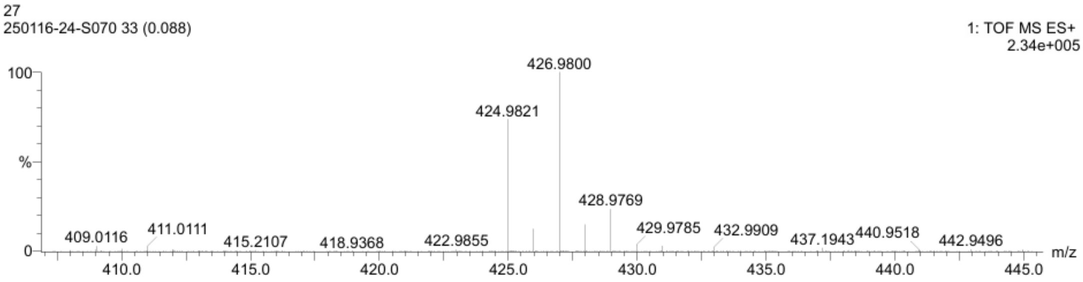

Mass	Calc. Mass	mDa	PPM	DBE	i-FIT	Norm	Conf(%)	Formula
Minimum:				-1.5				
Maximum:		5.0	10.0	50.0				
424.9821	424.9816	0.5	1.2	11.5	438.7	n/a	n/a	C16 H12 N4 O2 F Cl Br

Figure S4. HRMS spectrum of compound S070.

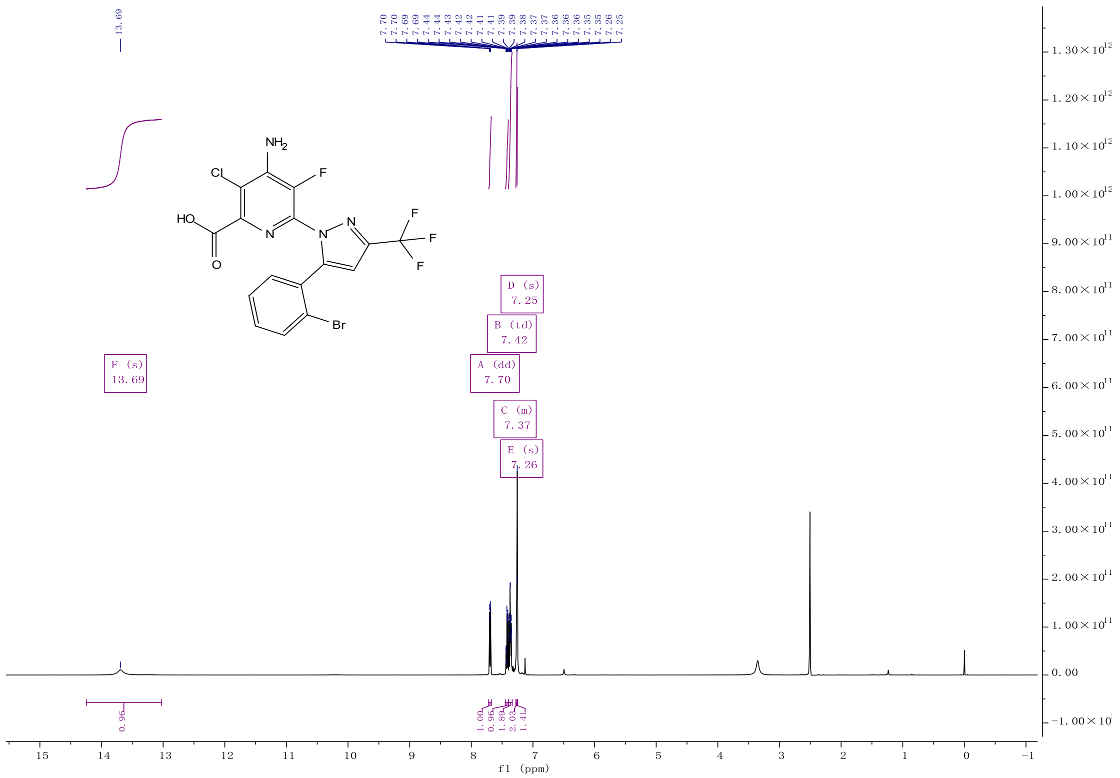

Figure S5. ^{1}H NMR (500.13 MHz, DMSO-d6) spectrum of compound S073.

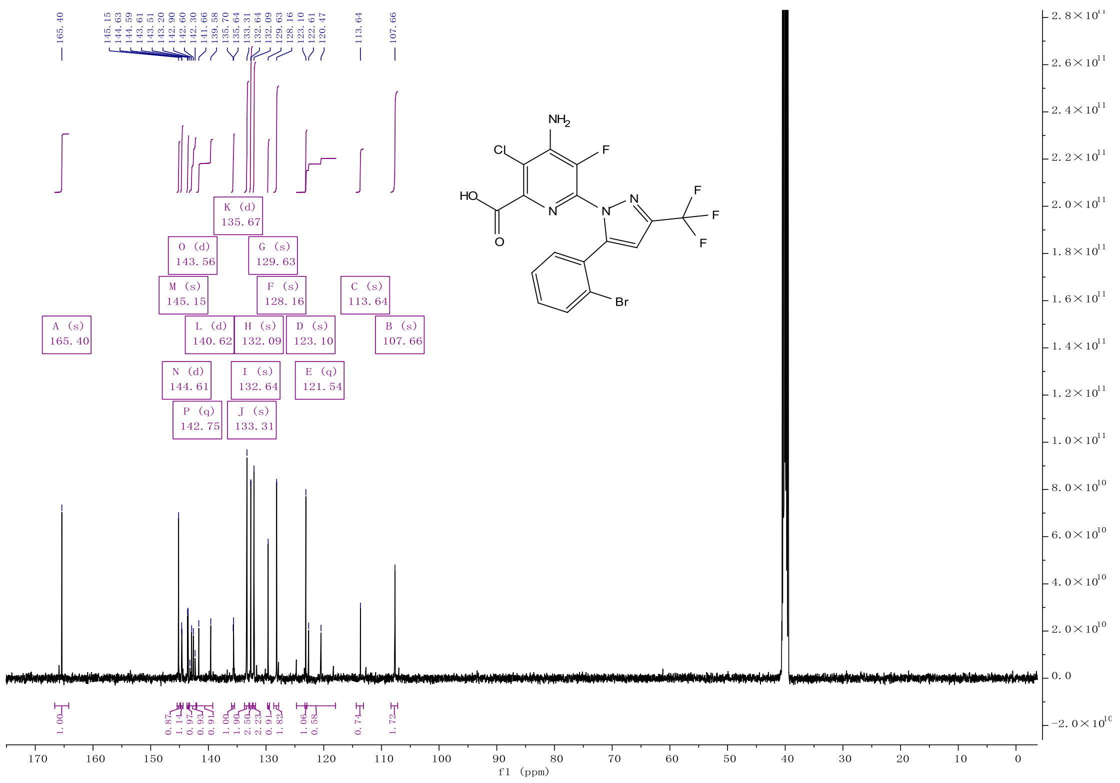

Figure S6. ^{13}C NMR (125.77 MHz, DMSO-d_6) spectrum of compound S073.

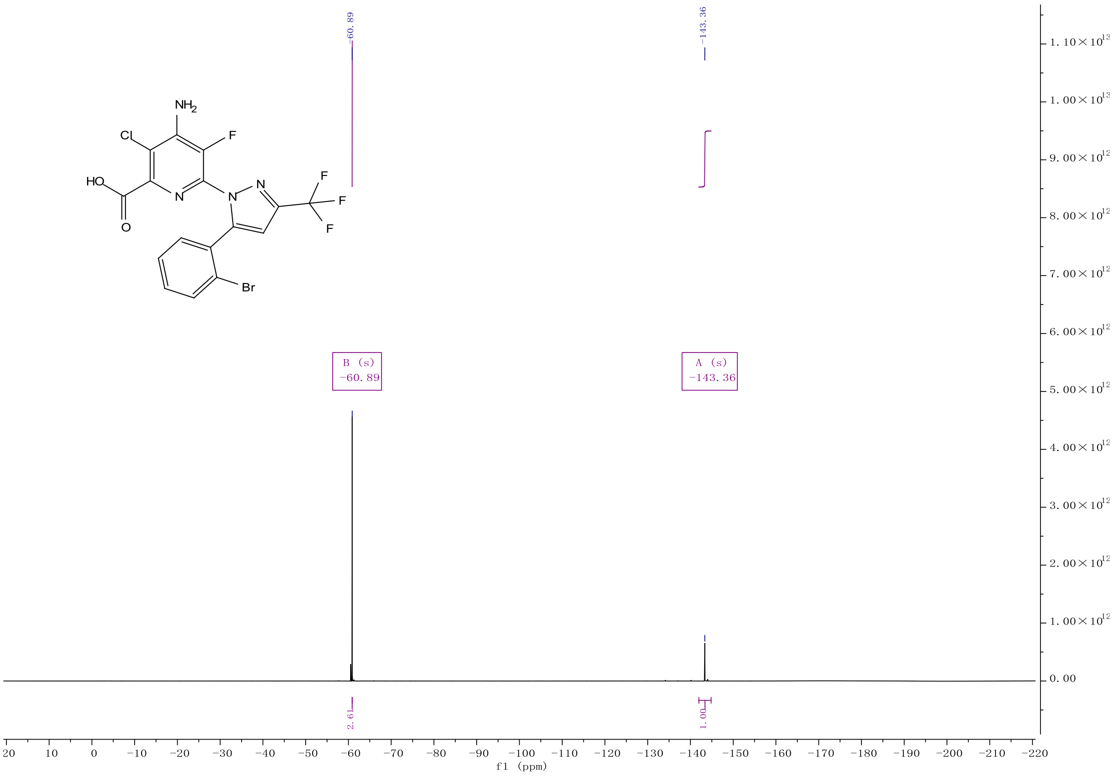

Figure S7. ^{19}F NMR (470.54 MHz, DMSO-d6) spectrum of compound S073.

Single Mass Analysis
Tolerance = 5.0 mDa / DBE: min = -1.5, max = 50.0
Element prediction: Off
Number of isotope peaks used for i-FIT = 3

Monoisotopic Mass, Even Electron Ions
12576 formula(e) evaluated with 1 results within limits (up to 50 best isotopic matches for each mass)
Elements Used:
C: 16-16 H: 8-8 N: 0-50 O: 0-50 F: 1-6 Na: 0-3 Cl: 1-2 Br: 1-2

27
250116-24-S073 35 (0.092) 1: TOF MS ES+
3.85e+004

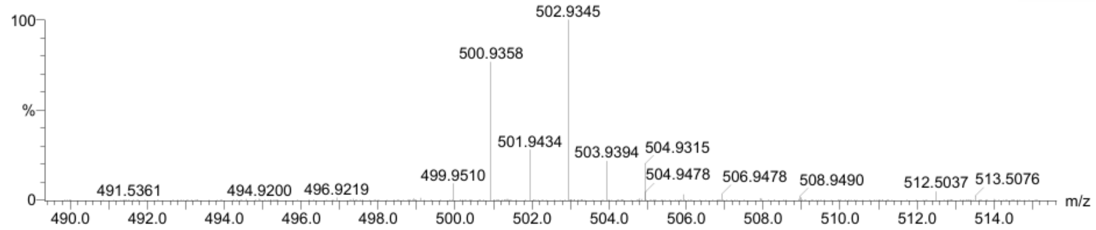

Minimum:				-1.5				
Maximum:		5.0	10.0	50.0				
Mass	Calc. Mass	mDa	PPM	DBE	i-FIT	Norm	Conf(%)	Formula
500.9358	500.9353	0.5	1.0	11.5	318.9	n/a	n/a	C16 H8 N4 O2 F4 Na Cl Br

Figure S8. HRMS spectrum of compound S073.

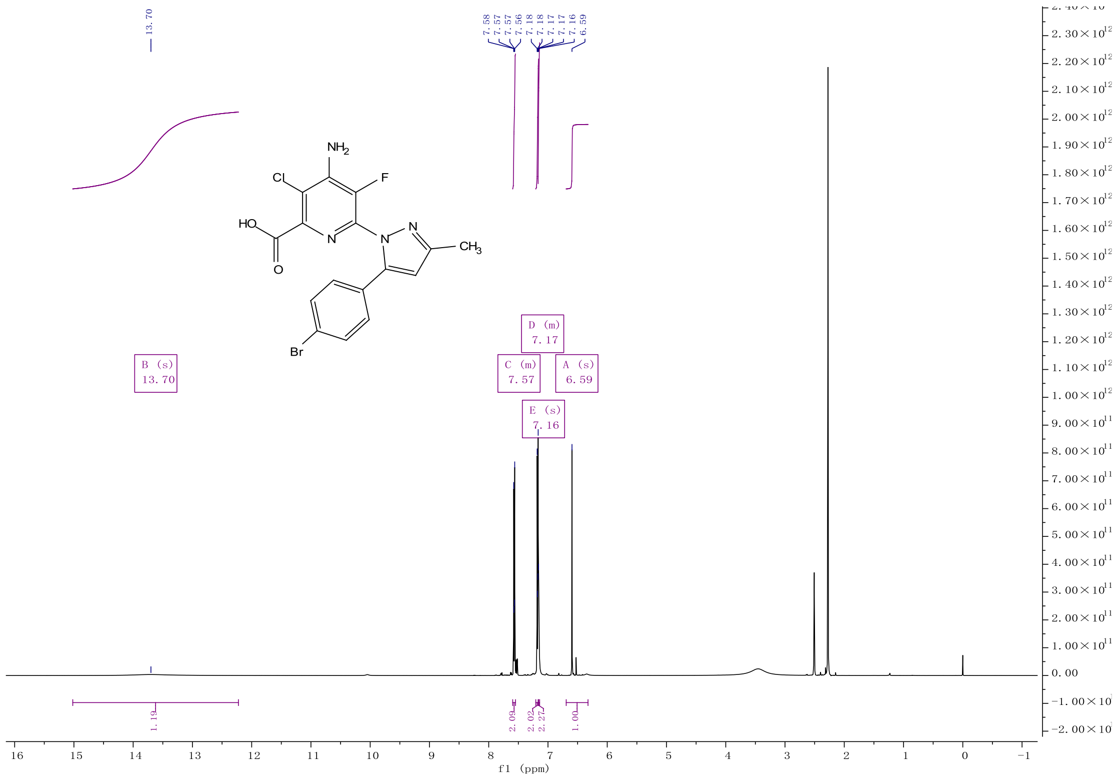

Figure S9. ^{1}H NMR (500.13 MHz, DMSO-d6) spectrum of compound S150.

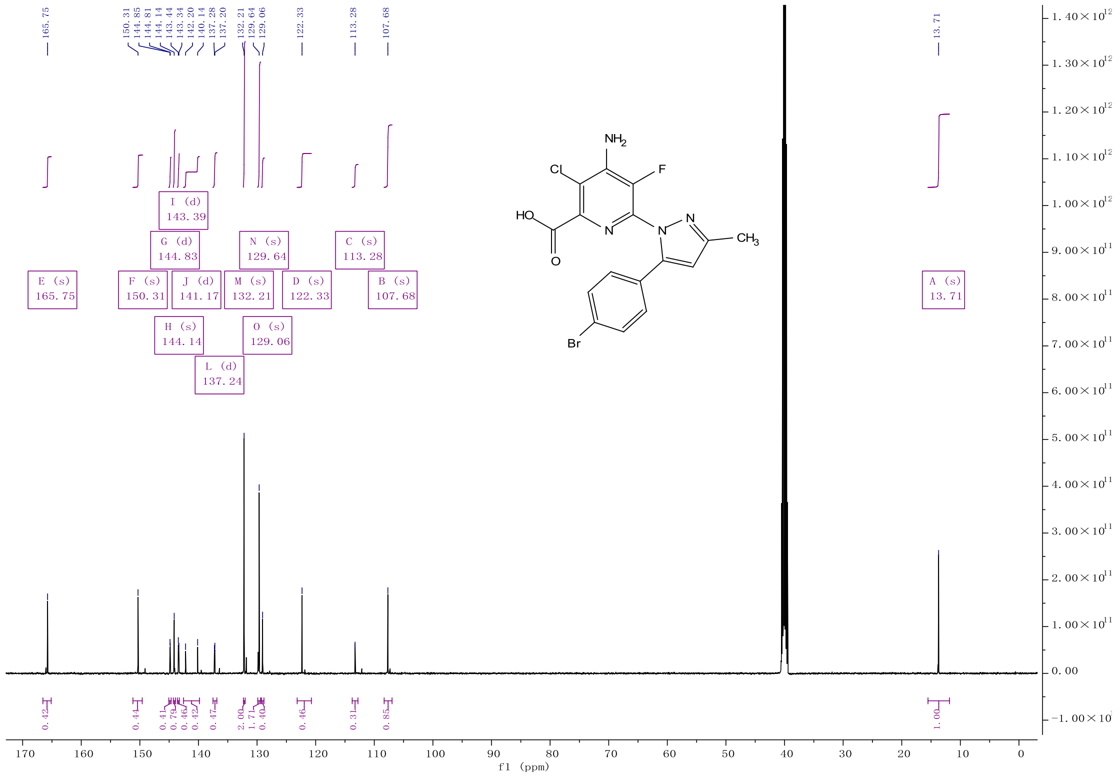

Figure S10. ^{13}C NMR (125.77 MHz, DMSO-d_6) spectrum of compound S150.

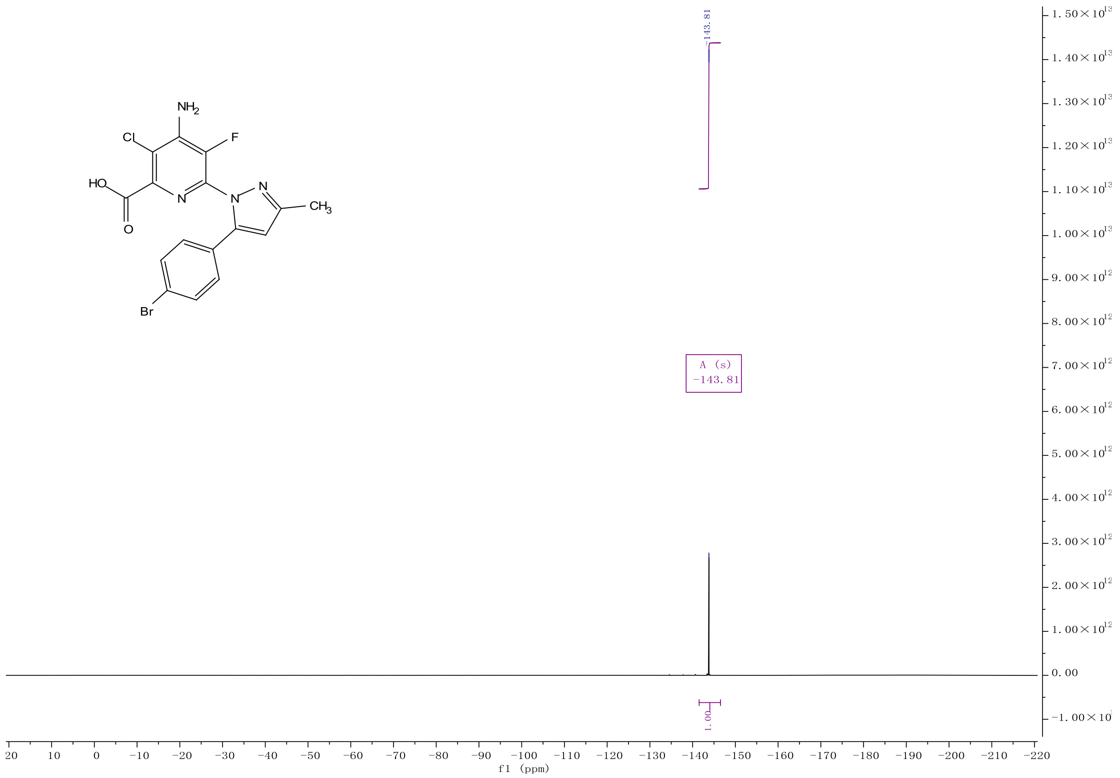

Figure S11. ^{19}F NMR (470.54 MHz, DMSO-d6) spectrum of compound S150.

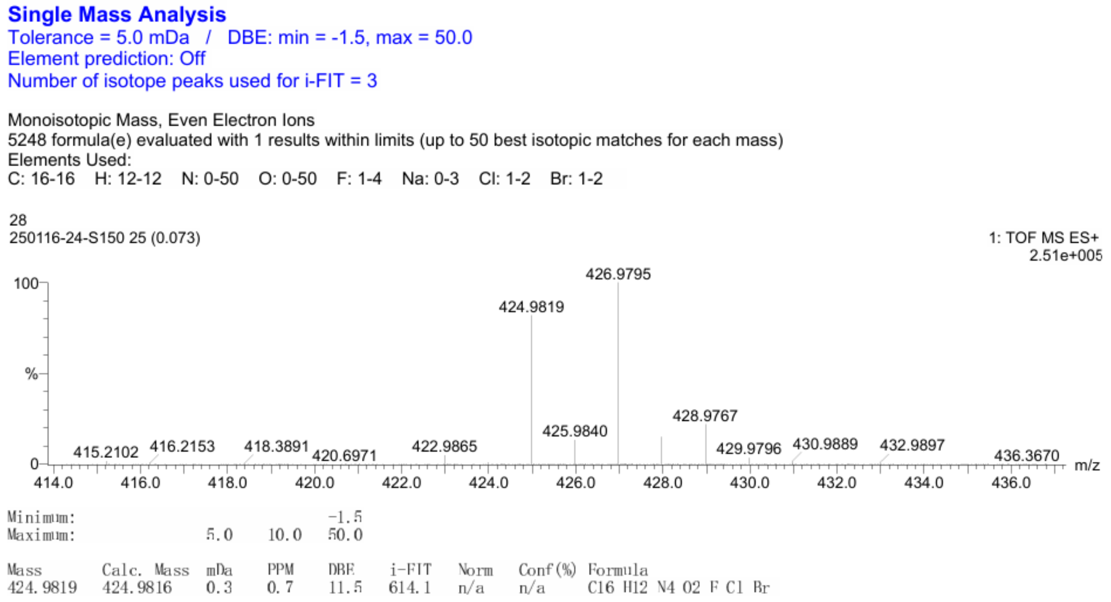

Figure S12. HRMS spectrum of compound S150.

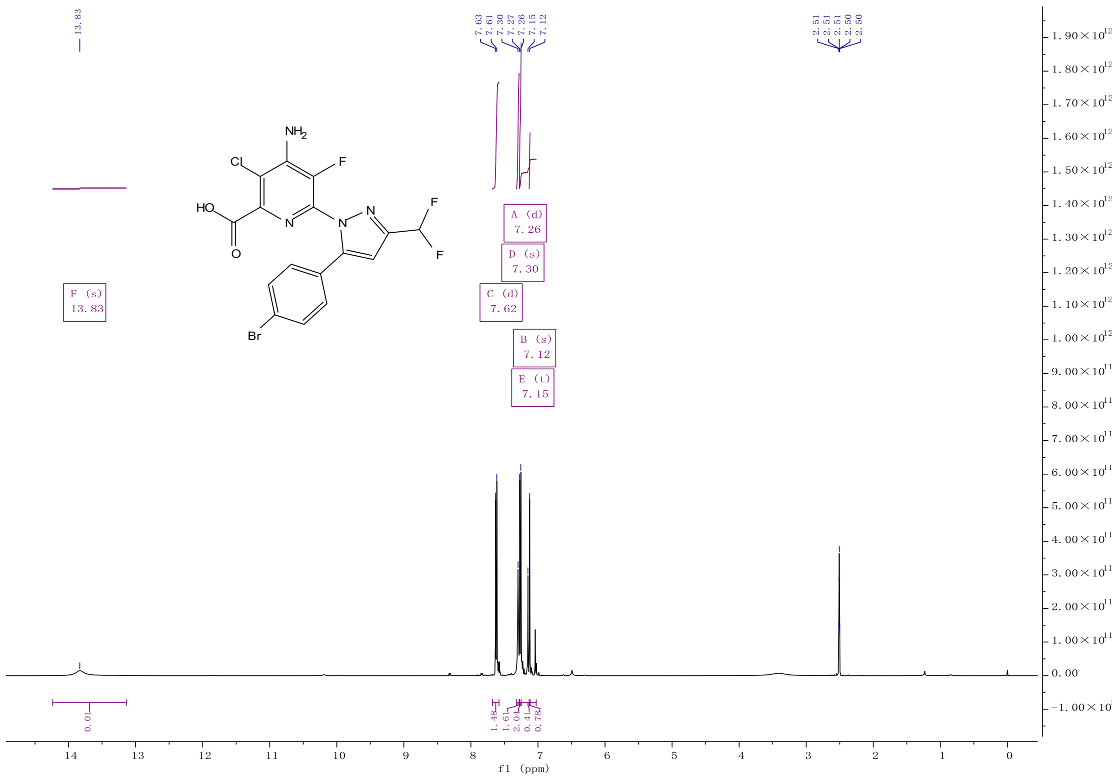

Figure S13. ^{1}H NMR (500.13 MHz, DMSO-d6) spectrum of compound S152.

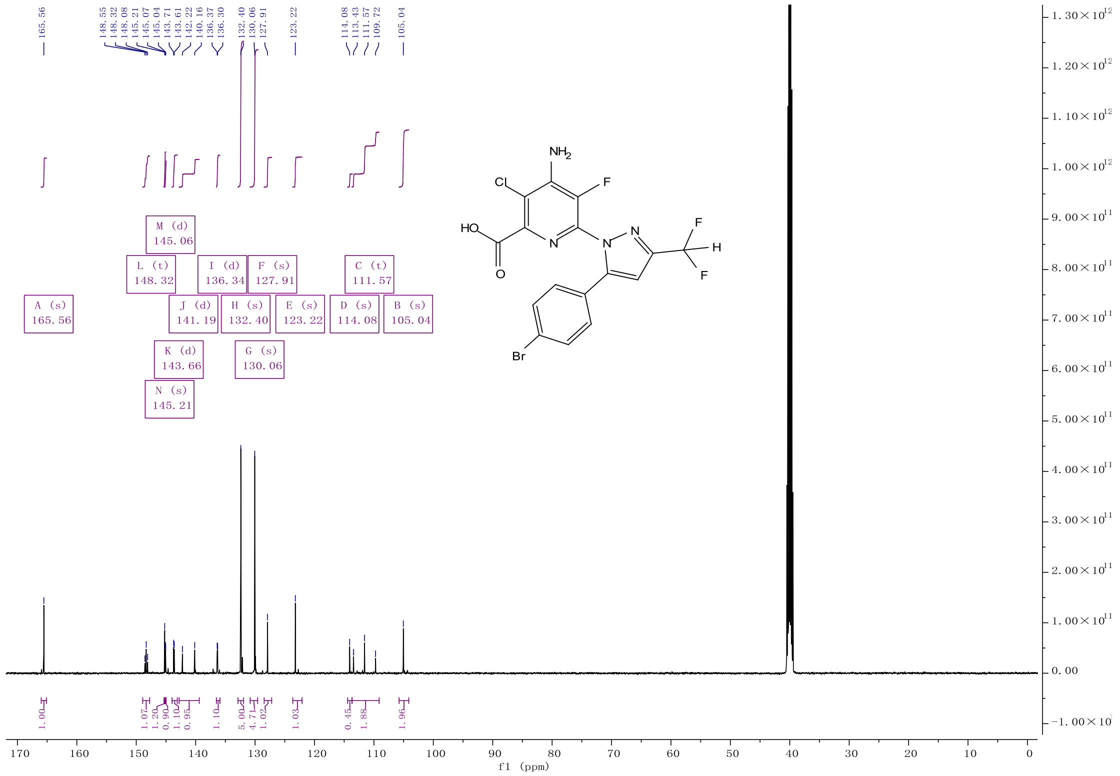

Figure S14. ^{13}C NMR (125.77 MHz, DMSO-d_6) spectrum of compound S152.

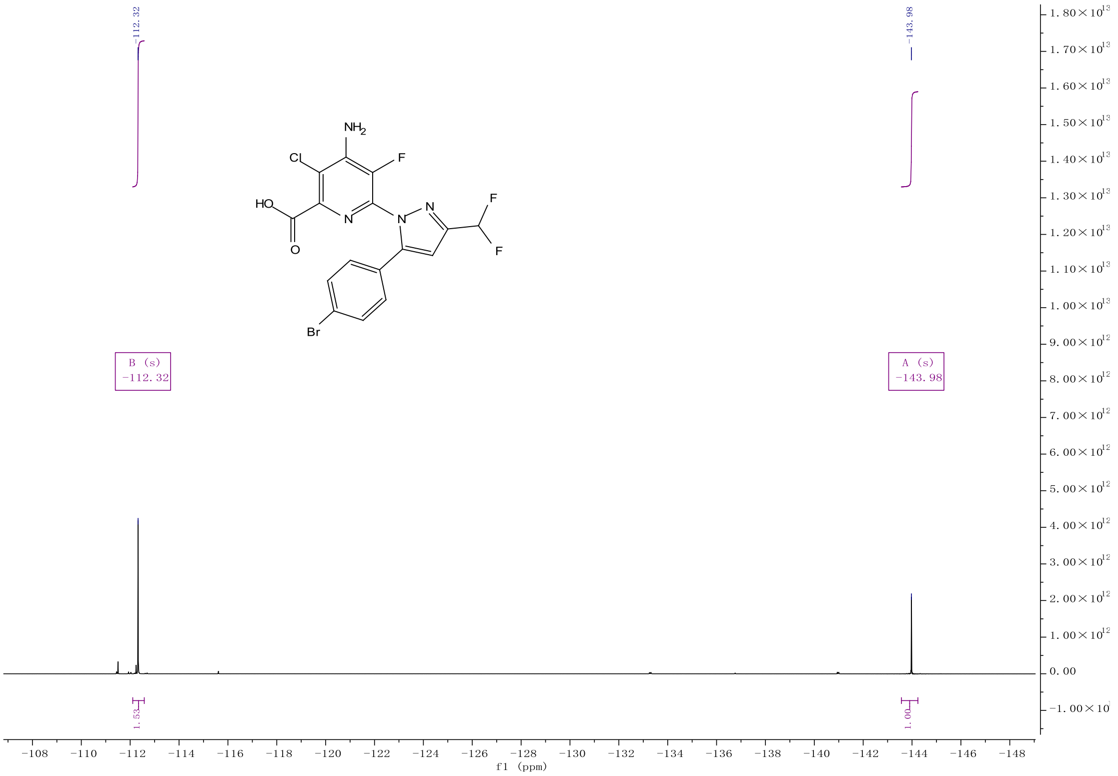

Figure S15. ^{19}F NMR (470.54 MHz, DMSO-d6) spectrum of compound S152.

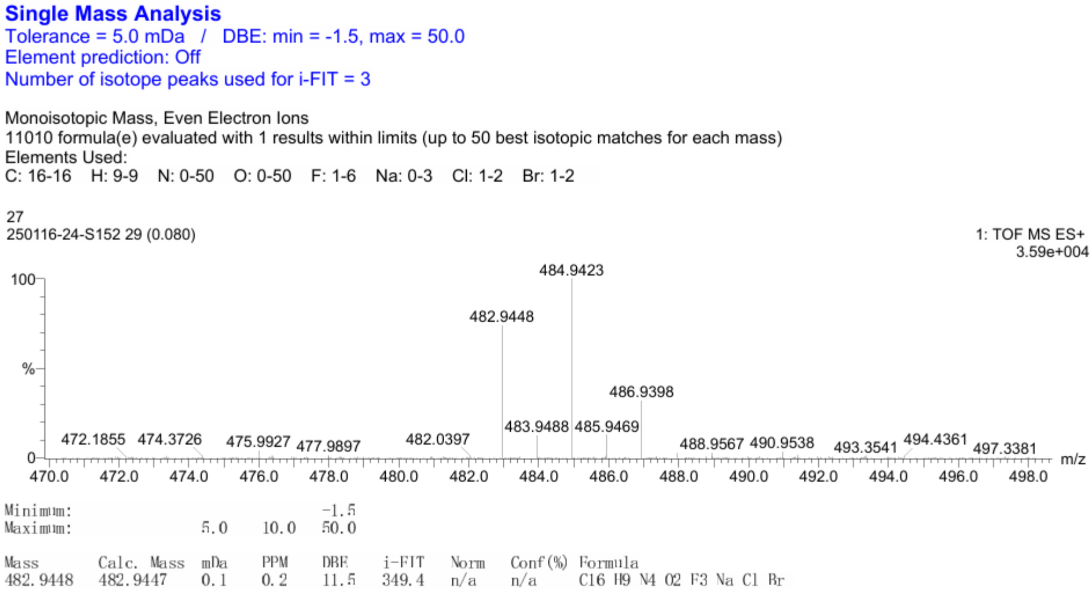

Figure S16. HRMS spectrum of compound S152.

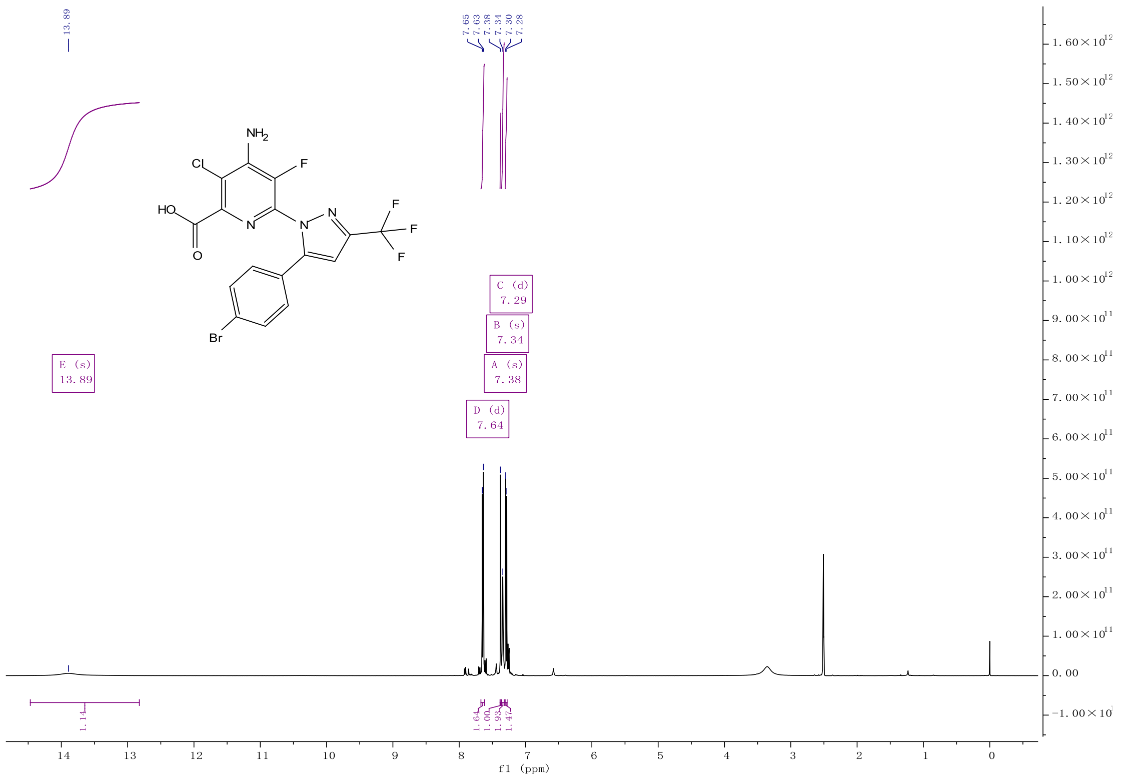

Figure S17. ^{1}H NMR (500.13 MHz, DMSO-d6) spectrum of compound S153.

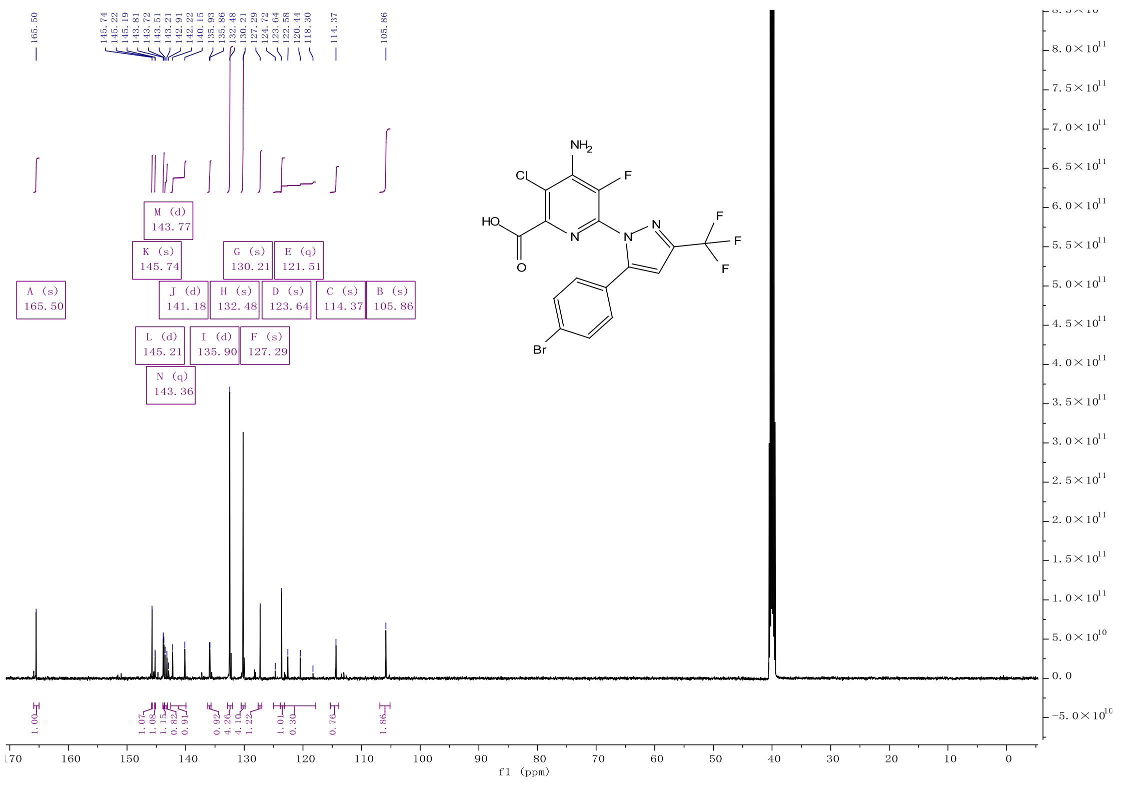

Figure S18. ^{13}C NMR (125.77 MHz, DMSO-d_6) spectrum of compound S153.

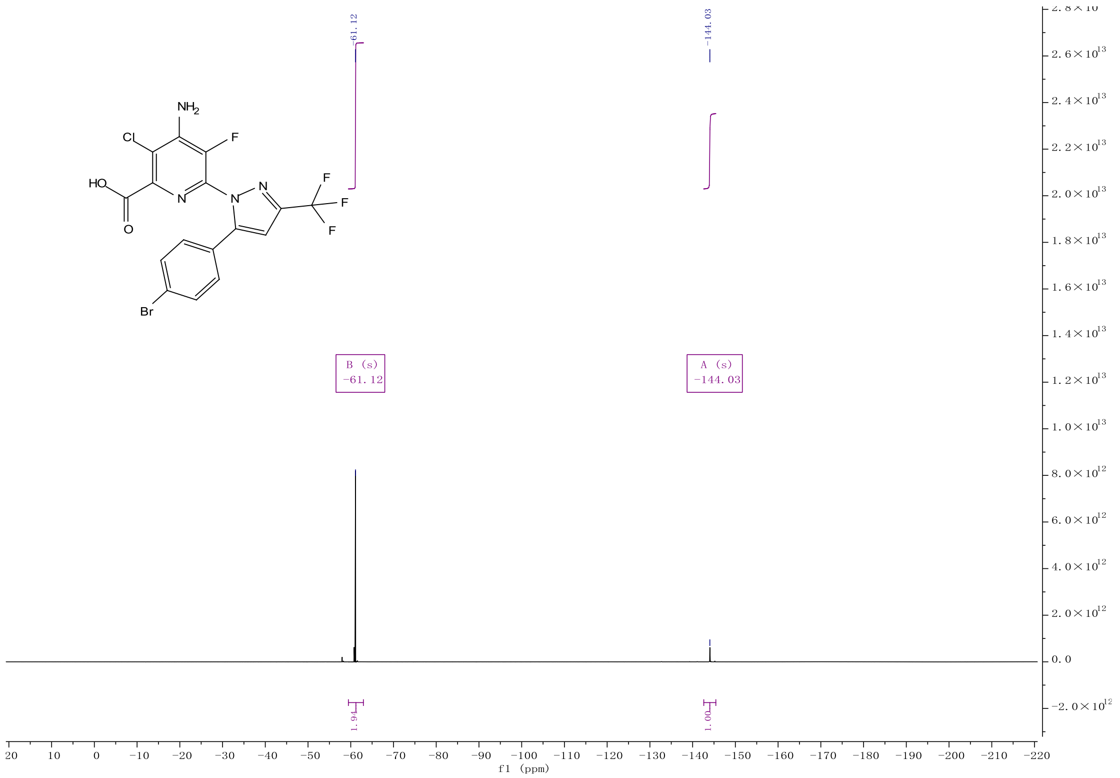

Figure S19. ^{19}F NMR (470.54 MHz, DMSO-d6) spectrum of compound S153.

Single Mass Analysis
Tolerance = 5.0 mDa / DBE: min = -1.5, max = 50.0
Element prediction: Off
Number of isotope peaks used for i-FIT = 3

Monoisotopic Mass, Even Electron Ions
9489 formula(e) evaluated with 1 results within limits (up to 50 best isotopic matches for each mass)
Elements Used:
C: 16-16 H: 8-8 N: 0-50 O: 0-50 F: 1-4 Na: 0-3 Cl: 1-2 Br: 1-2

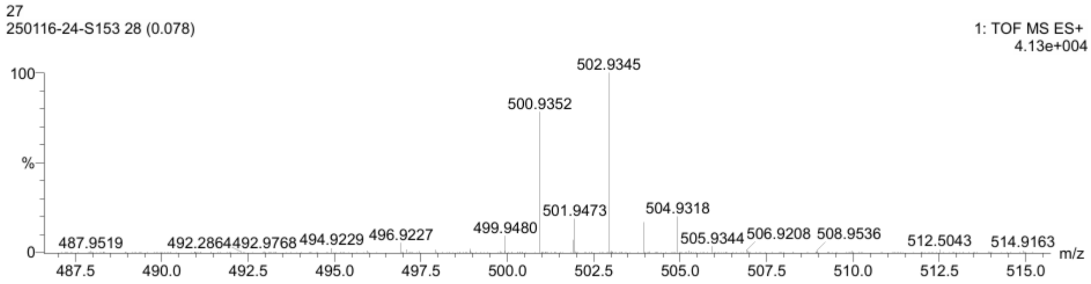

Minimum:				-1.5				
Maximum:		5.0	10.0	50.0				
Mass	Calc. Mass	mDa	PPM	DBE	i-FIT	Norm	Conf(%)	Formula
500.9352	500.9353	-0.1	-0.2	11.5	346.3	n/a	n/a	C16 H8 N4 O2 F4 Na Cl Br

Figure S20. HRMS spectrum of compound S153.

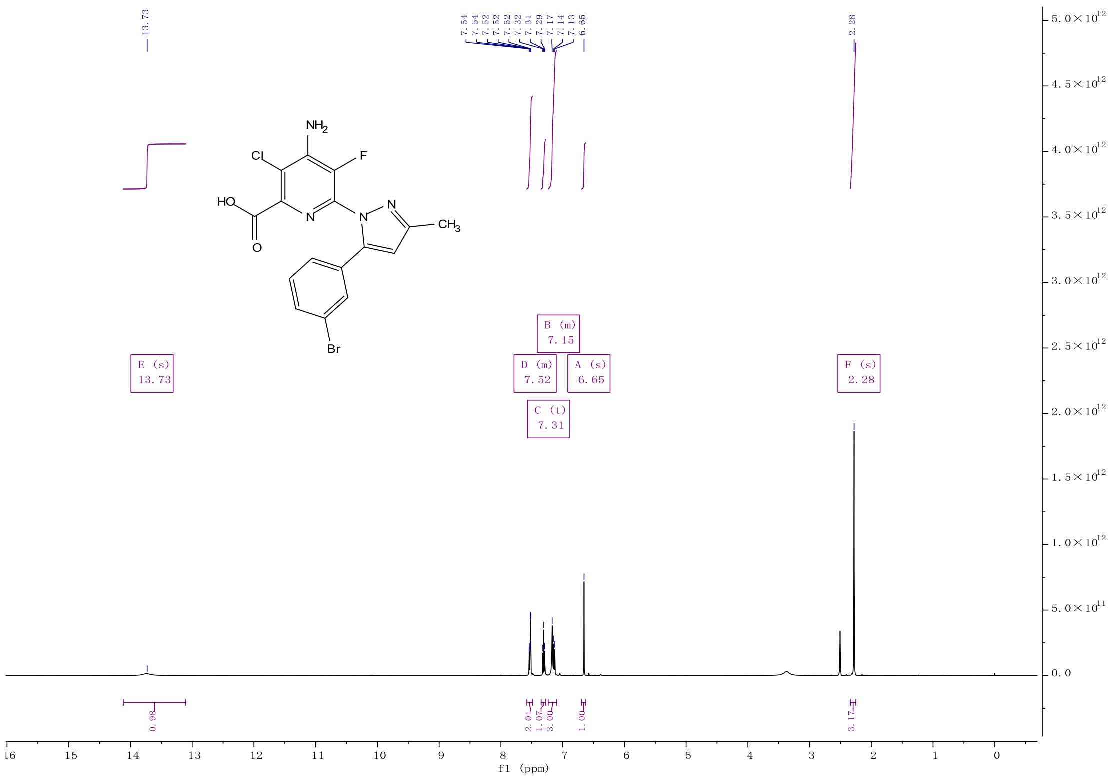

Figure S21. ^{1}H NMR (500.13 MHz, DMSO-d6) spectrum of compound S160.

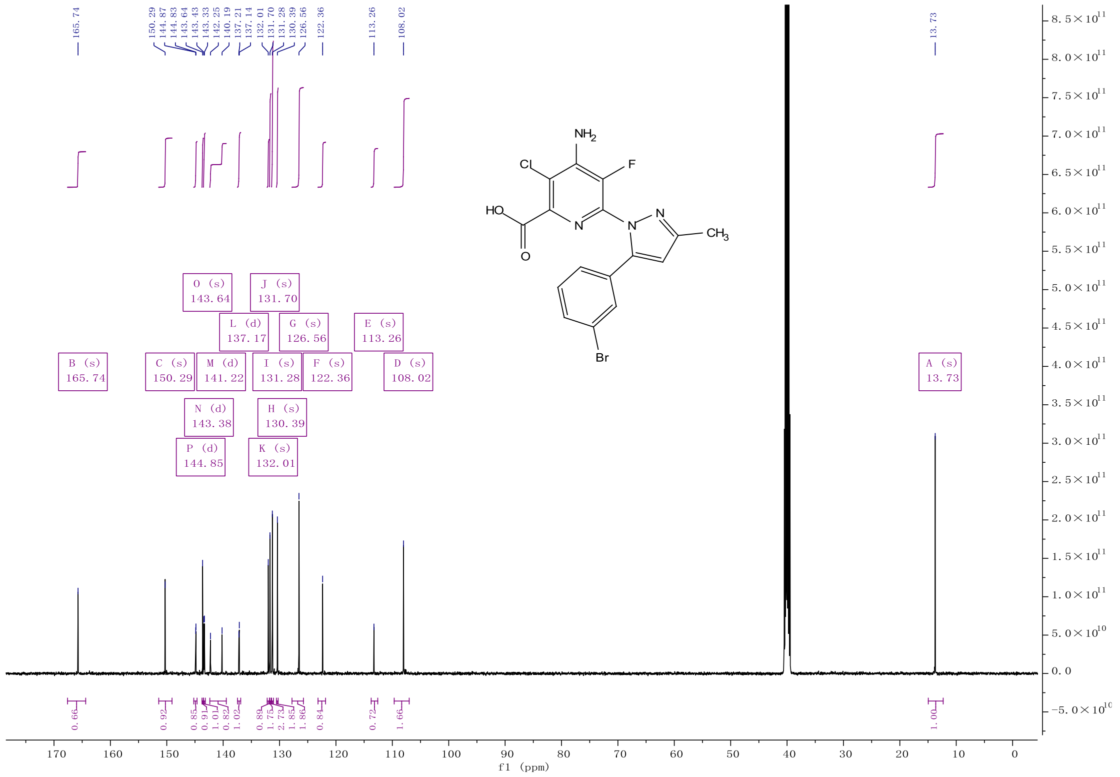

Figure S22. ^{13}C NMR (125.77 MHz, DMSO-d_6) spectrum of compound S160.

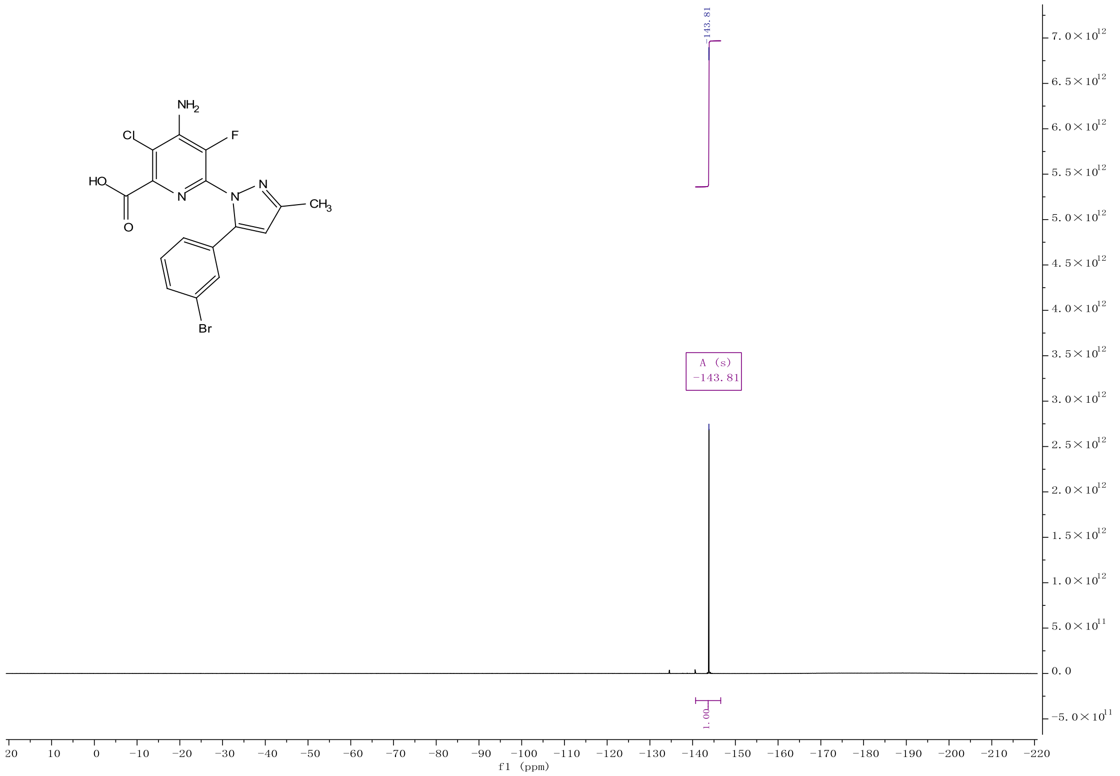

Figure S23. ^{19}F NMR (470.54 MHz, DMSO-d6) spectrum of compound S160.

Single Mass Analysis
Tolerance = 5.0 mDa / DBE: min = -1.5, max = 50.0
Element prediction: Off
Number of isotope peaks used for i-FIT = 3

Monoisotopic Mass, Even Electron Ions
1666 formula(e) evaluated with 1 results within limits (up to 50 best isotopic matches for each mass)
Elements Used:
C: 16-16 H: 12-12 N: 0-50 O: 0-50 F: 1-1 Na: 0-3 Cl: 1-2 Br: 1-2

4
250116-24-S160 20 (0.063) 1: TOF MS ES+ 7.50e+004

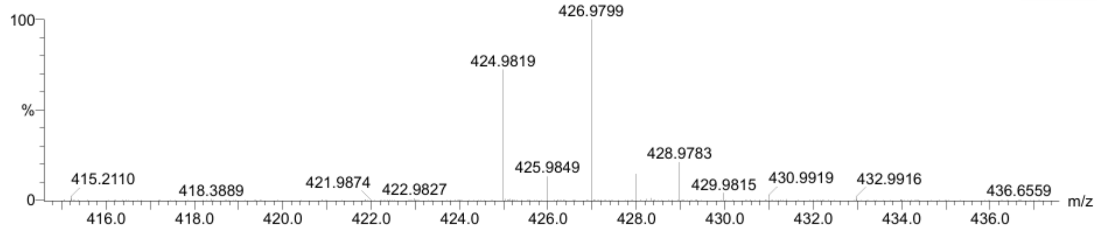

Minimum:				-1.5				
Maximum:		5.0	10.0	50.0				
Mass	Calc. Mass	mDa	PPM	DBE	i-FIT	Norm	Conf(%)	Formula
424.9819	424.9816	0.3	0.7	11.5	346.3	n/a	n/a	C16 H12 N4 O2 F Cl Br

Figure S24. HRMS spectrum of compound S160.

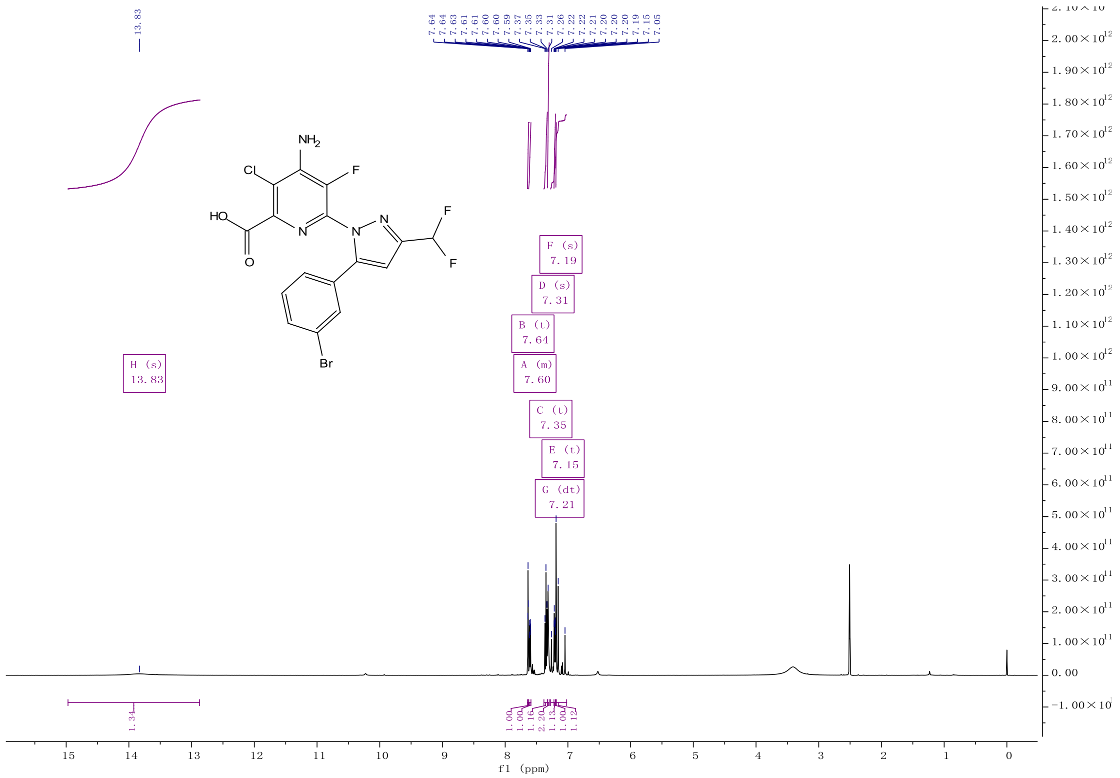

Figure S25. ^{1}H NMR (500.13 MHz, DMSO-d6) spectrum of compound S162.

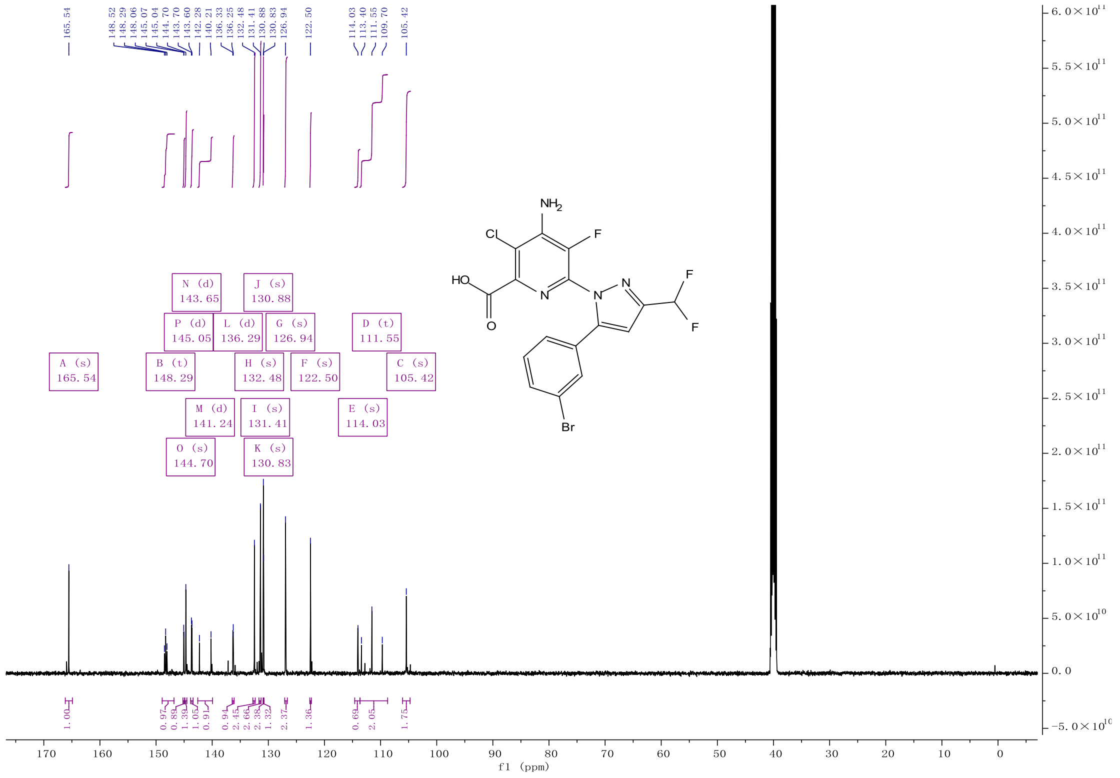

Figure S26. ^{13}C NMR (125.77 MHz, DMSO-d_6) spectrum of compound S162.

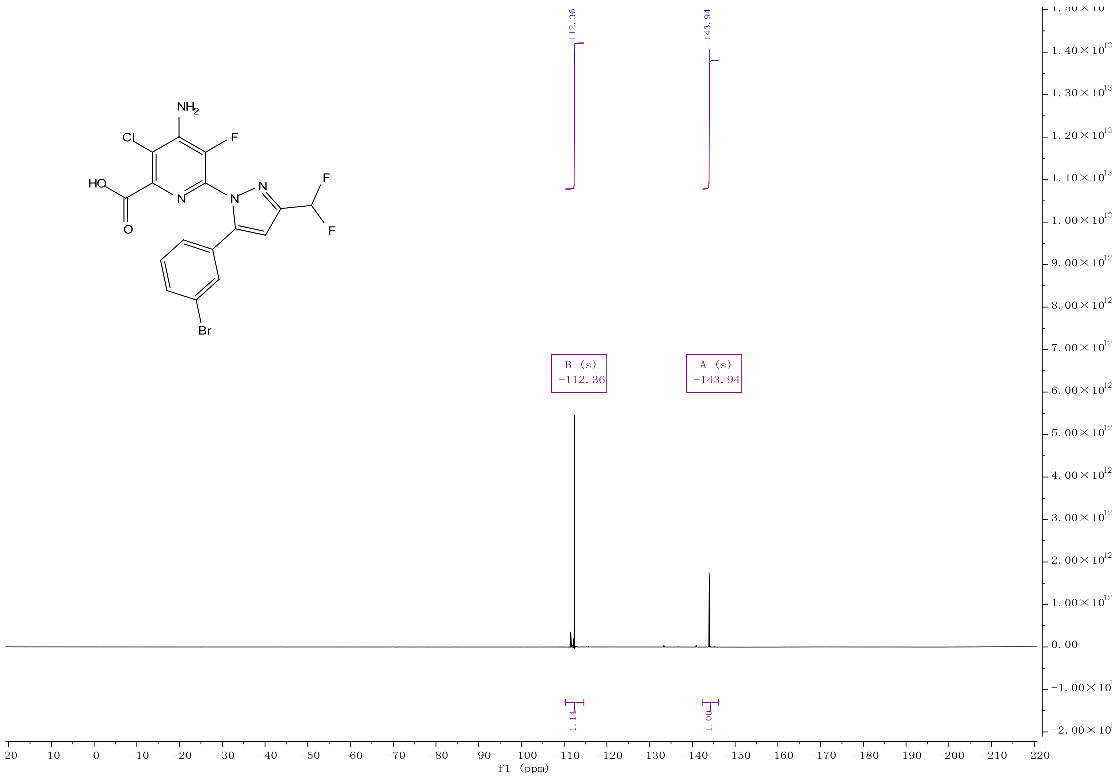

Figure S27. ^{19}F NMR (470.54 MHz, DMSO-d6) spectrum of compound S162.

Single Mass Analysis
Tolerance = 5.0 mDa / DBE: min = -1.5, max = 50.0
Element prediction: Off
Number of isotope peaks used for i-FIT = 3

Monoisotopic Mass, Even Electron Ions
1948 formula(e) evaluated with 1 results within limits (up to 50 best isotopic matches for each mass)
Elements Used:
C: 16-16 H: 9-9 N: 0-50 O: 0-50 F: 3-3 Na: 0-3 Cl: 1-2 Br: 1-2

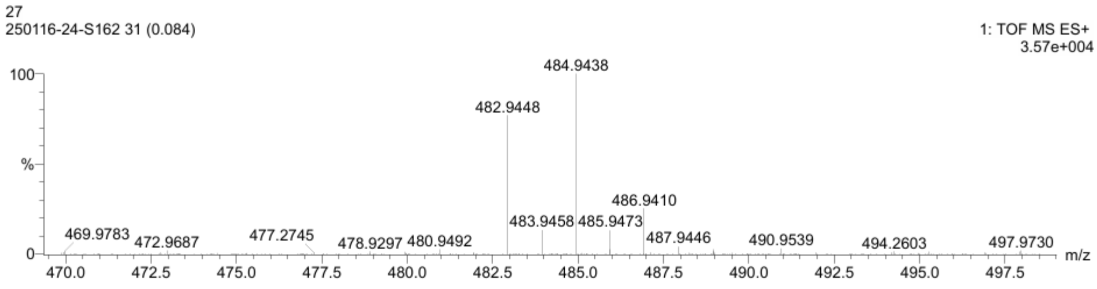

Minimum:				-1.5				
Maximum:		5.0	10.0	50.0				
Mass	Calc. Mass	mDa	PPM	DBE	i-FIT	Norm	Conf(%)	Formula
482.9448	482.9447	0.1	0.2	11.5	300.4	n/a	n/a	C16 H9 N4 O2 F3 Na Cl Br

Figure S28. HRMS spectrum of compound S162.

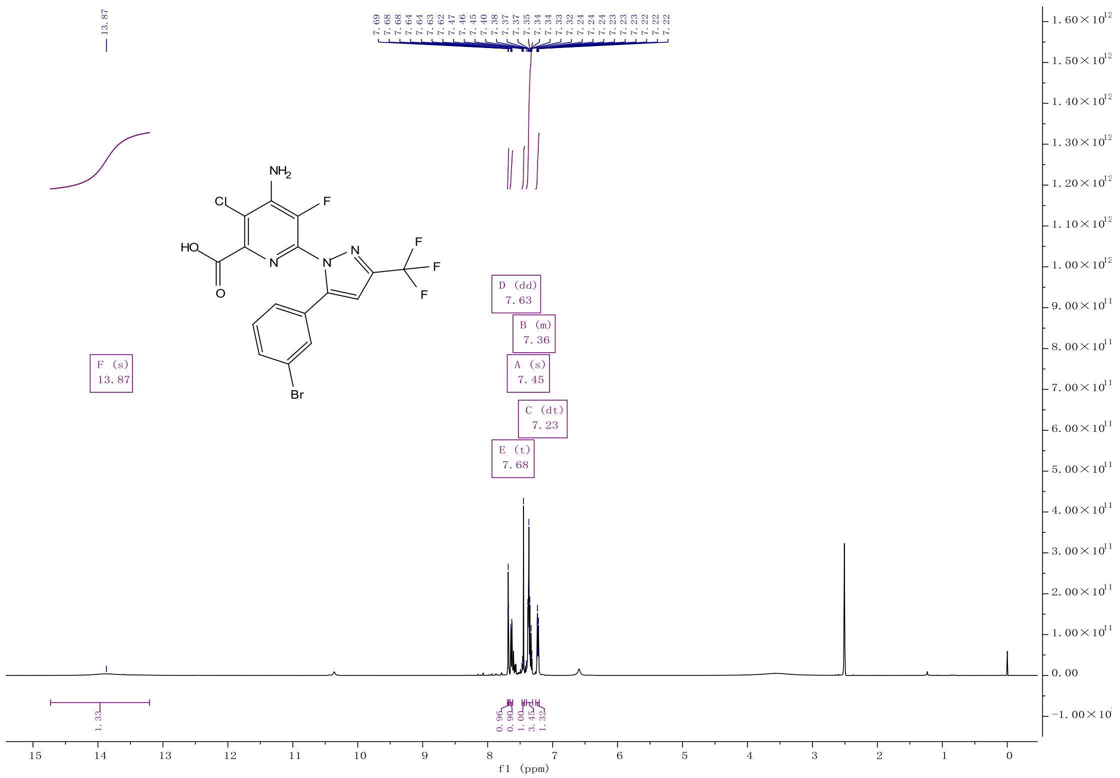

Figure S29. ^{1}H NMR (500.13 MHz, DMSO-d6) spectrum of compound S163.

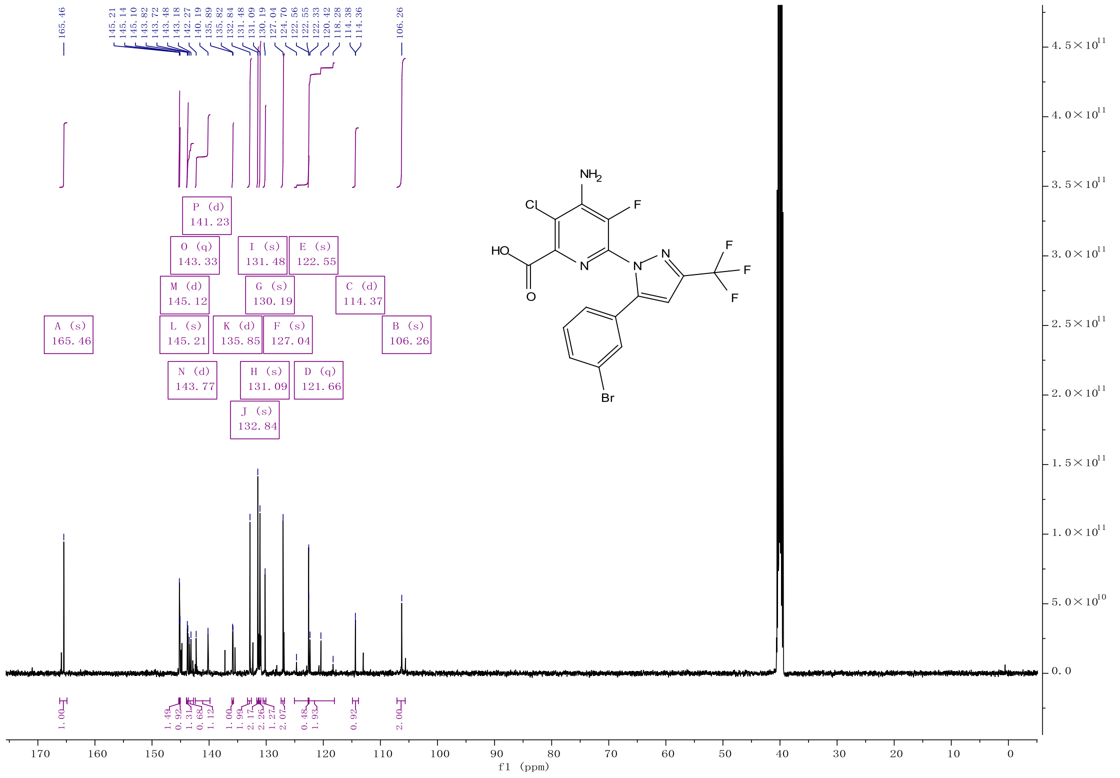

Figure S30. ^{13}C NMR (125.77 MHz, DMSO-d_6) spectrum of compound S163.

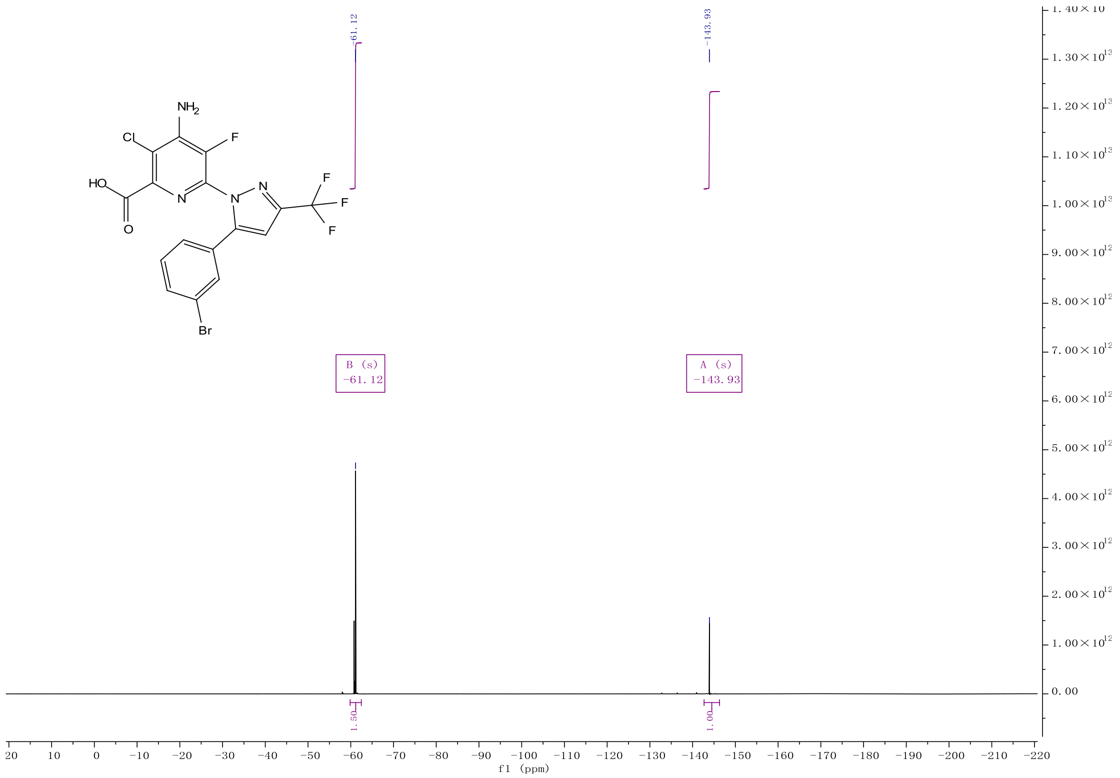

Figure S31. ^{19}F NMR (470.54 MHz, DMSO-d6) spectrum of compound S163.

Single Mass Analysis
Tolerance = 5.0 mDa / DBE: min = -1.5, max = 50.0
Element prediction: Off
Number of isotope peaks used for i-FIT = 3

Monoisotopic Mass, Even Electron Ions
5815 formula(e) evaluated with 1 results within limits (up to 50 best isotopic matches for each mass)
Elements Used:
C: 16-16 H: 8-8 N: 0-50 O: 0-50 F: 3-5 Na: 0-3 Cl: 1-2 Br: 1-2

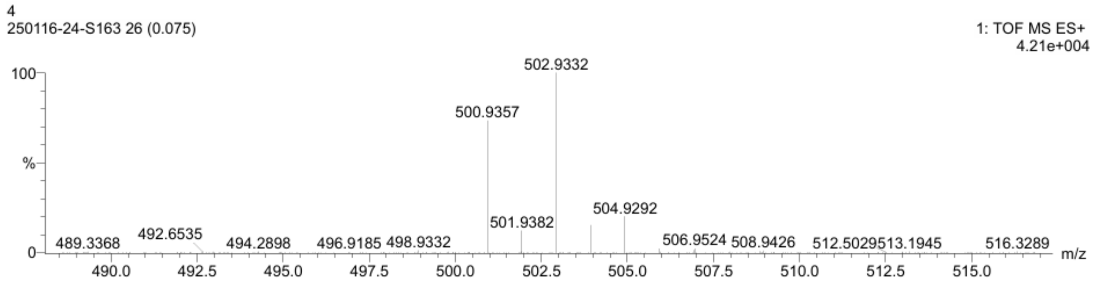

Minimum:				-1.5				
Maximum:		5.0	10.0	50.0				
Mass	Calc. Mass	mDa	PPM	DBE	i-FIT	Norm	Conf(%)	Formula
500.9357	500.9353	0.4	0.8	11.5	231.8	n/a	n/a	C16 H8 N4 O2 F4 Na Cl Br

Figure S32. HRMS spectrum of compound S163.

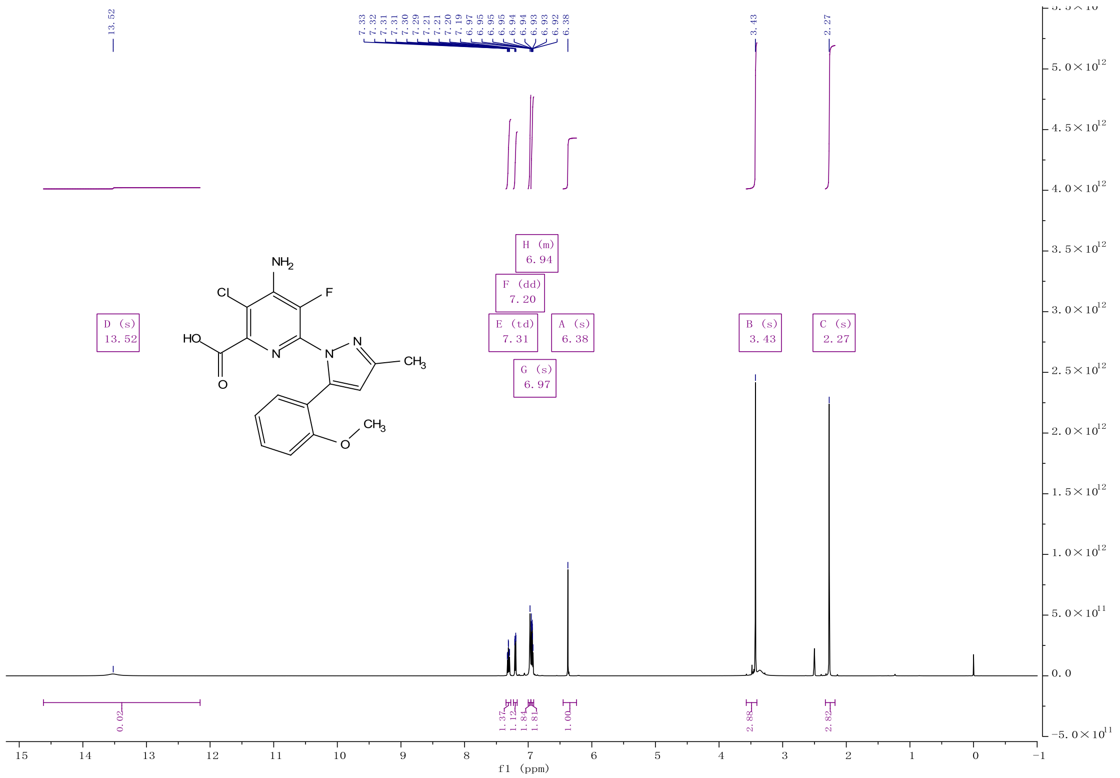

Figure S33. ^{1}H NMR (500.13 MHz, DMSO-d6) spectrum of compound S200.

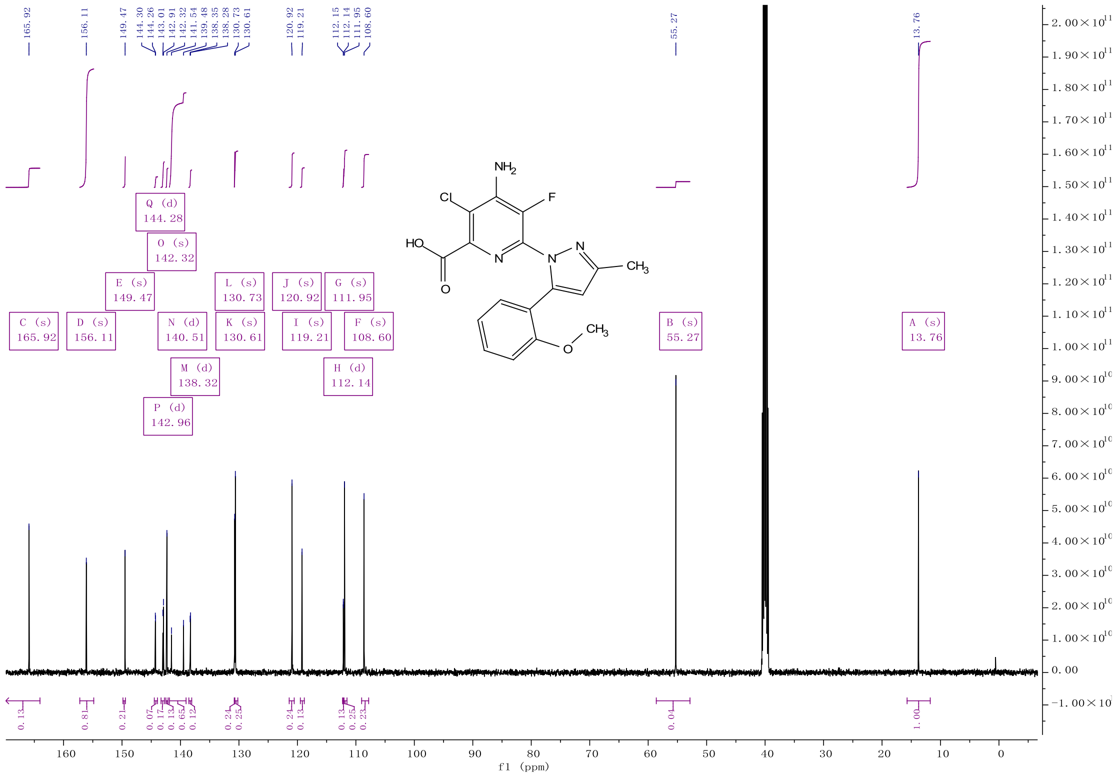

Figure S34. ^{13}C NMR (125.77 MHz, DMSO-d_6) spectrum of compound S200.

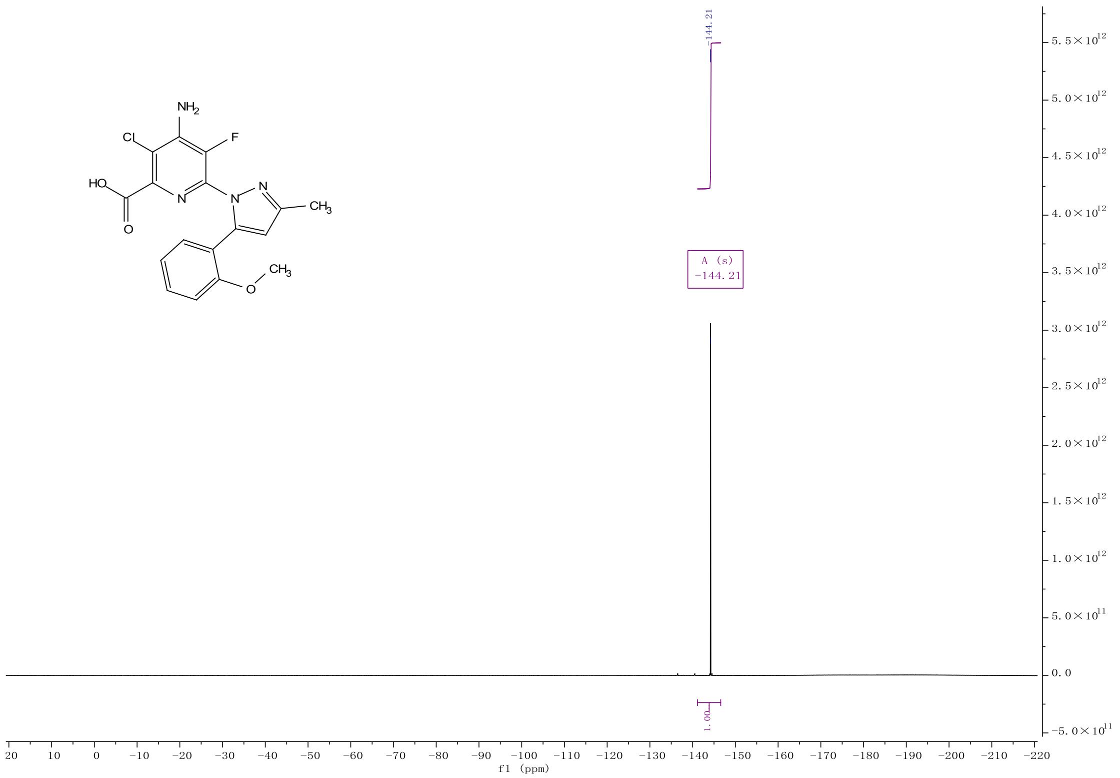

Figure S35. ^{19}F NMR (470.54 MHz, DMSO-d6) spectrum of compound S200.

Single Mass Analysis
Tolerance = 5.0 mDa / DBE: min = -1.5, max = 50.0
Element prediction: Off
Number of isotope peaks used for i-FIT = 3

Monoisotopic Mass, Even Electron Ions
4547 formula(e) evaluated with 1 results within limits (up to 50 best isotopic matches for each mass)
Elements Used:
C: 17-17 H: 15-15 N: 0-50 O: 0-50 F: 1-4 Na: 0-3 Cl: 1-2
28
250116-24-S200 37 (0.095)

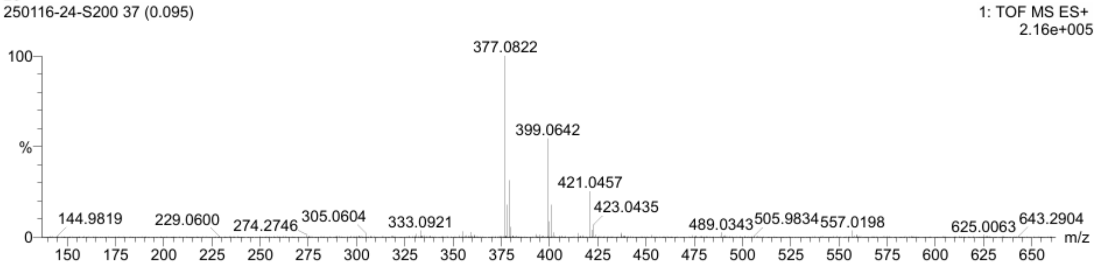

Minimum:				-1.5				
Maximum:		5.0	10.0	50.0				
Mass	Calc. Mass	mDa	PPM	DBE	i-FIT	Norm	Conf(%)	Formula
377.0822	377.0817	0.5	1.3	11.5	447.6	n/a	n/a	C17 H15 N4 O3 F Cl

Figure S36. HRMS spectrum of compound S200.

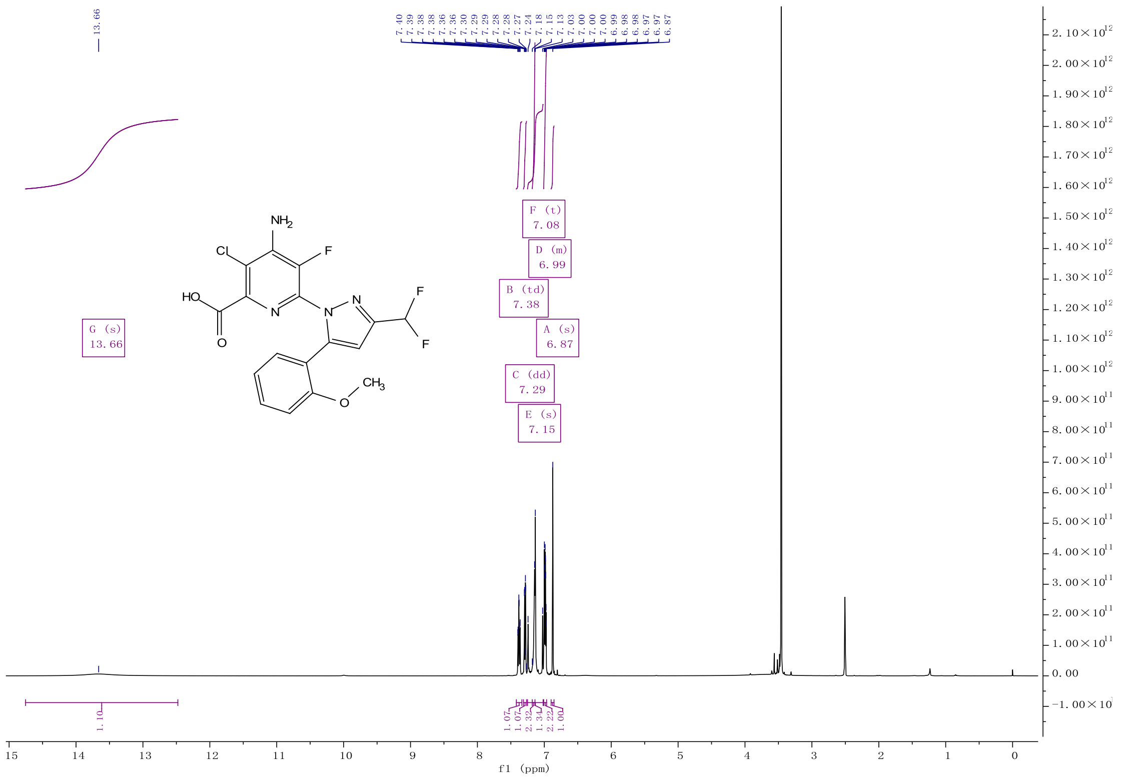

Figure S37. ^{1}H NMR (500.13 MHz, DMSO-d6) spectrum of compound S202.

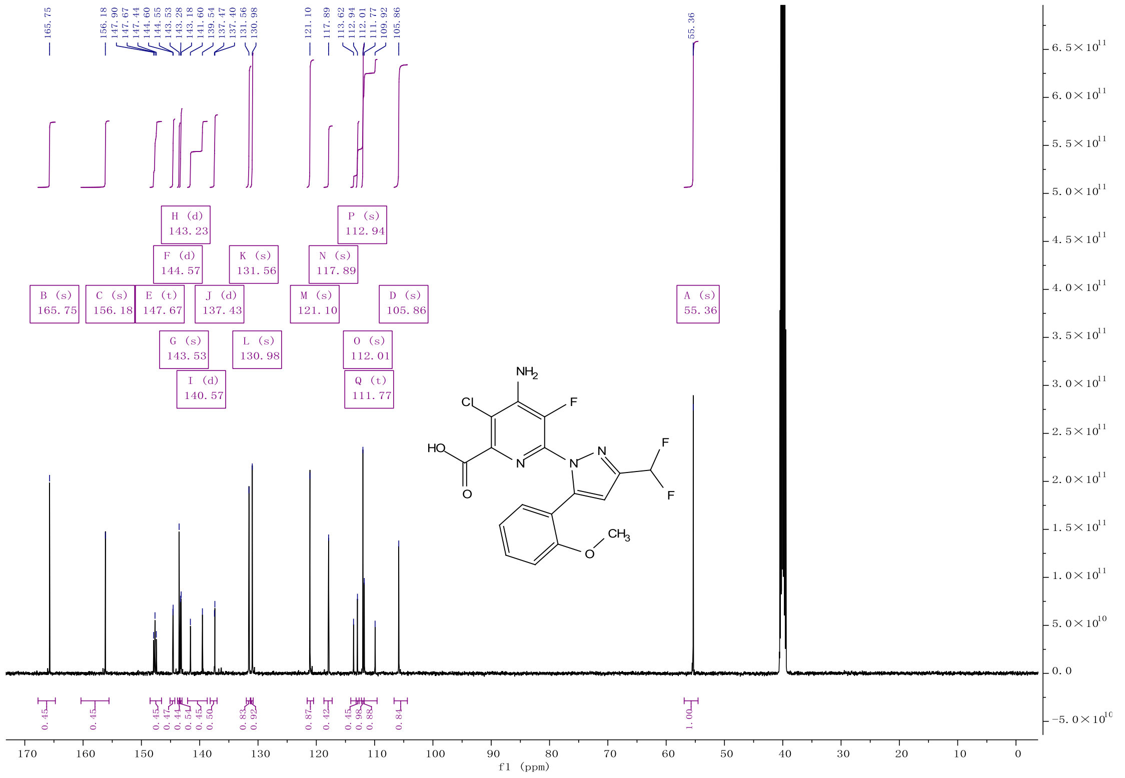

Figure S38. ^{13}C NMR (125.77 MHz, DMSO-d_6) spectrum of compound S202.

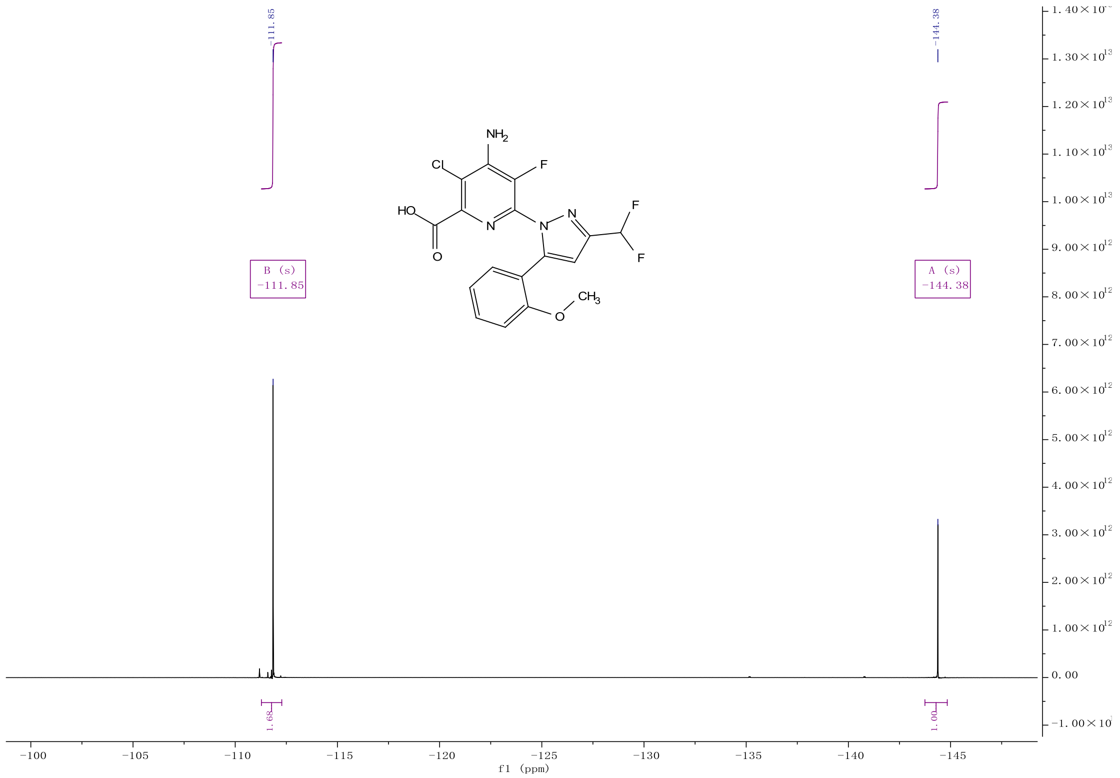

Figure S39. ^{19}F NMR (470.54 MHz, DMSO-d6) spectrum of compound S202.

Single Mass Analysis
Tolerance = 5.0 mDa / DBE: min = -1.5, max = 50.0
Element prediction: Off
Number of isotope peaks used for i-FIT = 3

Monoisotopic Mass, Even Electron Ions
2897 formula(e) evaluated with 1 results within limits (up to 50 best isotopic matches for each mass)
Elements Used:
C: 17-17 H: 12-12 N: 0-50 O: 0-50 F: 3-4 Na: 0-3 Cl: 1-2
27
250116-24-S202 33 (0.088)

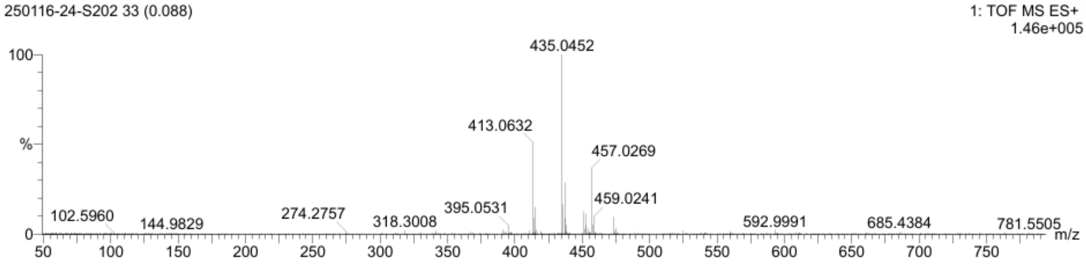

Minimum:				-1.5				
Maximum:		5.0	10.0	50.0				
Mass	Calc. Mass	mDa	PPM	DBE	i-FIT	Norm	Conf(%)	Formula
435.0452	435.0448	0.4	0.9	11.5	414.5	n/a	n/a	C17 H12 N4 O3 F3 Na Cl

Figure S40. HRMS spectrum of compound S202.

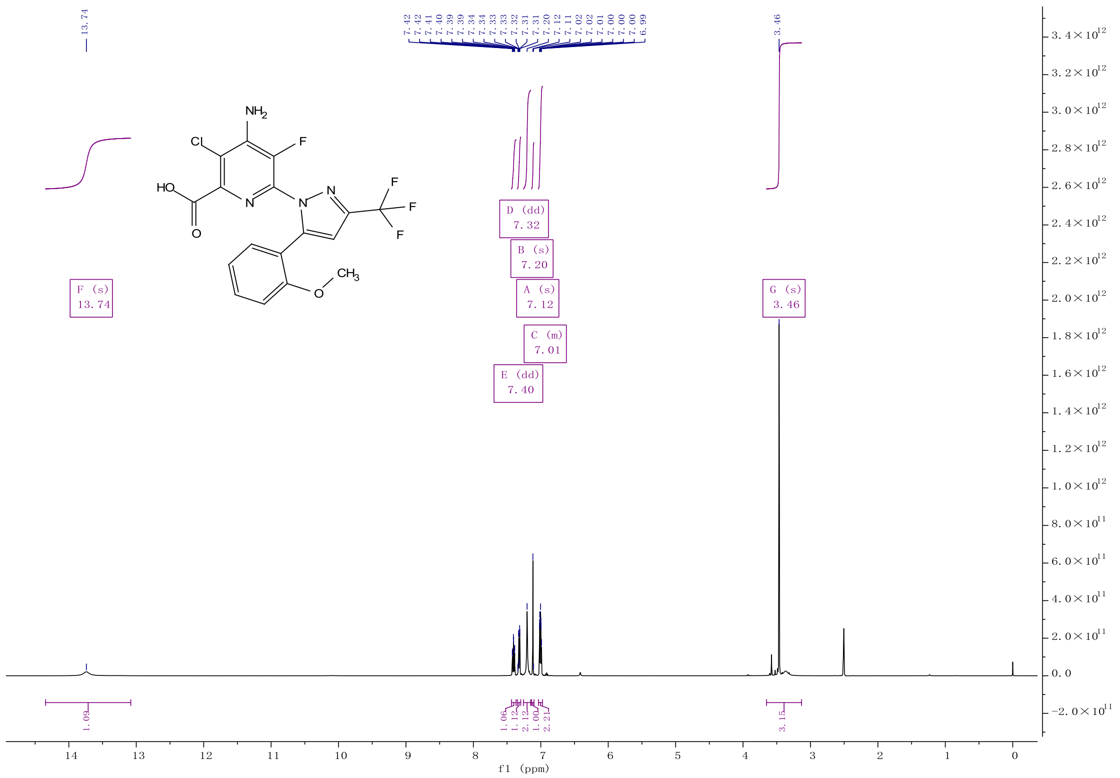

Figure S41. ^{1}H NMR (500.13 MHz, DMSO-d6) spectrum of compound S203.

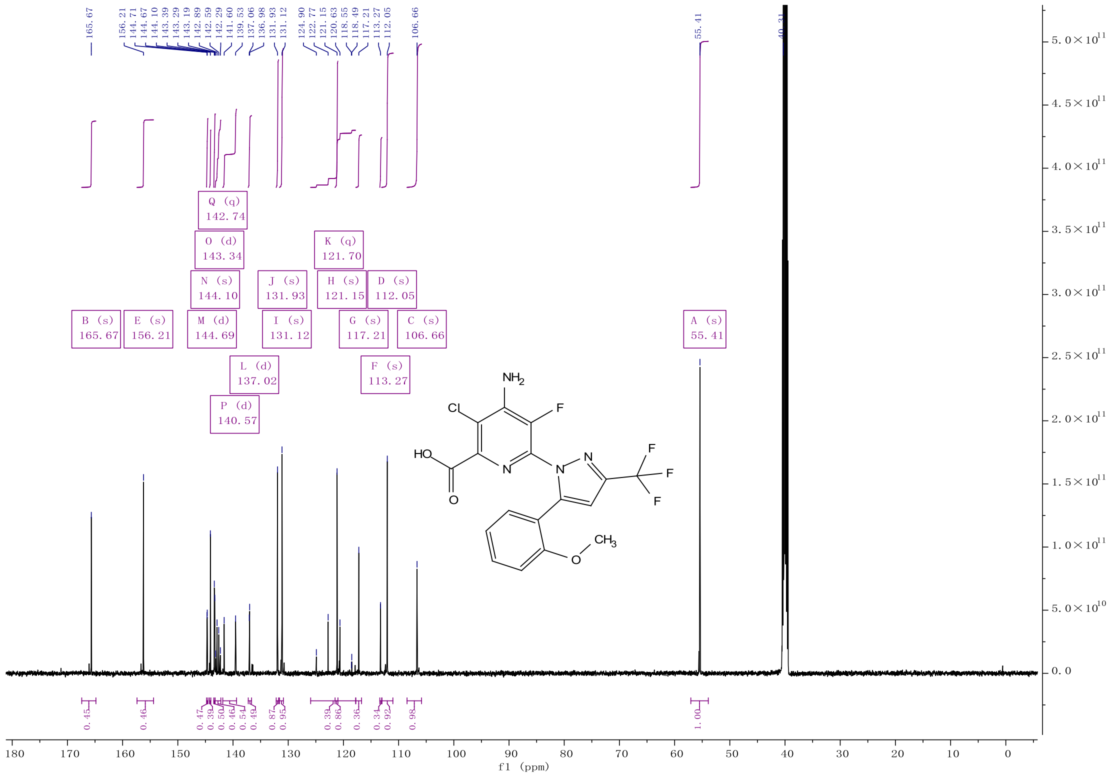

Figure S42. ^{13}C NMR (125.77 MHz, DMSO-d_6) spectrum of compound S203.

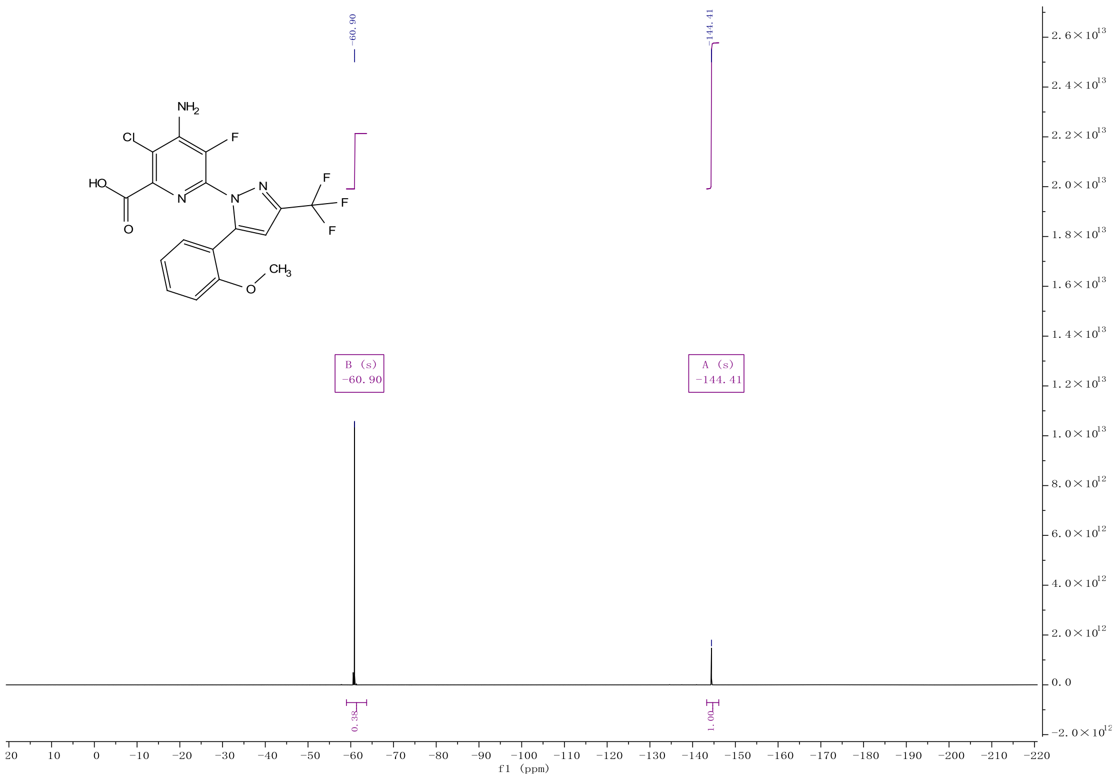

Figure S43. ^{19}F NMR (470.54 MHz, DMSO-d6) spectrum of compound S203.

Single Mass Analysis
Tolerance = 5.0 mDa / DBE: min = -1.5, max = 50.0
Element prediction: Off
Number of isotope peaks used for i-FIT = 3

Monoisotopic Mass, Even Electron Ions
5151 formula(e) evaluated with 1 results within limits (up to 50 best isotopic matches for each mass)
Elements Used:
C: 17-17 H: 11-11 N: 0-50 O: 0-50 F: 4-7 Na: 0-3 Cl: 1-2
27
250116-24-S203 26 (0.075) 1: TOF MS ES+ 2.03e+005

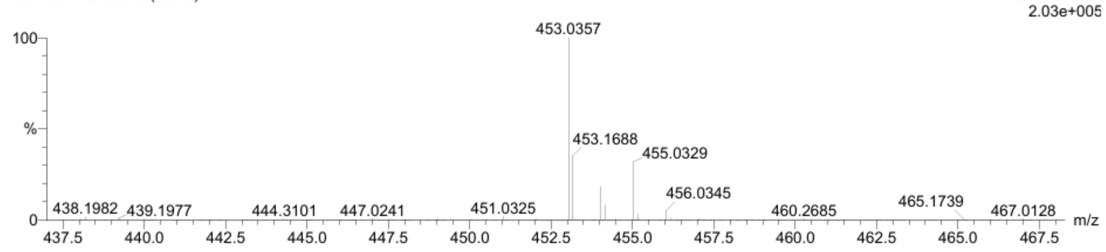

Minimum:				-1.5				
Maximum:		5.0	10.0	50.0				
Mass	Calc. Mass	mDa	PPM	DBE	i-FIT	Norm	Conf(%)	Formula
453.0357	453.0354	0.3	0.7	11.5	468.5	n/a	n/a	C17 H11 N4 O3 F4 Na Cl

Figure S44. HRMS spectrum of compound S203.

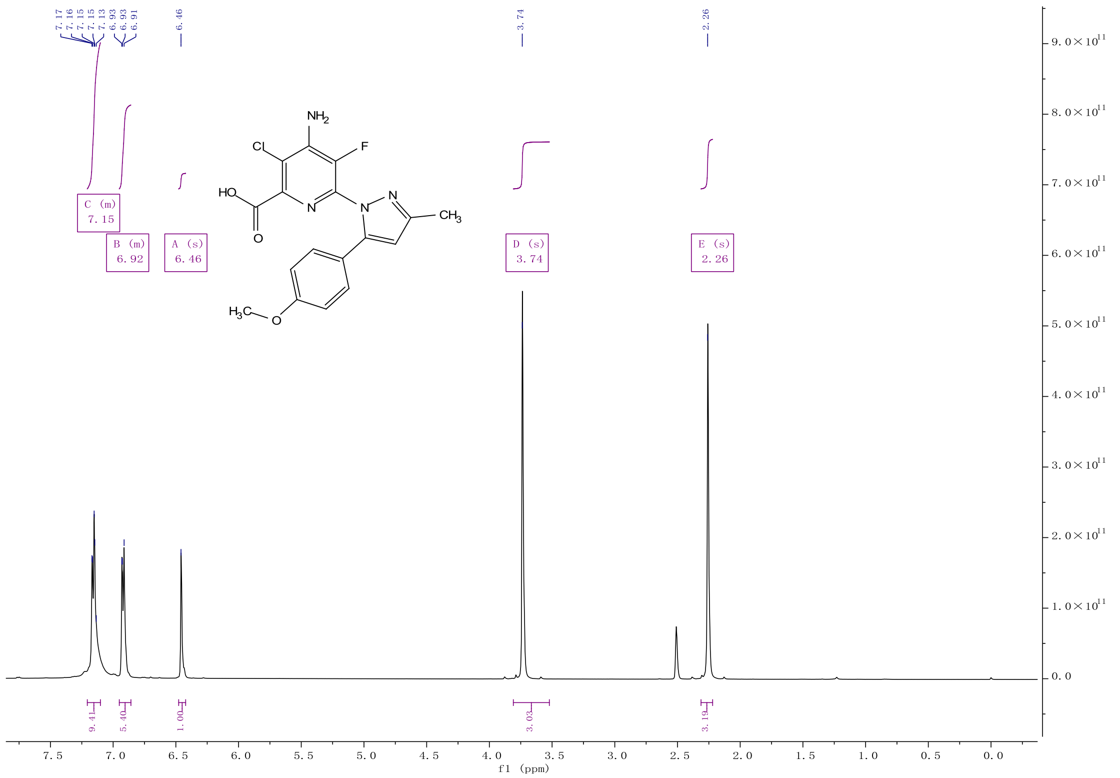

Figure S45. ^{1}H NMR (500.13 MHz, DMSO-d6) spectrum of compound S100.

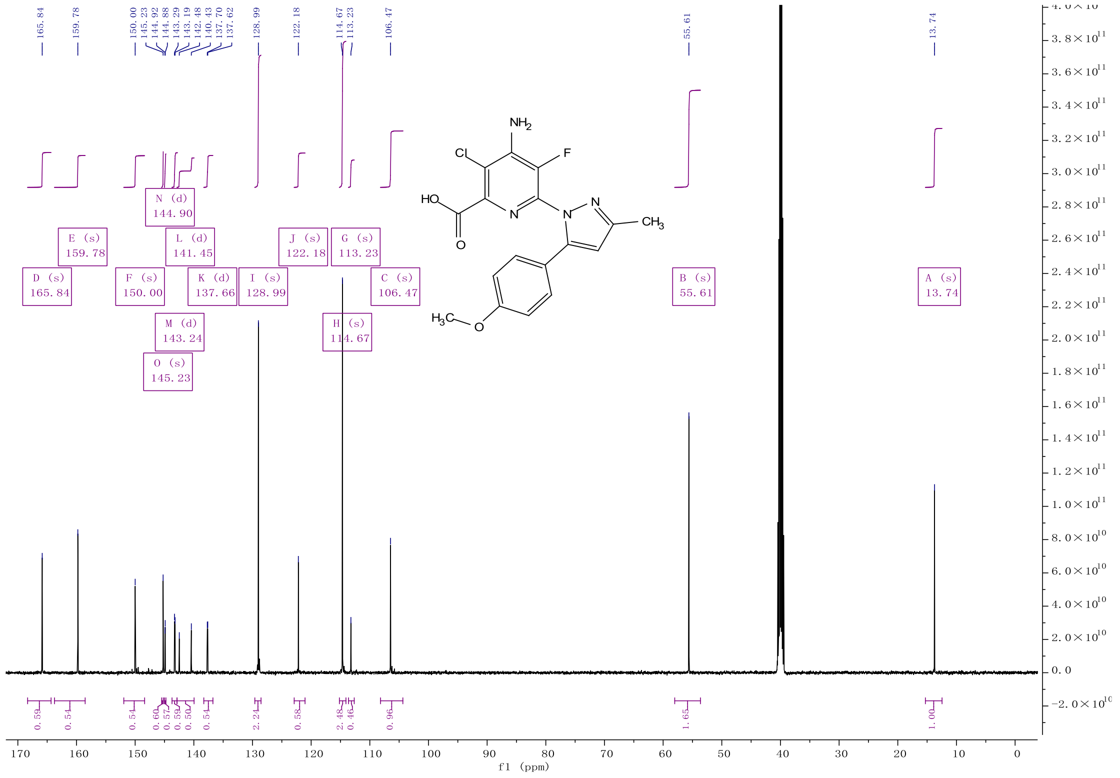

Figure S46. ^{13}C NMR (125.77 MHz, DMSO-d_6) spectrum of compound S100.

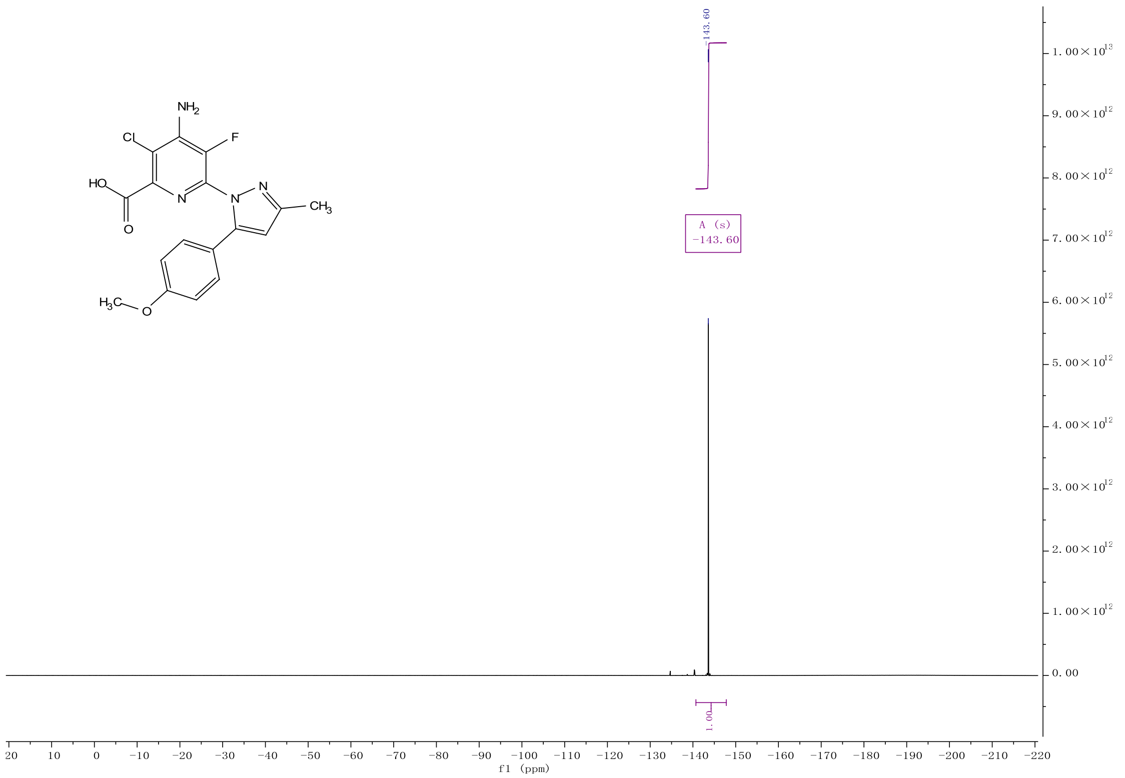

Figure S47. ^{19}F NMR (470.54 MHz, DMSO-d6) spectrum of compound S100.

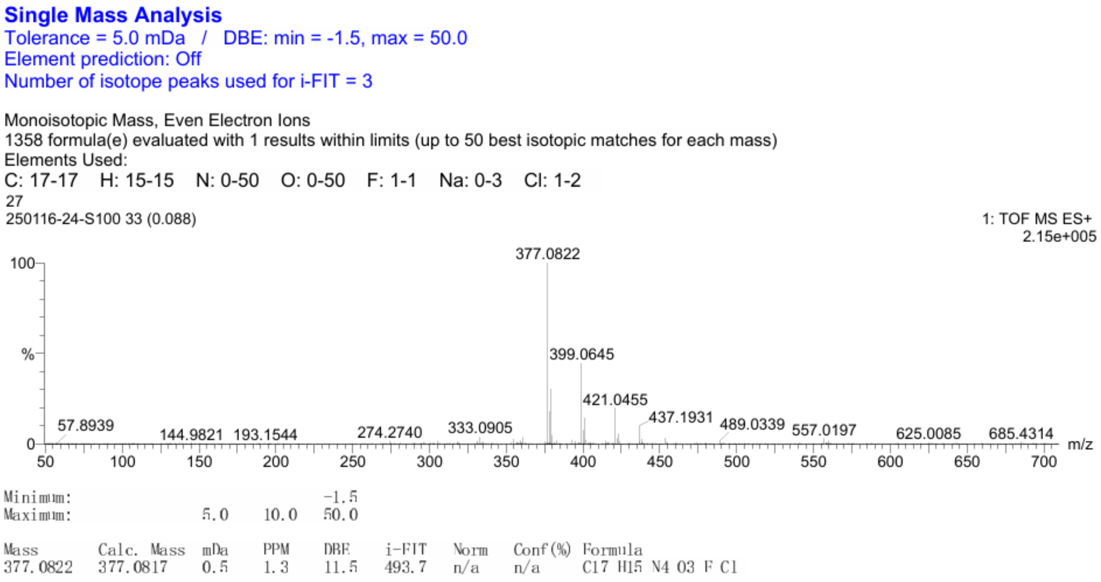

Figure S48. HRMS spectrum of compound S100.

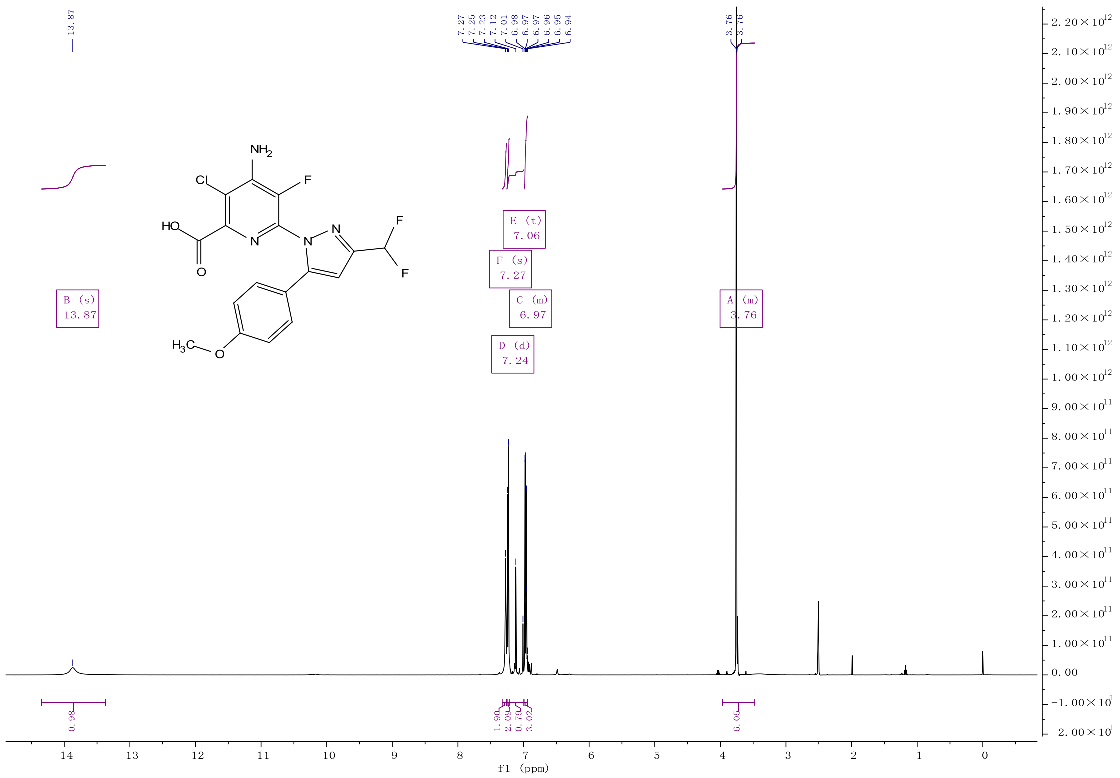

Figure S49. ^{1}H NMR (500.13 MHz, DMSO-d6) spectrum of compound S102.

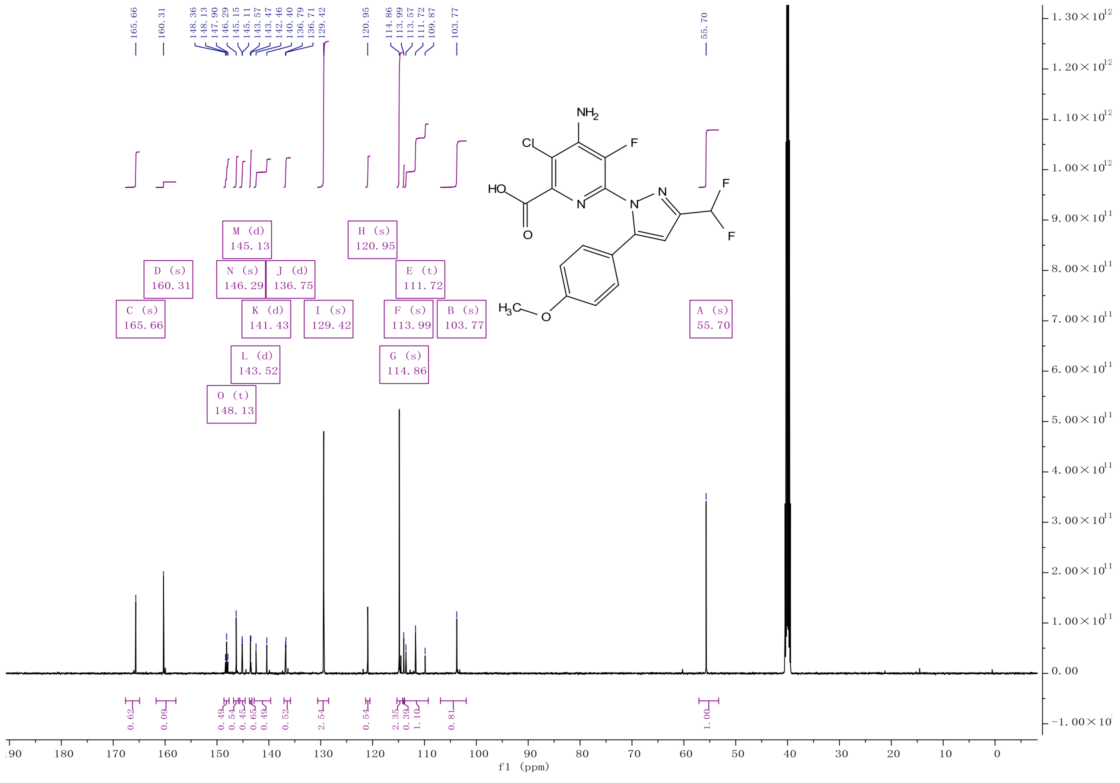

Figure S50. ^{13}C NMR (125.77 MHz, DMSO-d_6) spectrum of compound S102.

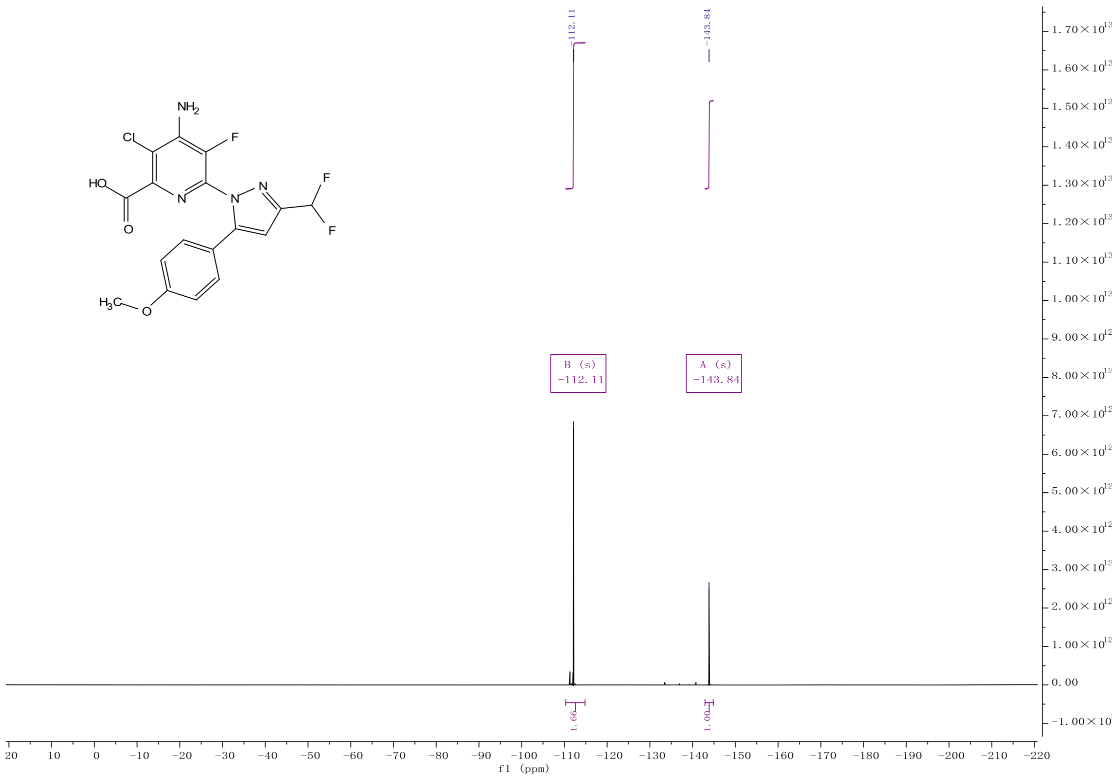

Figure S51. ^{19}F NMR (470.54 MHz, DMSO-d6) spectrum of compound S102.

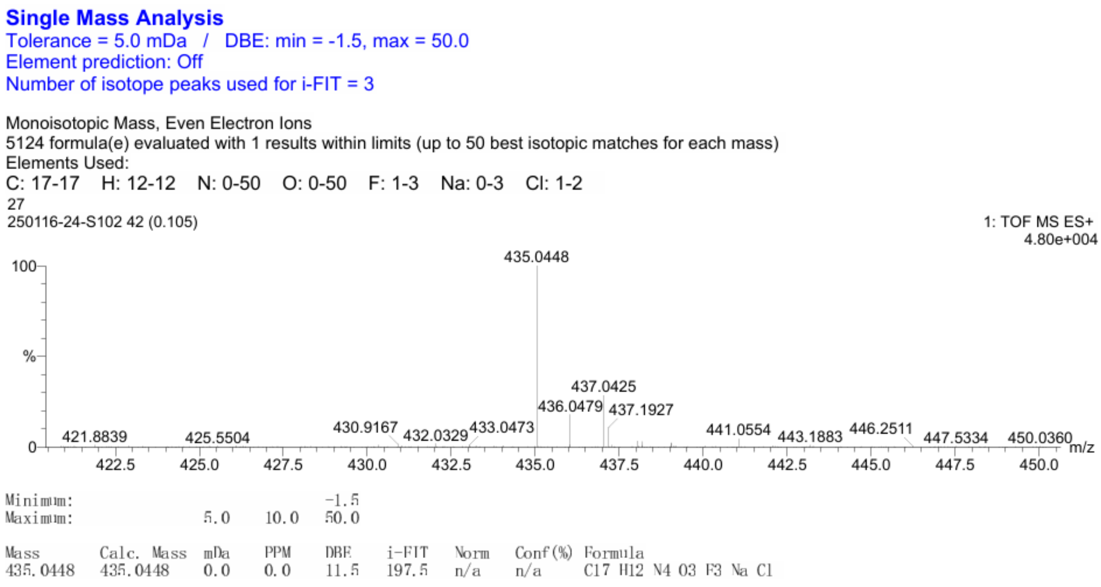

Figure S52. HRMS spectrum of compound S102.

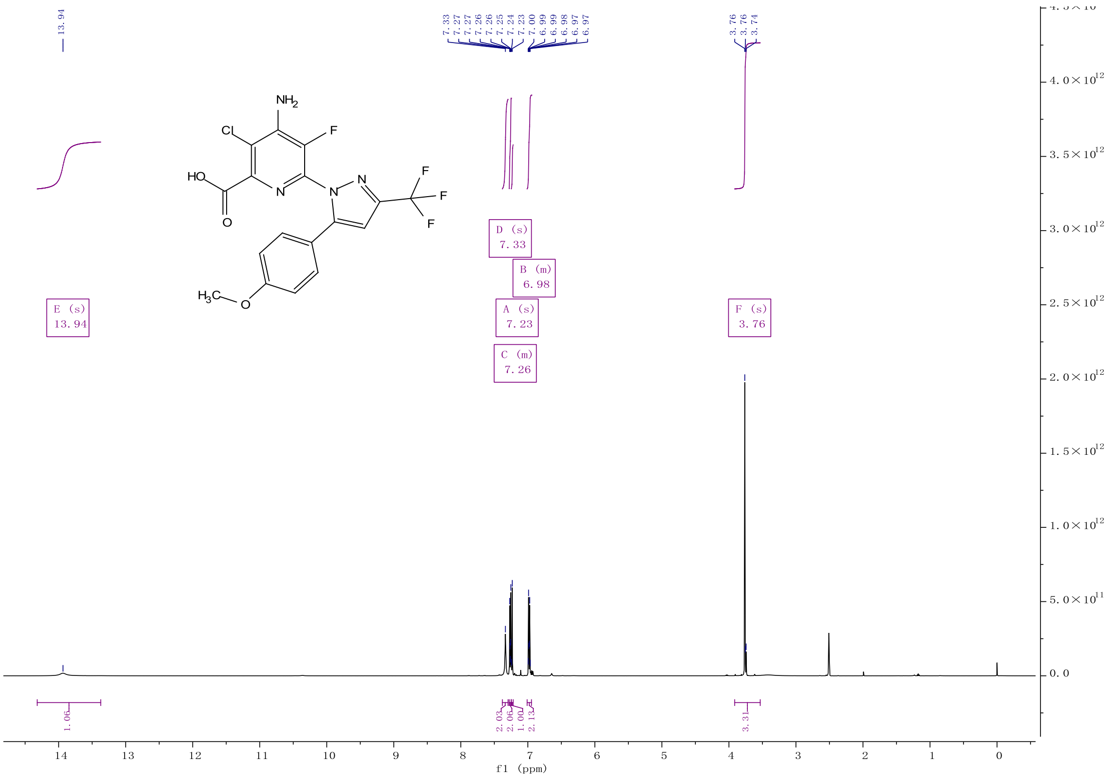

Figure S53. ^{1}H NMR (500.13 MHz, DMSO-d6) spectrum of compound S103.

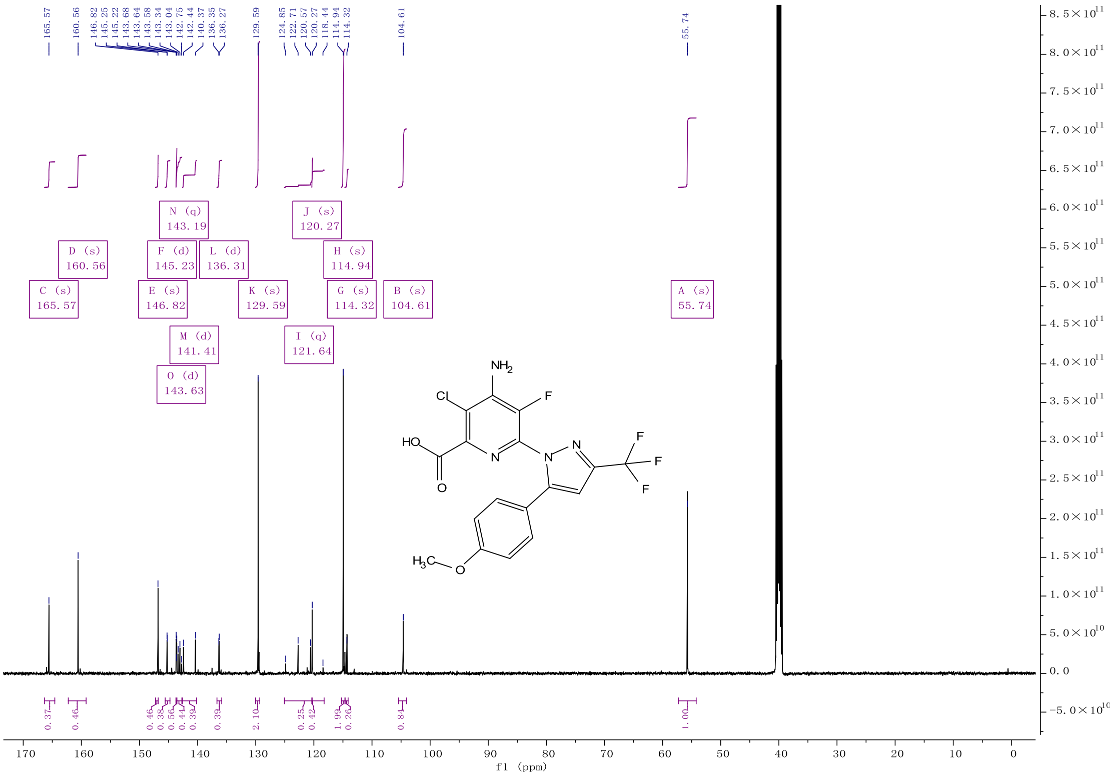

Figure S54. ^{13}C NMR (125.77 MHz, DMSO-d_6) spectrum of compound S103.

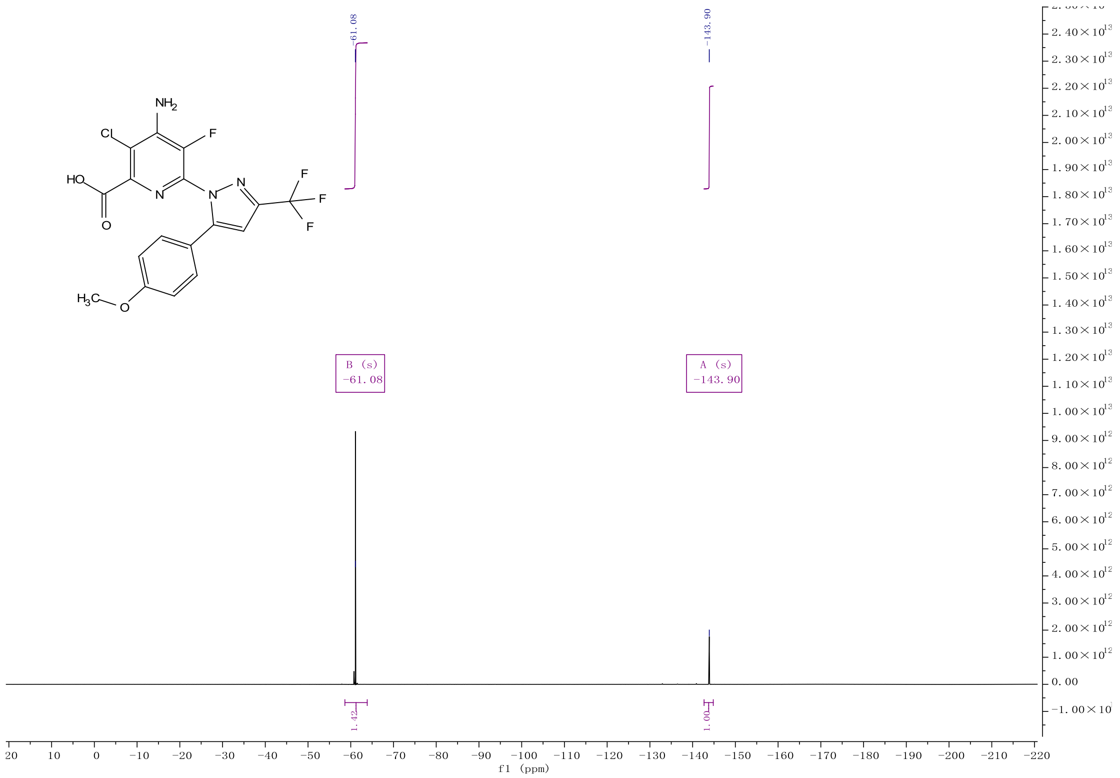

Figure S55. ^{19}F NMR (470.54 MHz, DMSO-d6) spectrum of compound S103.

Single Mass Analysis
Tolerance = 5.0 mDa / DBE: min = -1.5, max = 50.0
Element prediction: Off
Number of isotope peaks used for i-FIT = 3

Monoisotopic Mass, Even Electron Ions
4581 formula(e) evaluated with 1 results within limits (up to 50 best isotopic matches for each mass)
Elements Used:
C: 17-17 H: 11-11 N: 0-50 O: 0-50 F: 3-5 Na: 0-3 Cl: 1-2
4
250116-24-S103 24 (0.071) 1: TOF MS ES+ 1.34e+005

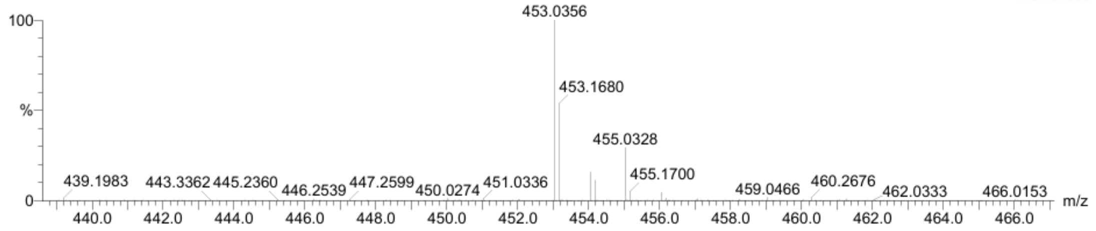

Minimum:				-1.5				
Maximum:		5.0	10.0	50.0				
Mass	Calc. Mass	mDa	PPM	DBE	i-FIT	Norm	Conf(%)	Formula
453.0356	453.0354	0.2	0.4	11.5	539.1	n/a	n/a	C17 H11 N4 O3 F4 Na Cl

Figure S56. HRMS spectrum of compound S103.

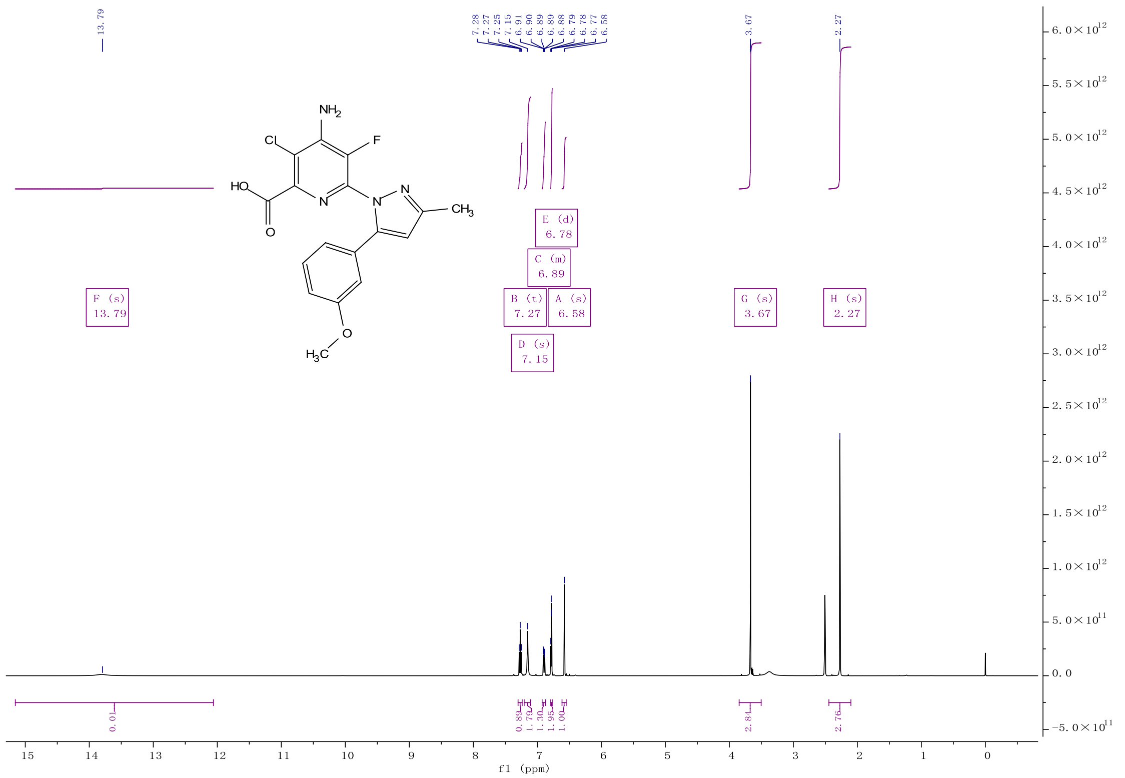

Figure S57. ^{1}H NMR (500.13 MHz, DMSO-d6) spectrum of compound S190.

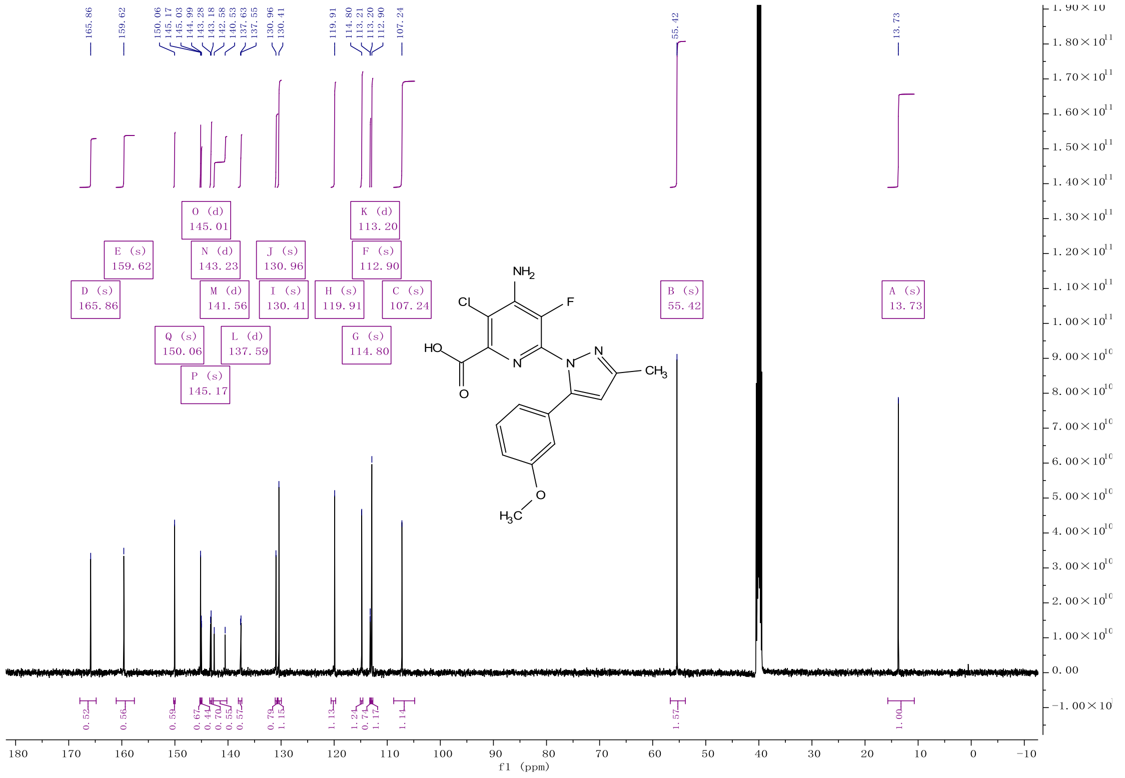

Figure S58. ^{13}C NMR (125.77 MHz, DMSO-d_6) spectrum of compound S190.

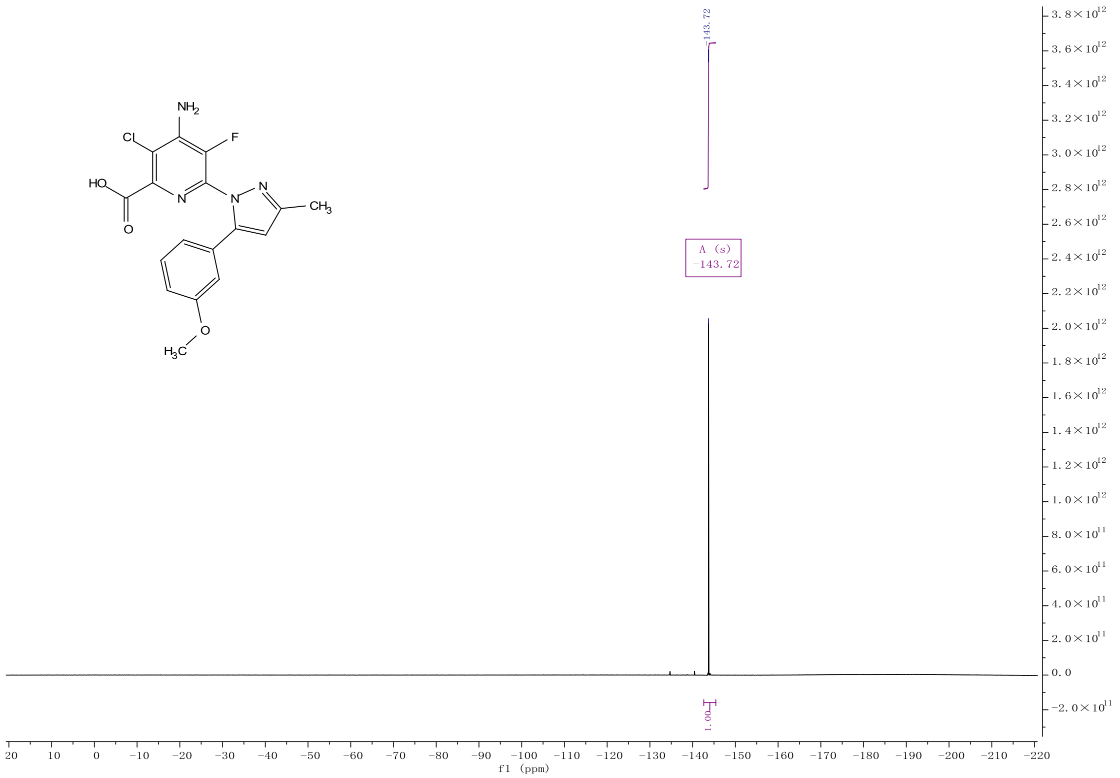

Figure S59. ^{19}F NMR (470.54 MHz, DMSO-d6) spectrum of compound S190.

Single Mass Analysis
Tolerance = 5.0 mDa / DBE: min = -1.5, max = 50.0
Element prediction: Off
Number of isotope peaks used for i-FIT = 3

Monoisotopic Mass, Even Electron Ions
4547 formula(e) evaluated with 1 results within limits (up to 50 best isotopic matches for each mass)
Elements Used:
C: 17-17 H: 15-15 N: 0-50 O: 0-50 F: 1-4 Na: 0-3 Cl: 1-2
28
250116-24-S190 33 (0.088)

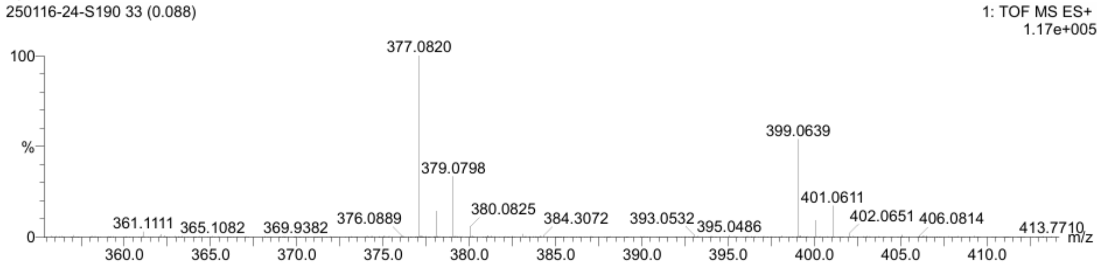

Minimum:				-1.5				
Maximum:		5.0	10.0	50.0				
Mass	Calc. Mass	mDa	PPM	DBE	i-FIT	Norm	Conf(%)	Formula
377.0820	377.0817	0.3	0.8	11.5	417.5	n/a	n/a	C17 H15 N4 O3 F Cl

Figure S60. HRMS spectrum of compound S190.

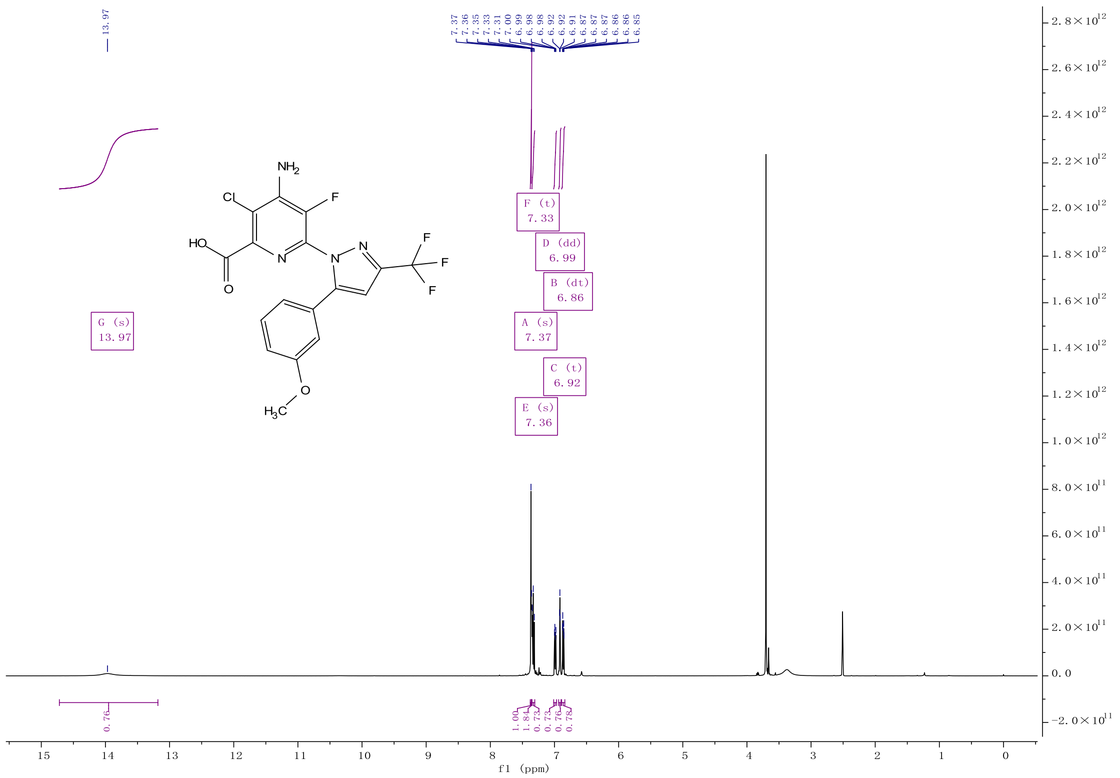

Figure S61. ^{1}H NMR (500.13 MHz, DMSO-d6) spectrum of compound S193.

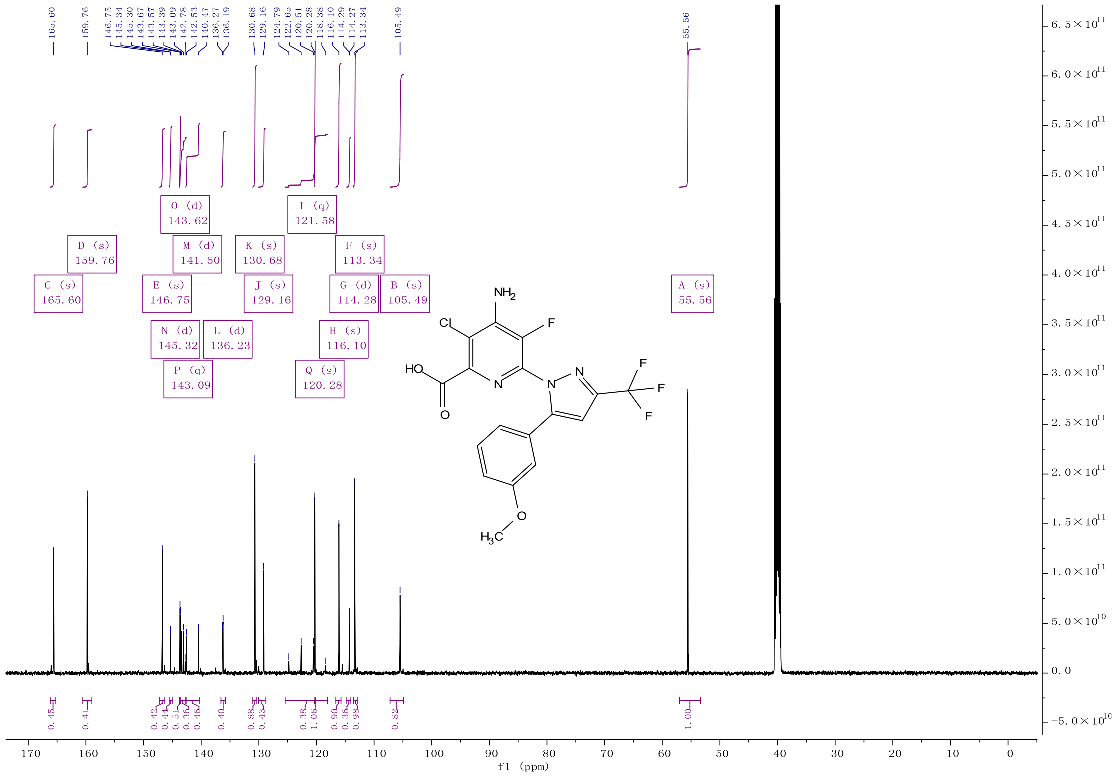

Figure S62. ^{13}C NMR (125.77 MHz, DMSO-d_6) spectrum of compound S193.

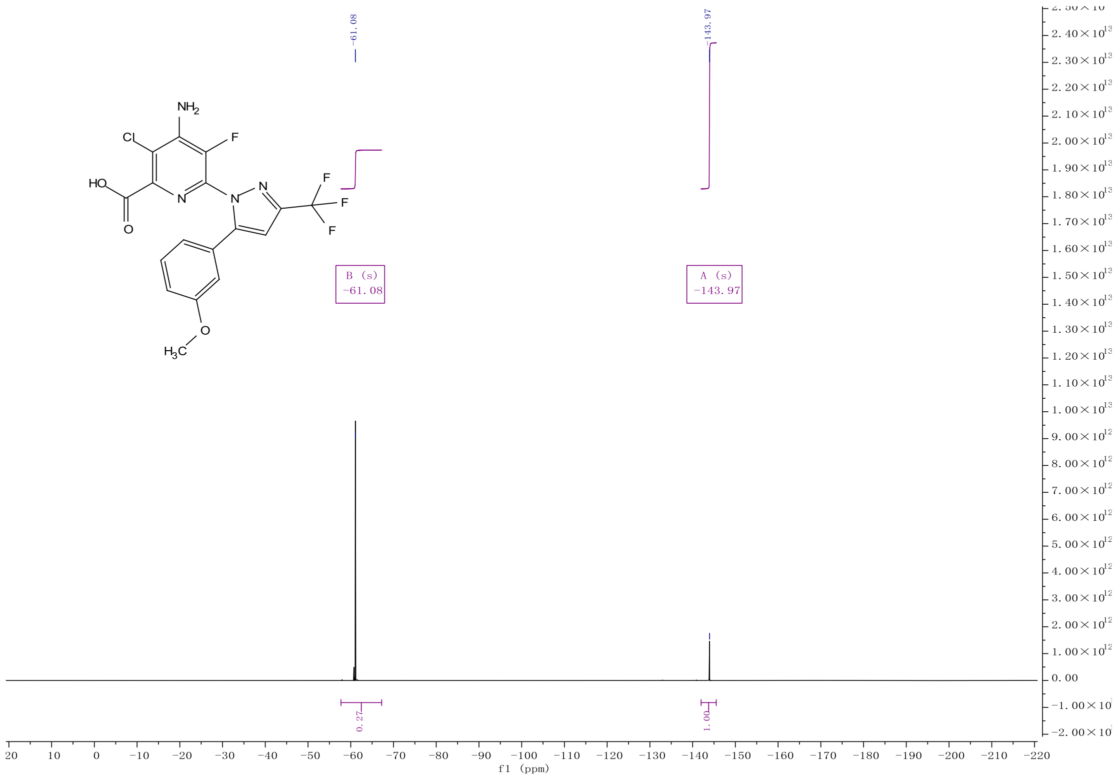

Figure S63. ^{19}F NMR (470.54 MHz, DMSO-d6) spectrum of compound S193.

Single Mass Analysis
Tolerance = 5.0 mDa / DBE: min = -1.5, max = 50.0
Element prediction: Off
Number of isotope peaks used for i-FIT = 3

Monoisotopic Mass, Even Electron Ions
4581 formula(e) evaluated with 1 results within limits (up to 50 best isotopic matches for each mass)
Elements Used:
C: 17-17 H: 11-11 N: 0-50 O: 0-50 F: 3-5 Na: 0-3 Cl: 1-2
4
250116-24-S193 26 (0.075) 1: TOF MS ES+ 3.46e+005

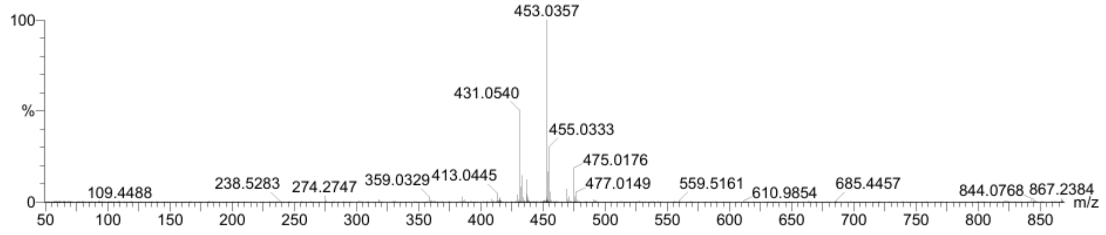

Minimum:				-1.5				
Maximum:		5.0	10.0	50.0				
Mass	Calc. Mass	mDa	PPM	DBE	i-FIT	Norm	Conf(%)	Formula
453.0357	453.0354	0.3	0.7	11.5	648.4	n/a	n/a	C17 H11 N4 O3 F4 Na Cl

Figure S64. HRMS spectrum of compound S193.

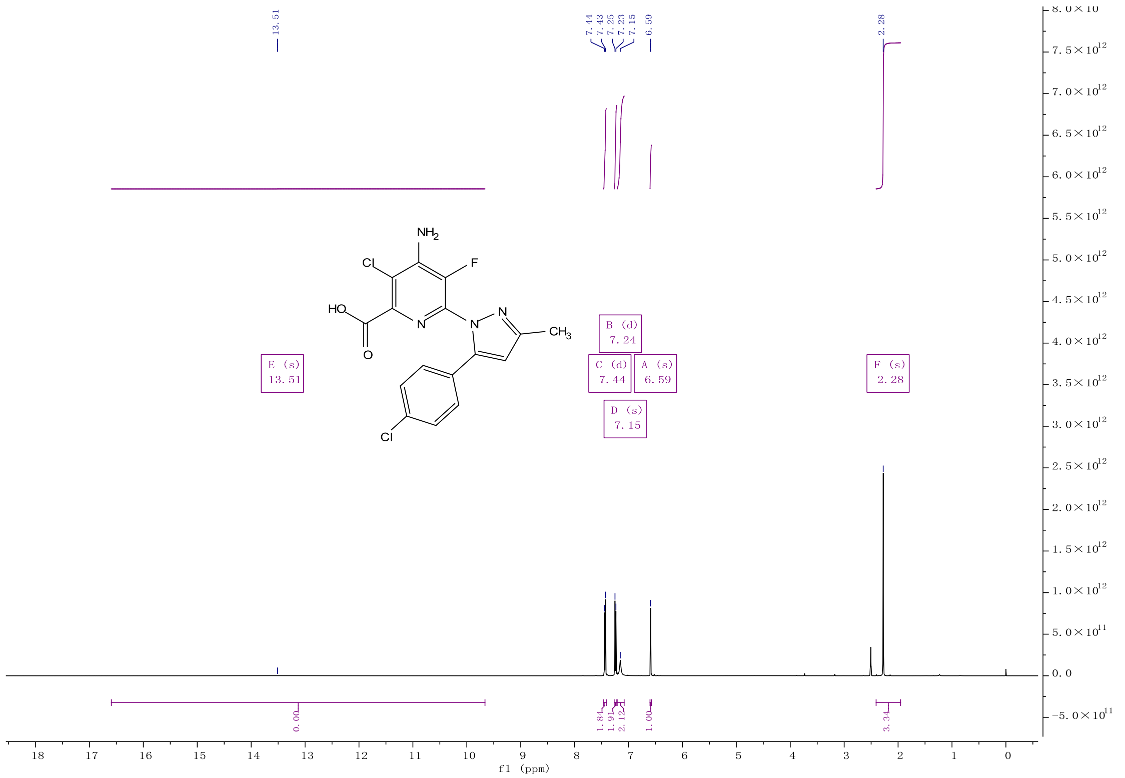

Figure S65. ^1H NMR (500.13 MHz, DMSO-d6) spectrum of compound S060.

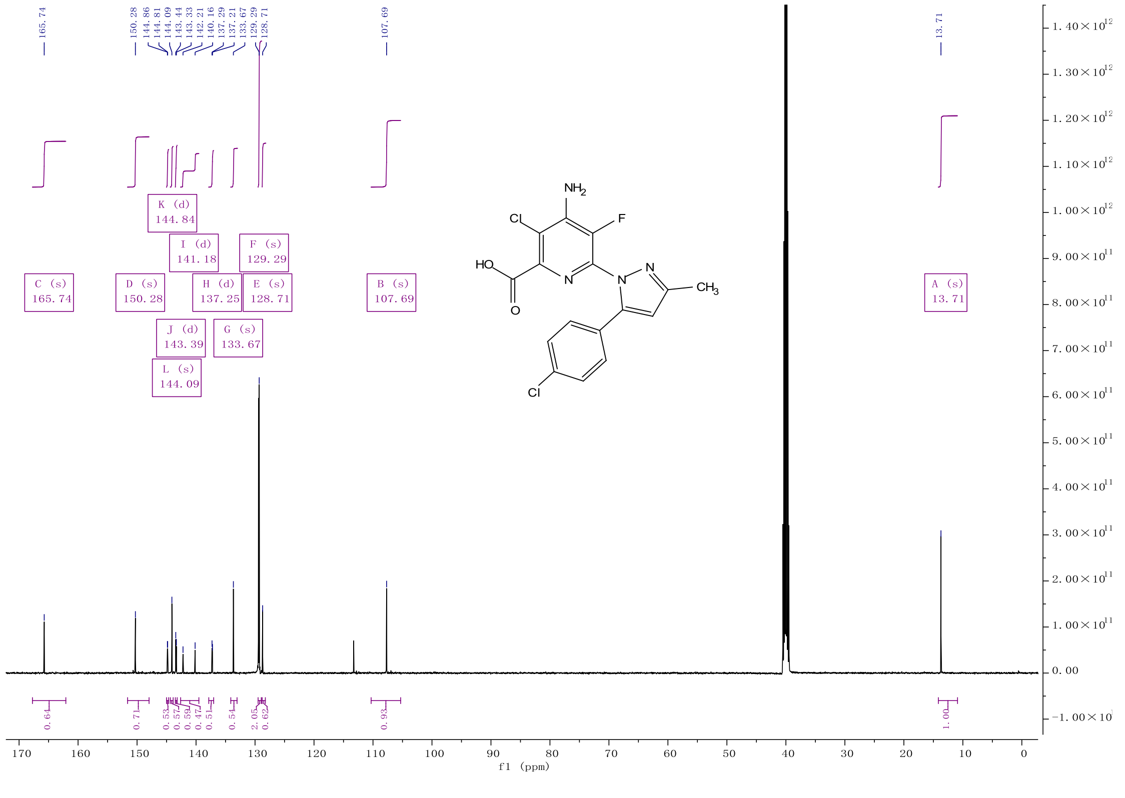

Figure S66. ^{13}C NMR (125.77 MHz, DMSO-d_6) spectrum of compound S060.

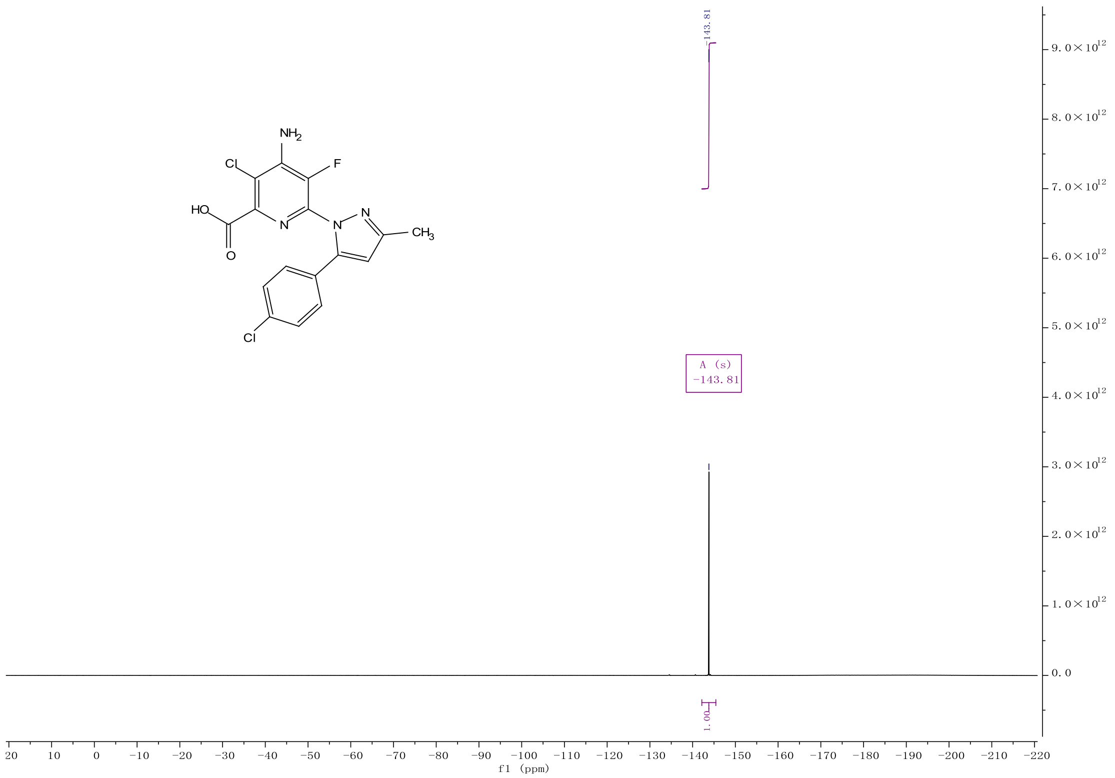

Figure S67. ^{19}F NMR (470.54 MHz, DMSO-d6) spectrum of compound S060.

Single Mass Analysis
Tolerance = 5.0 mDa / DBE: min = -1.5, max = 50.0
Element prediction: Off
Number of isotope peaks used for i-FIT = 3

Monoisotopic Mass, Even Electron Ions
6097 formula(e) evaluated with 1 results within limits (up to 50 best isotopic matches for each mass)
Elements Used:
C: 16-16 H: 12-12 N: 0-50 O: 0-50 F: 1-6 Na: 0-3 Cl: 1-2
27
250116-24-S060 40 (0.101)

1: TOF MS ES+
4.62e+004

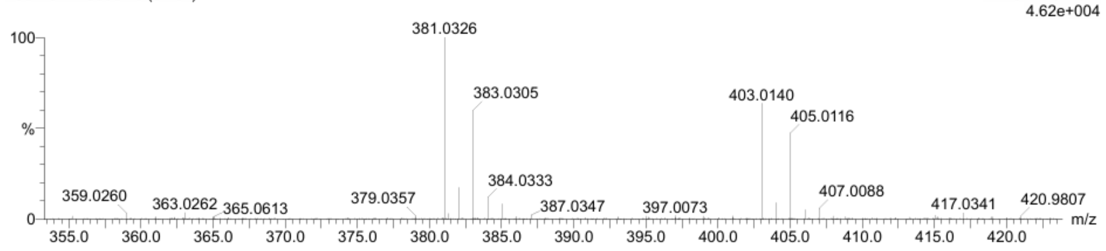

Minimum:				-1.5				
Maximum:		5.0	10.0	50.0				
Mass	Calc. Mass	mDa	PPM	DBE	i-FIT	Norm	Conf(%)	Formula
381.0326	381.0321	0.5	1.3	11.5	272.8	n/a	n/a	C16 H12 N4 O2 F Cl2

Figure S68. HRMS spectrum of compound S060.

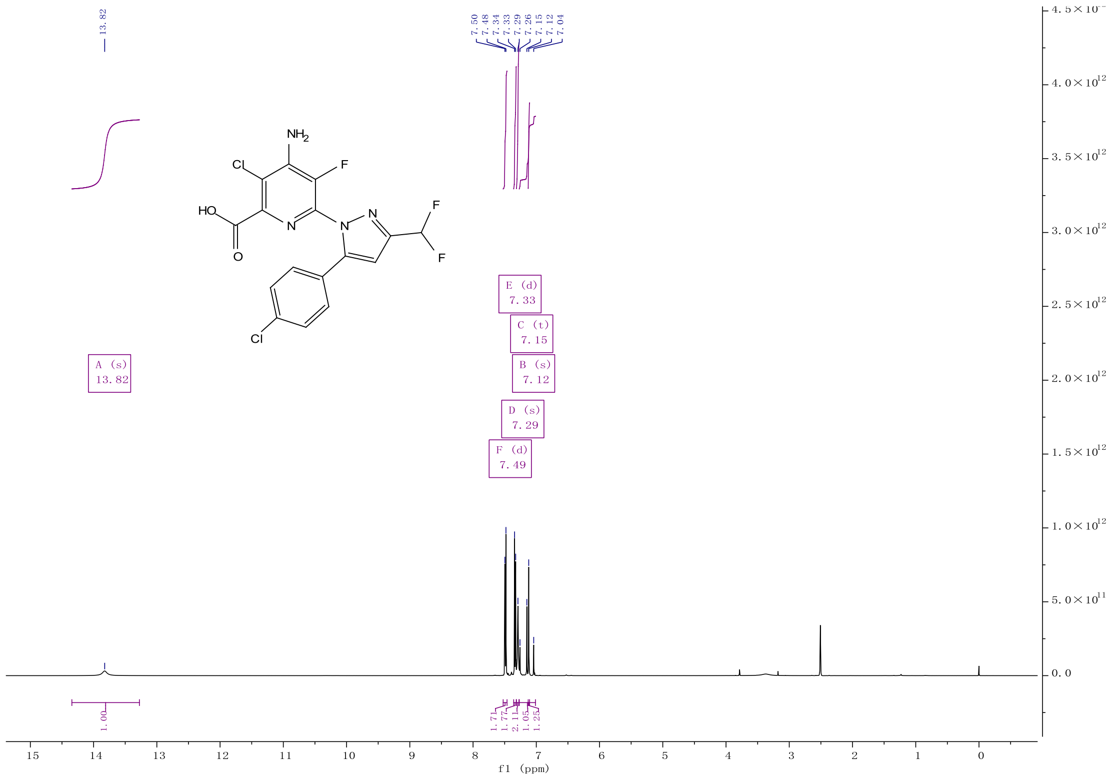

Figure S69. ^{1}H NMR (500.13 MHz, DMSO-d6) spectrum of compound S062.

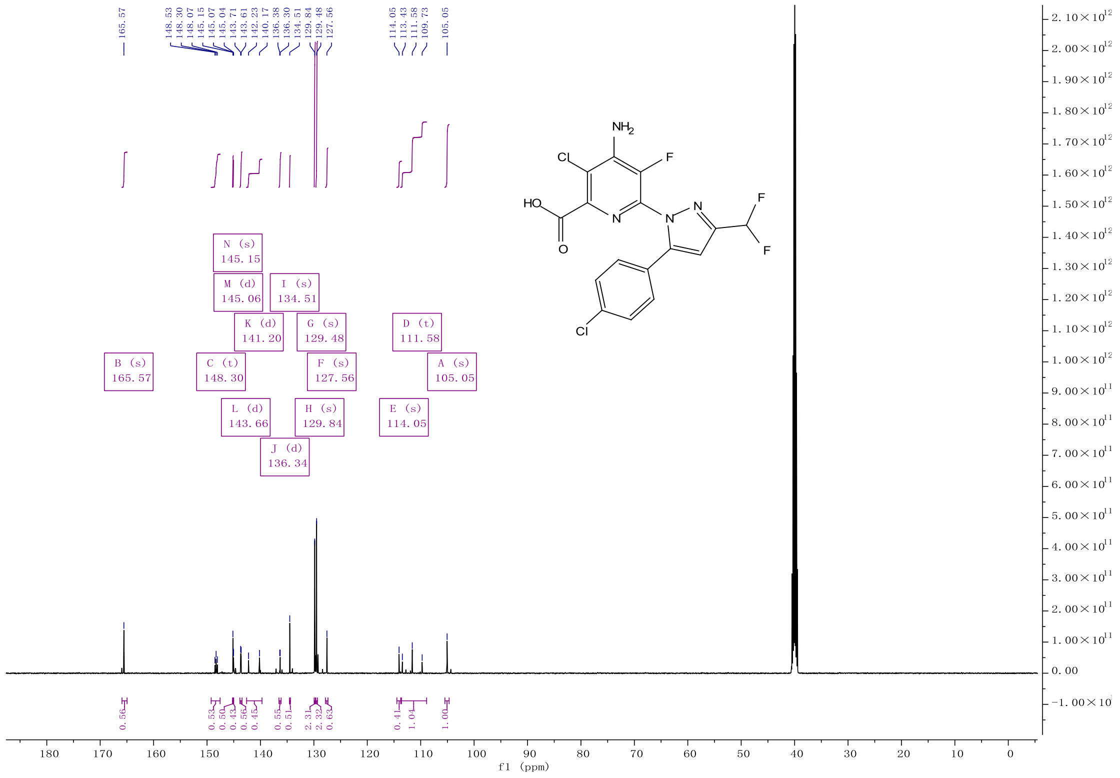

Figure S70. ^{13}C NMR (125.77 MHz, DMSO-d_6) spectrum of compound S062.

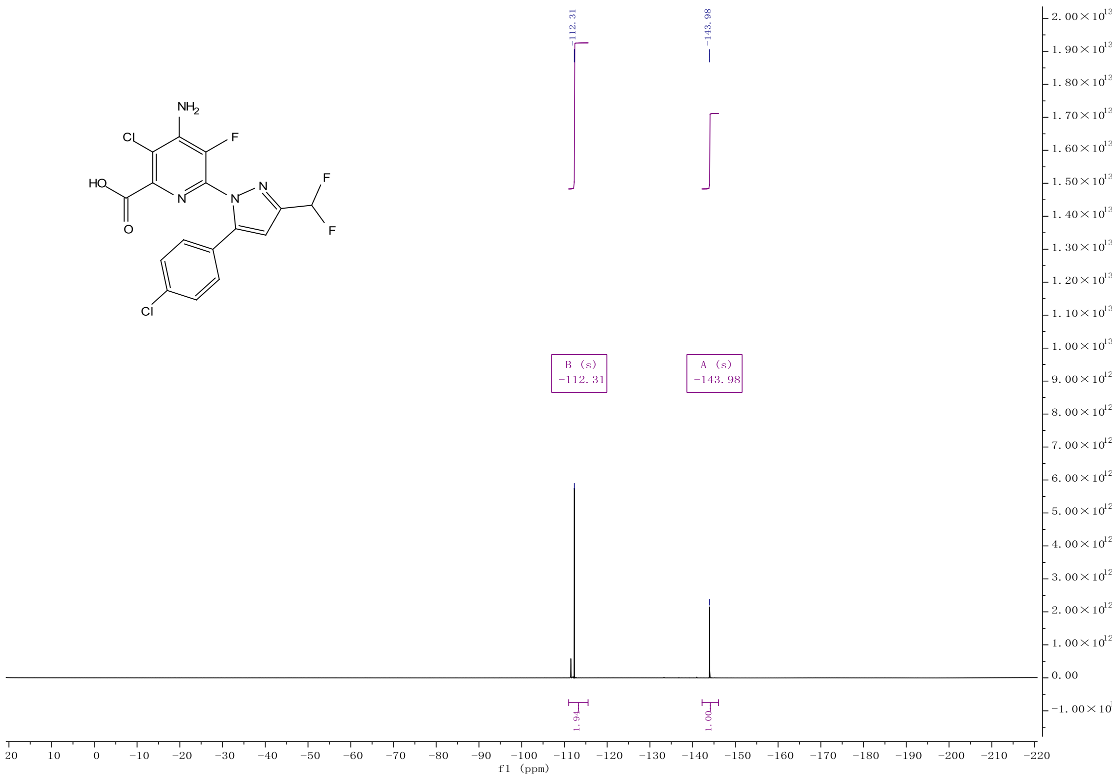

Figure S71. ^{19}F NMR (470.54 MHz, DMSO-d6) spectrum of compound S062.

Single Mass Analysis
Tolerance = 5.0 mDa / DBE: min = -1.5, max = 50.0
Element prediction: Off
Number of isotope peaks used for i-FIT = 3

Monoisotopic Mass, Even Electron Ions
5785 formula(e) evaluated with 1 results within limits (up to 50 best isotopic matches for each mass)
Elements Used:
C: 16-16 H: 10-10 N: 0-50 O: 0-50 F: 1-4 Na: 0-3 Cl: 1-2
27
250116-24-S062 26 (0.075)

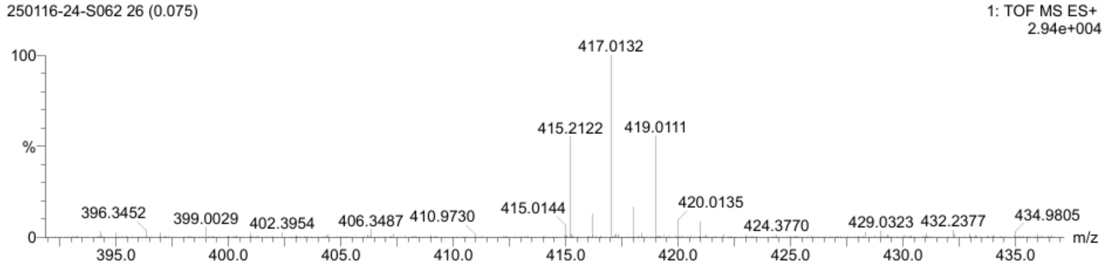

Minimum:				-1.5				
Maximum:		5.0	10.0	50.0				
Mass	Calc. Mass	mDa	PPM	DBE	i-FIT	Norm	Conf(%)	Formula
417.0132	417.0133	-0.1	-0.2	11.5	309.5	n/a	n/a	C16 H10 N4 O2 F3 Cl2

Figure S72. HRMS spectrum of compound S062.

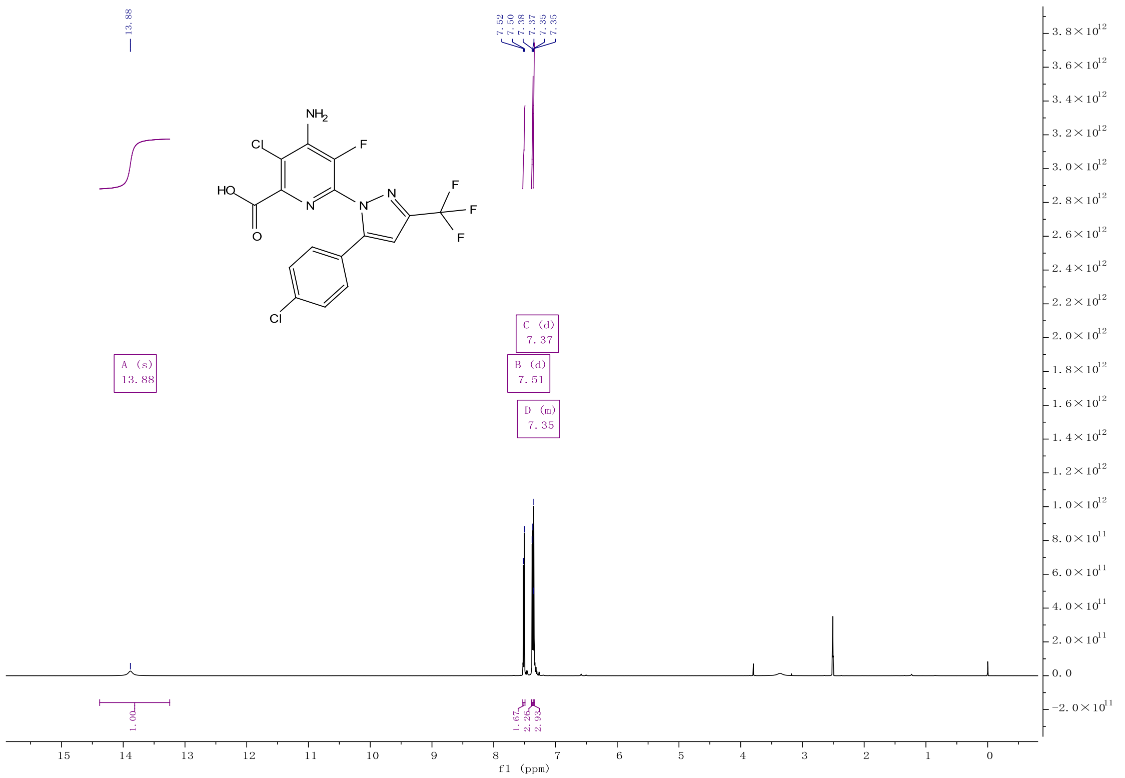

Figure S73. ^{1}H NMR (500.13 MHz, DMSO-d6) spectrum of compound S063.

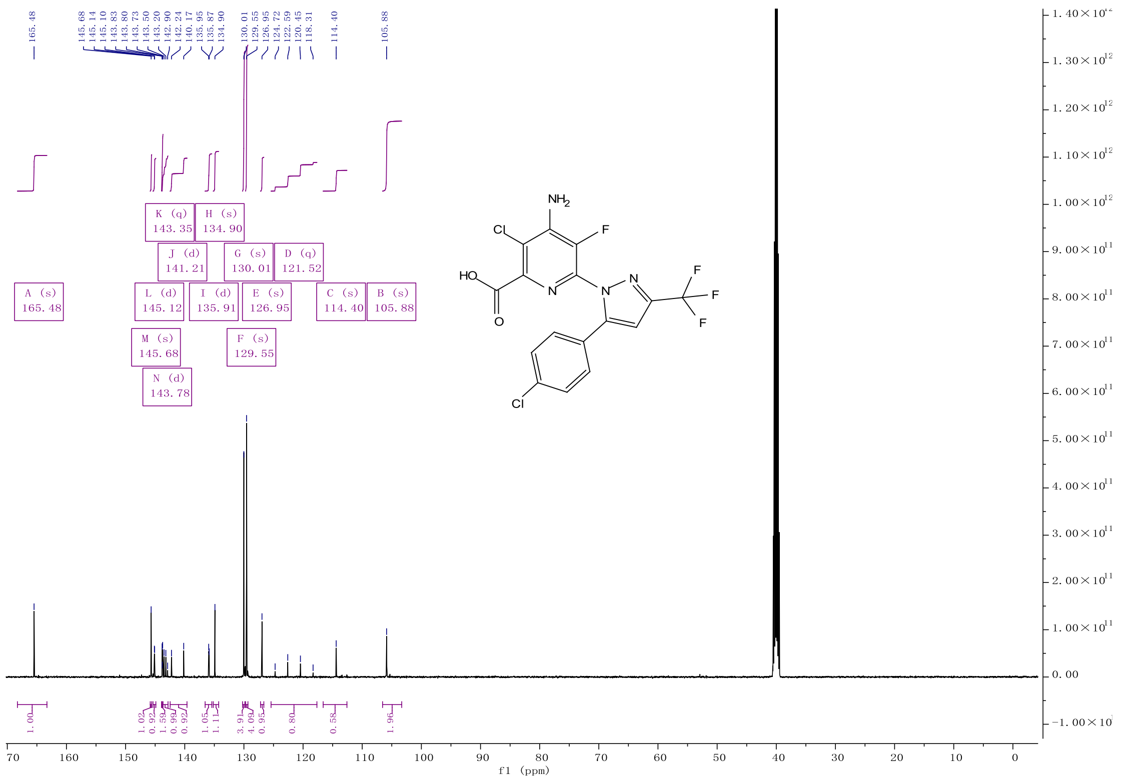

Figure S74. ^{13}C NMR (125.77 MHz, DMSO-d_6) spectrum of compound S063.

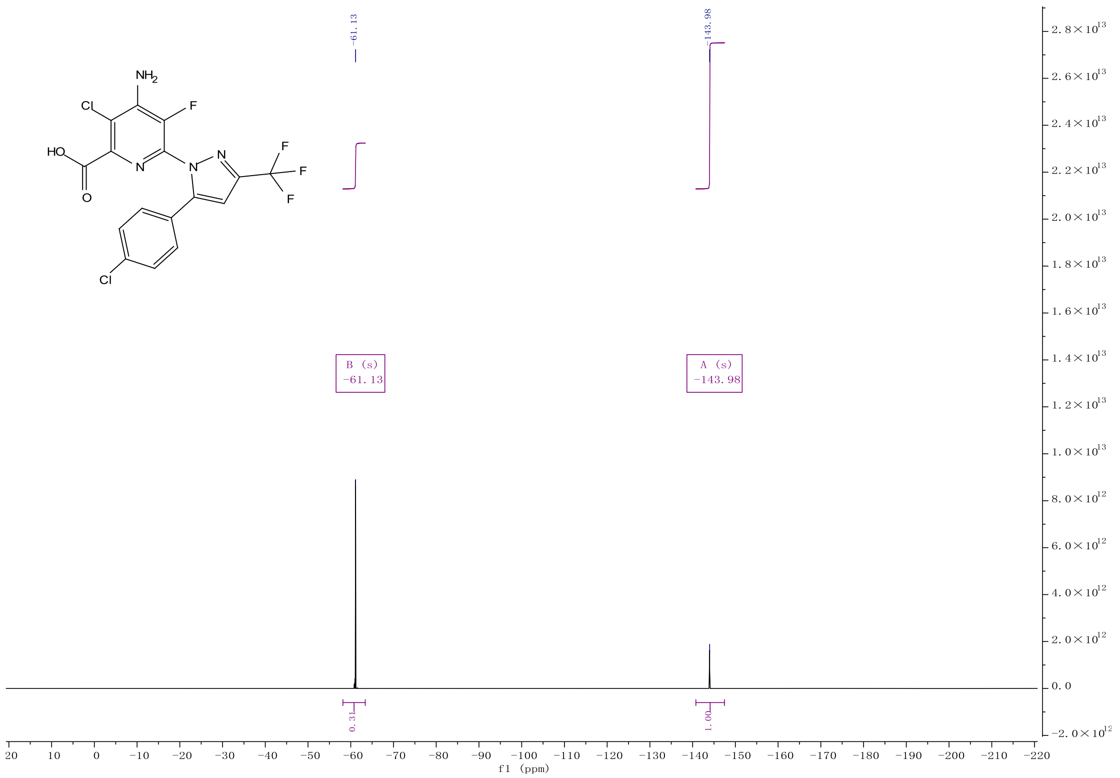

Figure S75. ^{19}F NMR (470.54 MHz, DMSO-d6) spectrum of compound S063.

Single Mass Analysis
Tolerance = 5.0 mDa / DBE: min = -1.5, max = 50.0
Element prediction: Off
Number of isotope peaks used for i-FIT = 3

Monoisotopic Mass, Even Electron Ions
7309 formula(e) evaluated with 1 results within limits (up to 50 best isotopic matches for each mass)
Elements Used:
C: 16-16 H: 8-8 N: 0-50 O: 0-50 F: 1-4 Na: 0-3 Cl: 1-2
28
250116-24-S063 32 (0.086) 1: TOF MS ES+ 3.41e+002

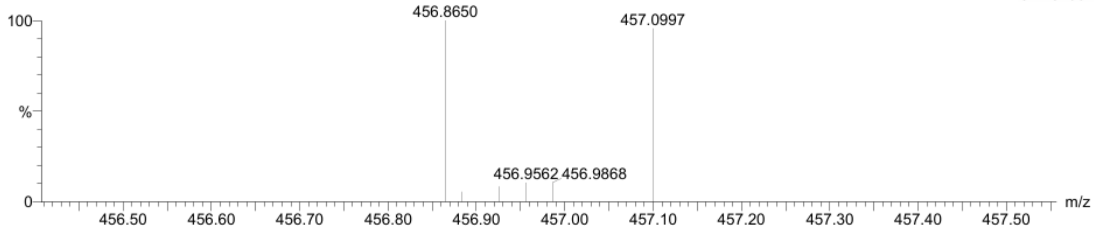

Minimum:				-1.5				
Maximum:		5.0	10.0	50.0				
Mass	Calc. Mass	mDa	PPM	DBE	i-FIT	Norm	Conf(%)	Formula
456.9868	456.9858	1.0	2.2	11.5	28.0	n/a	n/a	C16 H8 N4 O2 F4 Na Cl2

Figure S76. HRMS spectrum of compound S063.

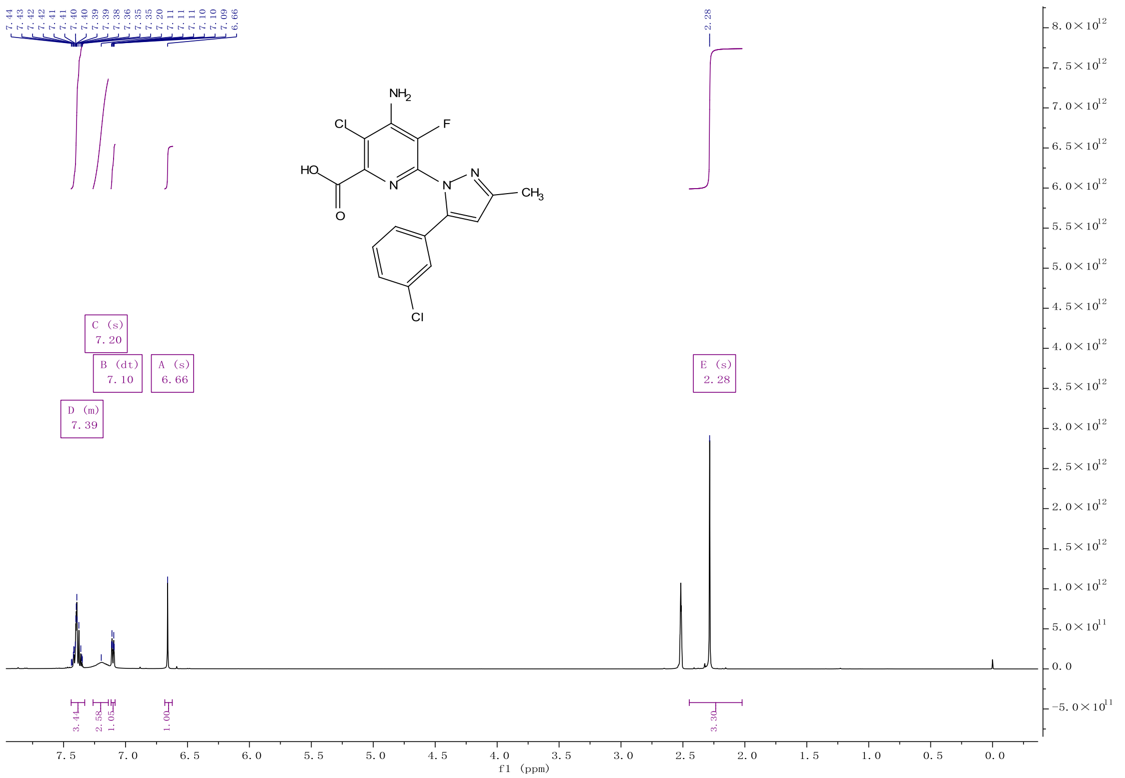

Figure S77. ^{1}H NMR (500.13 MHz, DMSO-d6) spectrum of compound S130.

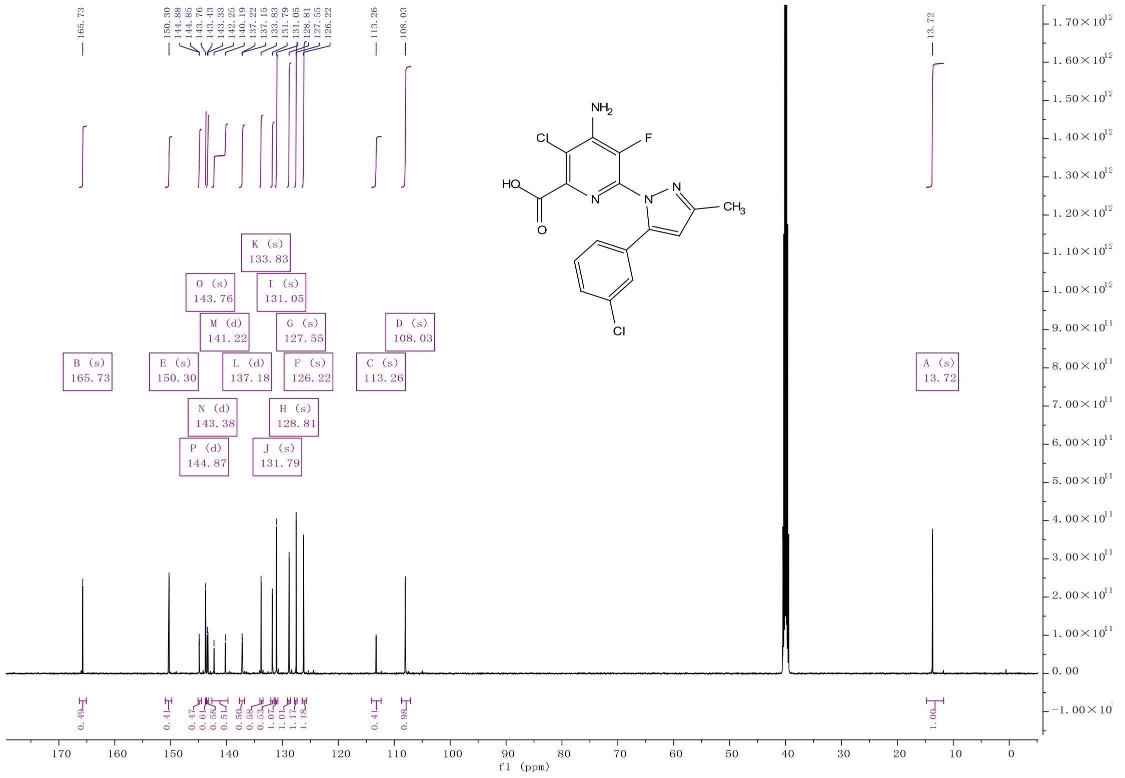

Figure S78. ^{13}C NMR (125.77 MHz, DMSO-d_6) spectrum of compound S130.

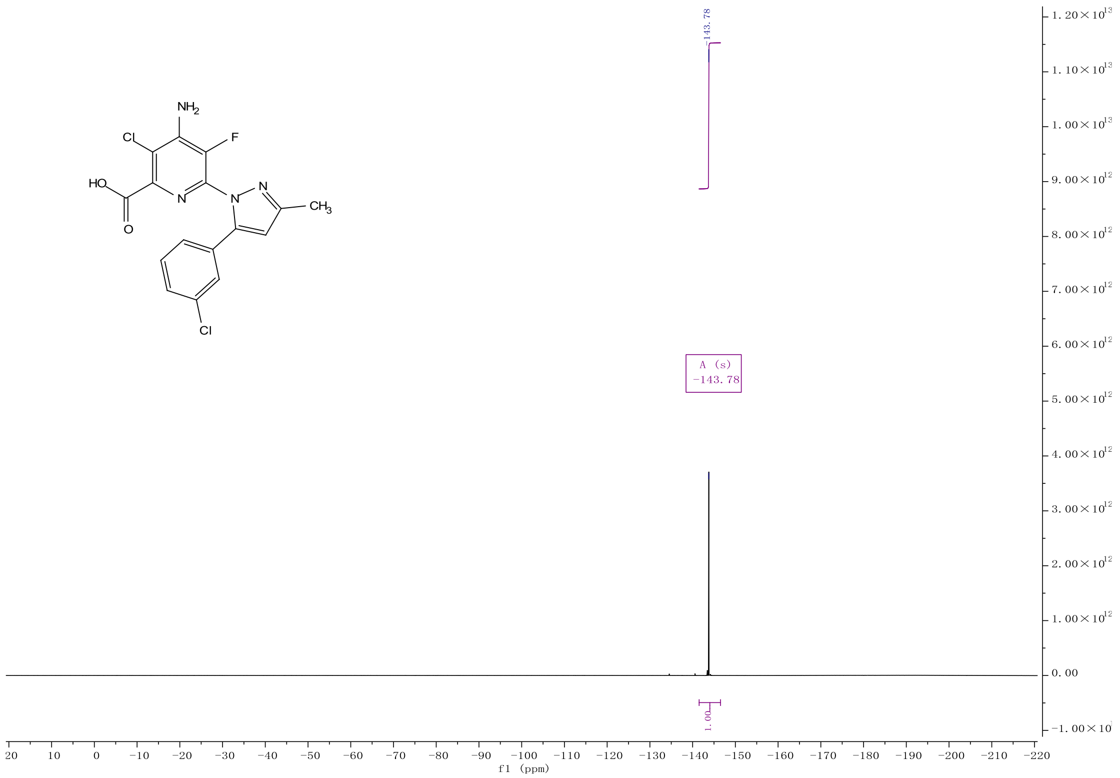

Figure S79. ^{19}F NMR (470.54 MHz, DMSO-d6) spectrum of compound S130.

Single Mass Analysis
Tolerance = 5.0 mDa / DBE: min = -1.5, max = 50.0
Element prediction: Off
Number of isotope peaks used for i-FIT = 3

Monoisotopic Mass, Even Electron Ions
6097 formula(e) evaluated with 1 results within limits (up to 50 best isotopic matches for each mass)
Elements Used:
C: 16-16 H: 12-12 N: 0-50 O: 0-50 F: 1-6 Na: 0-3 Cl: 1-2
27
250116-24-S130 32 (0.086)
1: TOF MS ES+
2.77e+005

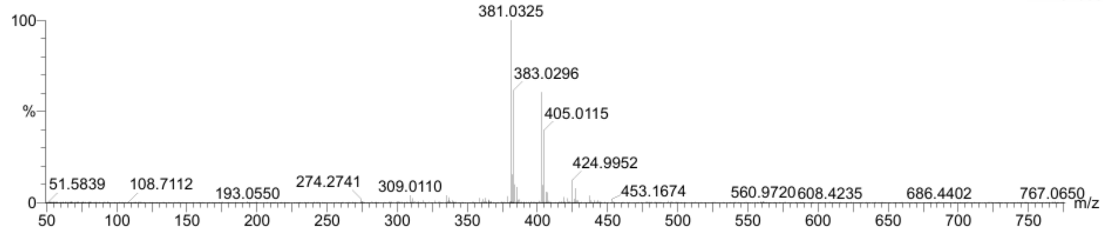

Minimum:				-1.5				
Maximum:		5.0	10.0	50.0				
Mass	Calc. Mass	mDa	PPM	DBE	i-FIT	Norm	Conf(%)	Formula
381.0325	381.0321	0.4	1.0	11.5	560.9	n/a	n/a	C16 H12 N4 O2 F Cl2

Figure S80. HRMS spectrum of compound S130.

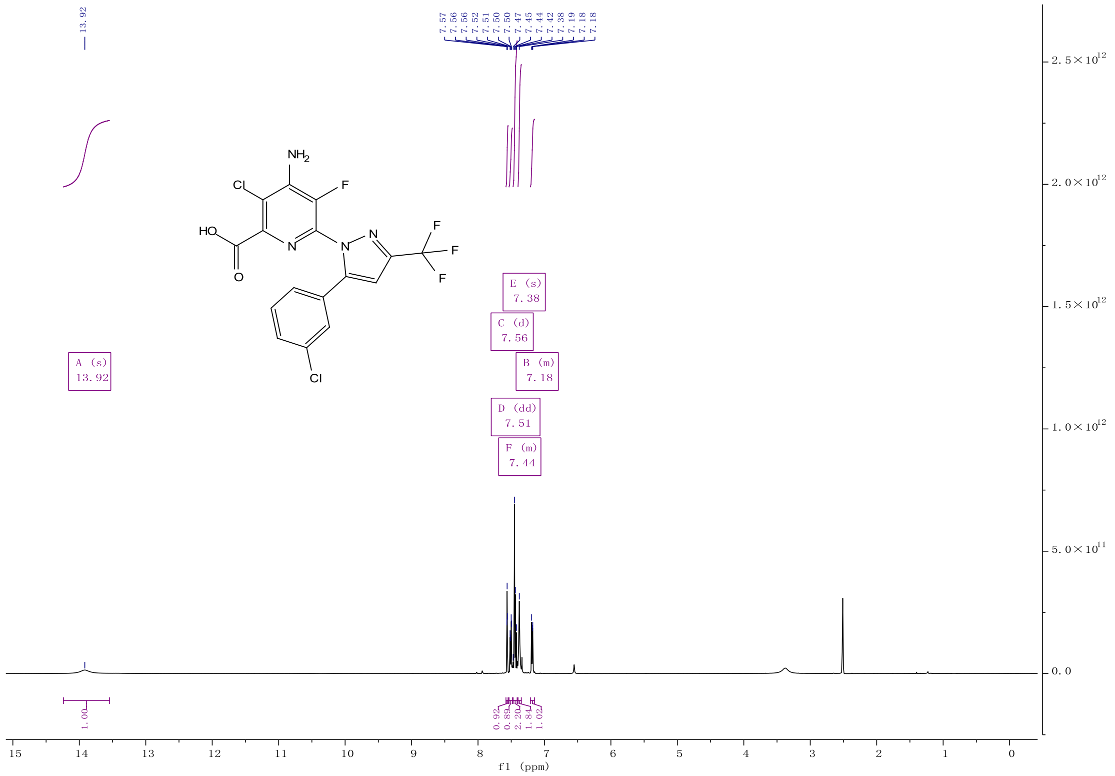

Figure S81. ^{1}H NMR (500.13 MHz, DMSO-d6) spectrum of compound S133.

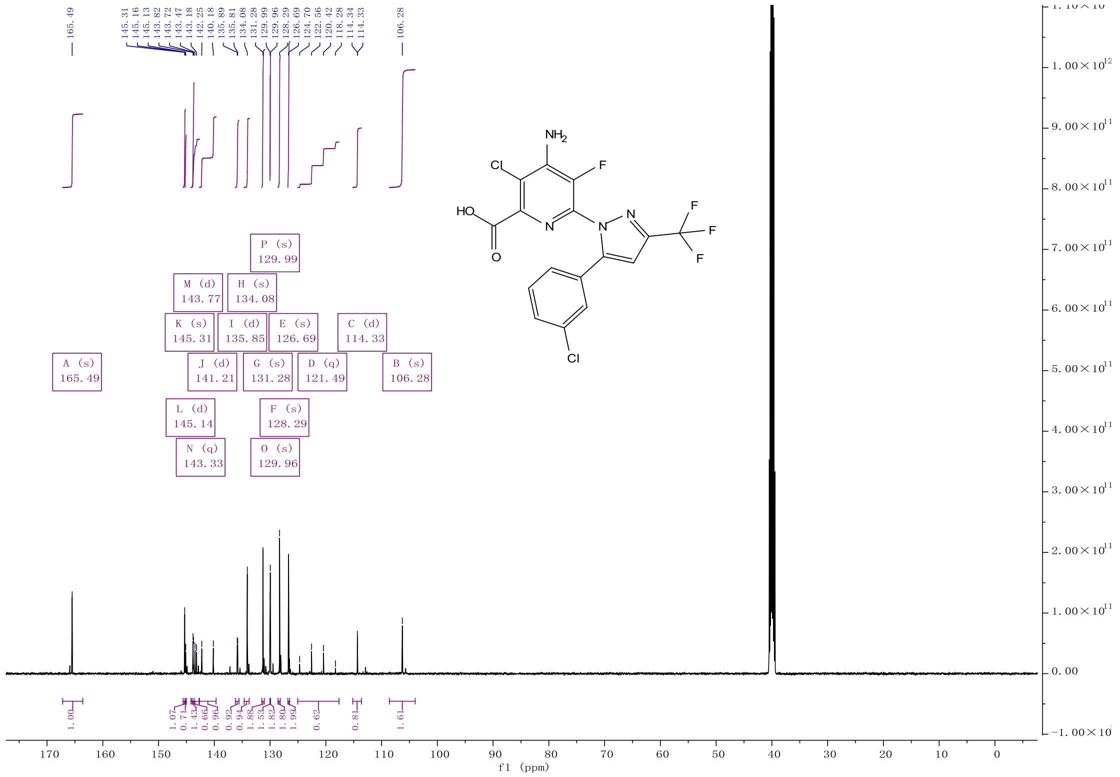

Figure S82. ^{13}C NMR (125.77 MHz, DMSO-d_6) spectrum of compound S133.

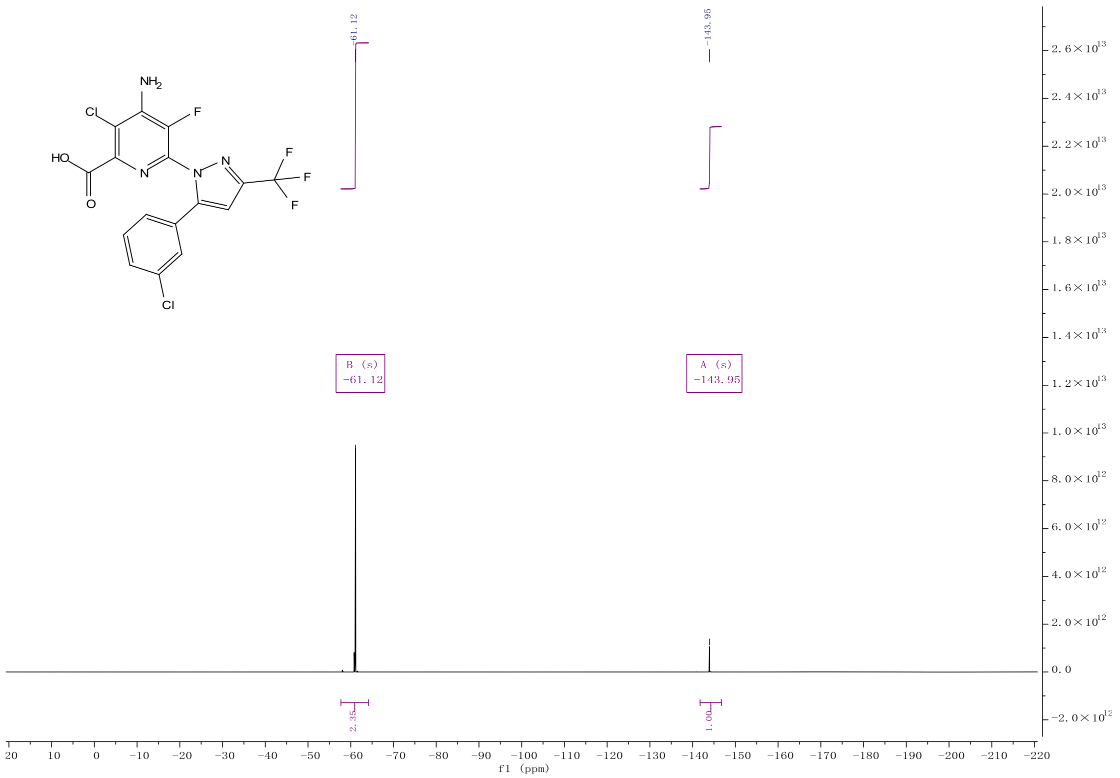

Figure S83. ^{19}F NMR (470.54 MHz, DMSO-d6) spectrum of compound S133.

Single Mass Analysis
Tolerance = 5.0 mDa / DBE: min = -1.5, max = 50.0
Element prediction: Off
Number of isotope peaks used for i-FIT = 3

Monoisotopic Mass, Even Electron Ions
7309 formula(e) evaluated with 1 results within limits (up to 50 best isotopic matches for each mass)
Elements Used:
C: 16-16 H: 8-8 N: 0-50 O: 0-50 F: 1-4 Na: 0-3 Cl: 1-2
28
250116-24-S133--------------- 71 (0.160)

1: TOF MS ES+
1.06e+004

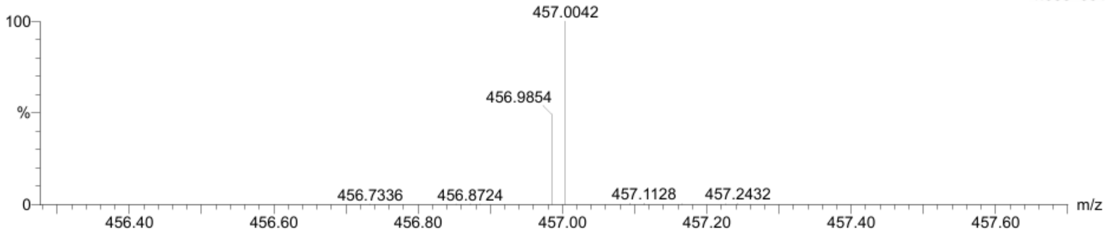

		mDa	PPM	DBE				
Minimum:				-1.5				
Maximum:		5.0	10.0	50.0				

Mass	Calc. Mass	mDa	PPM	DBE	i-FIT	Norm	Conf(%)	Formula
456.9854	456.9858	-0.4	-0.9	11.5	86.3	n/a	n/a	C16 H8 N4 O2 F4 Na Cl2

Figure S84. HRMS spectrum of compound S133.

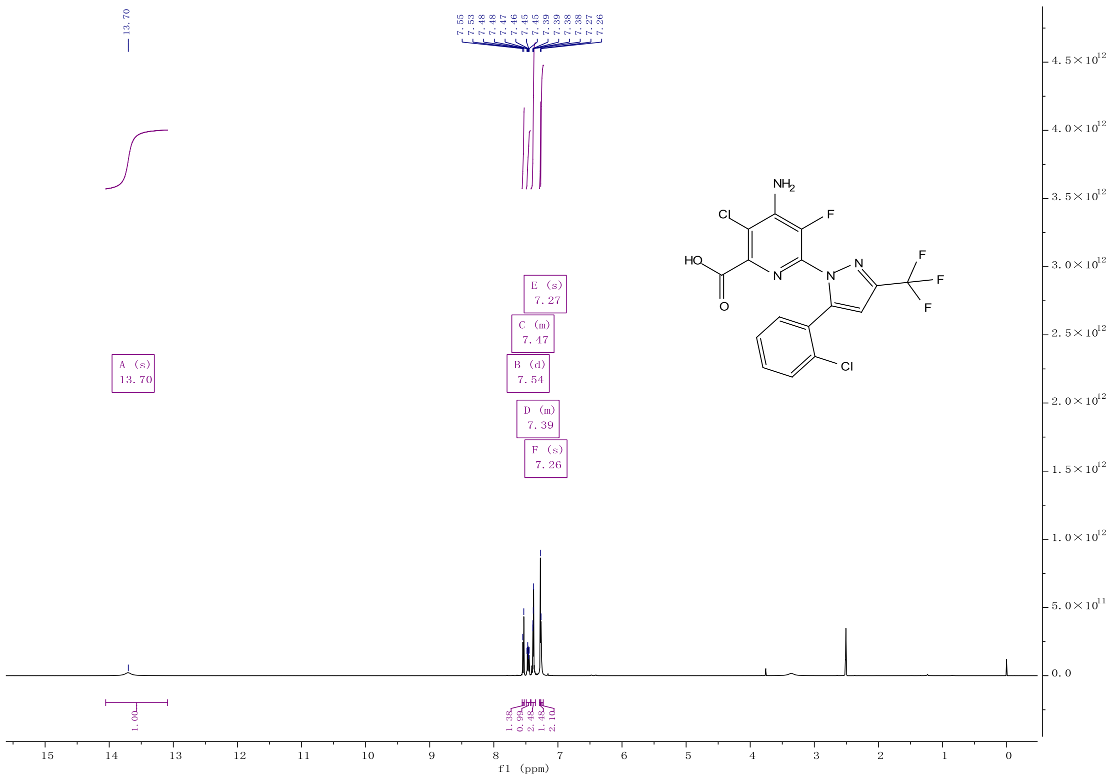

Figure S85. ^{1}H NMR (500.13 MHz, DMSO-d6) spectrum of compound S123.

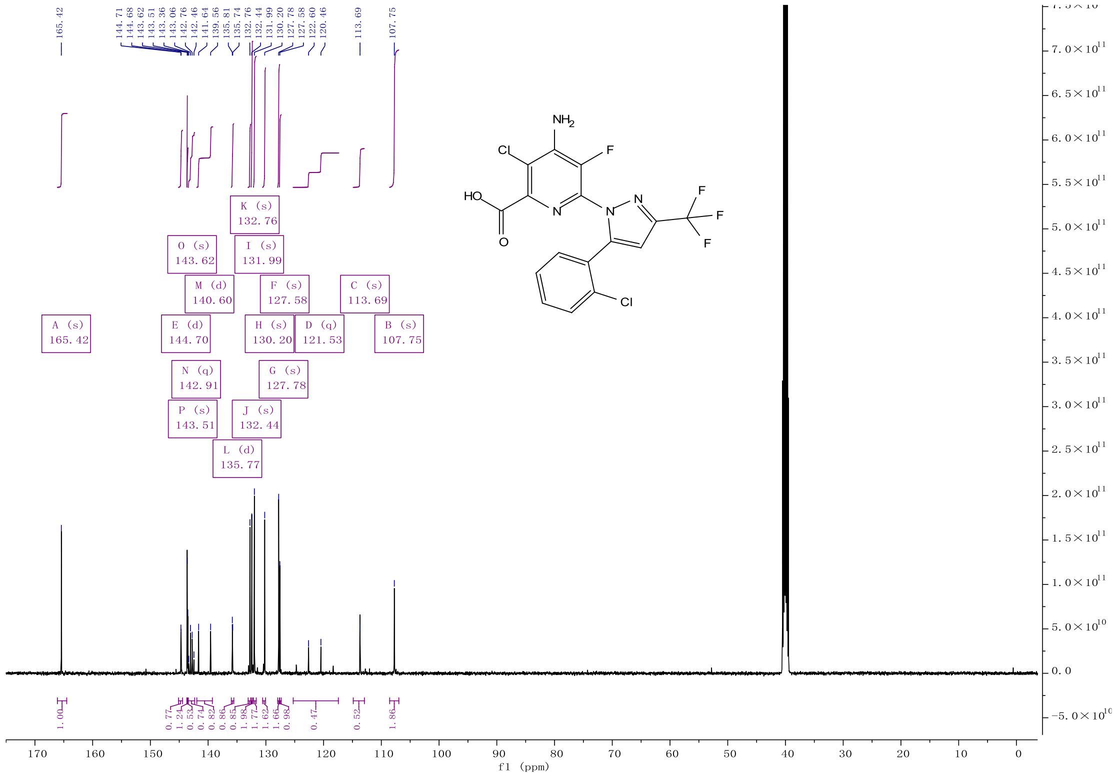

Figure S86. ^{13}C NMR (125.77 MHz, DMSO-d_6) spectrum of compound S123.

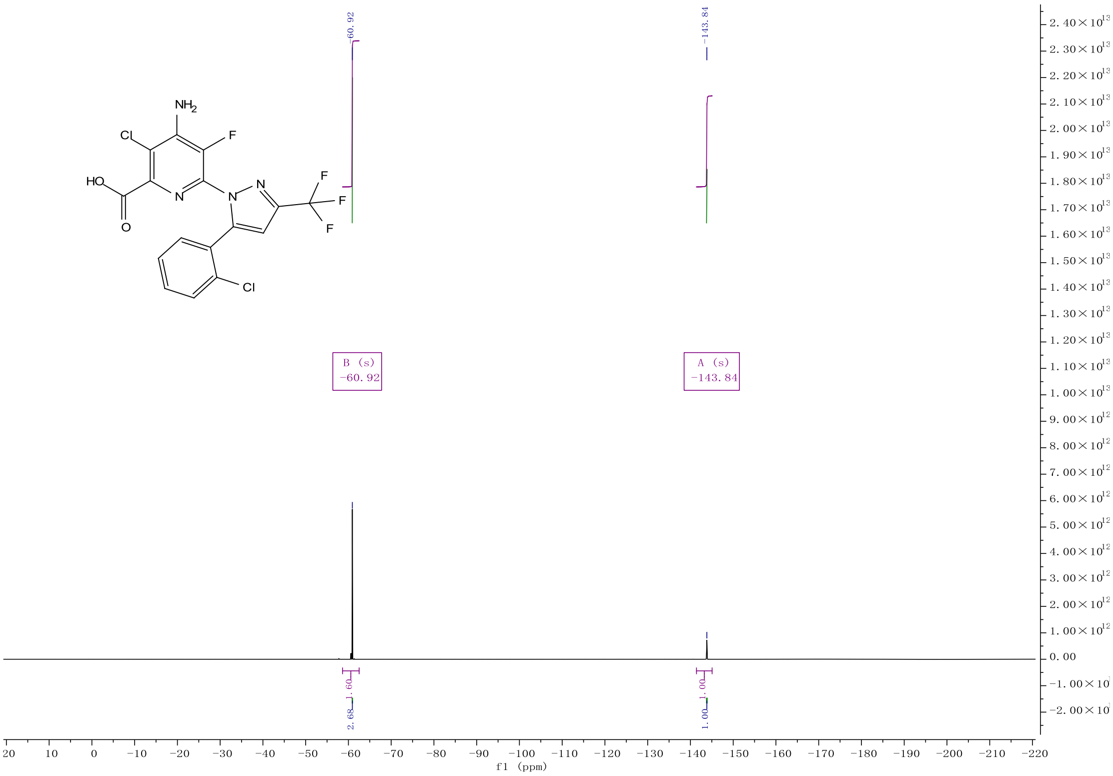

Figure S87. ^{19}F NMR (470.54 MHz, DMSO-d6) spectrum of compound S123.

Single Mass Analysis
Tolerance = 5.0 mDa / DBE: min = -1.5, max = 50.0
Element prediction: Off
Number of isotope peaks used for i-FIT = 3

Monoisotopic Mass, Even Electron Ions
5865 formula(e) evaluated with 1 results within limits (up to 50 best isotopic matches for each mass)
Elements Used:
C: 16-16 H: 8-8 N: 0-50 O: 0-50 F: 3-6 Na: 0-3 Cl: 1-2
27
250116-24-S123 29 (0.080) 1: TOF MS ES+ 2.64e+005

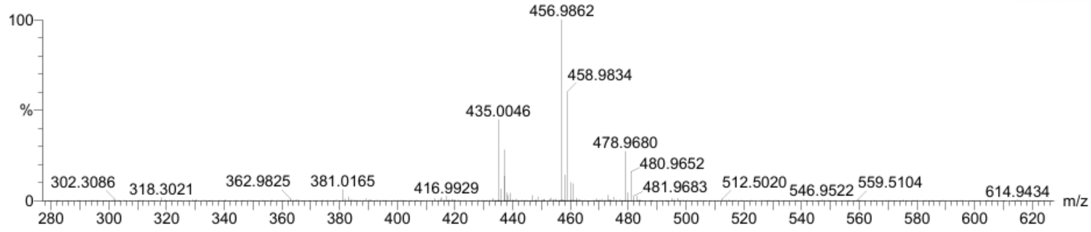

Minimum:				-1.5				
Maximum:		5.0	10.0	50.0				
Mass	Calc. Mass	mDa	PPM	DBE	i-FIT	Norm	Conf(%)	Formula
456.9862	456.9858	0.4	0.9	11.5	493.5	n/a	n/a	C16 H8 N4 O2 F4 Na Cl2

Figure S88. HRMS spectrum of compound S123.

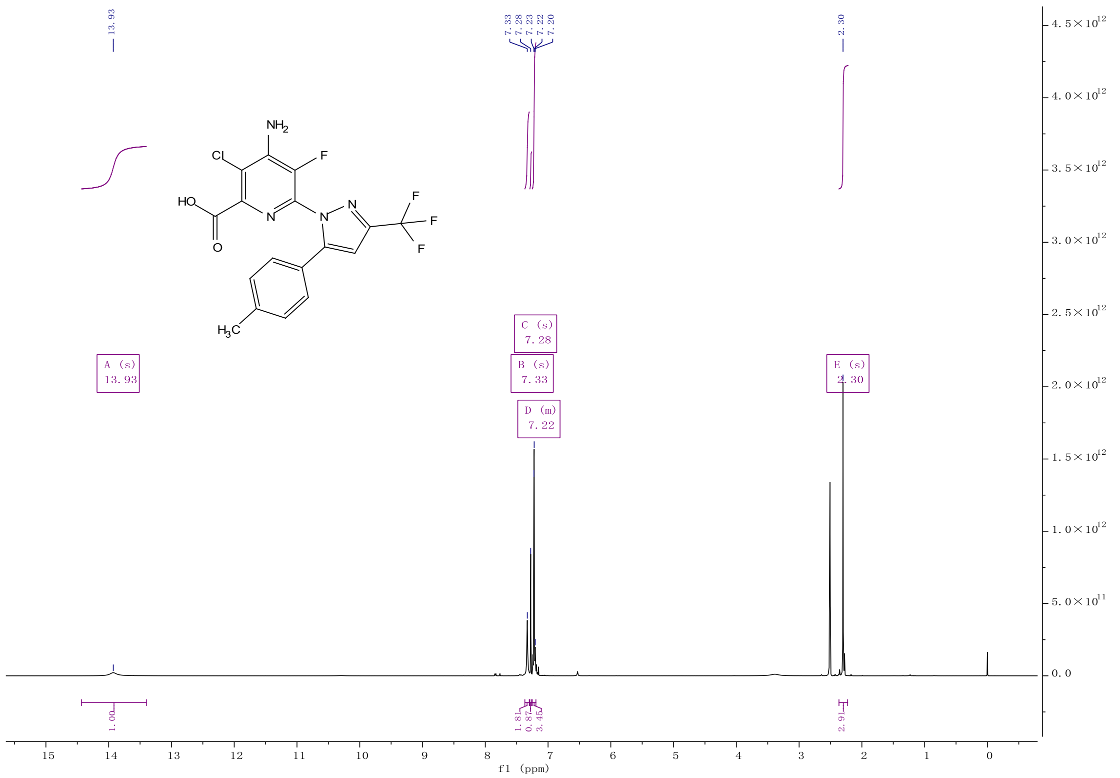

Figure S89. ^{1}H NMR (500.13 MHz, DMSO-d6) spectrum of compound S053.

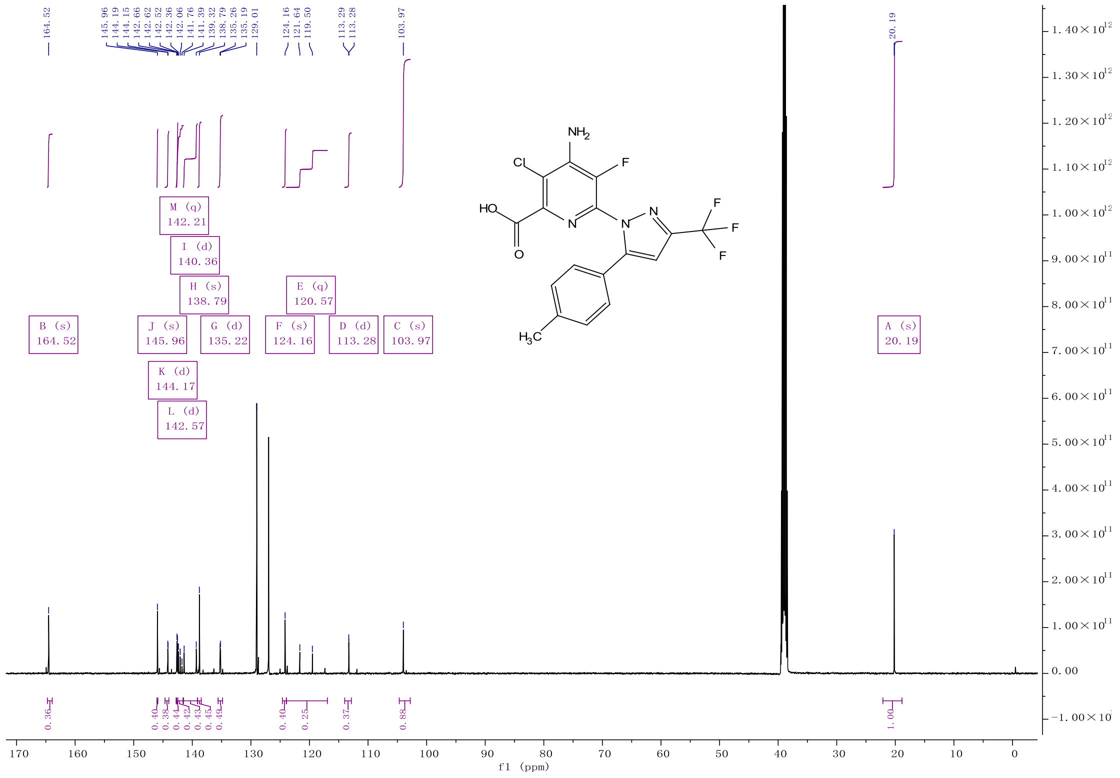

Figure S90. ^{13}C NMR (125.77 MHz, DMSO-d_6) spectrum of compound S053.

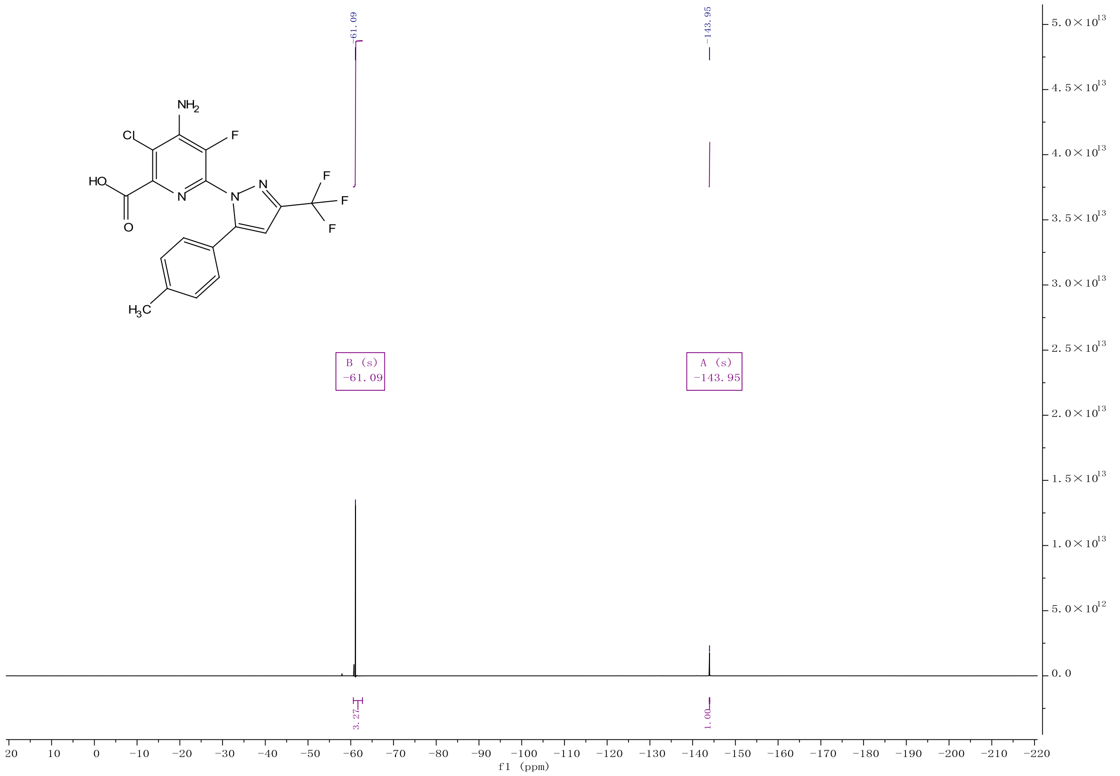

Figure S91. ^{19}F NMR (470.54 MHz, DMSO-d6) spectrum of compound S053.

Single Mass Analysis
Tolerance = 5.0 mDa / DBE: min = -1.5, max = 50.0
Element prediction: Off
Number of isotope peaks used for i-FIT = 3

Monoisotopic Mass, Even Electron Ions
6574 formula(e) evaluated with 1 results within limits (up to 50 best isotopic matches for each mass)
Elements Used:
C: 17-17 H: 11-11 N: 0-50 O: 0-50 F: 1-4 Na: 0-3 Cl: 1-2
4

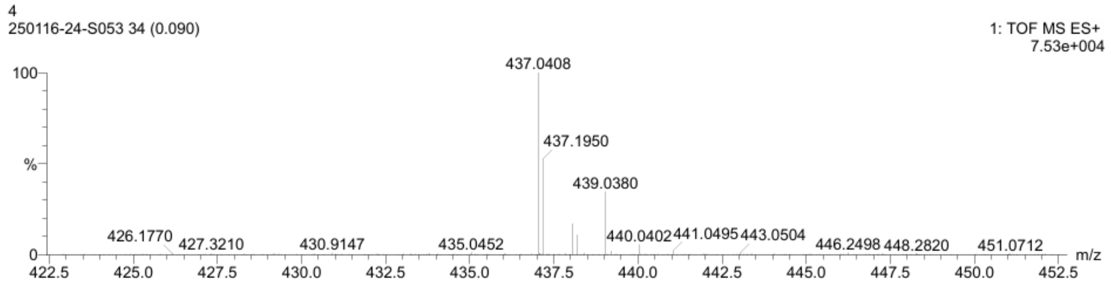

Minimum:				-1.5				
Maximum:		5.0	10.0	50.0				
Mass	Calc. Mass	mDa	PPM	DBE	i-FIT	Norm	Conf(%)	Formula
437.0408	437.0404	0.4	0.9	11.5	327.7	n/a	n/a	C17 H11 N4 O2 F4 Na Cl

Figure S92. HRMS spectrum of compound S053.

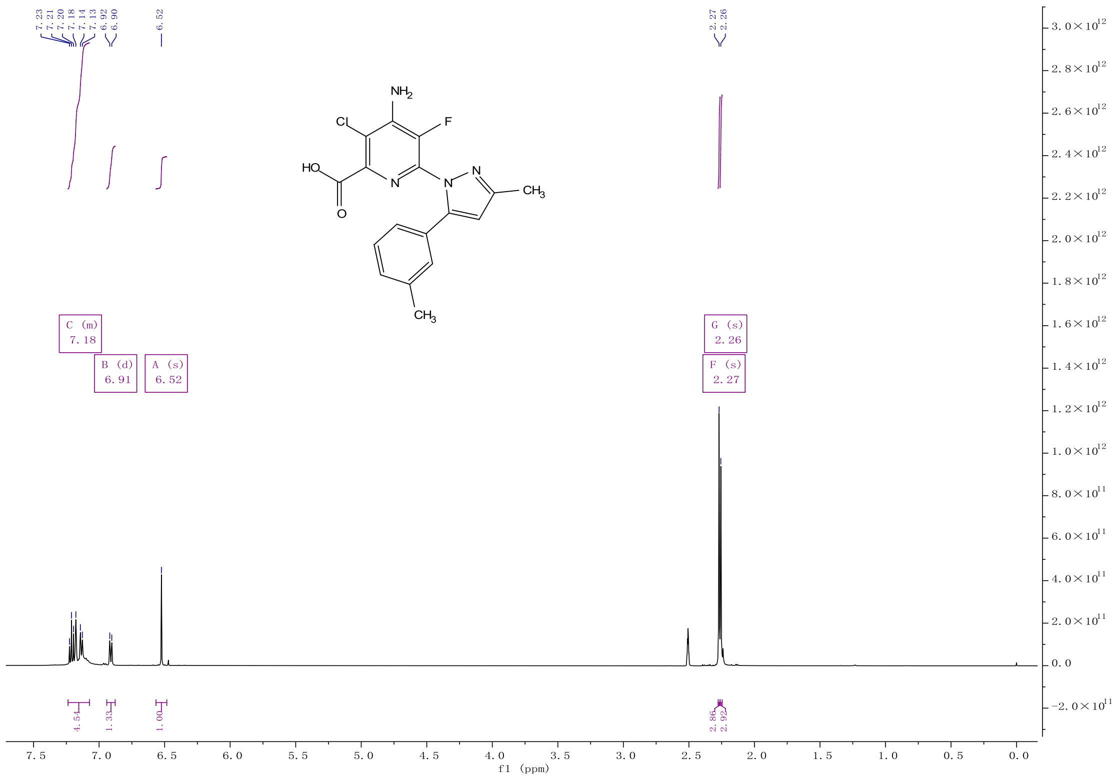

Figure S93. ^{1}H NMR (500.13 MHz, DMSO-d6) spectrum of compound S040.

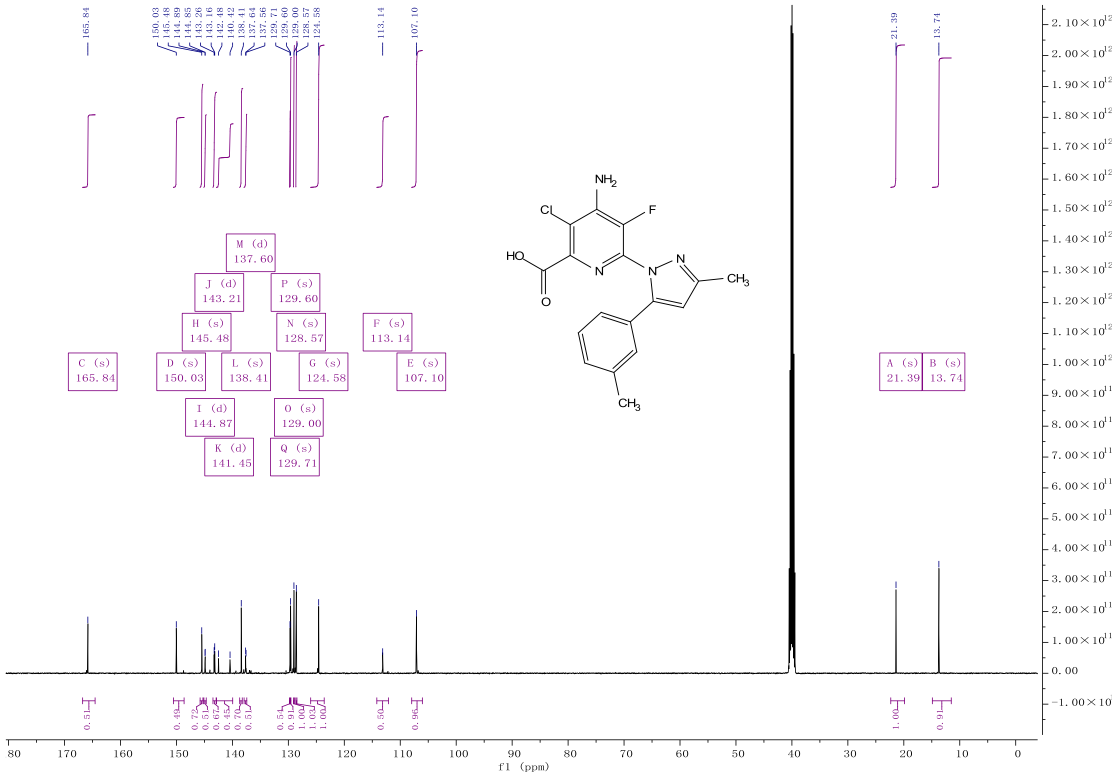

Figure S94. ^{13}C NMR (125.77 MHz, DMSO-d_6) spectrum of compound S040.

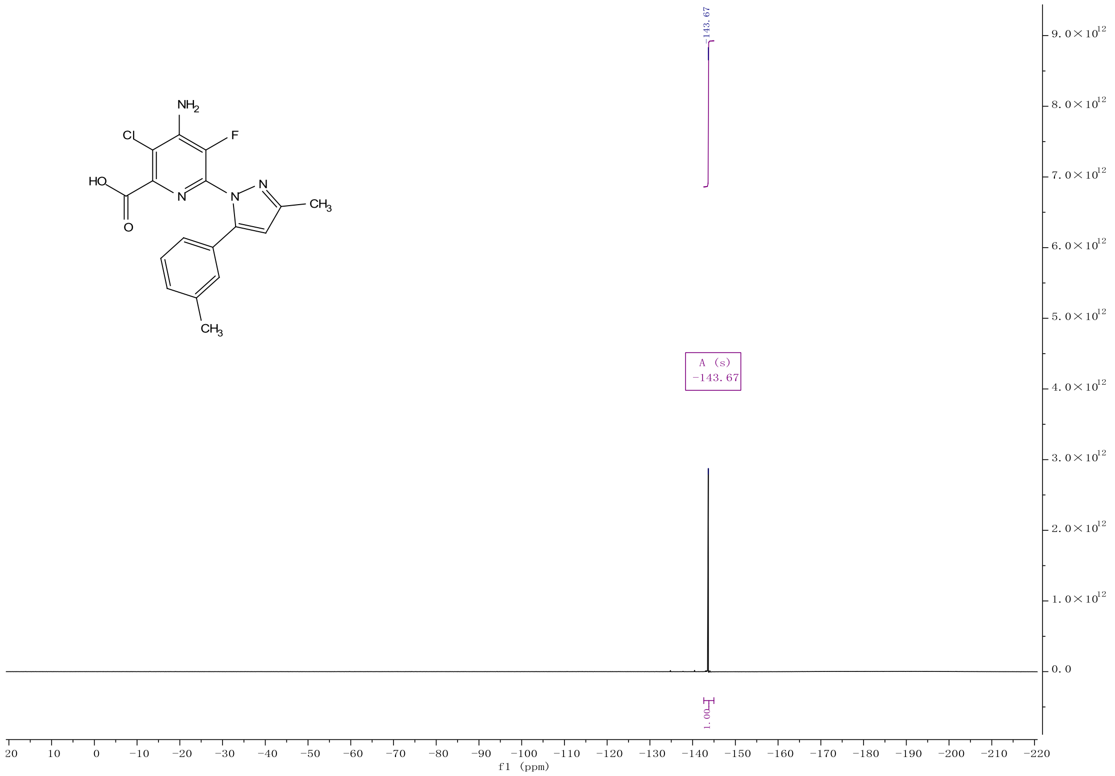

Figure S95. ^{19}F NMR (470.54 MHz, DMSO-d6) spectrum of compound S040.

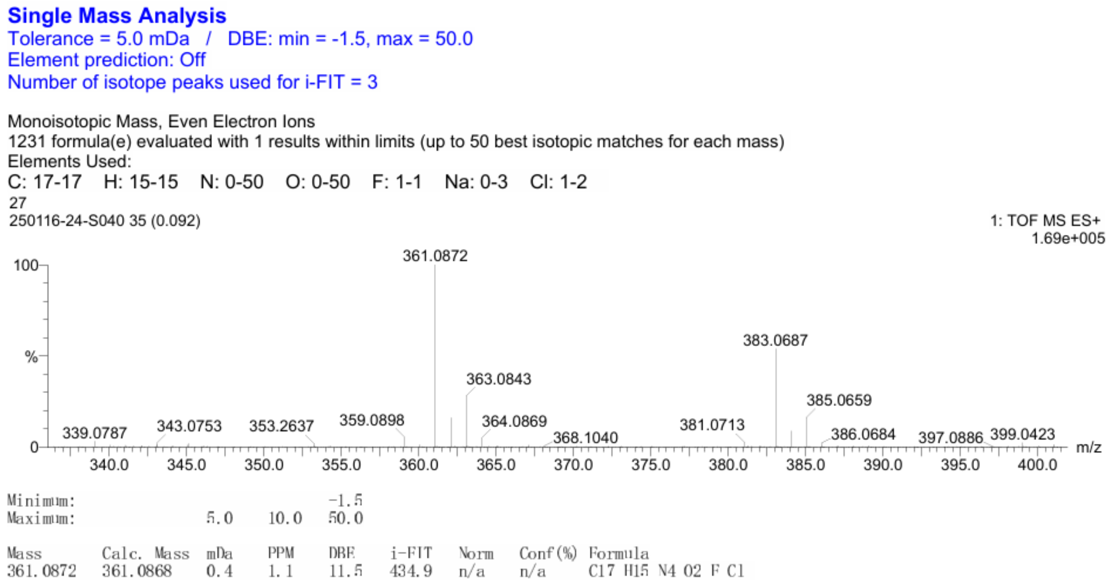

Figure S96. HRMS spectrum of compound S040.

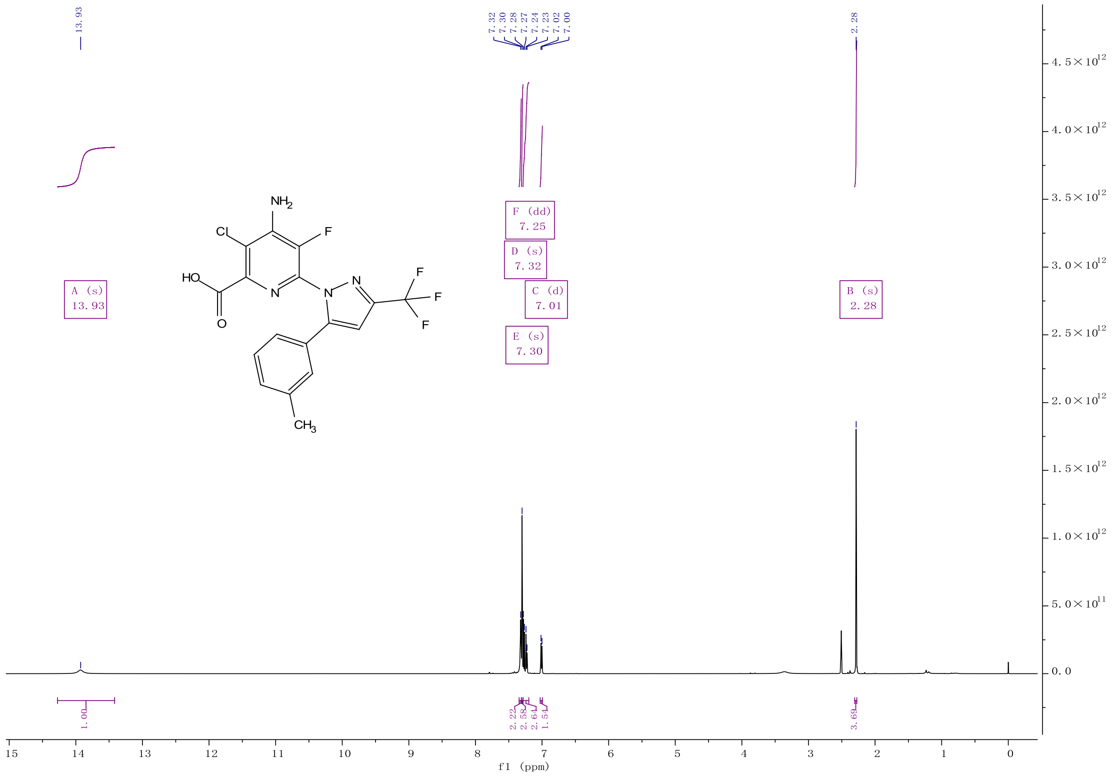

Figure S97. ^{1}H NMR (500.13 MHz, DMSO-d6) spectrum of compound S043.

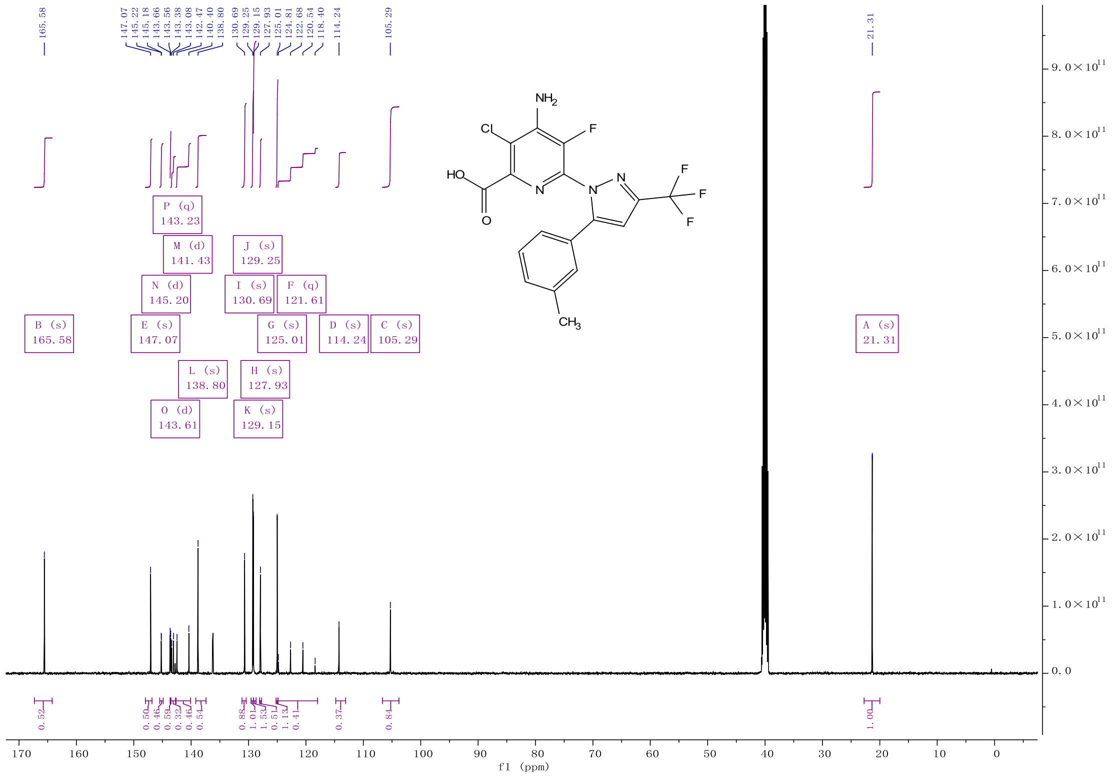

Figure S98. ^{13}C NMR (125.77 MHz, DMSO-d_6) spectrum of compound S043.

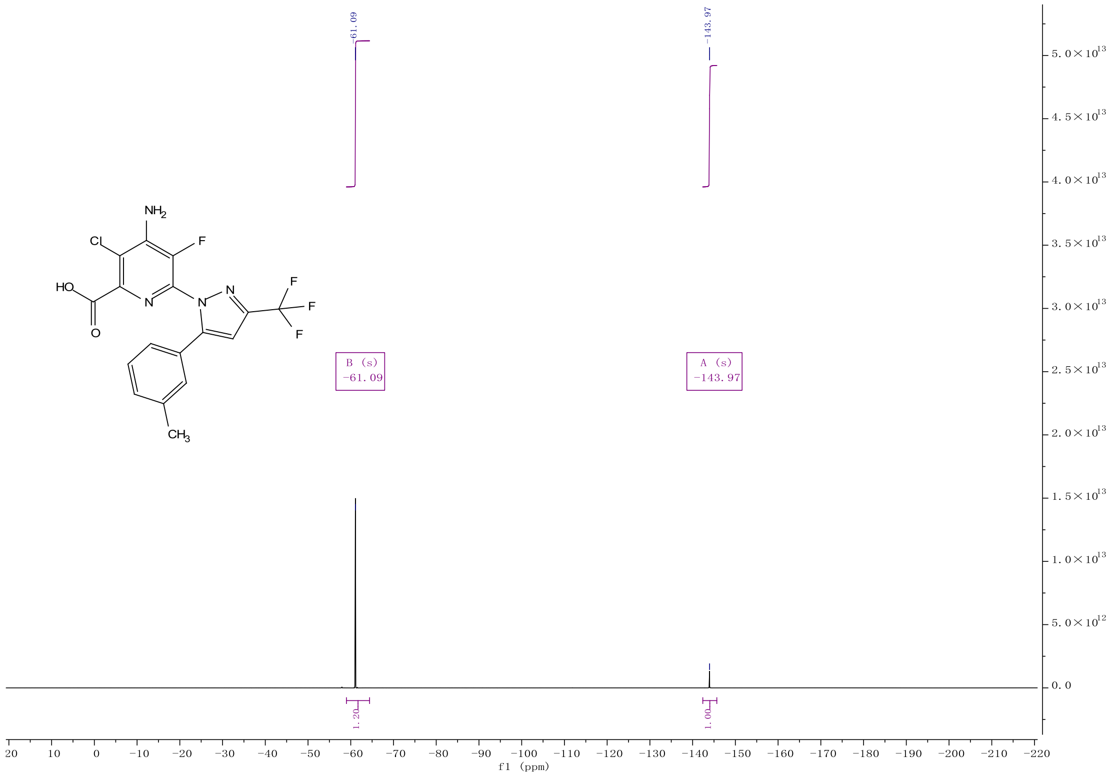

Figure S99. ^{19}F NMR (470.54 MHz, DMSO-d6) spectrum of compound S043.

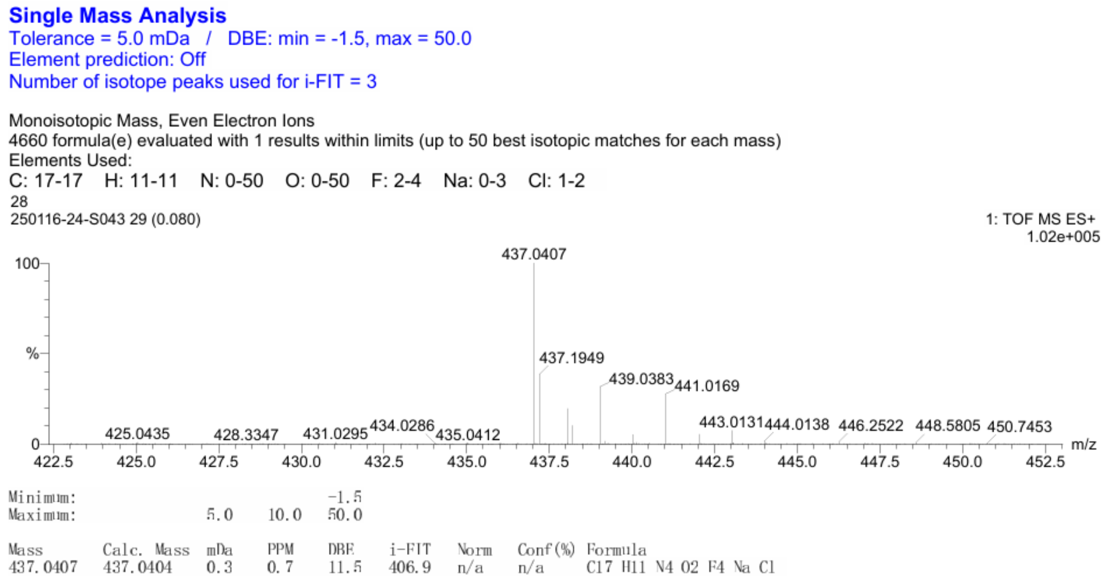

Figure S100. HRMS spectrum of compound S043.

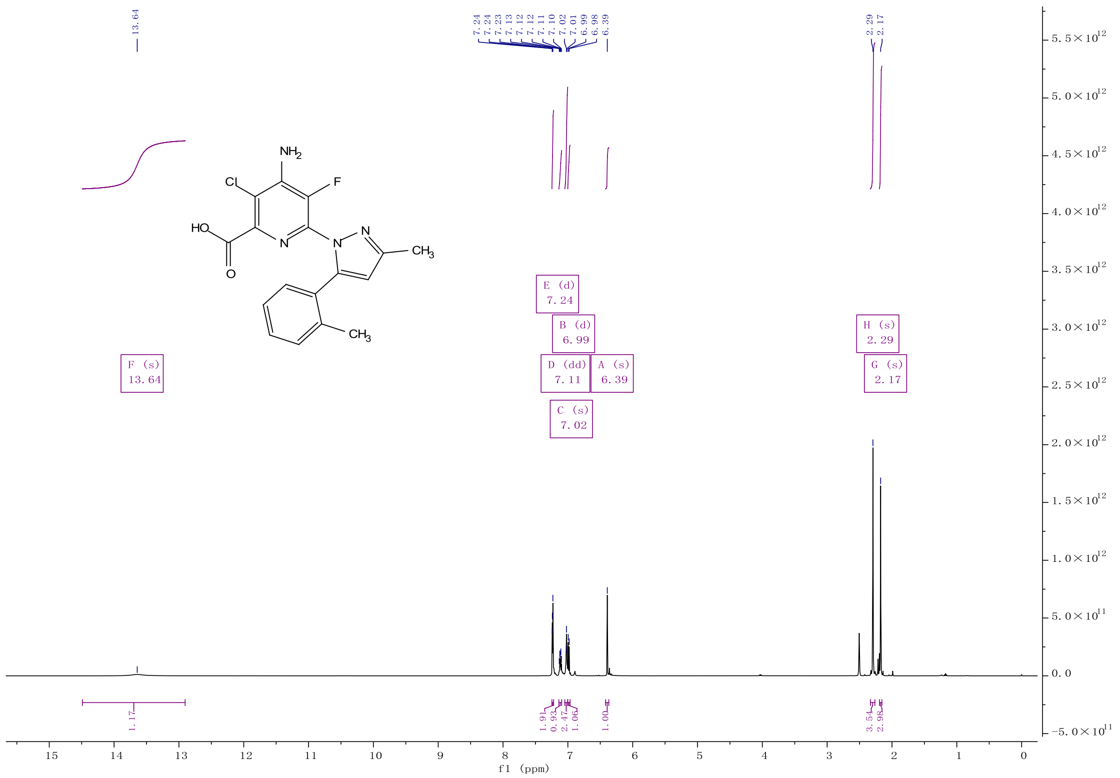

Figure S101. ^{1}H NMR (500.13 MHz, DMSO-d6) spectrum of compound S140.

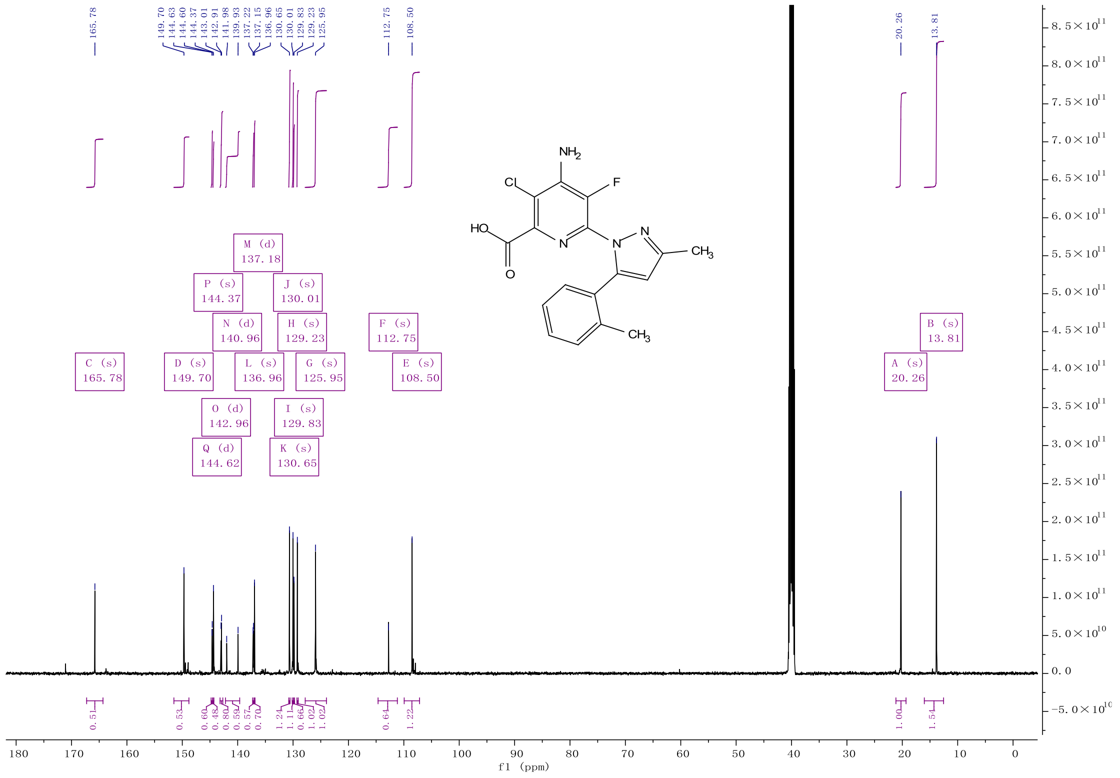

Figure S102. ^{13}C NMR (125.77 MHz, DMSO-d_6) spectrum of compound S140.

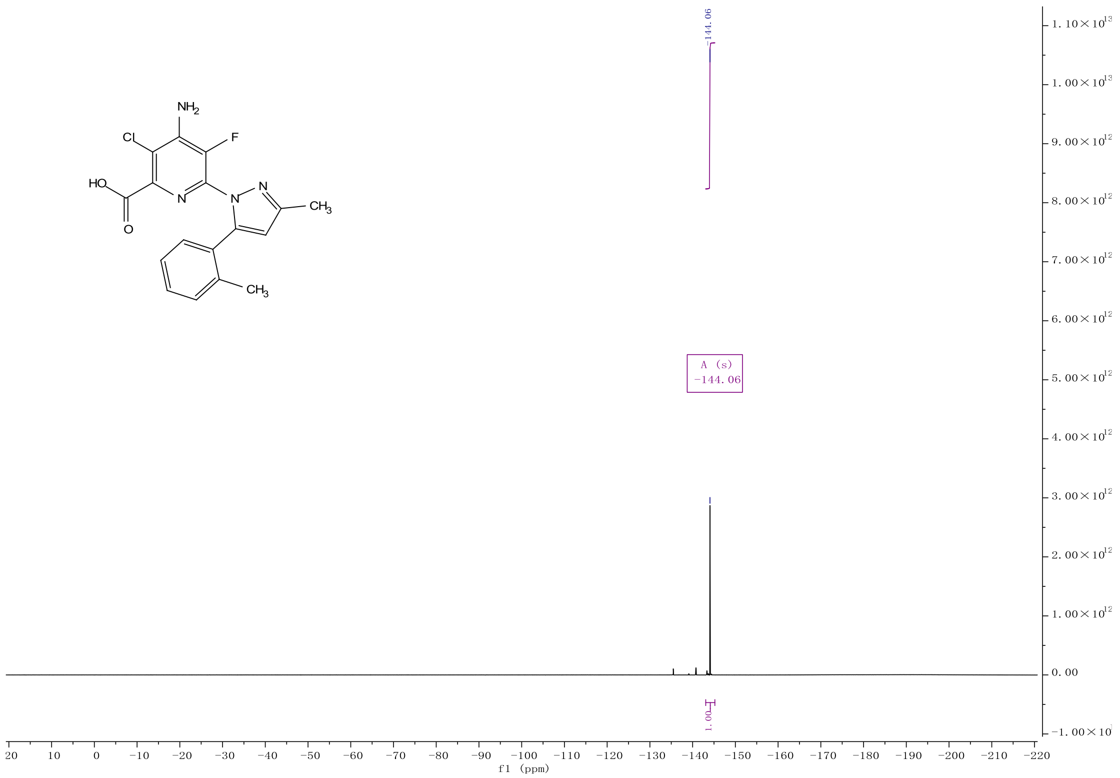

Figure S103. ^{19}F NMR (470.54 MHz, DMSO-d6) spectrum of compound S140.

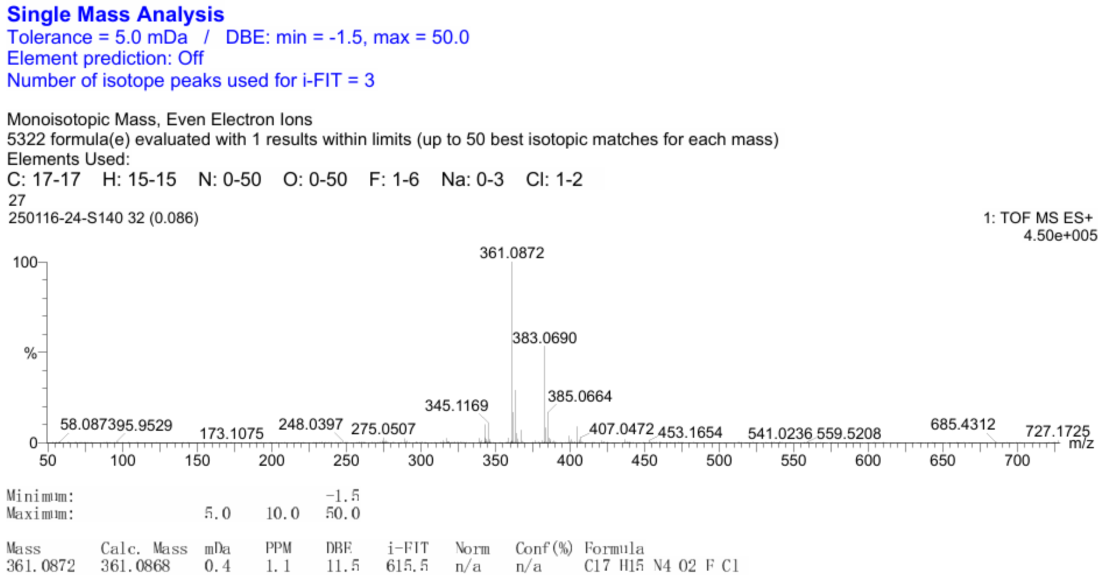

Figure S104. HRMS spectrum of compound S140.

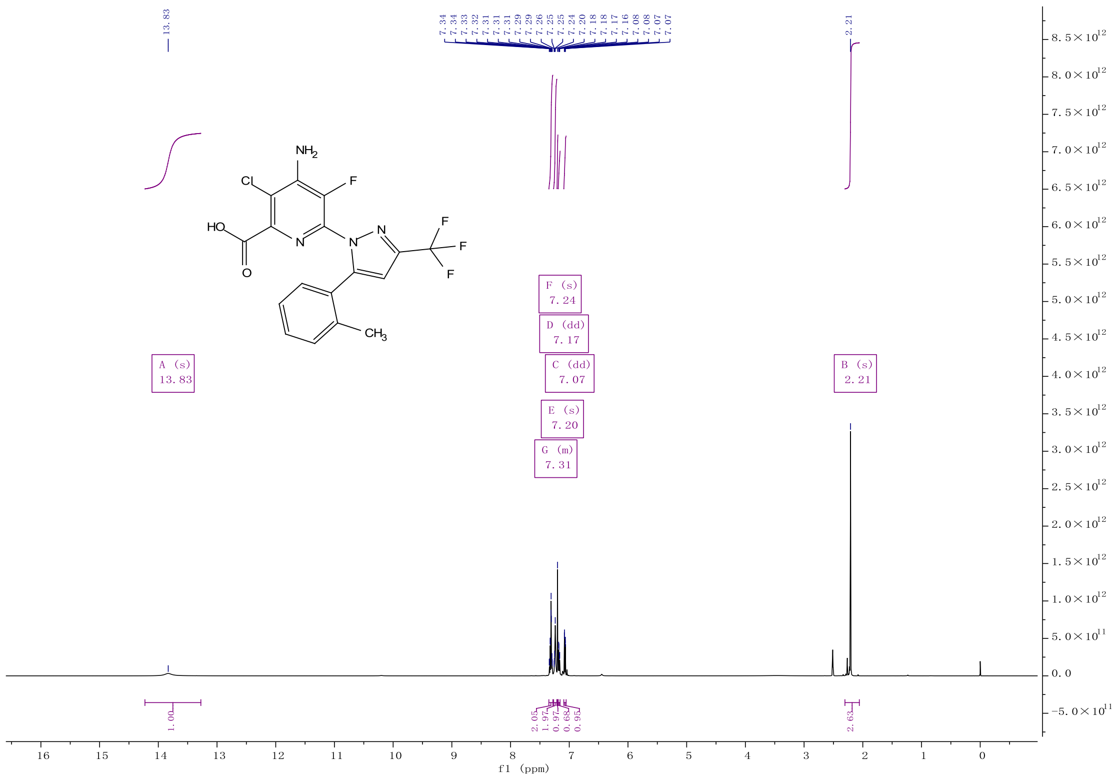

Figure S105. ^{1}H NMR (500.13 MHz, DMSO-d6) spectrum of compound S143.

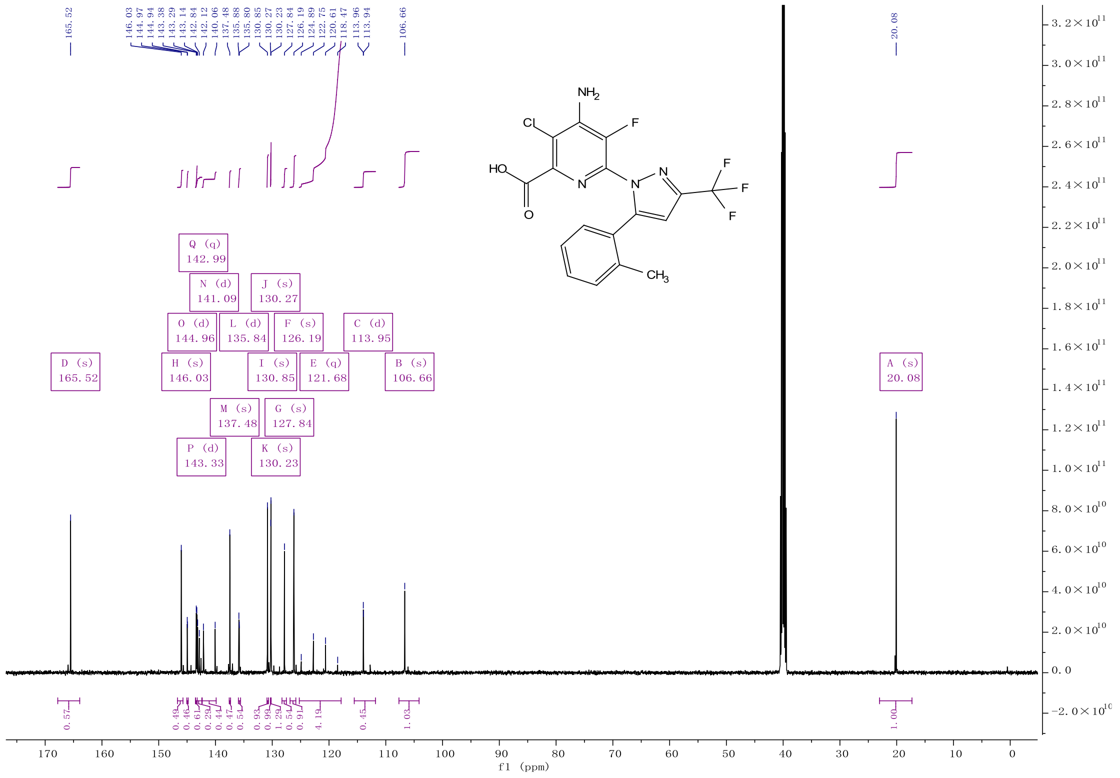

Figure S106. ^{13}C NMR (125.77 MHz, DMSO-d_6) spectrum of compound S143.

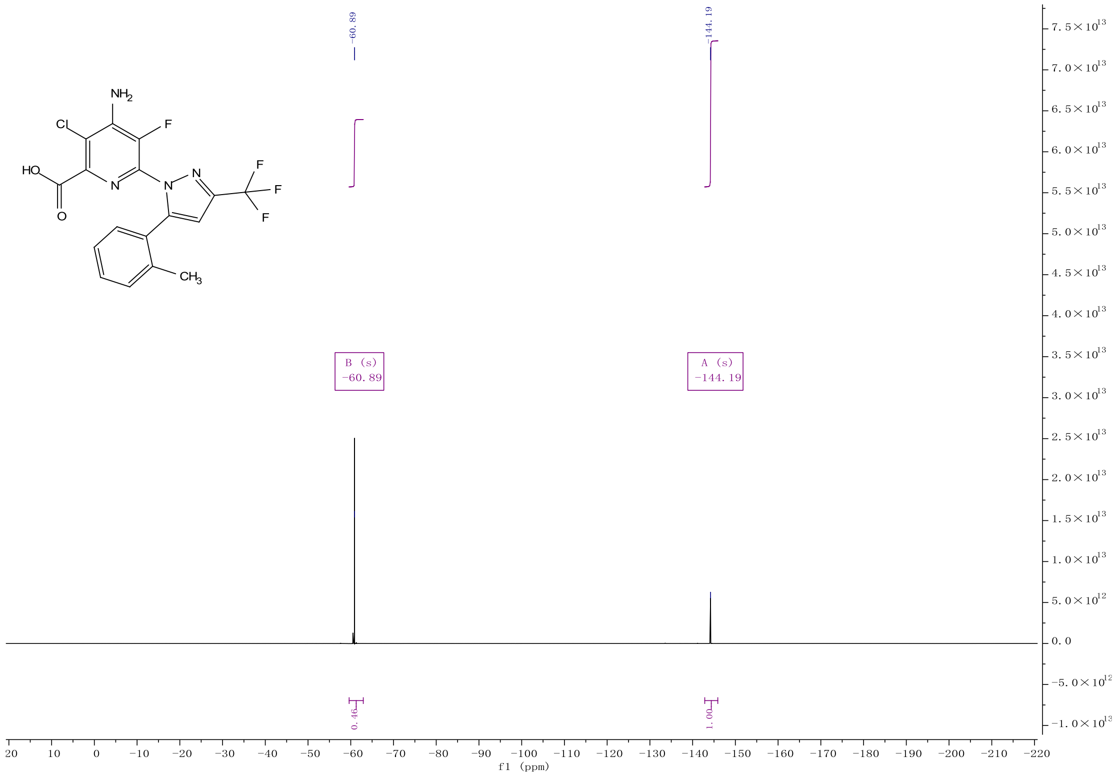

Figure S107. ^{19}F NMR (470.54 MHz, DMSO-d6) spectrum of compound S143.

Single Mass Analysis
Tolerance = 5.0 mDa / DBE: min = -1.5, max = 50.0
Element prediction: Off
Number of isotope peaks used for i-FIT = 3

Monoisotopic Mass, Even Electron Ions
4168 formula(e) evaluated with 1 results within limits (up to 50 best isotopic matches for each mass)
Elements Used:
C: 17-17 H: 11-11 N: 0-50 O: 0-50 F: 3-5 Na: 0-3 Cl: 1-2
4
250116-24-S143 27 (0.076)

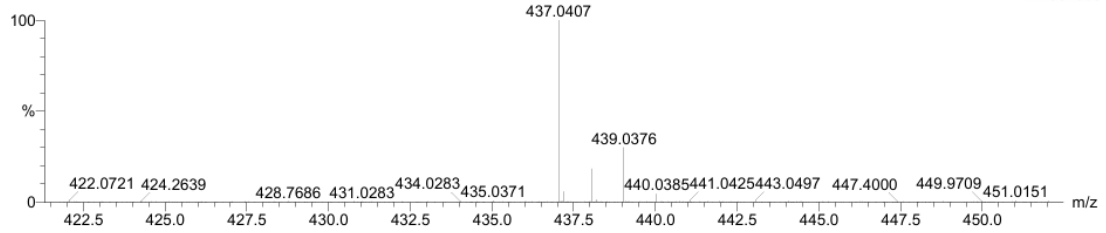

Minimum:				-1.5				
Maximum:		5.0	10.0	50.0				
Mass	Calc. Mass	mDa	PPM	DBE	i-FIT	Norm	Conf(%)	Formula
437.0407	437.0404	0.3	0.7	11.5	465.6	n/a	n/a	C17 H11 N4 O2 F4 Na Cl

Figure S108. HRMS spectrum of compound S143.

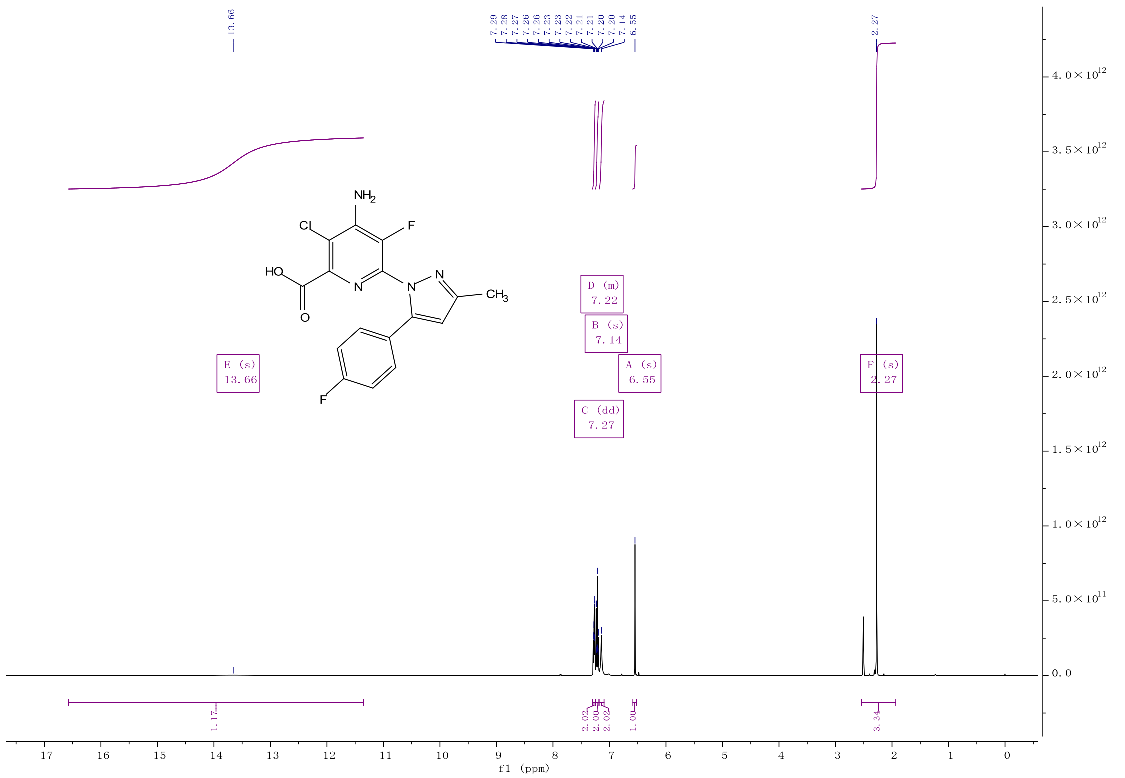

Figure S109. ^{1}H NMR (500.13 MHz, DMSO-d6) spectrum of compound S080.

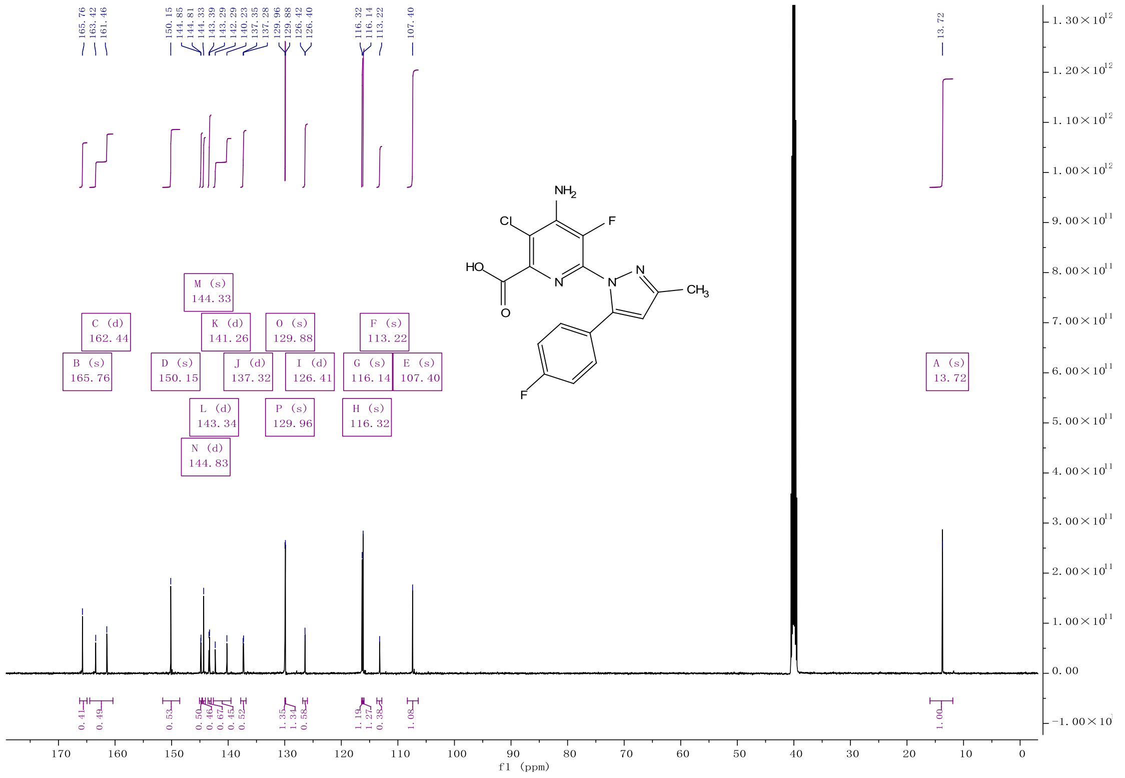

Figure S110. ^{13}C NMR (125.77 MHz, DMSO-d_6) spectrum of compound S080.

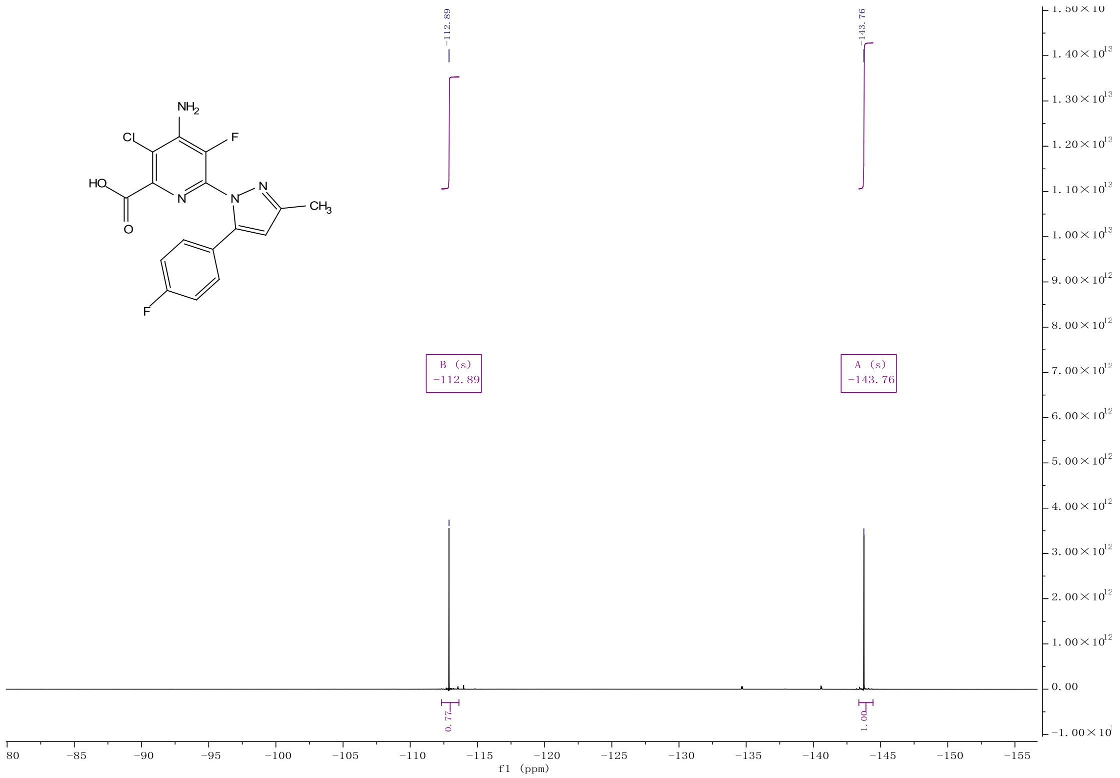

Figure S111. ^{19}F NMR (470.54 MHz, DMSO-d6) spectrum of compound S080.

Single Mass Analysis
Tolerance = 5.0 mDa / DBE: min = -1.5, max = 50.0
Element prediction: Off
Number of isotope peaks used for i-FIT = 3

Monoisotopic Mass, Even Electron Ions
5419 formula(e) evaluated with 1 results within limits (up to 50 best isotopic matches for each mass)
Elements Used:
C: 16-16 H: 12-12 N: 0-50 O: 0-50 F: 1-6 Na: 0-3 Cl: 1-2
27
250116-24-S080 49 (0.118) 1: TOF MS ES+
3.82e+004

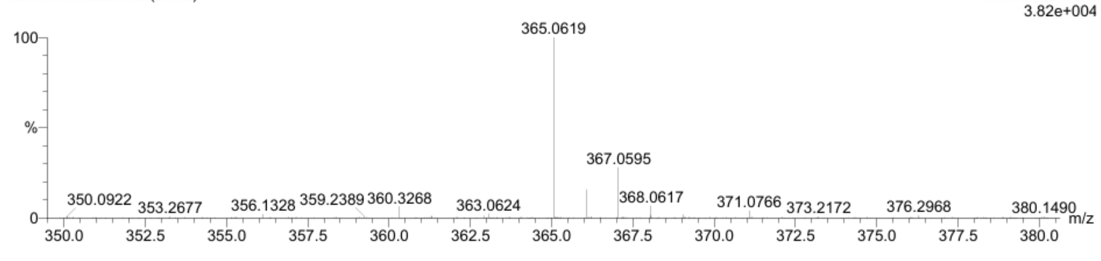

Minimum:				-1.5				
Maximum:		5.0	10.0	50.0				
Mass	Calc. Mass	mDa	PPM	DBE	i-FIT	Norm	Conf(%)	Formula
365.0619	365.0617	0.2	0.5	11.5	236.3	n/a	n/a	C16 H12 N4 O2 F2 Cl

Figure S112. HRMS spectrum of compound S080.

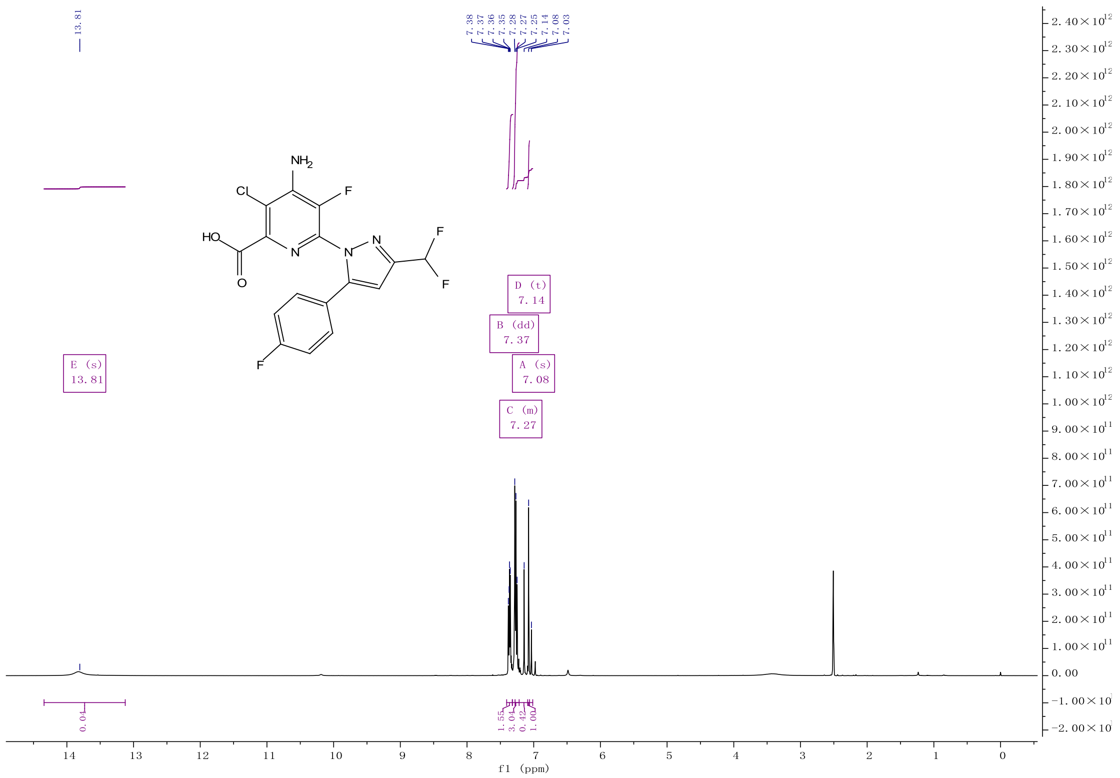

Figure S113. ^{1}H NMR (500.13 MHz, DMSO-d6) spectrum of compound S082.

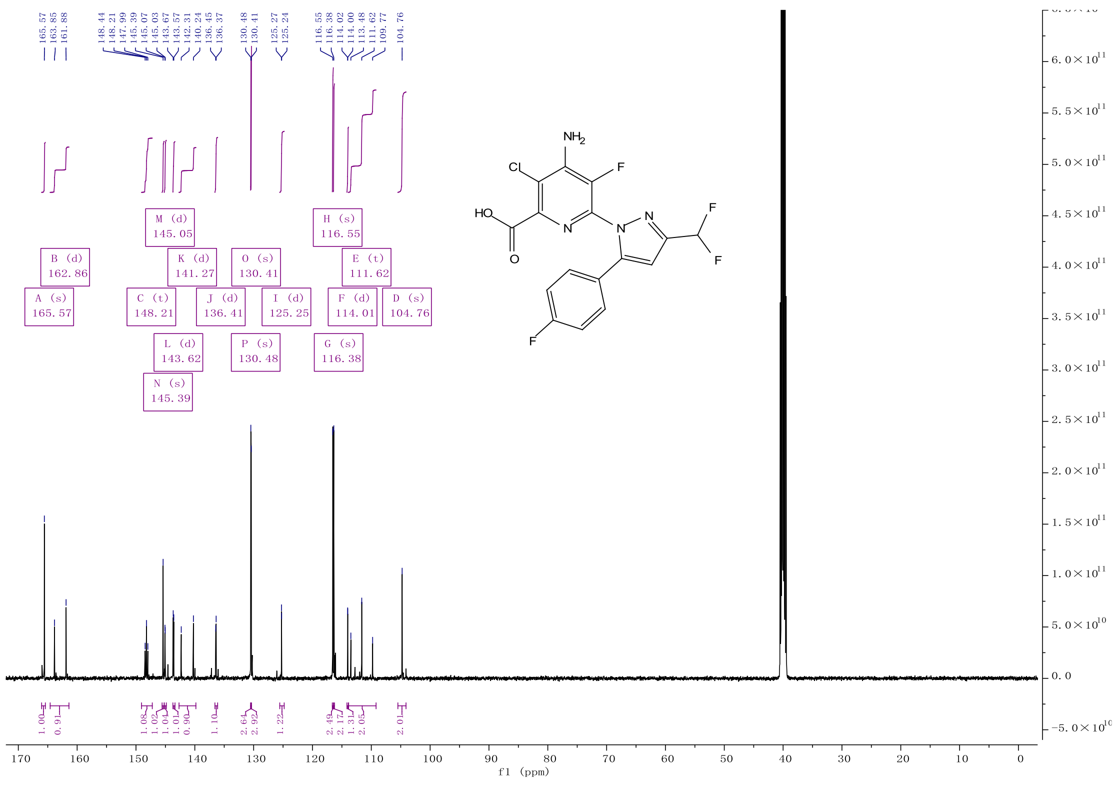

Figure S114. ^{13}C NMR (125.77 MHz, DMSO-d_6) spectrum of compound S082.

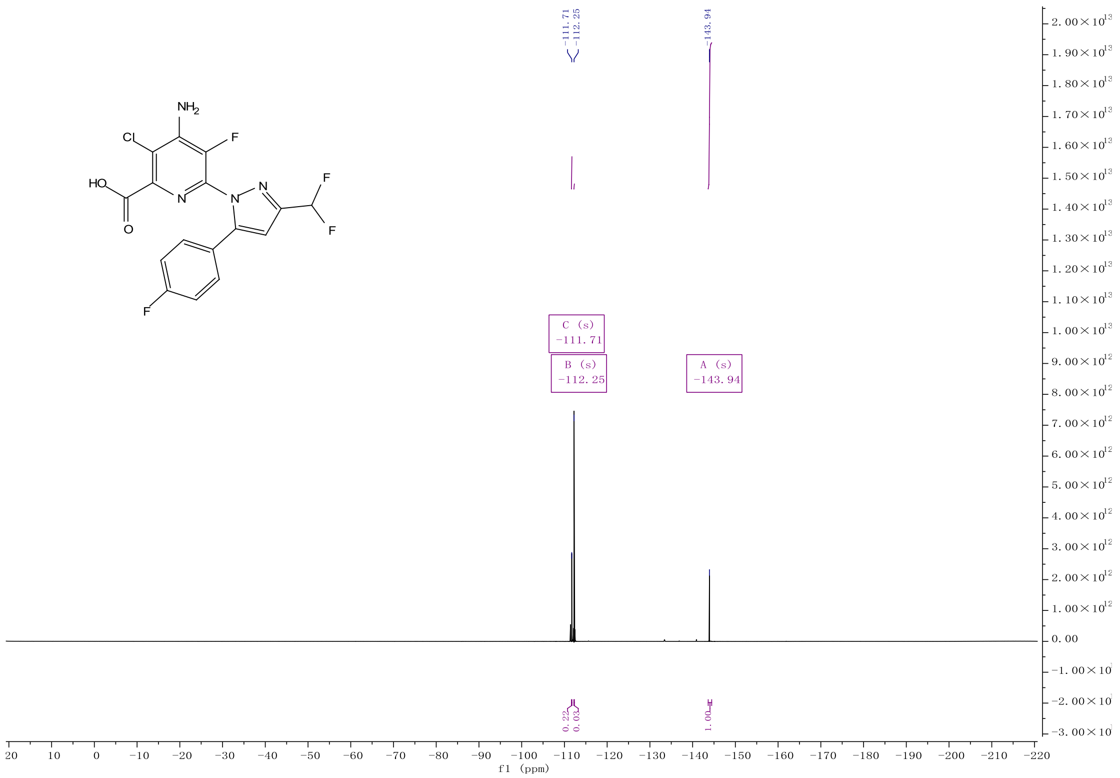

Figure S115. ^{19}F NMR (470.54 MHz, DMSO-d6) spectrum of compound S082.

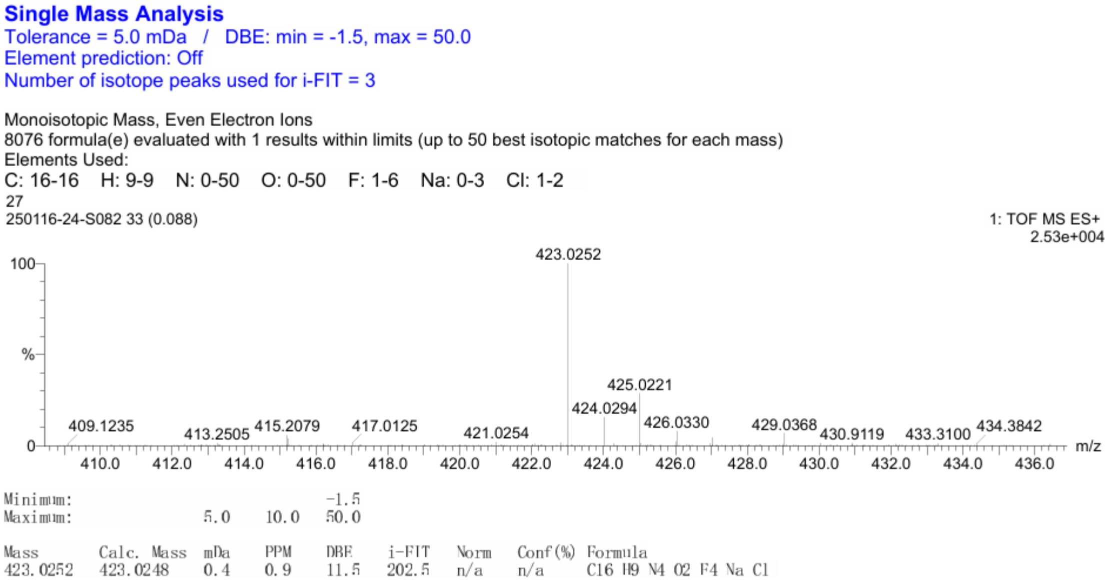

Figure S116. HRMS spectrum of compound S082.

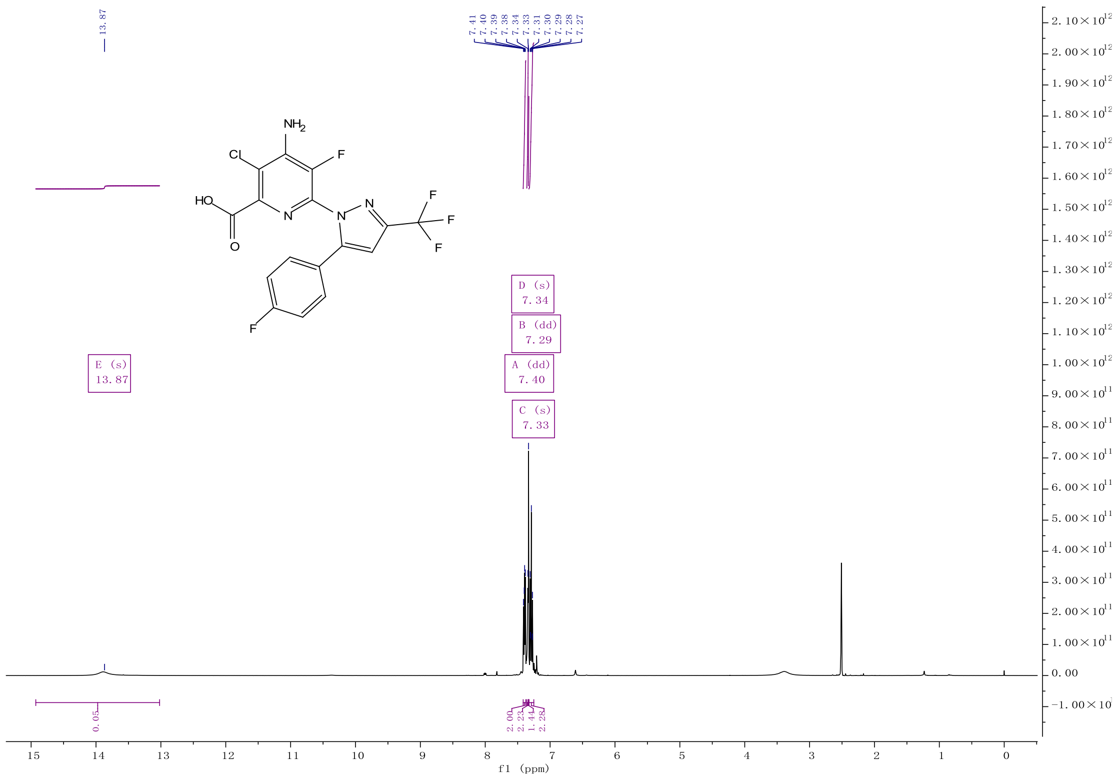

Figure S117. ^{1}H NMR (500.13 MHz, DMSO-d6) spectrum of compound S083.

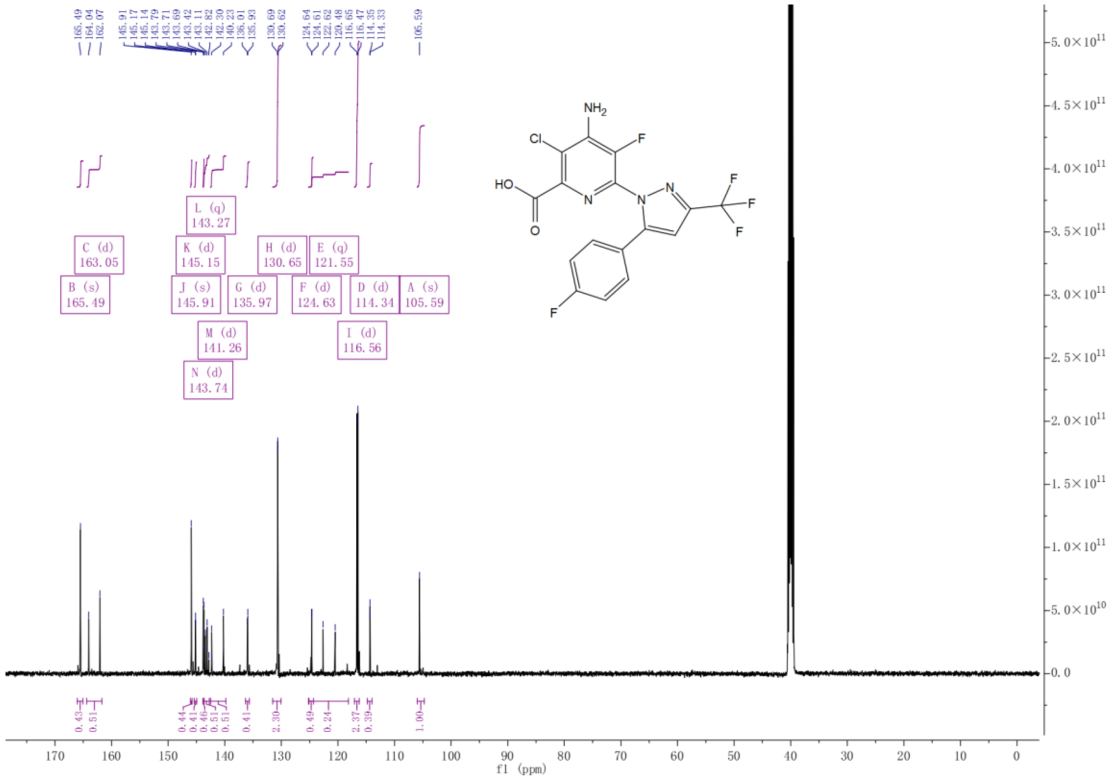

Figure S118. ^{13}C NMR (125.77 MHz, DMSO-d_6) spectrum of compound S083.

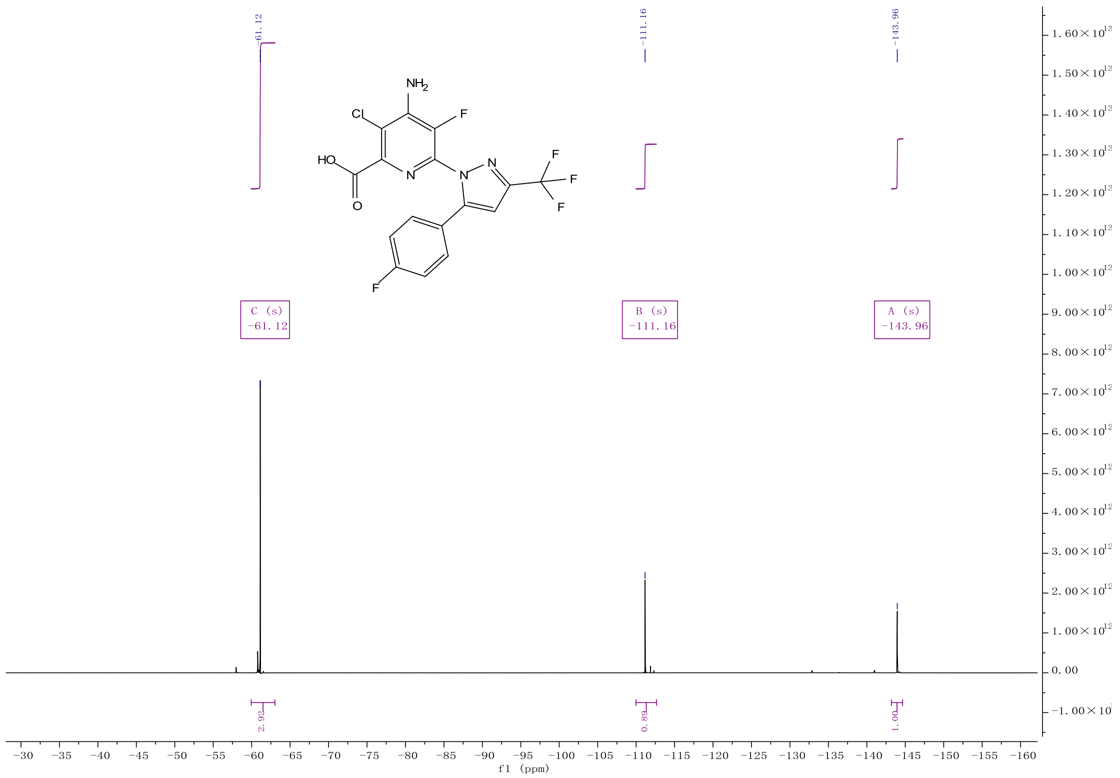

Figure S119. ^{19}F NMR (470.54 MHz, DMSO-d6) spectrum of compound S083.

Single Mass Analysis
Tolerance = 5.0 mDa / DBE: min = -1.5, max = 50.0
Element prediction: Off
Number of isotope peaks used for i-FIT = 3

Monoisotopic Mass, Even Electron Ions
2346 formula(e) evaluated with 1 results within limits (up to 50 best isotopic matches for each mass)
Elements Used:
C: 16-16 H: 8-8 N: 0-50 O: 0-50 F: 5-6 Na: 0-3 Cl: 1-2

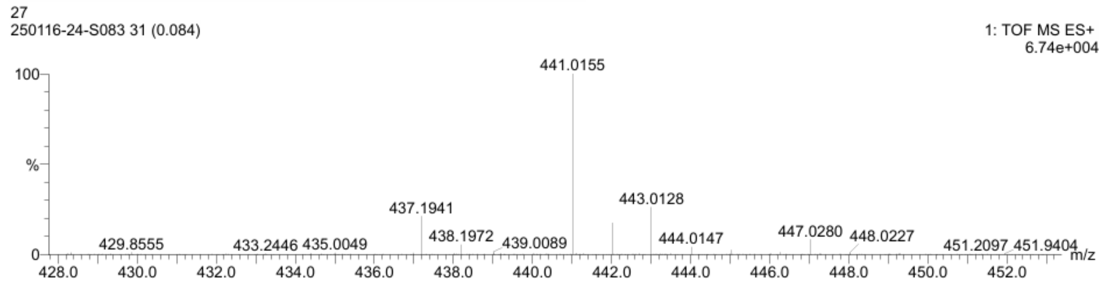

Minimum:				-1.5				
Maximum:		5.0	10.0	50.0				
Mass	Calc. Mass	mDa	PPM	DBE	i-FIT	Norm	Conf(%)	Formula
441.0155	441.0154	0.1	0.2	11.5	346.2	n/a	n/a	C16 H8 N4 O2 F5 Na Cl

Figure S120. HRMS spectrum of compound S083.

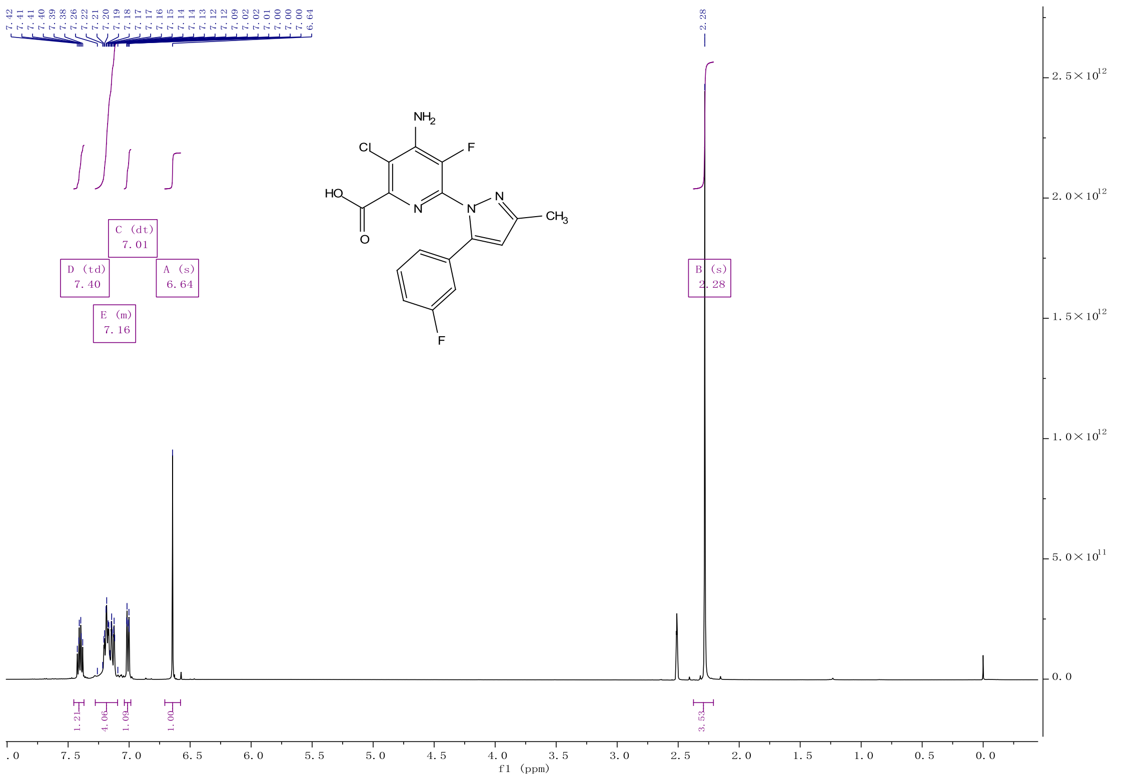

Figure S121. ^{1}H NMR (500.13 MHz, DMSO-d6) spectrum of compound S170.

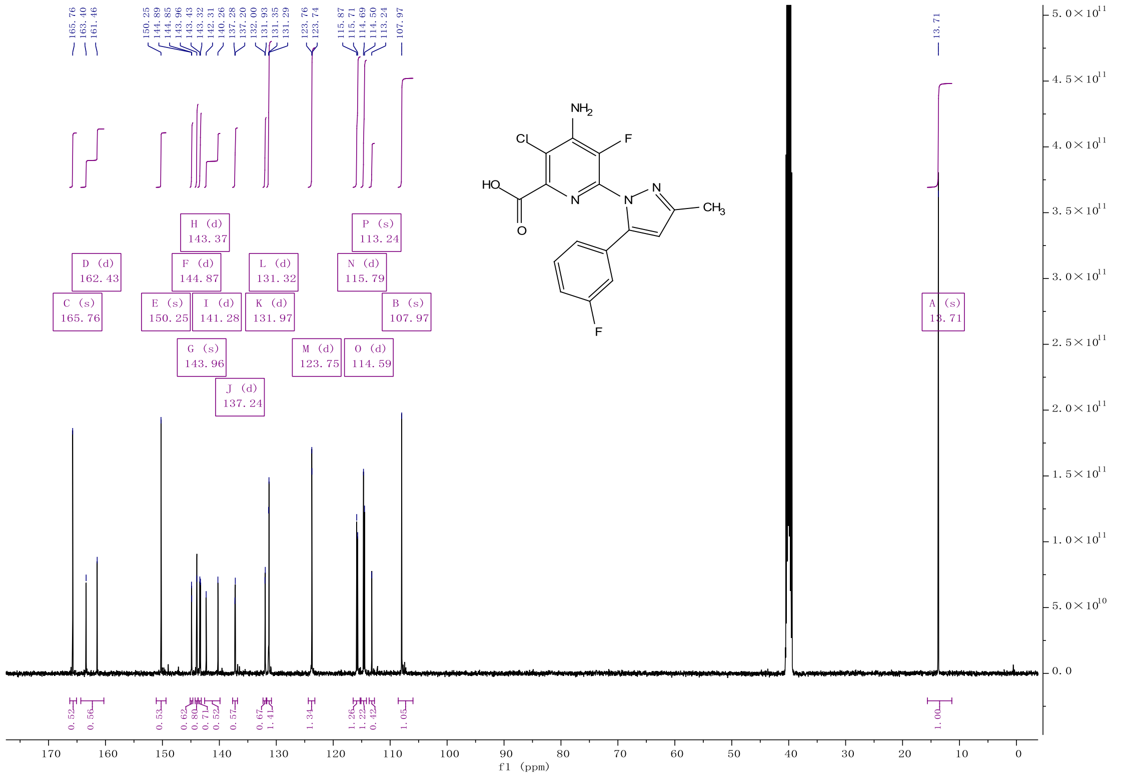

Figure S122. ^{13}C NMR (125.77 MHz, DMSO-d_6) spectrum of compound S170.

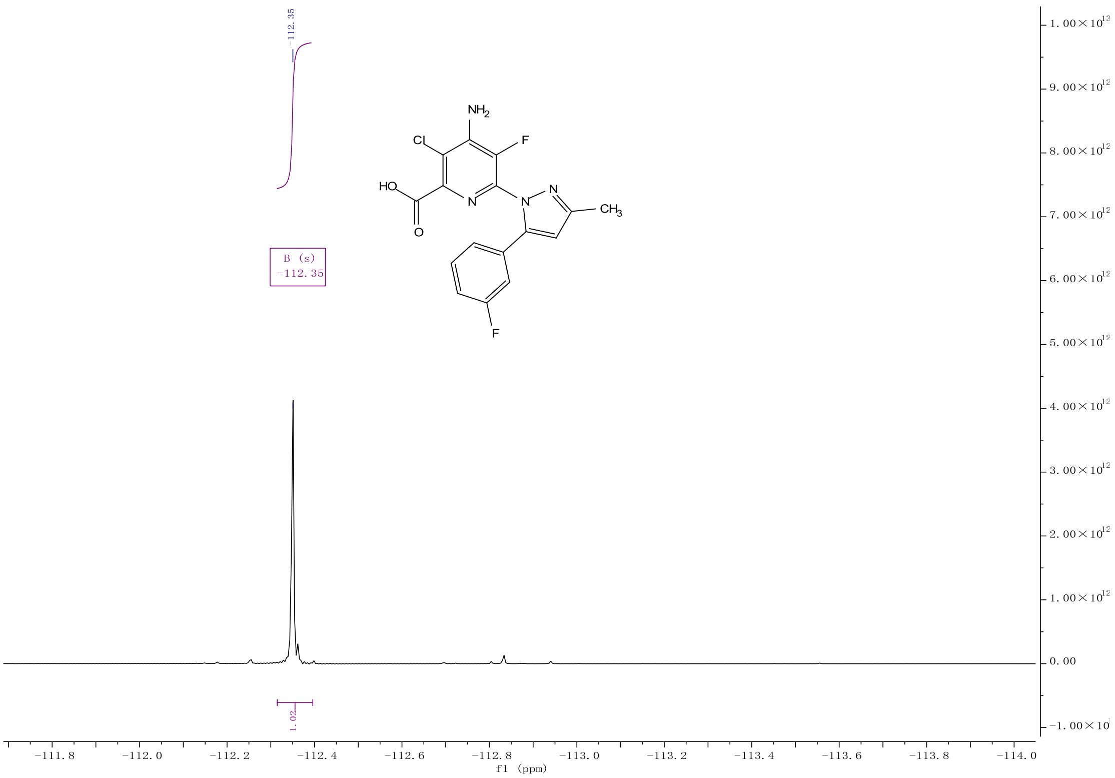

Figure S123. ^{19}F NMR (470.54 MHz, DMSO-d6) spectrum of compound S170.

Single Mass Analysis
Tolerance = 5.0 mDa / DBE: min = -1.5, max = 50.0
Element prediction: Off
Number of isotope peaks used for i-FIT = 3

Monoisotopic Mass, Even Electron Ions
1097 formula(e) evaluated with 1 results within limits (up to 50 best isotopic matches for each mass)
Elements Used:
C: 16-16 H: 12-12 N: 0-50 O: 0-50 F: 2-2 Na: 0-3 Cl: 1-2
28
250116-24-S170 34 (0.090)

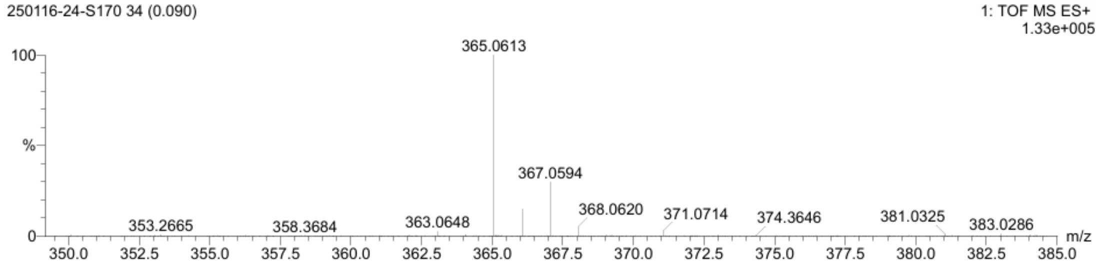

Minimum:				-1.5				
Maximum:		5.0	10.0	50.0				
Mass	Calc. Mass	mDa	PPM	DBE	i-FIT	Norm	Conf(%)	Formula
365.0613	365.0617	-0.4	-1.1	11.5	347.1	n/a	n/a	C16 H12 N4 O2 F2 Cl

Figure S124. HRMS spectrum of compound S170.

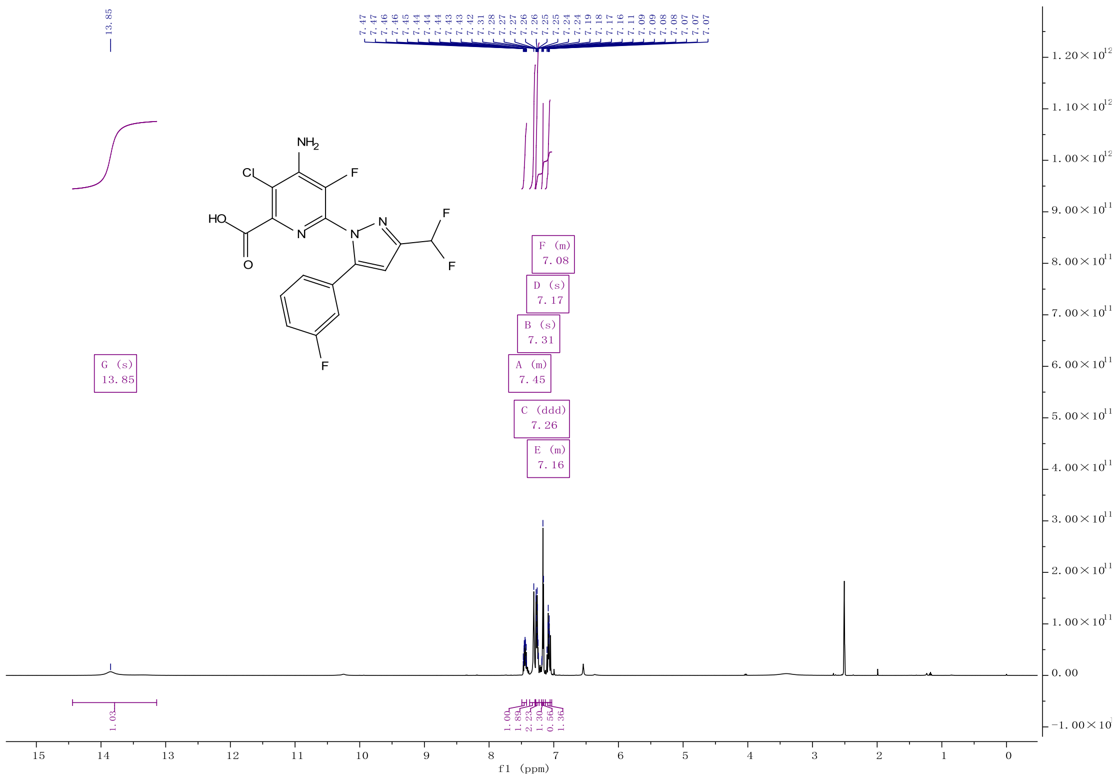

Figure S125. ^{1}H NMR (500.13 MHz, DMSO-d6) spectrum of compound S172.

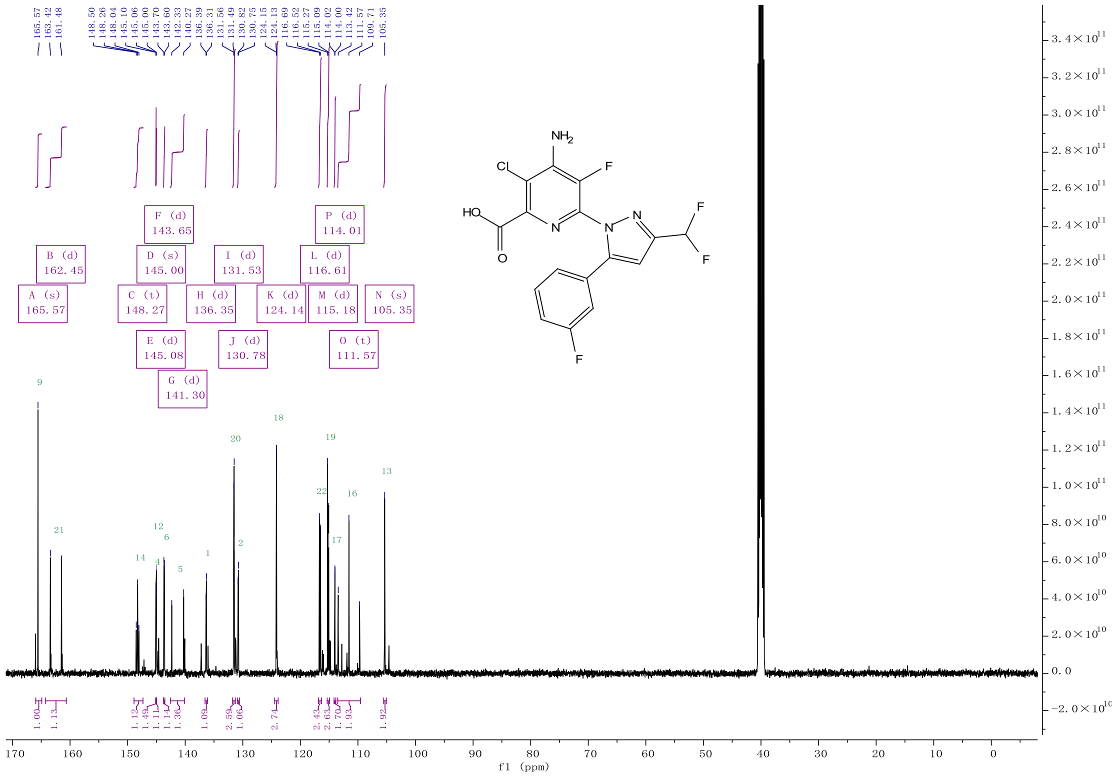

Figure S126. ^{13}C NMR (125.77 MHz, DMSO-d_6) spectrum of compound S172.

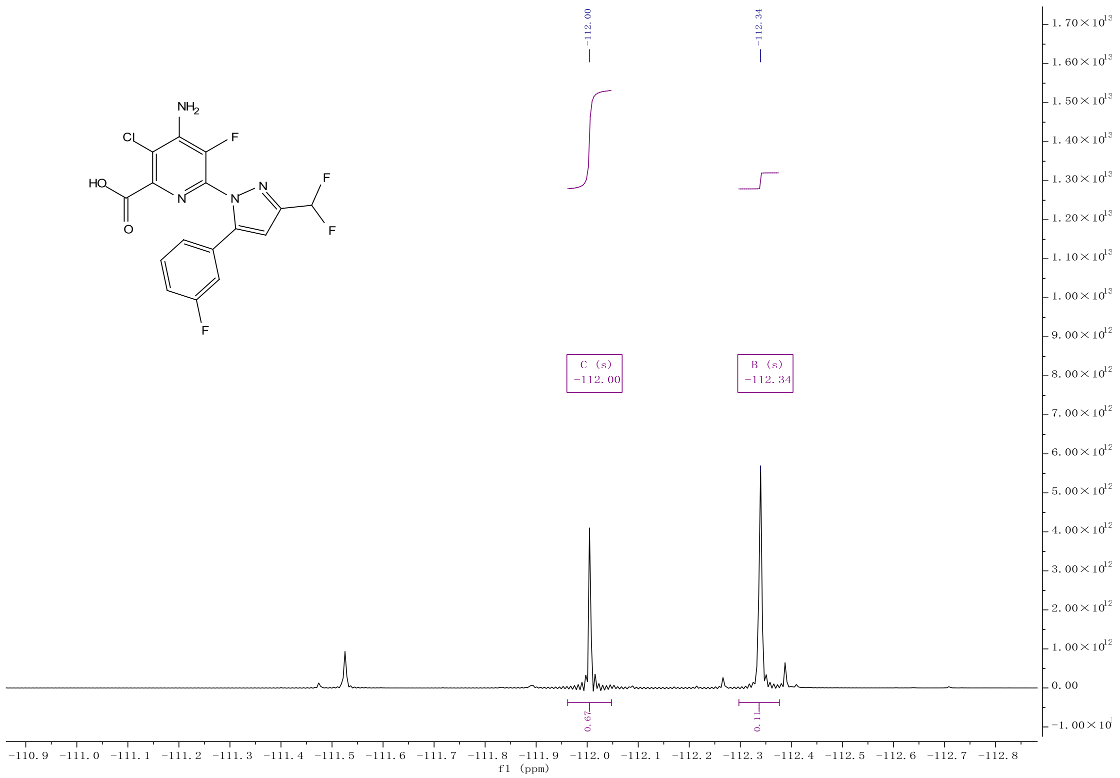

Figure S127. ^{19}F NMR (470.54 MHz, DMSO-d6) spectrum of compound S172.

Single Mass Analysis
Tolerance = 5.0 mDa / DBE: min = -1.5, max = 50.0
Element prediction: Off
Number of isotope peaks used for i-FIT = 3

Monoisotopic Mass, Even Electron Ions
2664 formula(e) evaluated with 1 results within limits (up to 50 best isotopic matches for each mass)
Elements Used:
C: 16-16 H: 9-9 N: 0-50 O: 0-50 F: 3-4 Na: 0-3 Cl: 1-2
27
250116-24-S172 31 (0.084)

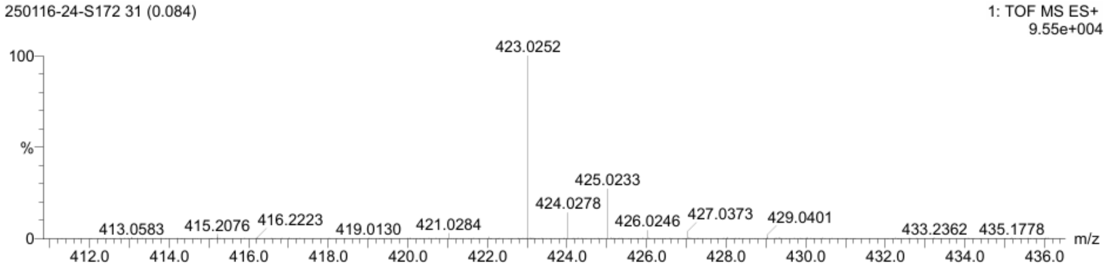

	Mass	Calc. Mass	mDa	PPM	DBE	i-FIT	Norm	Conf(%)	Formula
Minimum:					-1.5				
Maximum:			5.0	10.0	50.0				
	423.0252	423.0248	0.4	0.9	11.5	318.6	n/a	n/a	C16 H9 N4 O2 F4 Na Cl

Figure S128. HRMS spectrum of compound S172.

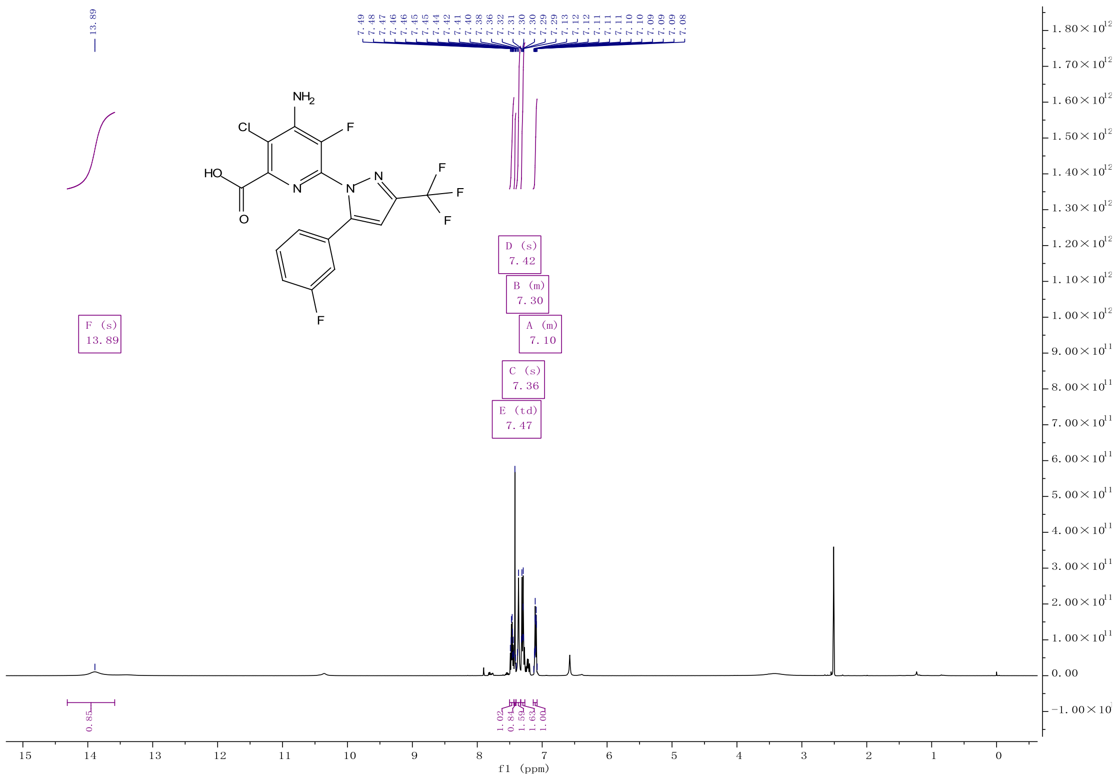

Figure S129. ^{1}H NMR (500.13 MHz, DMSO-d6) spectrum of compound S173.

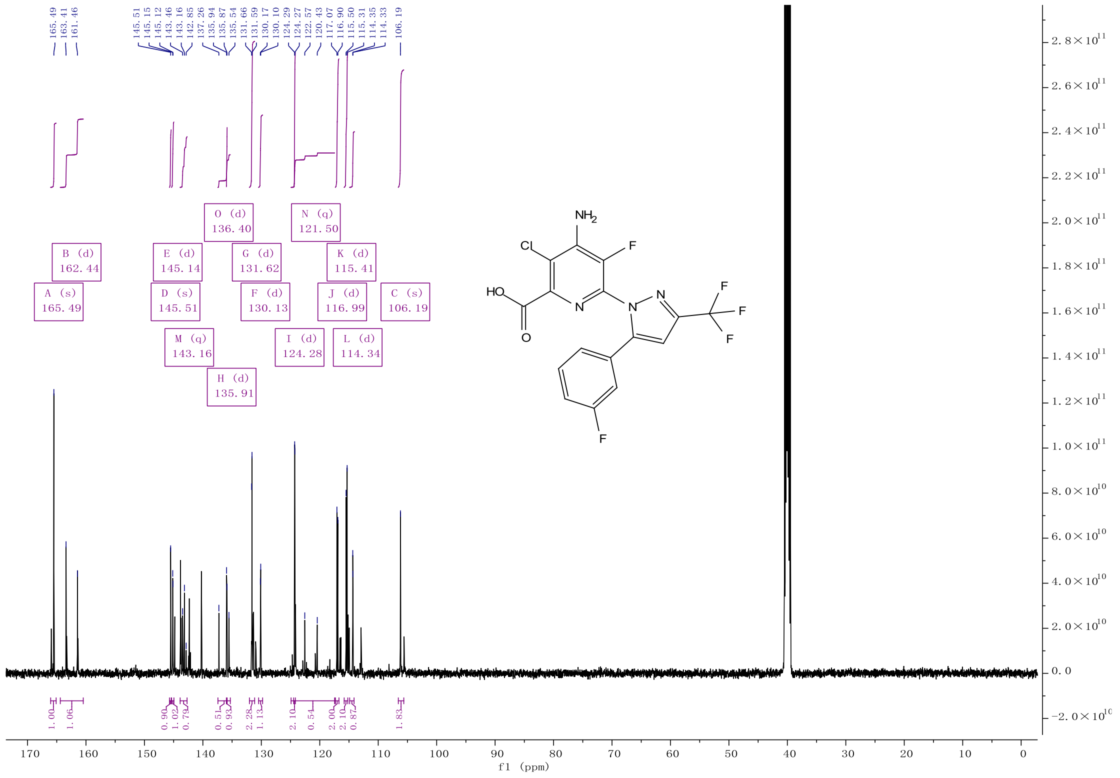

Figure S130. ^{13}C NMR (125.77 MHz, DMSO-d_6) spectrum of compound S173.

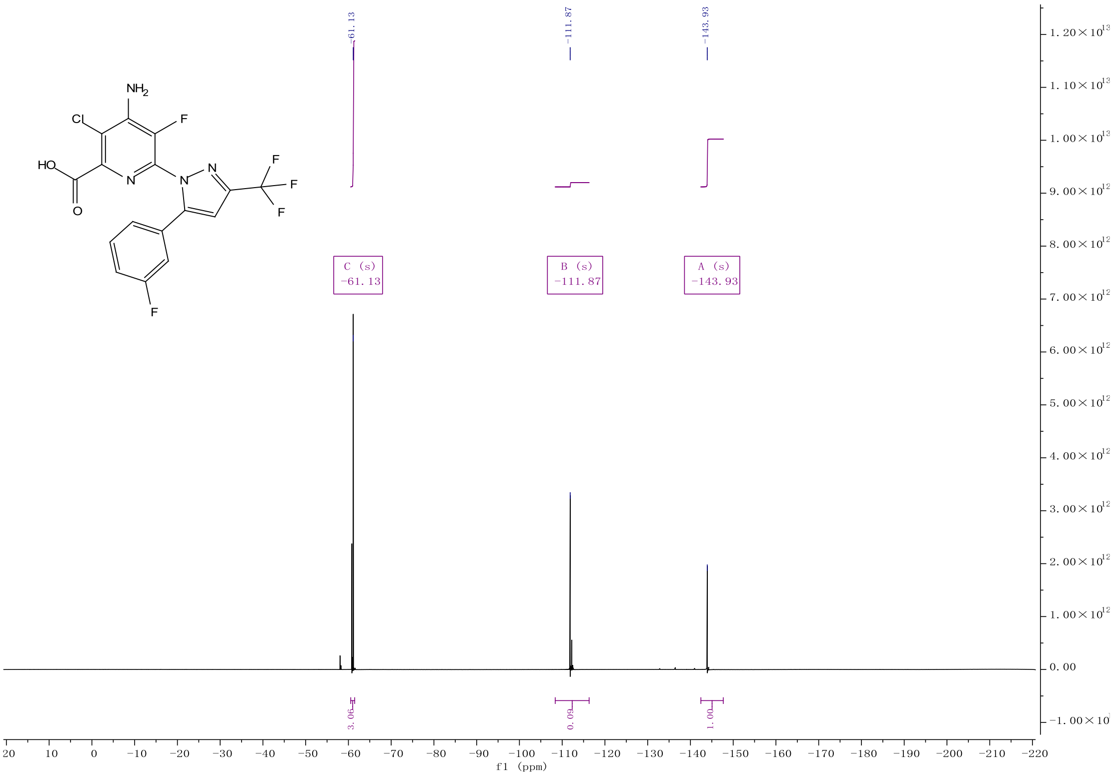

Figure S131. ^{19}F NMR (470.54 MHz, DMSO-d6) spectrum of compound S173.

Single Mass Analysis
Tolerance = 5.0 mDa / DBE: min = -1.5, max = 50.0
Element prediction: Off
Number of isotope peaks used for i-FIT = 3

Monoisotopic Mass, Even Electron Ions
1247 formula(e) evaluated with 1 results within limits (up to 50 best isotopic matches for each mass)
Elements Used:
C: 16-16 H: 8-8 N: 0-50 O: 0-50 F: 5-5 Na: 0-3 Cl: 1-2
27
250116-24-S173 27 (0.076)

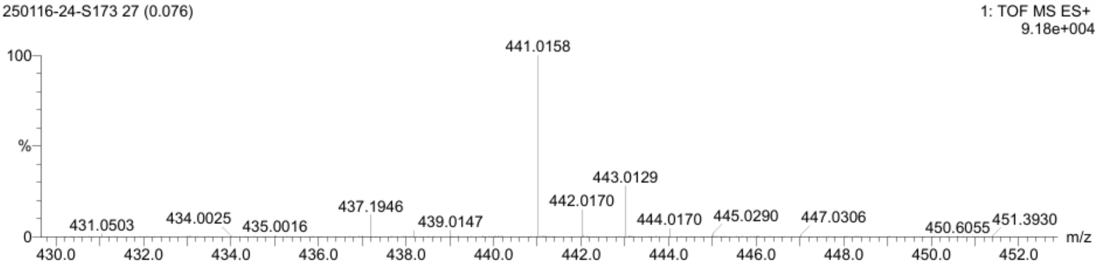

Minimum:				-1.5				
Maximum:		5.0	10.0	50.0				
Mass	Calc. Mass	mDa	PPM	DBE	i-FIT	Norm	Conf(%)	Formula
441.0158	441.0154	0.4	0.9	11.5	331.6	n/a	n/a	C16 H8 N4 O2 F5 Na Cl

Figure S132. HRMS spectrum of compound S173.

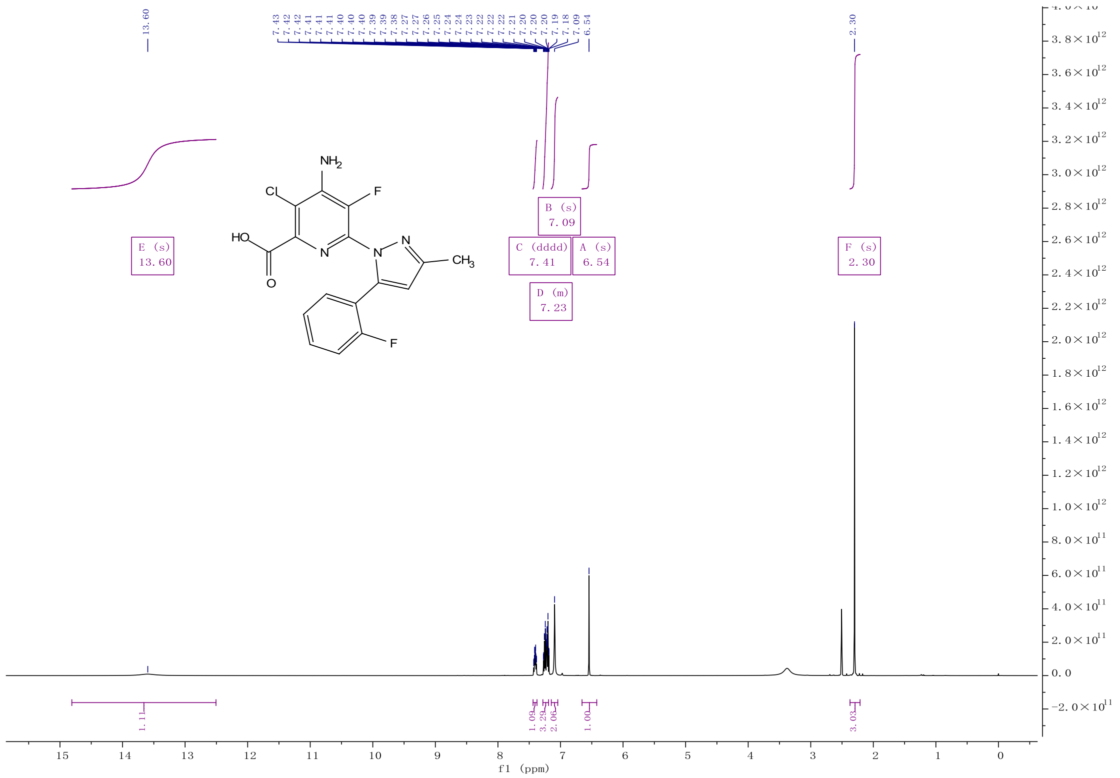

Figure S133. ^{1}H NMR (500.13 MHz, DMSO-d6) spectrum of compound S180.

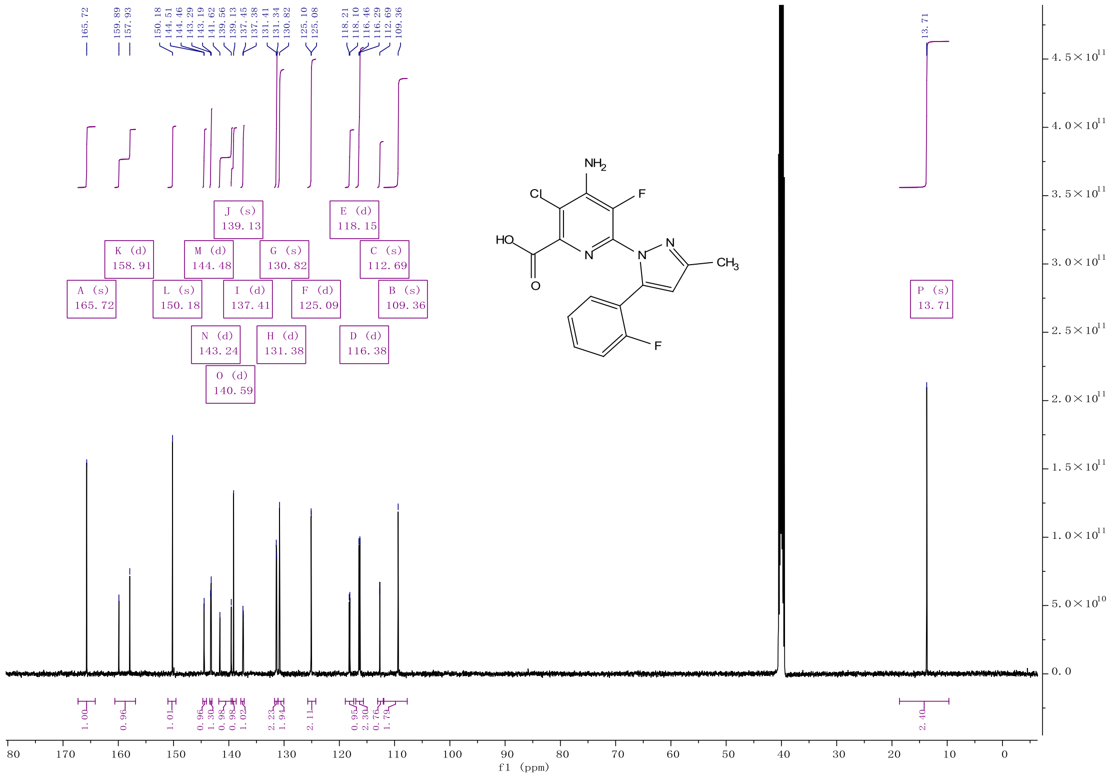

Figure S134. ^{13}C NMR (125.77 MHz, DMSO-d_6) spectrum of compound S180.

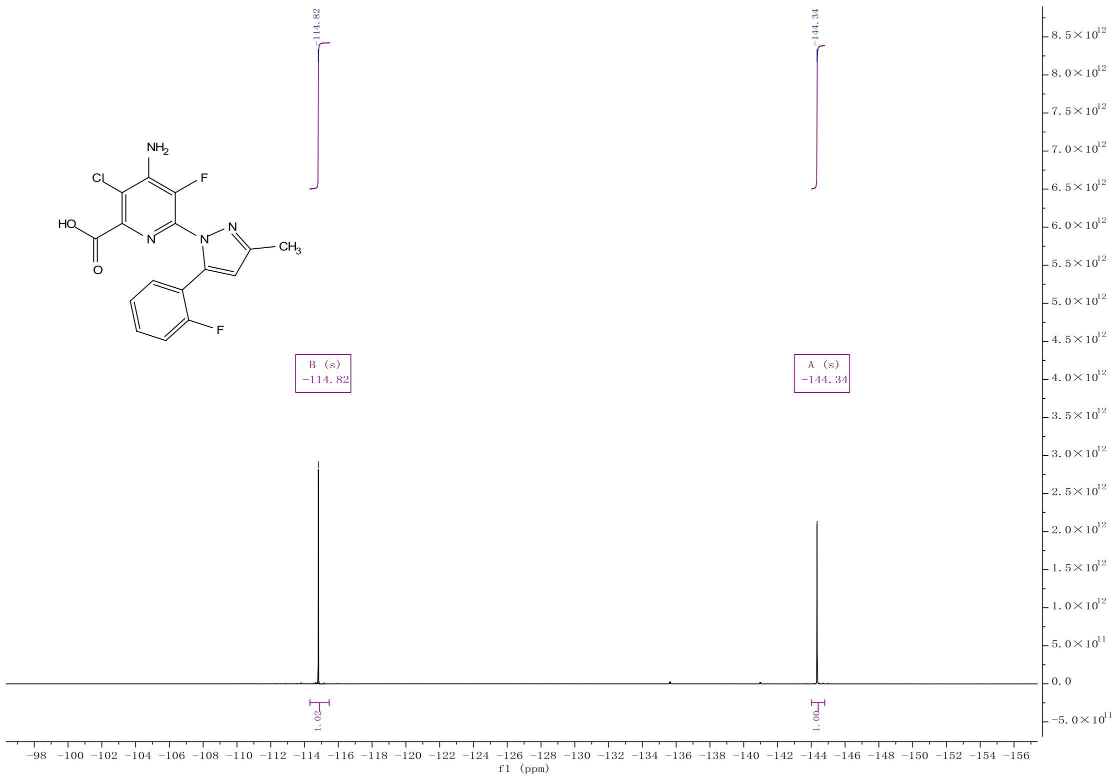

Figure S135. ^{19}F NMR (470.54 MHz, DMSO-d6) spectrum of compound S180.

Single Mass Analysis
Tolerance = 5.0 mDa / DBE: min = -1.5, max = 50.0
Element prediction: Off
Number of isotope peaks used for i-FIT = 3

Monoisotopic Mass, Even Electron Ions
4128 formula(e) evaluated with 1 results within limits (up to 50 best isotopic matches for each mass)
Elements Used:
C: 16-16 H: 12-12 N: 0-50 O: 0-50 F: 1-4 Na: 0-3 Cl: 1-2
4
250116-24-S180 36 (0.094) 1: TOF MS ES+ 8.46e+004

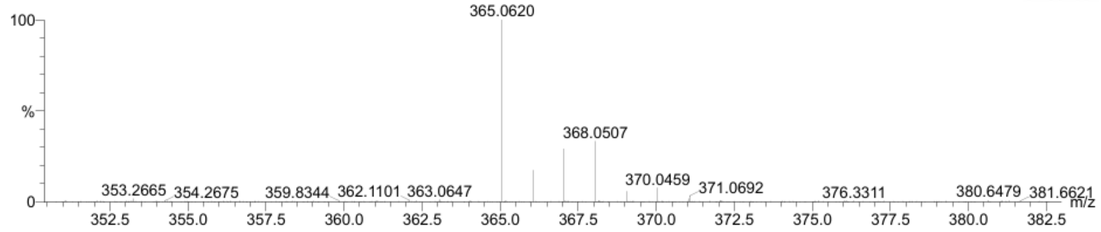

Minimum:				-1.5				
Maximum:		5.0	10.0	50.0				
Mass	Calc. Mass	mDa	PPM	DBE	i-FIT	Norm	Conf(%)	Formula
365.0620	365.0617	0.3	0.8	11.5	258.5	n/a	n/a	C16 H12 N4 O2 F2 Cl

Figure S136. HRMS spectrum of compound S180.

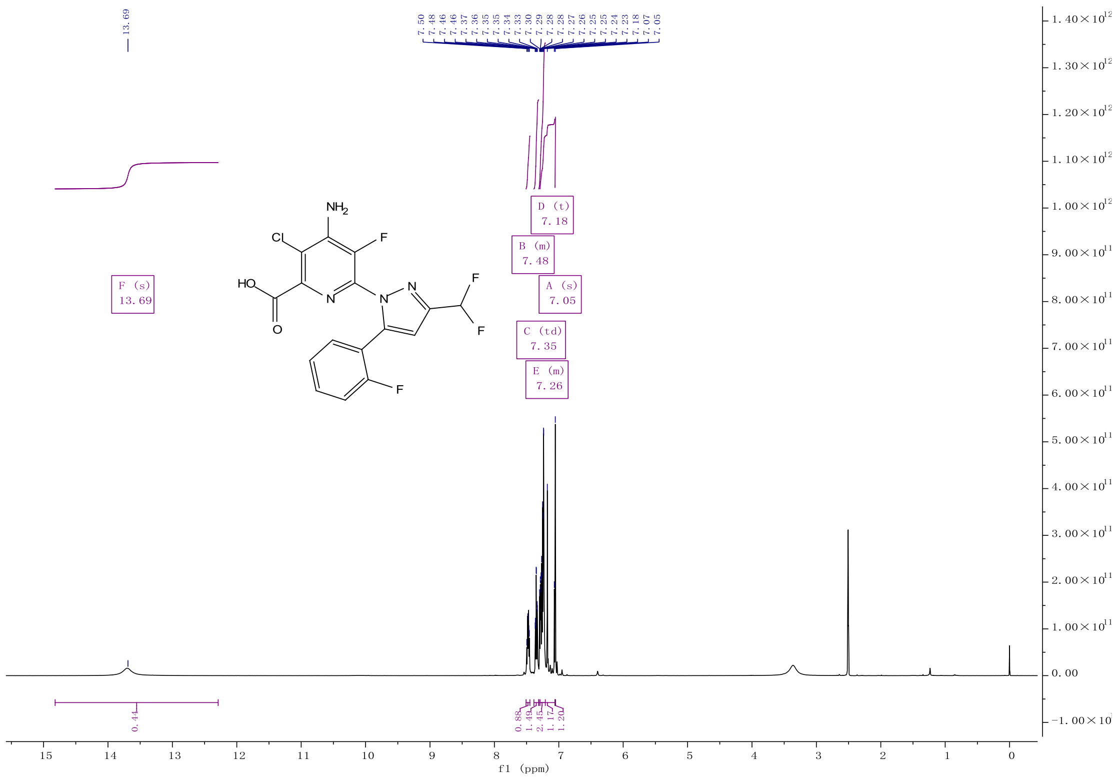

Figure S137. ^{1}H NMR (500.13 MHz, DMSO-d6) spectrum of compound S182.

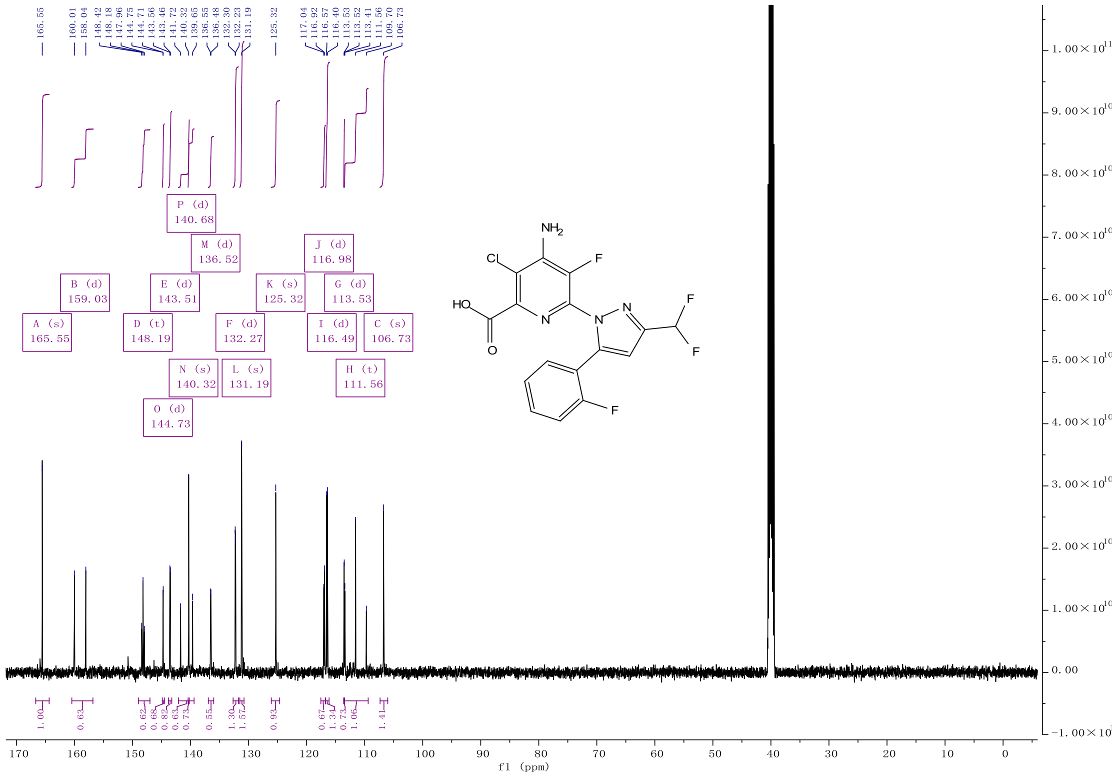

Figure S138. ^{13}C NMR (125.77 MHz, DMSO-d_6) spectrum of compound S182.

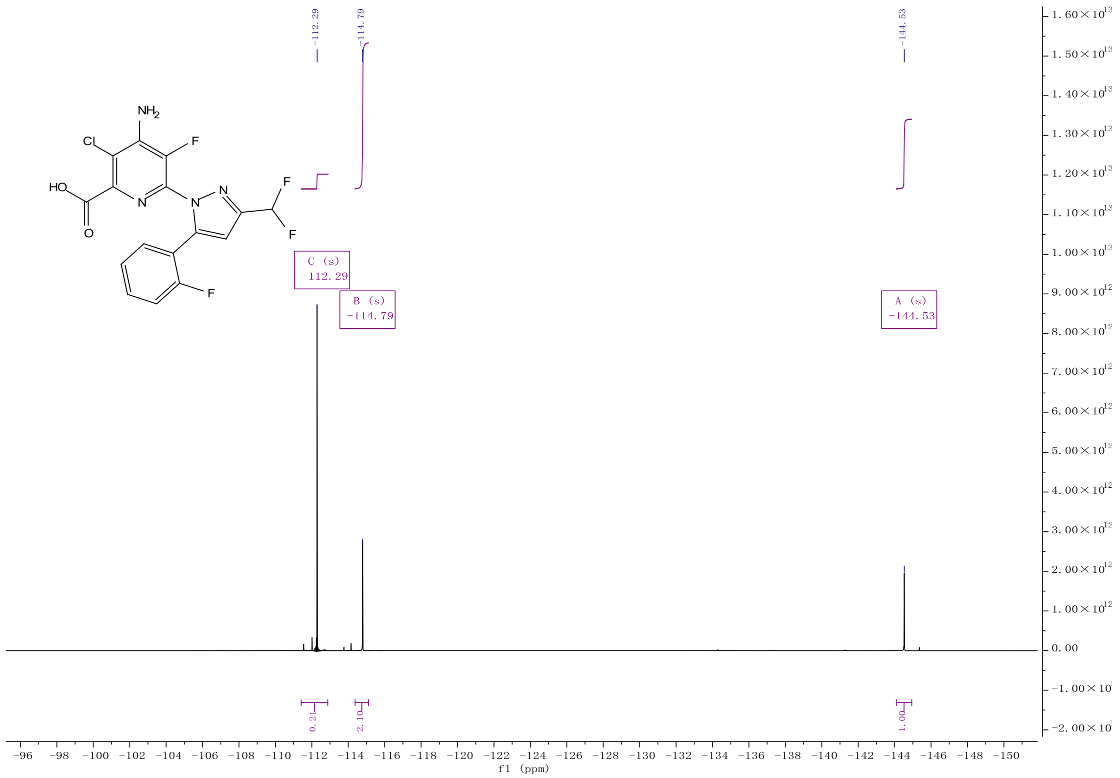

Figure S139. ^{19}F NMR (470.54 MHz, DMSO-d6) spectrum of compound S182.

Single Mass Analysis
Tolerance = 5.0 mDa / DBE: min = -1.5, max = 50.0
Element prediction: Off
Number of isotope peaks used for i-FIT = 3

Monoisotopic Mass, Even Electron Ions
6003 formula(e) evaluated with 1 results within limits (up to 50 best isotopic matches for each mass)
Elements Used:
C: 16-16 H: 9-9 N: 0-50 O: 0-50 F: 1-4 Na: 0-3 Cl: 1-2
28
250116-24-S182 44 (0.109)

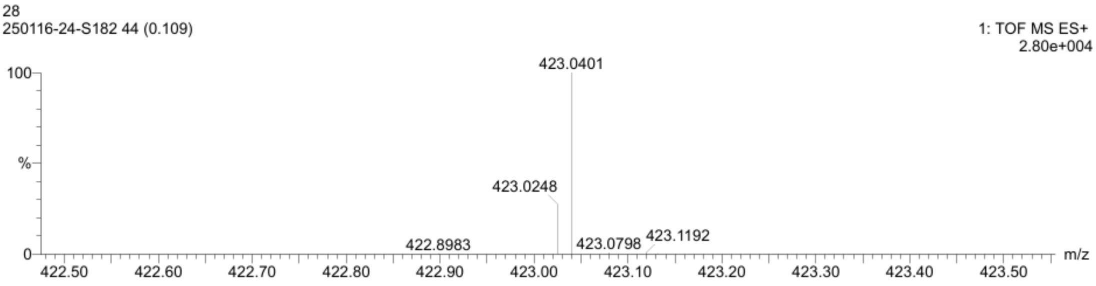

Minimum:				-1.5				
Maximum:		5.0	10.0	50.0				

Mass	Calc. Mass	mDa	PPM	DBE	i-FIT	Norm	Conf(%)	Formula
423.0248	423.0248	0.0	0.0	11.5	74.4	n/a	n/a	C16 H9 N4 O2 F4 Na Cl

Figure S140. HRMS spectrum of compound S182.

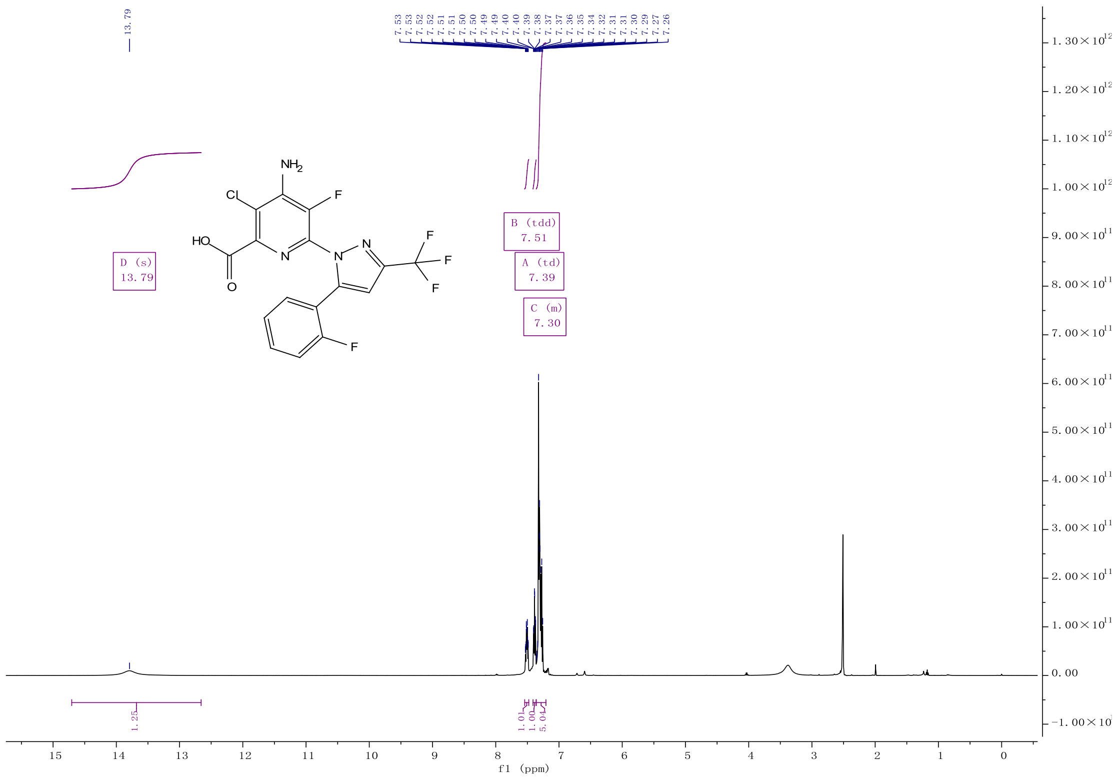

Figure S141. ^{1}H NMR (500.13 MHz, DMSO-d6) spectrum of compound S183.

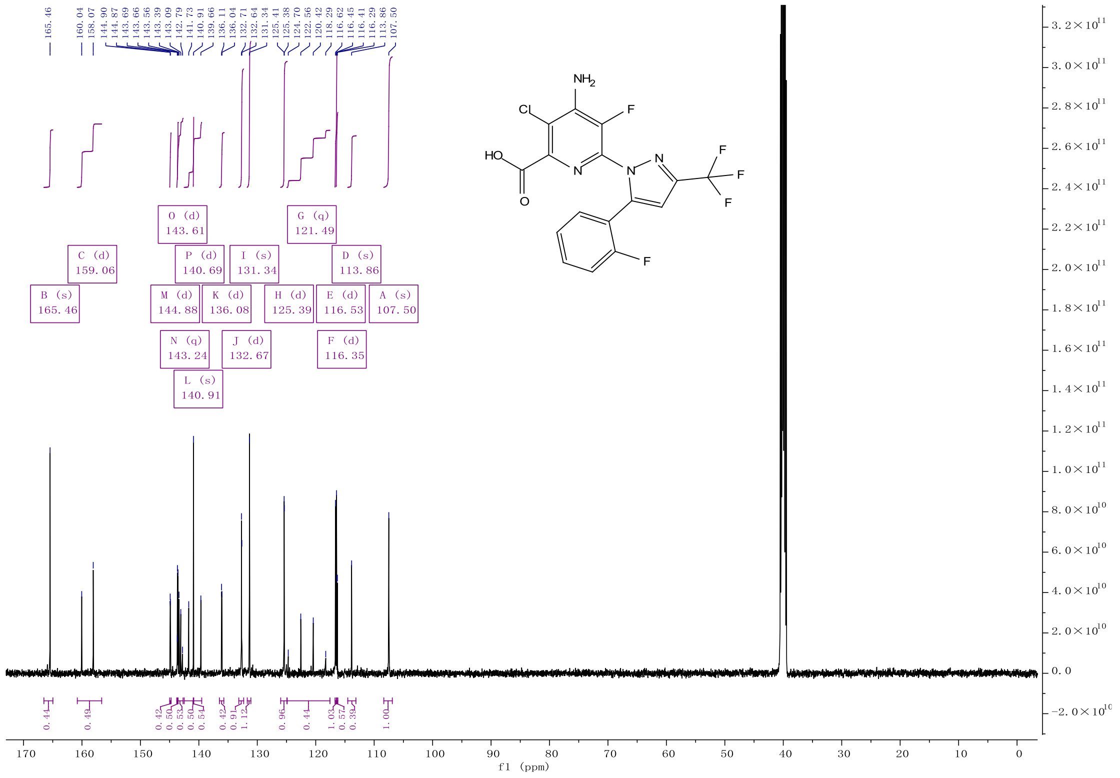

Figure S142. ^{13}C NMR (125.77 MHz, DMSO-d_6) spectrum of compound S183.

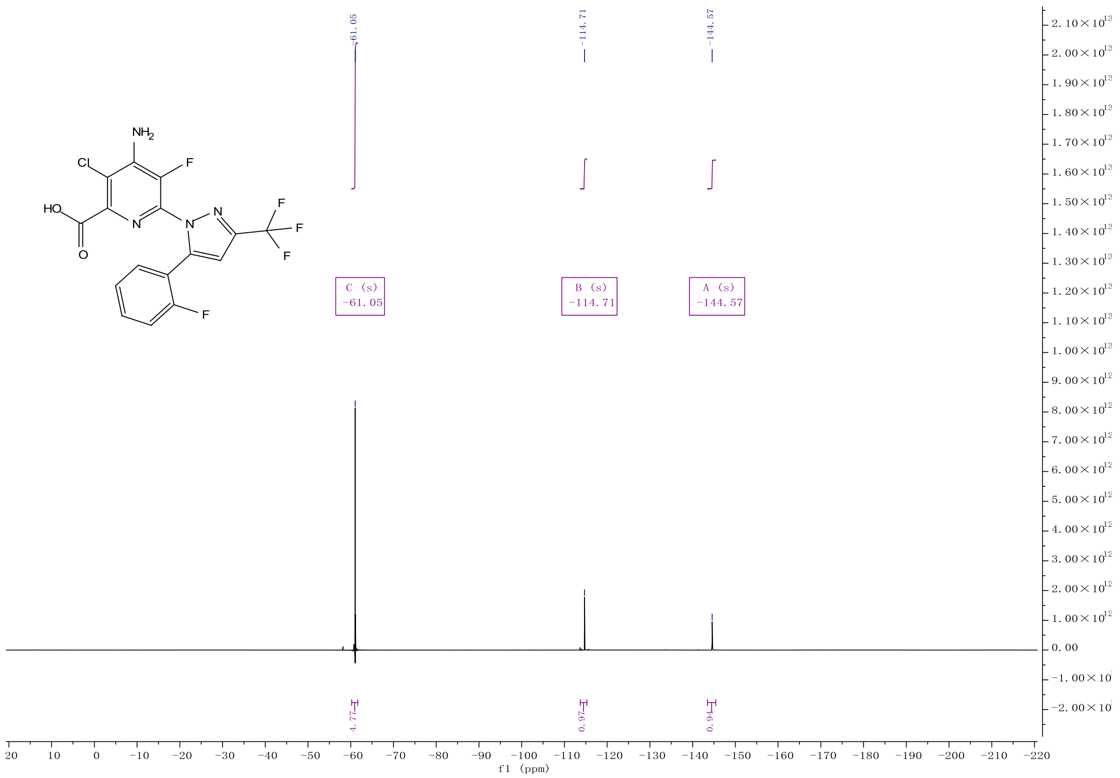

Figure S143. ^{19}F NMR (470.54 MHz, DMSO-d6) spectrum of compound S183.

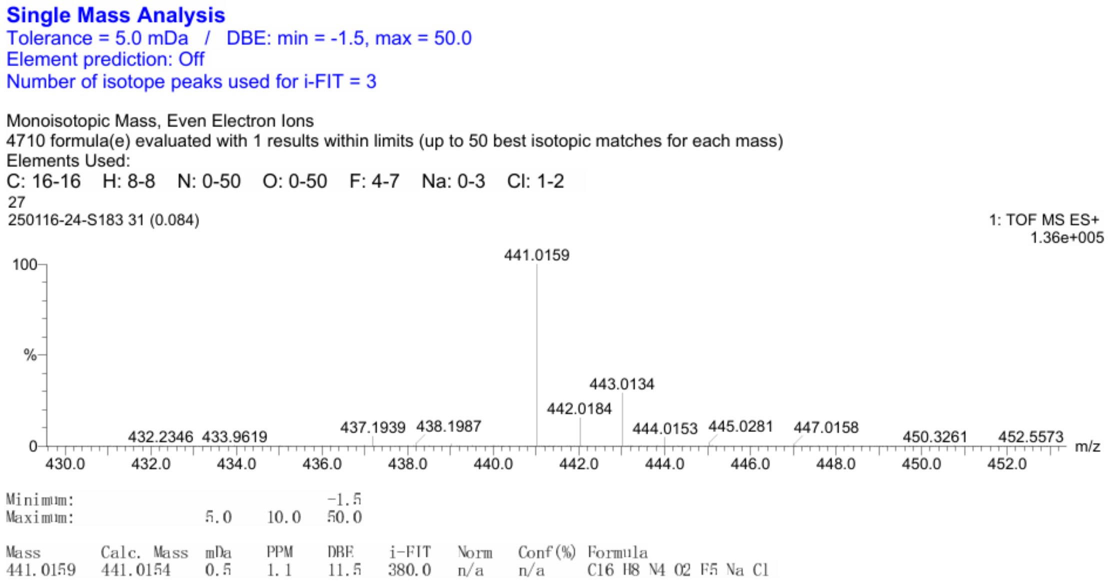

Figure S144. HRMS spectrum of compound S183.

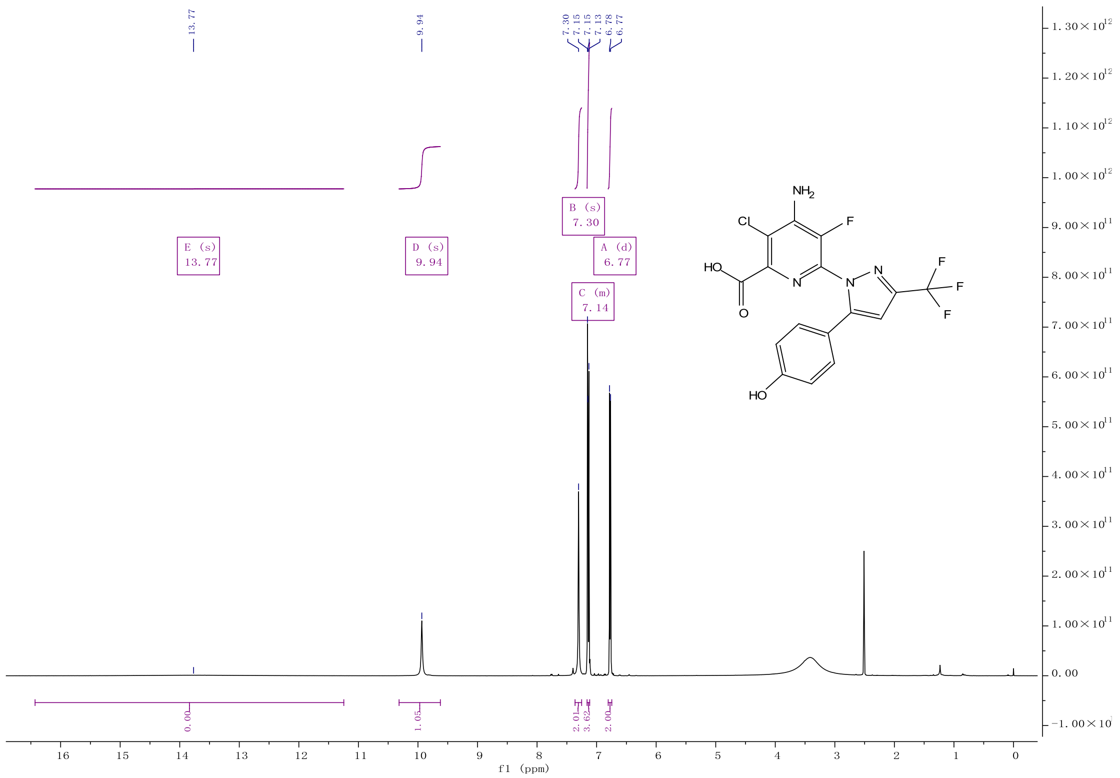

Figure S145. ^{1}H NMR (500.13 MHz, DMSO-d6) spectrum of compound S313.

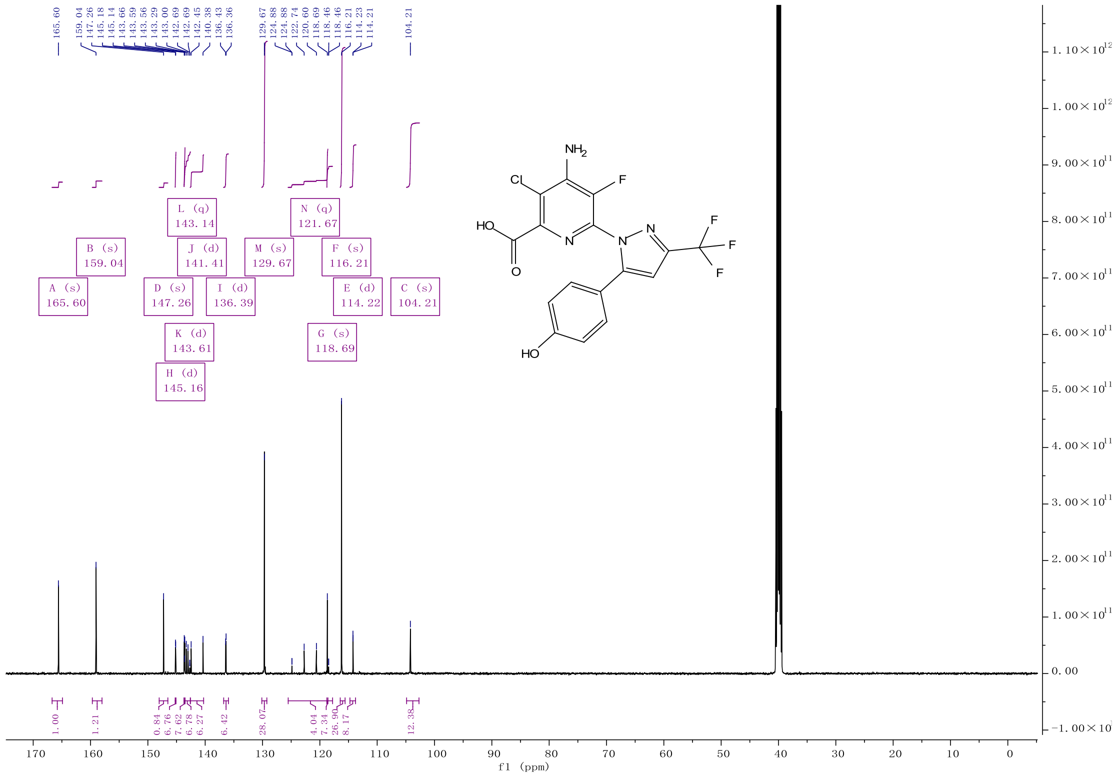

Figure S146. ^{13}C NMR (125.77 MHz, DMSO-d_6) spectrum of compound S313.

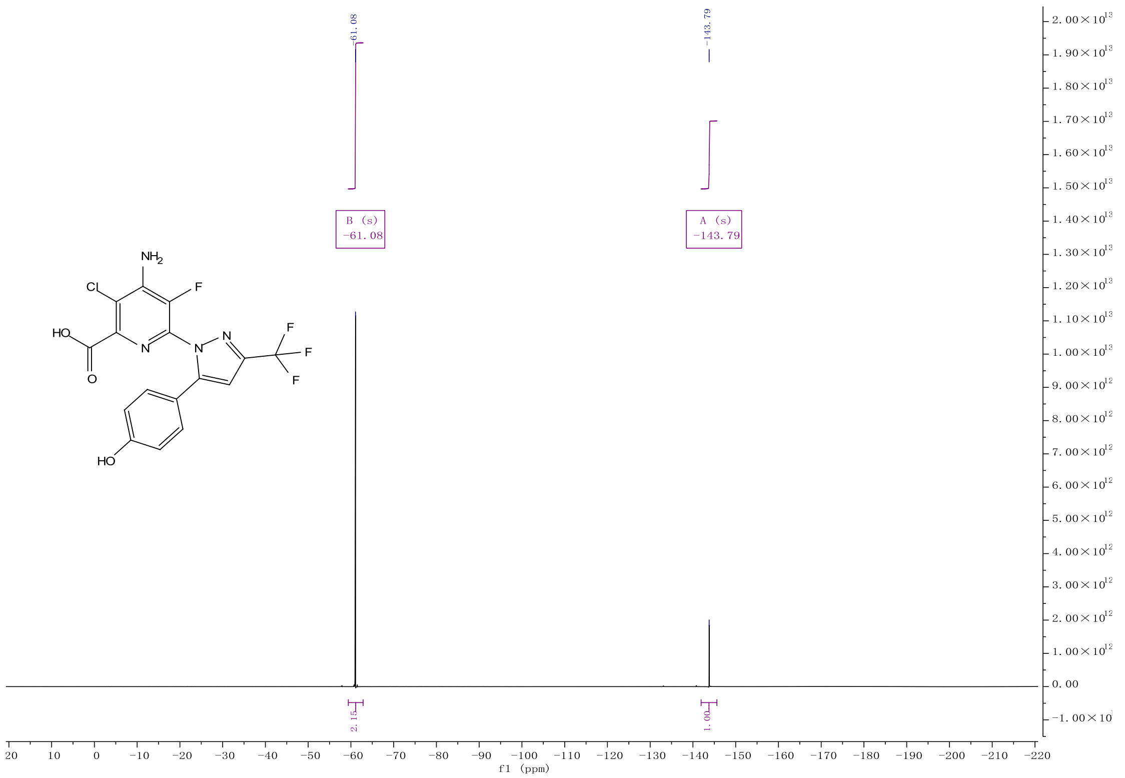

Figure S147. ^{19}F NMR (470.54 MHz, DMSO-d6) spectrum of compound S313.

Single Mass Analysis
Tolerance = 5.0 mDa / DBE: min = -1.5, max = 50.0
Element prediction: Off
Number of isotope peaks used for i-FIT = 3

Monoisotopic Mass, Even Electron Ions
6595 formula(e) evaluated with 1 results within limits (up to 50 best isotopic matches for each mass)
Elements Used:
C: 16-16 H: 9-9 N: 0-50 O: 0-50 F: 1-4 Na: 0-3 Cl: 1-2
27
250116-24-S313 33 (0.088)

1: TOF MS ES+
1.46e+005

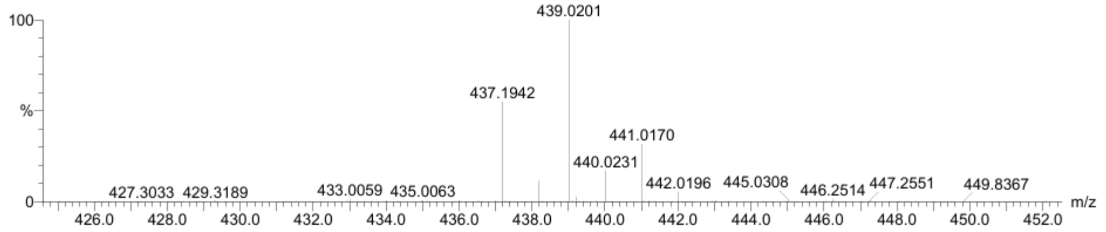

Minimum:				-1.5				
Maximum:		5.0	10.0	50.0				
Mass	Calc. Mass	mDa	PPM	DBE	i-FIT	Norm	Conf(%)	Formula
439.0201	439.0197	0.4	0.9	11.5	432.5	n/a	n/a	C16 H9 N4 O3 F4 Na Cl

Figure S148. HRMS spectrum of compound S313.

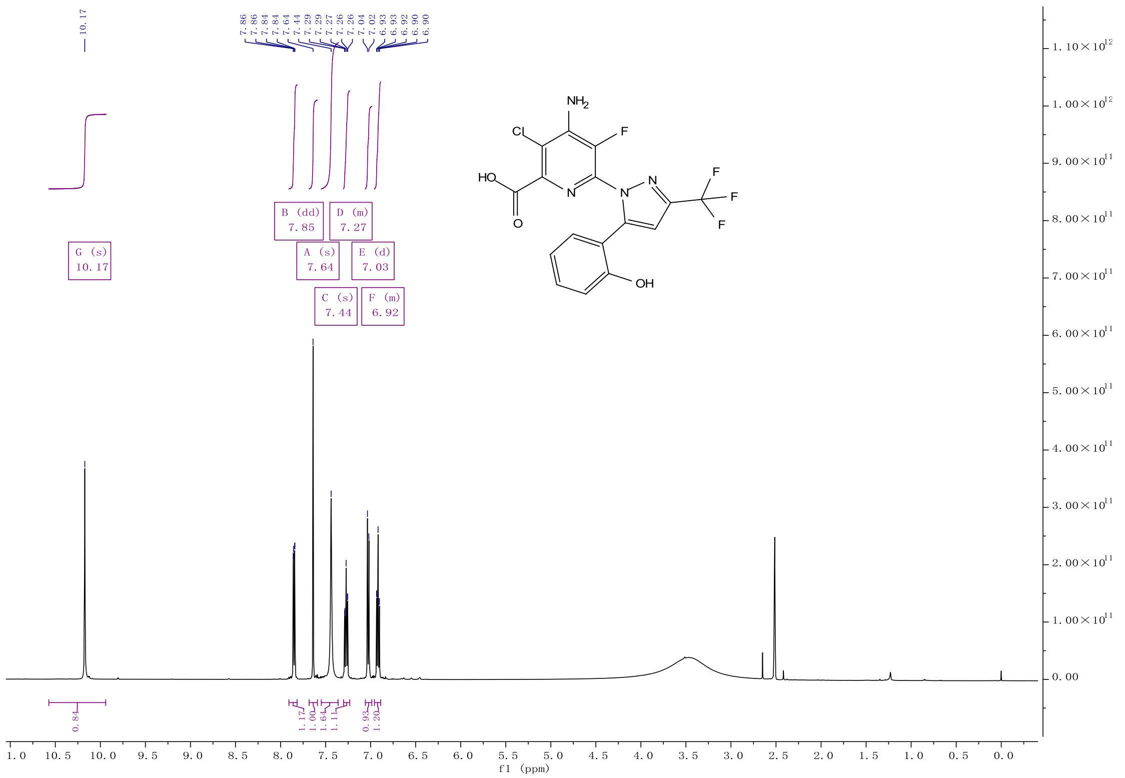

Figure S149. ^{1}H NMR (500.13 MHz, DMSO-d6) spectrum of compound S333.

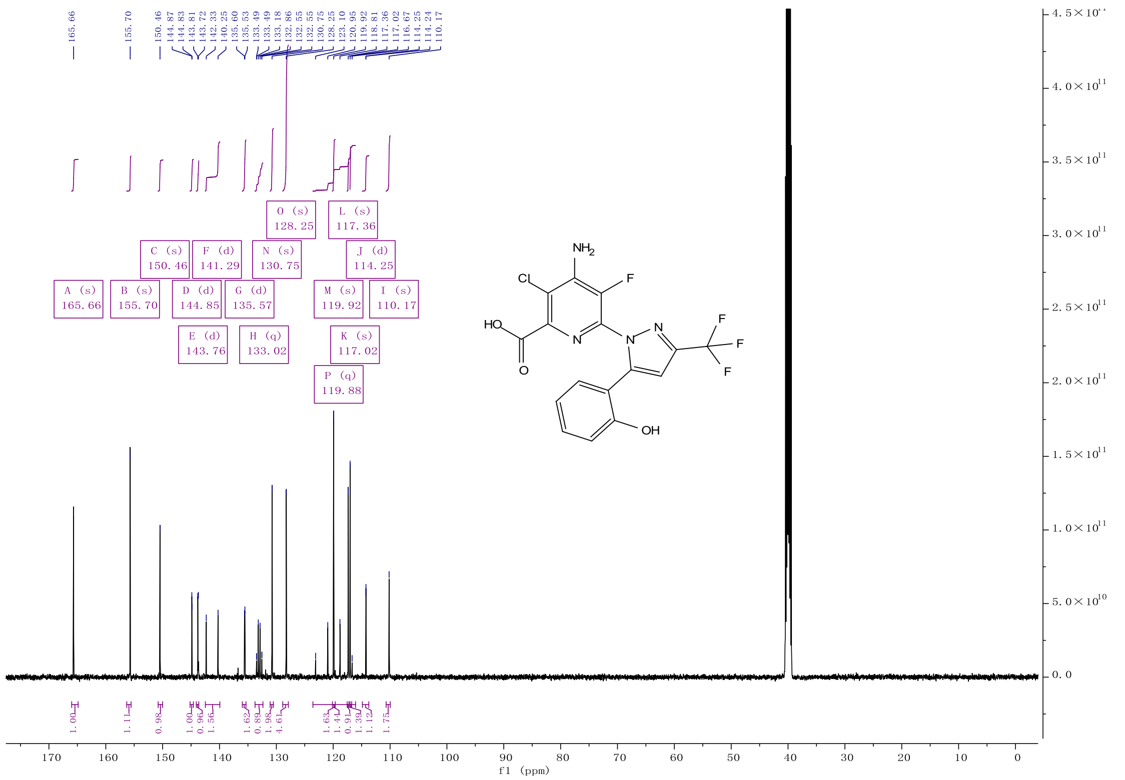

Figure S150. ^{13}C NMR (125.77 MHz, DMSO-d_6) spectrum of compound S333.

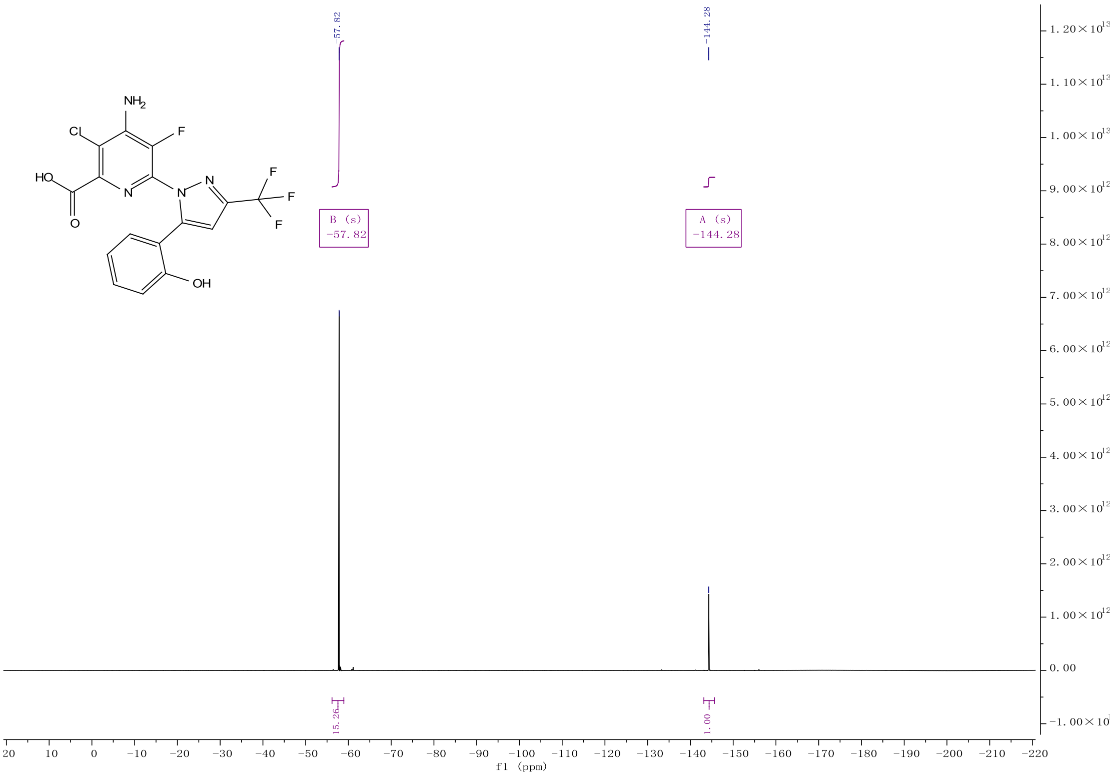

Figure S151. ^{19}F NMR (470.54 MHz, DMSO-d6) spectrum of compound S333.

Single Mass Analysis
Tolerance = 5.0 mDa / DBE: min = -1.5, max = 50.0
Element prediction: Off
Number of isotope peaks used for i-FIT = 3

Monoisotopic Mass, Even Electron Ions
8904 formula(e) evaluated with 1 results within limits (up to 50 best isotopic matches for each mass)
Elements Used:
C: 16-16 H: 9-9 N: 0-50 O: 0-50 F: 1-6 Na: 0-3 Cl: 1-2
27
250116-24-S333 27 (0.076)

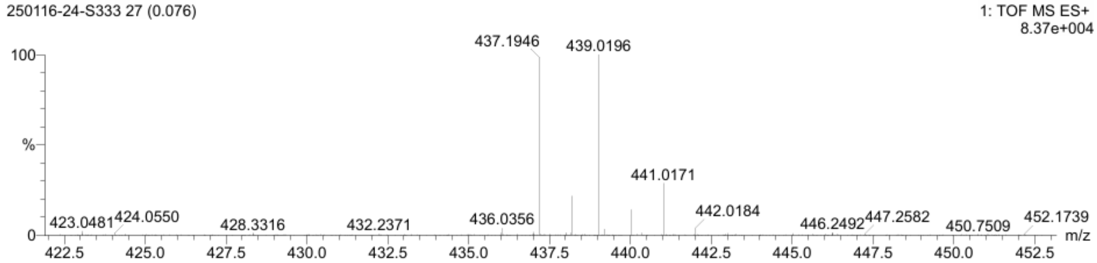

Minimum:				-1.5				
Maximum:		5.0	10.0	50.0				
Mass	Calc. Mass	mDa	PPM	DBE	i-FIT	Norm	Conf(%)	Formula
439.0196	439.0197	-0.1	-0.2	11.5	313.4	n/a	n/a	C16 H9 N4 O3 F4 Na Cl

Figure S152. HRMS spectrum of compound S333.

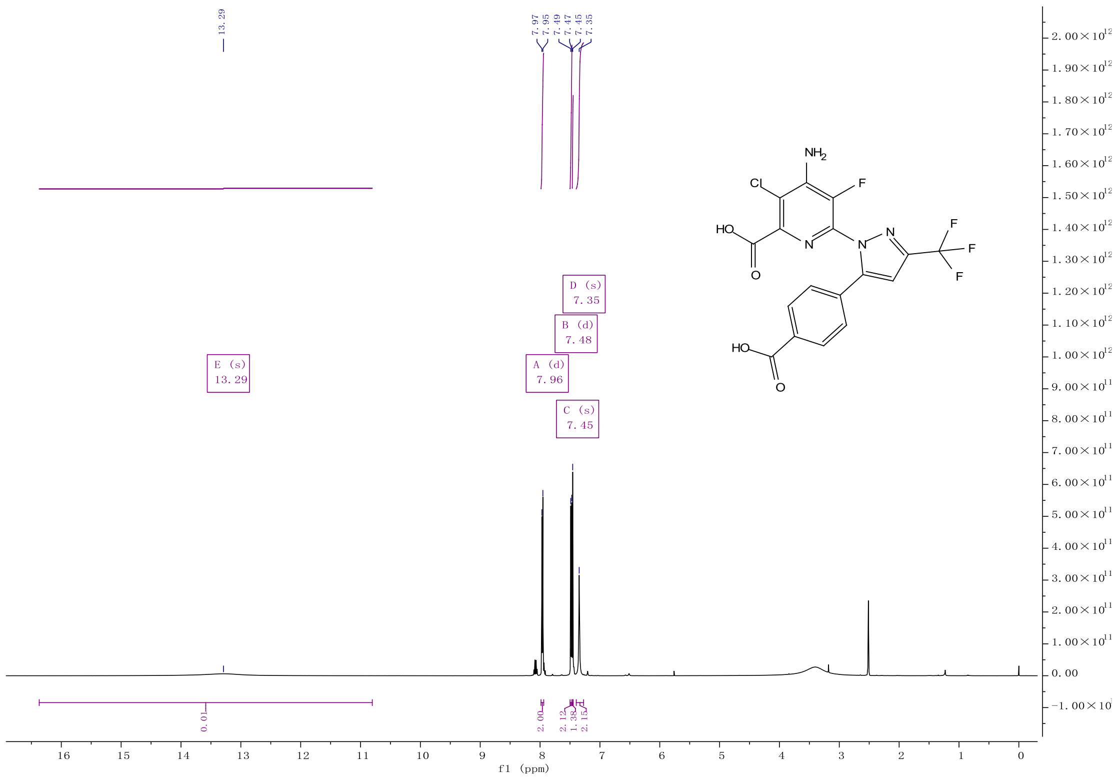

Figure S153. ^{1}H NMR (500.13 MHz, DMSO-d6) spectrum of compound S343.

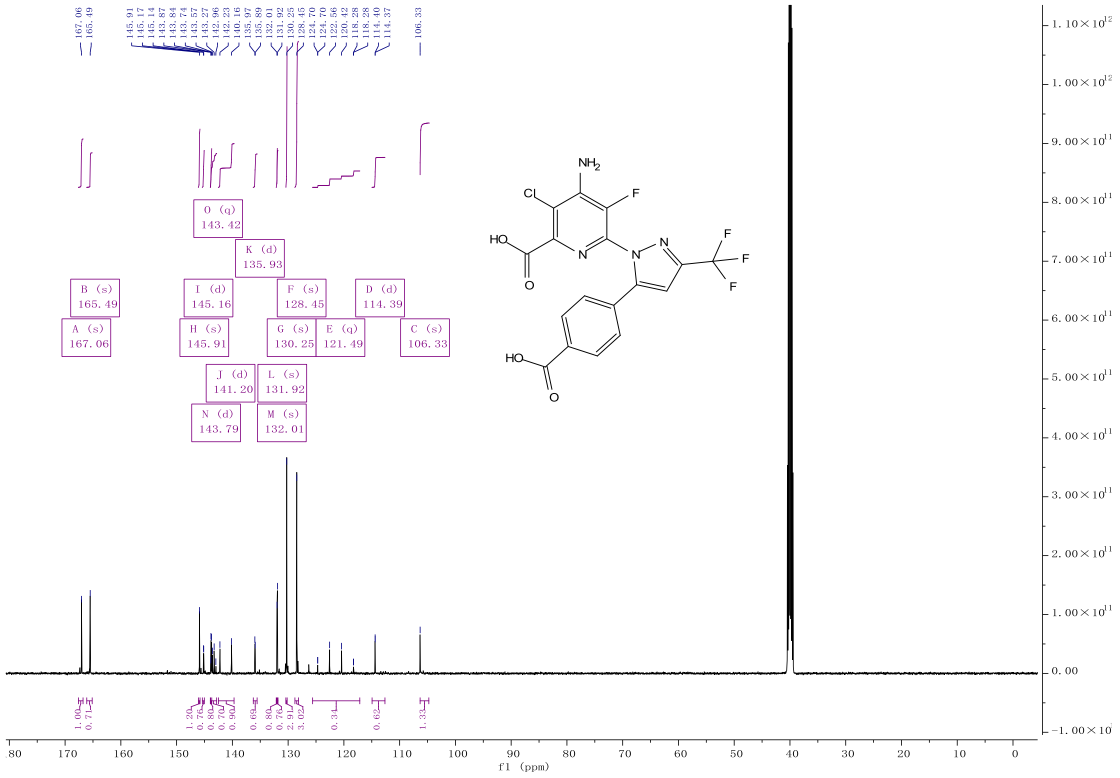

Figure S154. ^{13}C NMR (125.77 MHz, DMSO-d_6) spectrum of compound S343.

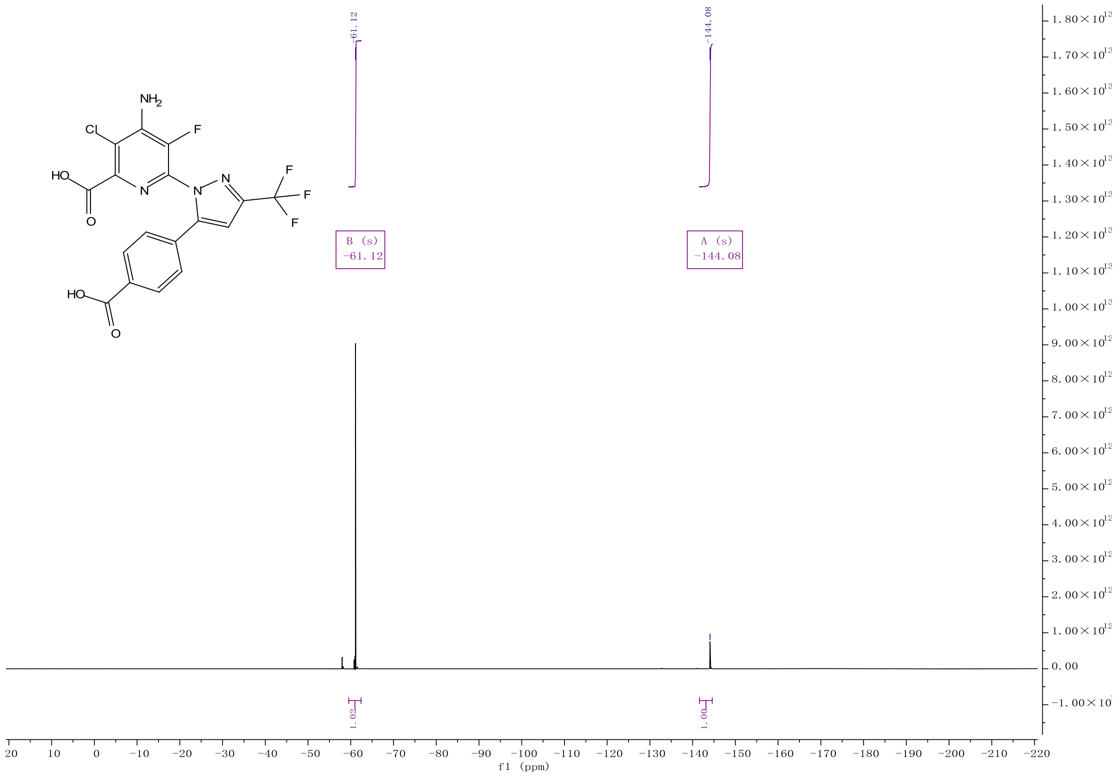

Figure S155. ^{19}F NMR (470.54 MHz, DMSO-d6) spectrum of compound S343.

Single Mass Analysis
Tolerance = 5.0 mDa / DBE: min = -1.5, max = 50.0
Element prediction: Off
Number of isotope peaks used for i-FIT = 3

Monoisotopic Mass, Even Electron Ions
4974 formula(e) evaluated with 1 results within limits (up to 50 best isotopic matches for each mass)
Elements Used:
C: 17-17 H: 9-9 N: 0-50 O: 0-50 F: 3-5 Na: 0-3 Cl: 1-2
4
250116-24-S343 36 (0.094) 1: TOF MS ES+ 3.66e+004

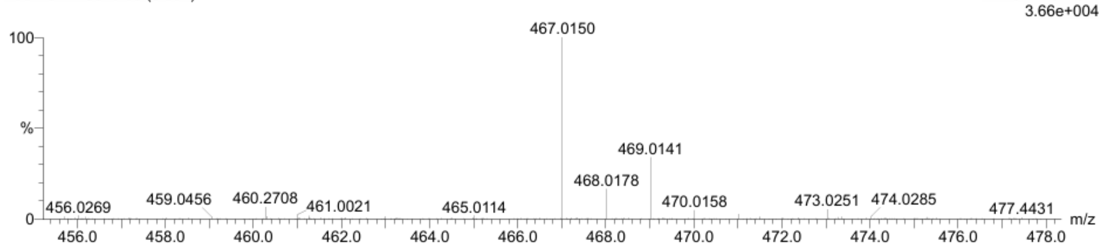

Minimum:				-1.5				
Maximum:		5.0	10.0	50.0				
Mass	Calc. Mass	mDa	PPM	DBE	i-FIT	Norm	Conf(%)	Formula
467.0150	467.0146	0.4	0.9	12.5	286.4	n/a	n/a	C17 H9 N4 O4 F4 Na Cl

Figure S156. HRMS spectrum of compound S343.

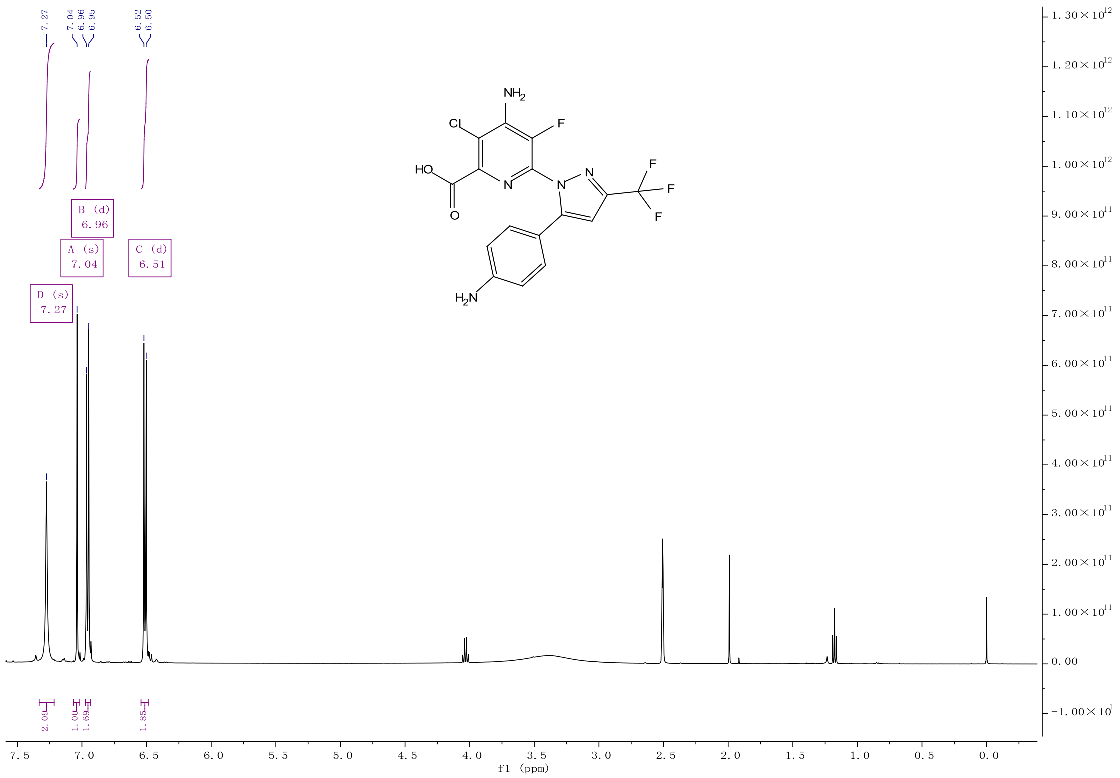

Figure S157. ^{1}H NMR (500.13 MHz, DMSO-d6) spectrum of compound S113.

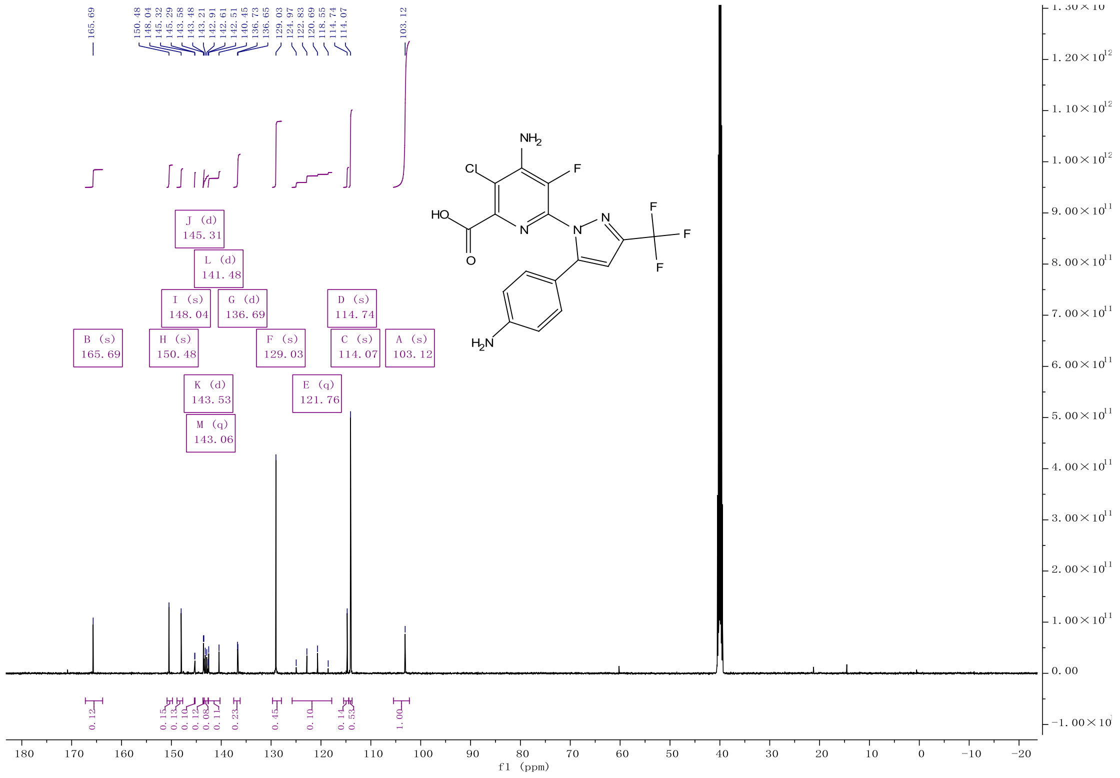

Figure S158. ^{13}C NMR (125.77 MHz, DMSO-d_6) spectrum of compound S113.

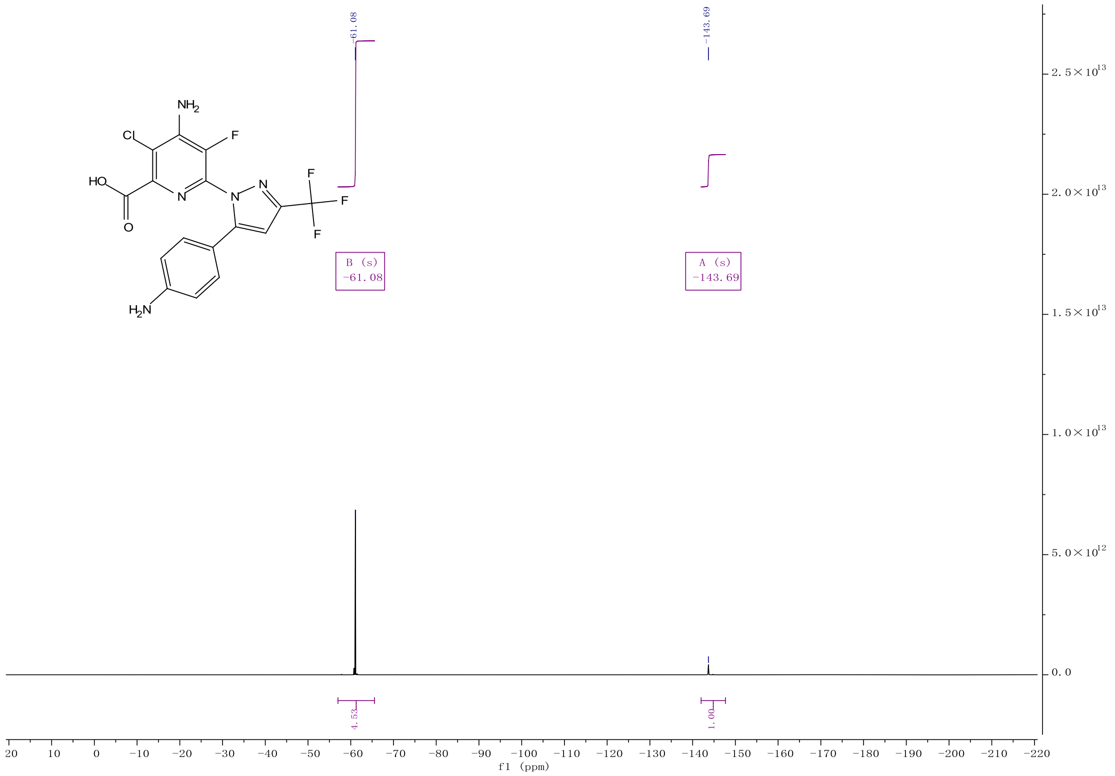

Figure S159. ^{19}F NMR (470.54 MHz, DMSO-d6) spectrum of compound S113.

Single Mass Analysis
Tolerance = 5.0 mDa / DBE: min = -1.5, max = 50.0
Element prediction: Off
Number of isotope peaks used for i-FIT = 3

Monoisotopic Mass, Even Electron Ions
3957 formula(e) evaluated with 1 results within limits (up to 50 best isotopic matches for each mass)
Elements Used:
C: 16-16 H: 11-11 N: 0-50 O: 0-50 F: 4-7 Na: 0-3 Cl: 1-2
27
250116-24-S113 56 (0.132)

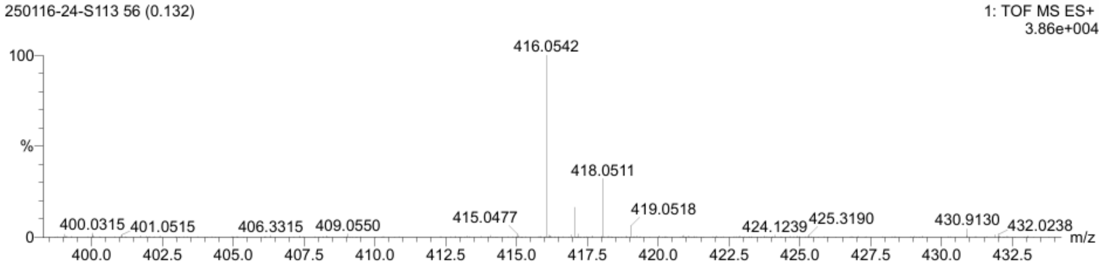

Minimum:				-1.5				
Maximum:		5.0	10.0	50.0				
Mass	Calc. Mass	mDa	PPM	DBE	i-FIT	Norm	Conf(%)	Formula
416.0542	416.0537	0.5	1.2	11.5	262.2	n/a	n/a	C16 H11 N5 O2 F4 Cl

Figure S160. HRMS spectrum of compound S113.

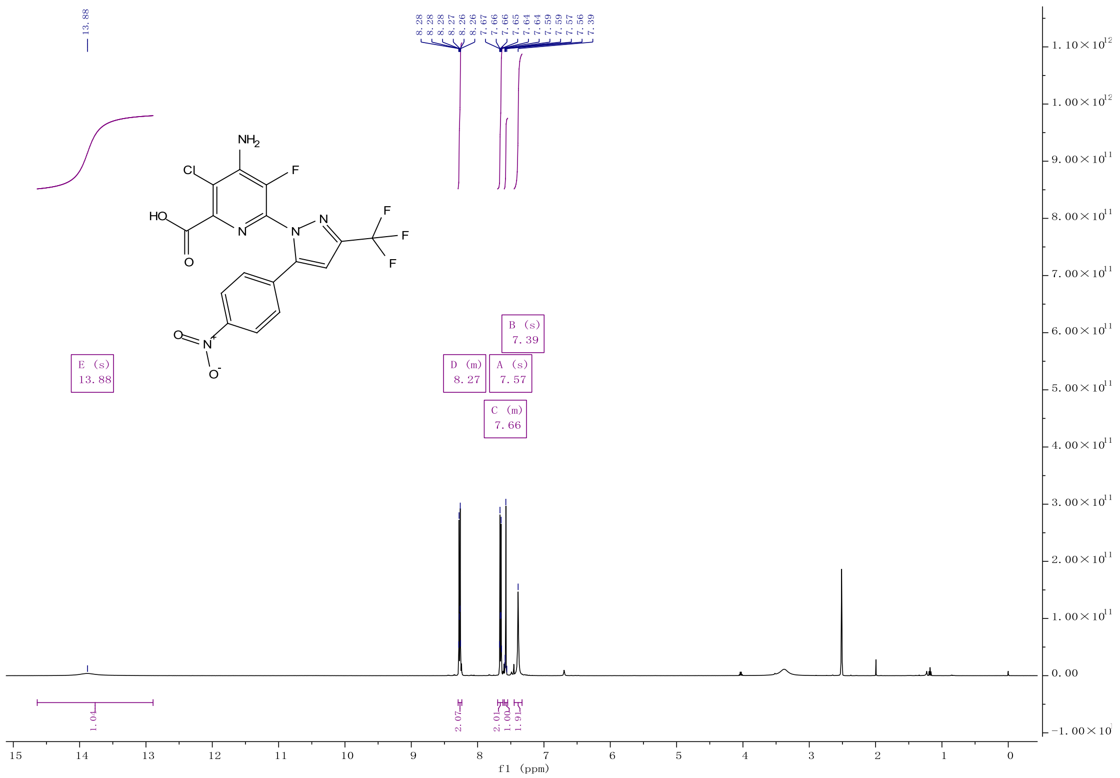

Figure S161. ^{1}H NMR (500.13 MHz, DMSO-d6) spectrum of compound S233.

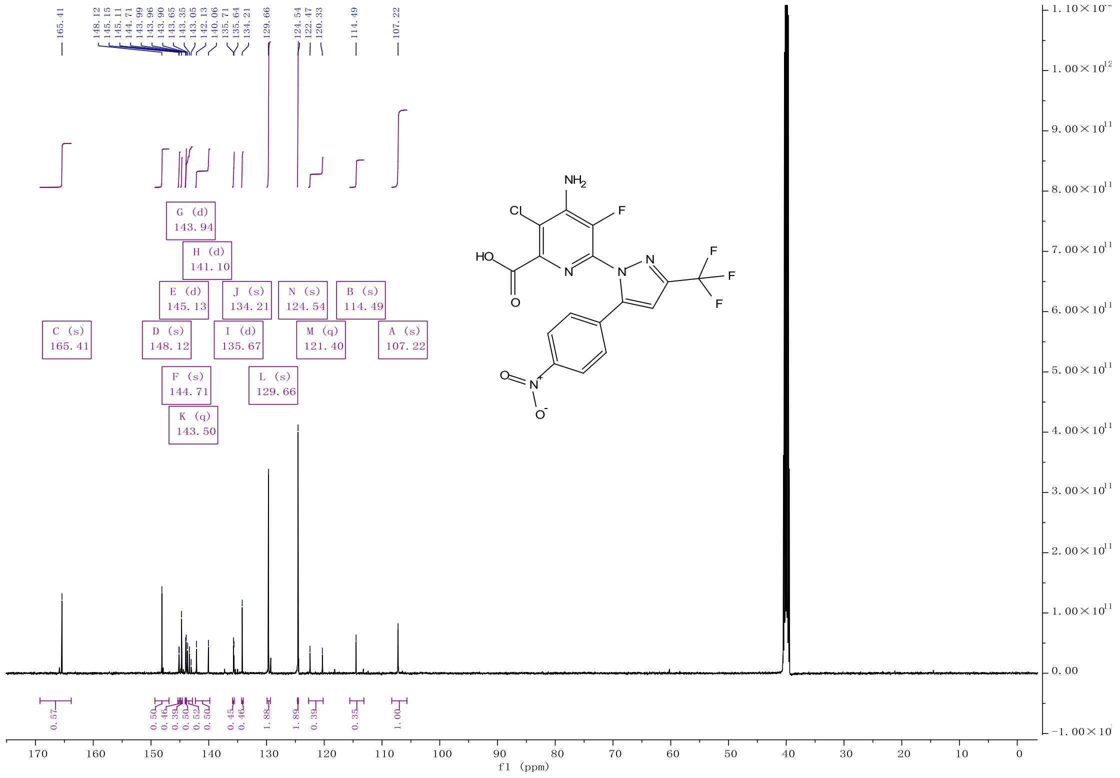

Figure S162. ^{13}C NMR (125.77 MHz, DMSO-d_6) spectrum of compound S233.

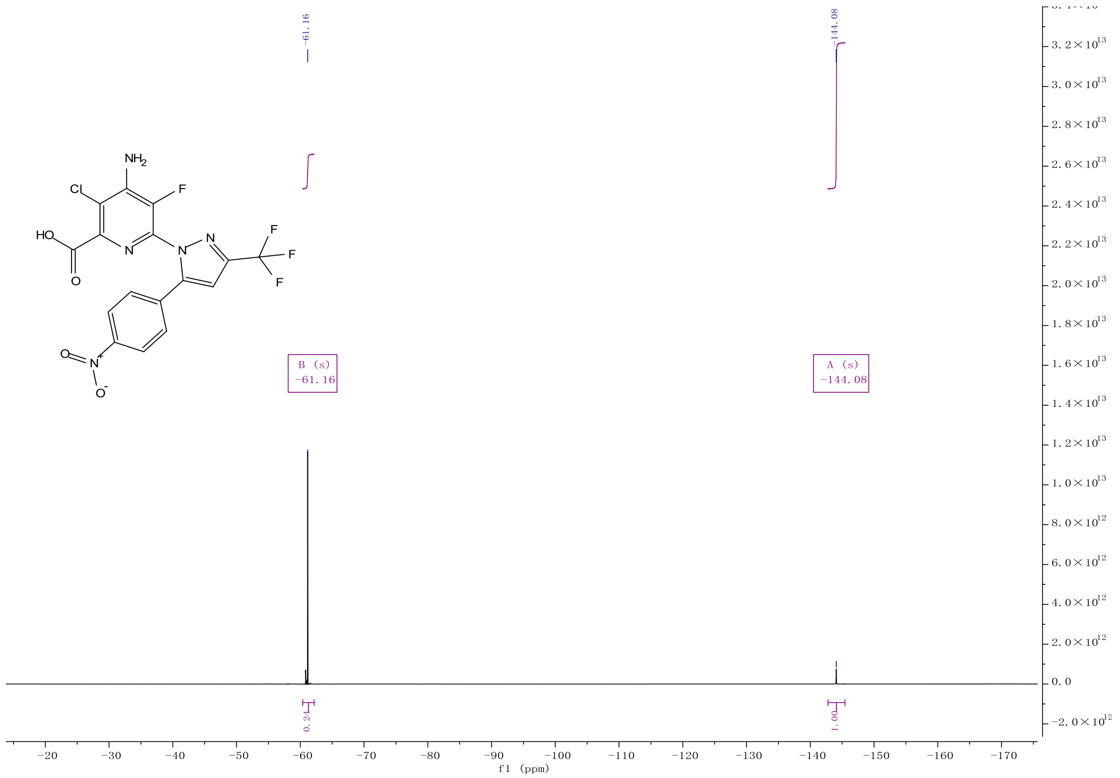

Figure S163. ^{19}F NMR (470.54 MHz, DMSO-d6) spectrum of compound S233.

Single Mass Analysis
Tolerance = 5.0 mDa / DBE: min = -1.5, max = 50.0
Element prediction: Off
Number of isotope peaks used for i-FIT = 3

Monoisotopic Mass, Even Electron Ions
9283 formula(e) evaluated with 1 results within limits (up to 50 best isotopic matches for each mass)
Elements Used:
C: 16-16 H: 9-9 N: 0-50 O: 0-50 F: 1-6 Na: 0-3 Cl: 1-2
27
250116-24-S233 28 (0.078)

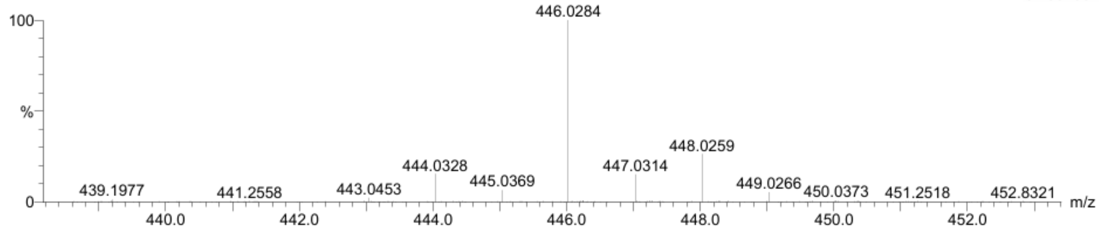

Minimum:			-1.5
Maximum:	5.0	10.0	50.0

Mass	Calc. Mass	mDa	PPM	DBE	i-FIT	Norm	Conf(%)	Formula
446.0284	446.0279	0.5	1.1	12.5	343.8	n/a	n/a	C16 H9 N5 O4 F4 Cl

Figure S164. HRMS spectrum of compound S233.

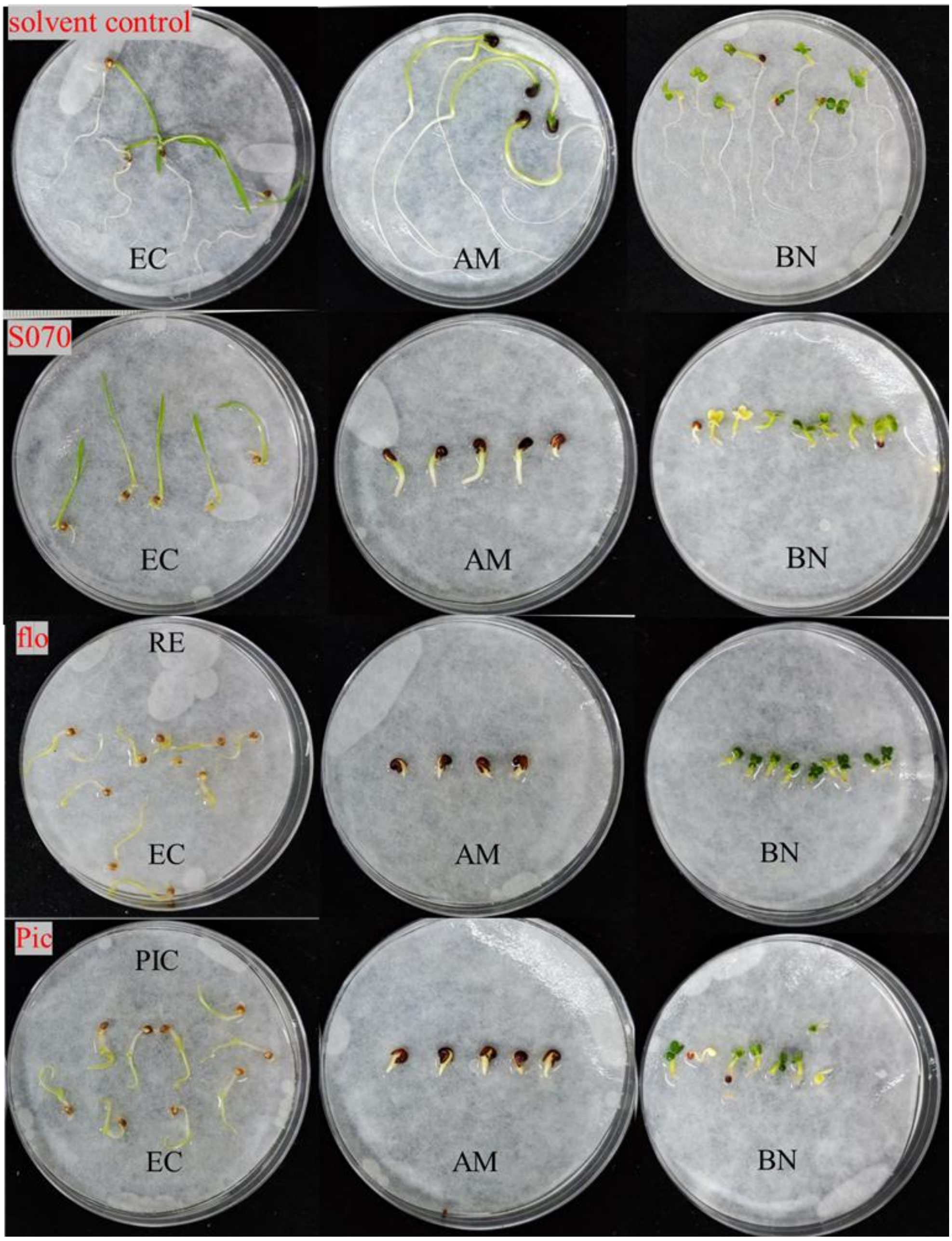

Figure S165. The inhibitory effects of compound S070, solvent control and control drug, on three types of weed seeds at the concentration of 250 μM
